# Global burden of leukemia in women of child-bearing age, 1990 to 2021: An update from the Global Burden of Disease Study 2021

**DOI:** 10.1097/MD.0000000000047217

**Published:** 2026-01-23

**Authors:** Xing-Biao Chen, Zhe-Han Yang, Wei-Jun Ling, Qi-Jian Feng, Zi-Yuan Lu

**Affiliations:** a Department of Hematology, The Third Affiliated Hospital, Guangzhou Medical University, Guangzhou, China; b Guangdong Provincial Key Laboratory of Major Obstetric Diseases, Guangzhou, China; c Guangdong Provincial Clinical Research Center for Obstetrics and Gynecology, Guangzhou, China; d Department of Clinical Medicine, Guangzhou Medical University, Guangzhou, China; e Dongguan University of Technology-Conservatoire National des Arts er Metrers (DGUT-CNAM) Institute, Dongguan University of Technology, Dongguan, China; f Department of Endocrinology, Zhujiang Hospital, Southern Medical University, Guangzhou, China.

**Keywords:** ALL, AML, CLL, CML, Global Burden of Disease Study, women of child-bearing age

## Abstract

While the overall burden of leukemia has been studied, a comprehensive and up-to-date epidemiological analysis specifically focused on women of childbearing age (WCBA) using the latest global burden of disease 2021 data, particularly detailing the 4 major subtypes, is lacking. This knowledge gap limits the development of targeted healthcare policies for this unique demographic. This study aims to fill this gap by assessing the global, regional, and national burden of leukemia in WCBA from 1990 to 2021. We collected and analyzed data on acute lymphoblastic leukemia (ALL), acute myeloid leukemia (AML), chronic lymphocytic leukemia, and chronic myeloid leukemia (CML) in WCBA (aged 15–49) from the Global Burden of Disease Study 2021. We evaluated incidence, mortality, and disability-adjusted life years (DALYs), along with their age-standardized rates, categorized by age, region, country, and socio-demographic index. We also analyzed risk factors associated with leukemia-related DALYs and mortality. In 2021, an estimated 40,200 (95% UI 31.3, 45.1) new cases of leukemia among WCBA were reported globally. From 1990 to 2021, the age-standardized incidence rate declined by 22.4% (95% UI–31.2,–11.4), with larger reductions in age-standardized death rate (–34.2%; 95% UI–40.5,–25.7) and DALY rate (–43.1%; 95% UI–50.6,–30.6). The annual disease burden, in descending order, were AML, ALL, CLL, and CML. Epidemiological patterns showed significant regional differences, though CML declined across all regions. The burden of AML, CLL, and CML was positively correlated with age, while ALL peaked in the youngest (15–19) group. Risk factor analysis identified obesity (high-income, middle-aged) and occupational exposures (younger) as significant contributors. While the overall leukemia burden in WCBA has decreased globally, significant disparities persist by subtype, age, and region, with AML remaining the dominant burden. These findings underscore the need for tailored public health strategies and resource allocation to address high-risk populations and specific leukemia subtypes.

## 1. Introduction

Leukemia is characterized by the malignant proliferation of hematopoietic stem cells in the bone marrow, leading to the accumulation of cancer cells that are arrested at various stages of development. These leukemia cells significantly impair normal hematopoietic production and manifest as symptoms such as anemia, bleeding, and increased susceptibility to infections. Based on the degree of differentiation and maturation of the leukemia cells, as well as the disease course, leukemia is categorized into acute and chronic leukemias. Acute leukemias can be further subdivided into acute myeloid leukemia (AML) and acute lymphoblastic leukemia (ALL), depending on the predominant cell lineage involved. Chronic leukemias mainly include chronic lymphocytic leukemia (CLL) and chronic myeloid leukemia (CML).^[1]^ Recent advancements in salvage chemotherapy, targeted therapy, hematopoietic stem cell transplantation, and combined treatment modalities have the potential to enhance the treatment of these diseases. However, they also contribute to the financial burden on already limited healthcare resources.^[2–5]^

Each type of leukemia exhibits a distinct epidemiological pattern. Previous studies analyzing global burden of disease (GBD) data have provided comprehensive overviews of specific subtypes, such as AML, noting global increases in incidence and mortality, though with varying impacts related to the socio-demographic index (SDI).^[6]^ Other studies have highlighted that ALL, while common in childhood, presents unique challenges in young adults.^[7]^ CML and CLL burdens have also been described, often showing different trajectories influenced by therapy advancements like tyrosine kinase inhibitors.^[8,9]^

However, these general analyses often overlook the specific implications for women of childbearing age (WCBA). Although the overall incidence in this group may be lower than in older populations, the impact of leukemia and its aggressive treatments on reproductive health is profound. Diagnosis and therapy can compromise fertility, interact with hormonal profiles, and pose significant risks during pregnancy, potentially affecting both maternal and fetal health. Consequently, the standardized diagnosis, specialized treatment, and comprehensive healthcare management, including fertility preservation and pregnancy counseling, are of critical importance for safeguarding the health of WCBA. Therefore, a focused assessment of the leukemia burden in WCBA is warranted to better understand its unique impact and to encourage healthcare providers to offer tailored, comprehensive healthcare services.

The GBD, Injuries, and Risk Factors Study the GBD serves as a seminal resource for understanding the epidemiological landscape of leukemias among WCBA in 204 countries and 21 territories over the past 3 decades.^[10]^ This study focuses on a comprehensive assessment of the 4 major types of leukemia (AML, ALL, CML and CLL) in WCBA, examining incidence, deaths, disability-adjusted life years (DALYs), and risk factors by age, geographical region, country, and SDI, utilizing data from the latest 2021 GBD study. This study seeks to promote collaboration among clinicians, epidemiologists, and health policymakers to develop effective prevention, screening, and treatment strategies for leukemia in WCBA on a global scale.

## 2. Methods

### 2.1. Data source

This study used leukemia burden data from the GBD 2021 study, covering the evaluation period from 1990 to 2021. Consistent with the World Health Organization (WHO) guidelines for the classification of hematological malignancies, the GBD 2021 framework clearly excludes myelodysplastic syndromes (MDS) and myeloproliferative neoplasms (MPN) from the leukemia classification.^[11]^ Leukemia can be divided into AML, ALL, CML, CLL, and other rare types of leukemia. The International Classification of Diseases, Tenth Revision (ICD-10) codes for included subtypes were as follows: AML (C92.0–92.6), ALL (C91.0), CML (C92.1), CLL (C91.1), and other leukemias (C91.2–91.9, C92.7–94.9), with full code mappings accessible via the GBD Cause Hierarchy. All mortality data referenced in this analysis pertain specifically to leukemia-specific deaths. WCBA were defined as females aged 15 to 49 years according to WHO reproductive health standards.^[12]^

The GBD 2021 study represents an international collaboration of 11,518 researchers across 164 countries, employing standardized protocols to quantify global disease burden through systematic data aggregation. Primary data sources included vital registration systems (63% geographic coverage), population-based cancer registries (28%), and verbal autopsy records (9%), all catalogued in the GBD 2021 Data Input Sources Tool. Leukemia-specific estimates were generated using Cause of Death Ensemble modeling for mortality and DisMod-MR 2.1 for incidence and prevalence, with methodological details described in the GBD 2021 Cancer Methods Appendix. In instances where data were limited or epidemiological patterns were atypical, alternative modeling strategies and approaches were employed. In regions lacking comprehensive cancer registry coverage or reliable mortality data, the GBD 2021 study relied on predictive modeling, data-sharing initiatives, and expert consultations to estimate missing data.

Leukemia-specific data for WCBA, including incidence, deaths, DALYs, and corresponding age-standardized rates (ASRs), were extracted from the Global Health Data Exchange website (https://vizhub.healthdata.org/gbd-results/). The WCBA population with leukemia was stratified into 7 age groups: 15 to 19, 20 to 24, 25 to 29, 30 to 34, 35 to 39, 40 to 44, and 45 to 49 years. The SDI, which ranges from 0 to 1, was used to reflect the development status of a region. A recent GBD 2021 capstone paper described how SDI is assembled and categorized the 204 countries into 5 quintiles (low, low-middle, middle, high-middle, and high) based on their country-level SDI estimates for the year 2021.^[10]^

### 2.2. Statistical analysis

Previous studies have thoroughly explained the procedures and techniques employed in GBD investigations. In this study, we employed annual incidence cases, death cases, DALYs, and their respective ASR per 100,000 persons to illustrate the burden of AML, ALL, CML, and CLL.

ASRs per 100,000 WCBA were calculated using the GBD 2021 reference population structure, following the direct standardization formula^[13]^:


$$\begin{array}{l} ASR=\frac{\overset{7}{\underset{i=1}{\sum }}\left(\alpha_{i}\cdot W_{i}\right)}{\overset{7}{\underset{i=1}{\sum }}W_{i}}\times 100,000 \end{array}$$

Where $\alpha_{i}$ represents the age-specific leukemia rate in the $i^{th}$ 5-year age group, $W_{i}$ denotes the corresponding population weights from the GBD standard population, and the summation spans 7 age strata.

DALYs, quantifying both premature mortality and nonfatal health loss, were computed as:


$$\begin{array}{l} DALY=YLL+YLD \end{array}$$

Where YLL (years of life lost) is derived from age-specific mortality and standard life expectancy, and YLD (years lived with disability) is calculated by multiplying incidence, average disease duration, and disability weight.^[14]^

Uncertainty quantification was performed through 1000 bootstrap iterations using the age adjust direct function within the R package epitools (version 2.0.1), generating 95% uncertainty intervals.^[15]^ Temporal trends were assessed by calculating the percentage change in ASRs between 1990 and 2021:


$$\begin{array}{l} Percentage\;change=\left(\frac{ASR_{2021}-ASR_{1990}}{ASR_{1990}}\right)\times 100 \end{array}$$

A trend was considered statistically significant if the 95% UI of the percentage change excluded zero. Socio-demographic contextualization was achieved through locally weighted scatterplot smoothing (LOESS) models, implemented via the geom_smooth function in ggplot2 (version 3.4.4), to visualize associations between SDI quintiles and leukemia burden metrics across 204 nations. Spearman’s rank correlation coefficients (ρ) were computed to evaluate monotonic relationships between 2021 ASRs and SDI values, as well as between percentage ASR changes and SDI temporal trajectories.

All analyses were conducted in R version 4.3.3, utilizing specialized packages including GBD tools (version 2.1.0) for data extraction and mice (version 3.16.0) for handling incomplete demographic data. Sensitivity analyses included alternative standardization approaches using WHO 2000 population weights and subtype-stratified validation.

### 2.3. Ethical considerations

The GBD study uses de-identified, publicly available data sources. As this study was a secondary analysis of aggregated, anonymous data from the GBD 2021, institutional review board approval and patient informed consent were not required.

## 3. Results

### 3.1. Global, regional, and national burden of overall leukemias

**Figure 1.**
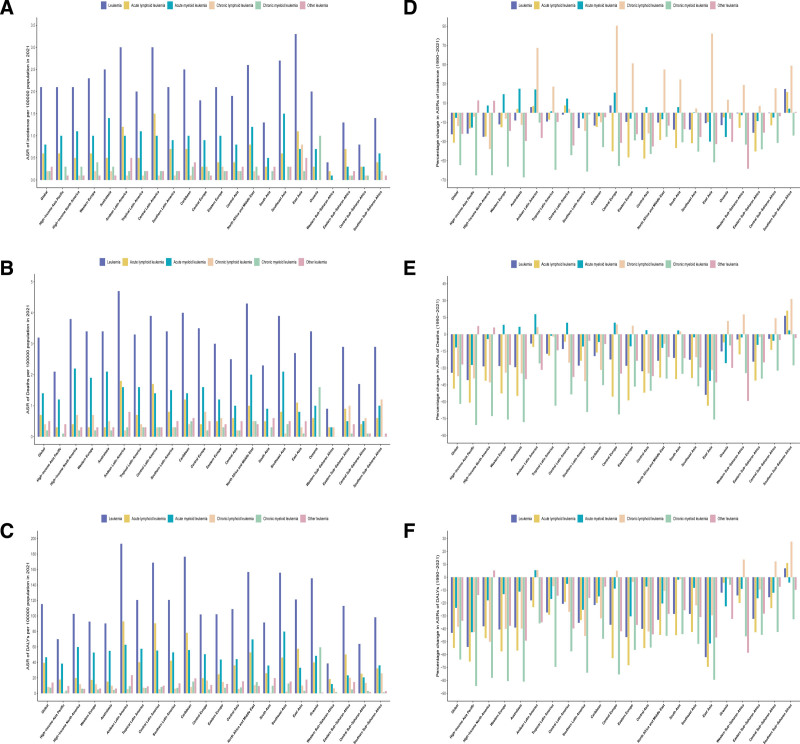
Age-standardized incidence, deaths, and DALYs rates in 2021, and the percentage change in the age-standardized rates from 1990 to 2021 for leukemia among WCBA, globally and by 21 GBD regions. Age-standardized rates of incidence (A), deaths (B), and DALYs (C), and the percentage change in the age-standardized rates from 1990 to 2021 of incidence (D), deaths (E), and DALYs (F). DALYs = disability-adjusted life years, GBD = global burden of disease, WCBA = women of child-bearing age.

**Figure 2.**
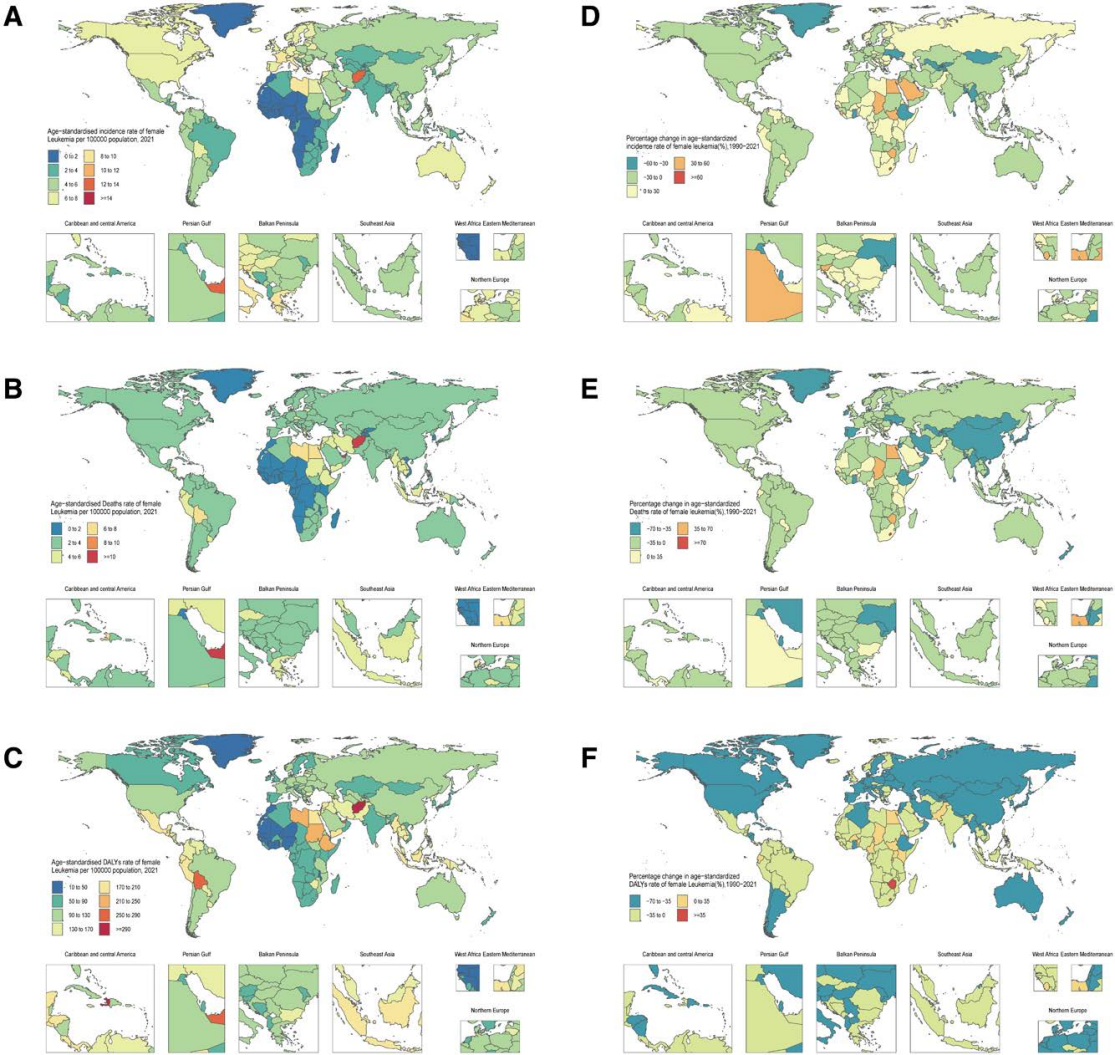
National age-standardized incidence, deaths, and DALY rates in 2021, and the percentage change in the age-standardized rates from 1990 to 2021 for leukemia among WCBA. Age-standardized rates of incidence (A), deaths (B), and DALYs (C). The percentage change in the age-standardized rates from 1990 to 2021 of incidence (D), deaths (E), and DALYs (F). DALYs = disability-adjusted life years, WCBA = women of child-bearing age.

In 2021, the global incidence of leukemia among WCBA was estimated at 40.200 cases (95% UI 31.3, 45.1), corresponding to an age-standardized incidence rate (ASIR) of 2.1 per 100,000 (1.6, 2.3). ASIR declined by 22.4% (95% UI −31.2, −11.4), with larger reductions in age-standardized death rate (ASDR) (-34.2%; −40.5, −25.7) and DALY rate (−43.1%; −50.6, −30.6). Mortality and DALYs showed parallel declines, with 29,000 deaths (22.6, 32.3) and 1,651,000 DALYs (1286.1, 1837.1) in 2021 (Table 1). Geospatial disparities were pronounced: East Asia had the highest ASIR (3.3 per 100,000; 2.0, 4.3), while Andean Latin America exhibited the highest ASDR (4.7; 3.4, 5.8), contrasting sharply with Afghanistan’s 12.0 ASDR per 100,000 (5.9, 18.7): the highest mortality burden among all 204 countries (Table 1, Fig. 1A and B, Table S1, Supplemental Digital Content, https://links.lww.com/MD/R200, Fig. 2A and B). The heaviest absolute DALY burdens fell in Andean Latin America (193,200; 137.9, 237.6) and Afghanistan (463,300; 212.3, 724.1), reflecting healthcare access gradients (Table 1, Table S1, Supplemental Digital Content, https://links.lww.com/MD/R200, Figs. 1C and 2C). Between 1990 and 2021, Southern sub-Saharan Africa exhibited the most pronounced increase in ASIR of leukemia among WCBA, with a rise of 24.6% (95% UI −1.0, 48.3), contrasting sharply with Central Asia’s 28.3% reduction (−36.7, −19.2) (Table 1, Figs. 1D and 2D). Mortality trends showed parallel divergence: while ASDR declined globally, Southern sub-Saharan Africa experienced a 16.4% increase (−9.5, 40.3), alongside a 6.9% rise (−11.6, 27.6) in DALY rate: the only region with upward trajectories. East Asia demonstrated the steepest declines in both ASDR (−53.8%; −66.0, −36.5) and DALY rate (−61.9%; −72.6, −44.1) (Table 1, Fig. 1E and F, Fig. 2E and F).

**Table 1:** Cases and age-standardized rates of incidence, deaths, and DALYs in 2021, and the percentage change in the age-standardized rates from 1990 to 2021 for leukemia in WCBA (15–49 years), globally and by 21 GBD regions.

	Incidence (95% UI)			Deaths (95% UI)			DALYs (95% UI)		
	No, in thousands (95% UI)	ASRs per 100,000 (95% UI)	Percentage changes in ASRs from 1990–2021	No, in thousands (95% UI)	ASRs per 100,000 (95% UI)	Percentage changes in ASRs from 1990–2021	No, in thousands (95% UI)	ASRs per 100,000 (95% UI)	Percentage changes in ASRs from 1990–2021
Global	40.2 (31.3, 45.1)	2.1 (1.6, 2.3)	−22.4 (−31.2, −11.4)	29 (22.6, 32.3)	3.2 (2.6, 3.5)	−34.2 (−40.5, −25.7)	1651 (1286.1, 1837.1)	115.3 (92.4, 127.6)	−43.1 (−50.6, −30.6)
Causes									
ALL	12.1 (7.2, 14.2)	0.6 (0.4, 0.7)	−31.1 (−55.6, −4)	9.4 (5.6, 11.2)	0.7 (0.4, 0.9)	−48.3 (−64.7, −31.7)	562.8 (332.1, 662.7)	39.4 (24.7, 46.4)	−54.8 (−70.3, −37)
AML	15.2 (12, 19.8)	0.8 (0.6, 1)	−5.3 (−20.5, 13)	12.4 (9.9, 16.2)	1.4 (1.1, 1.6)	−11.7 (−26.1, 6.1)	697 (552.6, 924.2)	46.6 (38, 59.2)	−23.7 (−41.3, 0)
CLL	3.9 (2, 5.4)	0.2 (0.1, 0.3)	−13.8 (−27.6, −1.9)	1.0 (0.5, 1.4)	0.4 (0.3, 0.4)	−37.3 (−45.3, −29.3)	53.8 (24.6, 73)	8.2 (5.6, 9.5)	−38.4 (−49.2, −25.8)
CML	3.9 (2.8, 5.2)	0.2 (0.1, 0.3)	−54.4 (−62.3, −43.3)	2.3 (1.6, 3.3)	0.2 (0.2, 0.3)	−61.9 (−69.3, −51.4)	127.6 (87.8, 183.2)	7.3 (5.4, 10)	−63.7 (−72.4, −50.9)
GBD regions									
High-income Asia Pacific	0.8 (0.7, 0.9)	2.1 (1.9, 2.2)	−21.7 (−30.7, −12.4)	0.4 (0.3, 0.4)	2.1 (1.8, 2.3)	−40.7 (−46.7, −36.4)	20.5 (18.5, 21.8)	69.9 (62.7, 75.5)	−54 (−57.4, −48.8)
High-income North America	1.8 (1.7, 1.9)	2.1 (2, 2.2)	−25 (−28.4, −21.9)	0.9 (0.8, 0.9)	3.8 (3.4, 4.1)	−28.5 (−31.8, −25.9)	48.3 (46.2, 50.5)	102.7 (96.4, 107.2)	−38 (−40.2, −35.9)
Western Europe	2.1 (2, 2.2)	2.3 (2.1, 2.4)	−12.1 (−18.2, −6.7)	0.9 (0.8, 0.9)	3.4 (3, 3.7)	−28.2 (−33, −23.3)	47.1 (45.1, 49.4)	92.6 (86.6, 98.2)	−40.5 (−43.8, −37.3)
Australasia	0.2 (0.2, 0.2)	2.5 (2.1, 3)	−8 (−19.8, 6.7)	0.1 (0.1, 0.1)	3.4 (2.9, 3.9)	−29.2 (−37.9, −18.4)	3.8 (3.3, 4.4)	90.2 (79.9, 102.4)	−39 (−45.8, −30.6)
Andean Latin America	0.5 (0.4, 0.7)	3 (2.2, 3.8)	5.8 (−21.2, 36.8)	0.4 (0.3, 0.5)	4.7 (3.4, 5.8)	−8.2 (−30.3, 16.8)	25 (18, 31.5)	193.2 (137.9, 237.6)	−17.9 (−41.5, 6.2)
Tropical Latin America	1.2 (1.2, 1.3)	2 (1.9, 2.1)	−9.1 (−14.4, −2.8)	1 (1, 1.1)	3.3 (3, 3.5)	−17.3 (−22.3, −11.8)	56.7 (53.8, 59.5)	120.5 (112.8, 128.4)	−27.3 (−32, −21.9)
Central Latin America	2.1 (1.8, 2.3)	3 (2.6, 3.4)	−2 (−14.9, 11.1)	1.7 (1.5, 2)	3.9 (3.4, 4.5)	−13.1 (−24, −2.3)	99.8 (86.1, 113.4)	168.8 (147.9, 193.4)	−20.6 (−31.2, −9.6)
Southern Latin America	0.4 (0.3, 0.4)	2.1 (1.9, 2.3)	−15.9 (−26, −5.1)	0.3 (0.2, 0.3)	3.4 (3.1, 3.8)	−27.8 (−36.1, −18.9)	15.5 (14, 17.1)	120.6 (107.8, 135.5)	−35.3 (−43, −26.9)
Caribbean	0.3 (0.2, 0.4)	2.5 (1.9, 3.4)	−13.3 (−26.6, 0.7)	0.3 (0.2, 0.3)	4 (3.2, 5.1)	−19.4 (−32, −6.1)	14.5 (10.6, 20.2)	176.5 (124.9, 242.8)	−21.5 (−36.7, −4.7)
Central Europe	0.5 (0.4, 0.5)	1.8 (1.6, 2)	7.5 (−1.5, 17.2)	0.3 (0.3, 0.3)	3.5 (3.2, 3.8)	−22.4 (−28.4, −16.1)	15.5 (13.9, 17.1)	101.9 (93.1, 111.4)	−36.8 (−42, −30.9)
Eastern Europe	1 (0.9, 1.2)	2.1 (1.8, 2.4)	−9.5 (−17.9, 0.7)	0.7 (0.6, 0.8)	3 (2.7, 3.4)	−28 (−35.2, −19.9)	34.8 (30.4, 40.3)	102.1 (91.7, 113.9)	−46.4 (−51.8, −40.2)
Central Asia	0.5 (0.4, 0.5)	1.9 (1.6, 2.2)	−28.3 (−36.7, −19.2)	0.4 (0.3, 0.4)	2.5 (2.2, 2.8)	−32.8 (−40.6, −24.1)	21.2 (18, 25.1)	108.8 (94.3, 126.8)	−40 (−47.9, −30.9)
North Africa and Middle East	4.2 (2.7, 5.2)	2.6 (1.7, 3.3)	−10.2 (−25.4, 13.5)	3.3 (2.2, 4.2)	4.3 (3, 5)	−23.5 (−36.1, −7)	189.7 (123, 238.8)	156.7 (107.2, 186.9)	−33 (−44.6, −14.9)
South Asia	6.5 (5.3, 8.4)	1.3 (1.1, 1.7)	−17.6 (−31, 4.6)	5.8 (4.8, 7.5)	2.3 (1.8, 2.9)	−21 (−33.3, −2.5)	343 (282.7, 441.5)	91.5 (72.3, 114.9)	−28.4 (−42.4, 2.4)
Southeast Asia	4.9 (3.7, 6.2)	2.7 (2, 3.4)	−17.5 (−31.9, 3.6)	4.3 (3.1, 5.4)	3.9 (3, 4.9)	−22.6 (−35.7, −4.2)	240.2 (177, 302.8)	155.8 (117.3, 192.4)	−28.4 (−43.3, −2.7)
East Asia	10.8 (6.6, 14.4)	3.3 (2, 4.3)	−10.8 (−42.4, 26)	6.1 (3.9, 8)	2.7 (1.8, 3.5)	−53.8 (−66, −36.5)	338.7 (215.3, 447.5)	121.2 (80.5, 152.5)	−61.9 (−72.6, −44.1)
Oceania	0.1 (0, 0.1)	2 (1.1, 2.9)	−12.9 (−28.4, 9)	0.1 (0, 0.1)	3.4 (2, 4.4)	−15.2 (−30.6, 6)	3.8 (2.1, 5.6)	148.6 (85.8, 206.3)	−11.9 (−30.4, 12.5)
Western Sub-Saharan Africa	0.4 (0.2, 0.6)	0.4 (0.2, 0.5)	−0.6 (−24.5, 26.6)	0.4 (0.2, 0.5)	0.9 (0.5, 1.1)	−4.6 (−26, 21)	24 (13.8, 31.6)	38.5 (21.3, 50)	−14.1 (−37, 16.4)
Eastern Sub-Saharan Africa	1.4 (1, 2)	1.3 (1, 1.9)	−20.8 (−43.6, 25.5)	1.3 (0.9, 1.8)	2.9 (2, 3.9)	−24.3 (−45.8, 18.4)	80.4 (57.9, 110.1)	112.9 (77.9, 149.9)	−32.2 (−53.7, 17.1)
Central Sub-Saharan Africa	0.3 (0.2, 0.4)	0.8 (0.5, 1.2)	−0.4 (−30.1, 42.6)	0.2 (0.1, 0.4)	1.7 (1, 2.4)	−4.1 (−32.6, 36.6)	14.8 (8.8, 21.7)	63.8 (39.3, 90.1)	−15.5 (−43.4, 34.3)
Southern Sub−Saharan Africa	0.3 (0.2, 0.4)	1.4 (1, 1.7)	24.6 (−1, 48.3)	0.2 (0.2, 0.3)	2.9 (2, 3.4)	16.4 (−9.5, 40.3)	13.9 (10.1, 17.9)	98.2 (69.5, 118.5)	6.9 (−11.6, 27.6)

ALL = acute lymphoblastic leukemia, AML = acute myeloid leukemia, ASRs = age-standardized rates, CLL = chronic lymphocytic leukemia, CML = chronic myeloid leukemia, GBD = global burden of disease, DALYs = disability-adjusted life years, WCBA = women of child-bearing age.

From 1990 to 2021, the ASIR of leukemia among WCBA showed extreme disparities at the national level. Tokelau exhibited the most dramatic ASIR increase (63.3%; 95% UI 12.2, 156.0), followed by Egypt (57.5%; −22.0, 124.4), whereas the Maldives had the steepest decline (-53.8%; −72.9, 15.2) (Table S1, Supplemental Digital Content, https://links.lww.com/MD/R200, Fig. 2D). Mortality patterns were equally polarized: Lesotho’s ASDR surged by 83.4% (−0.1, 195.5), contrasting with Kuwait’s remarkable reduction (−63.2%; −70.9, −53.8) (Table S1, Supplemental Digital Content, https://links.lww.com/MD/R200, Fig. 2E). The DALY rate extremes were observed in Niue (138.1% increase; 76.7, 231.1) and the Maldives (−67.3%; −81.2, −13.2), with both trends showing UI ranges crossing zero, indicating statistical uncertainty (Table S1, Supplemental Digital Content, https://links.lww.com/MD/R200, Fig. 2F). Due to the rarity of this subtype among other leukemias, subsequent discussions will focus solely on the remaining 4 major types of leukemia.

### 3.2. Global, regional, and national disparities in the burden of 4 leukemias

**Figure 3.**
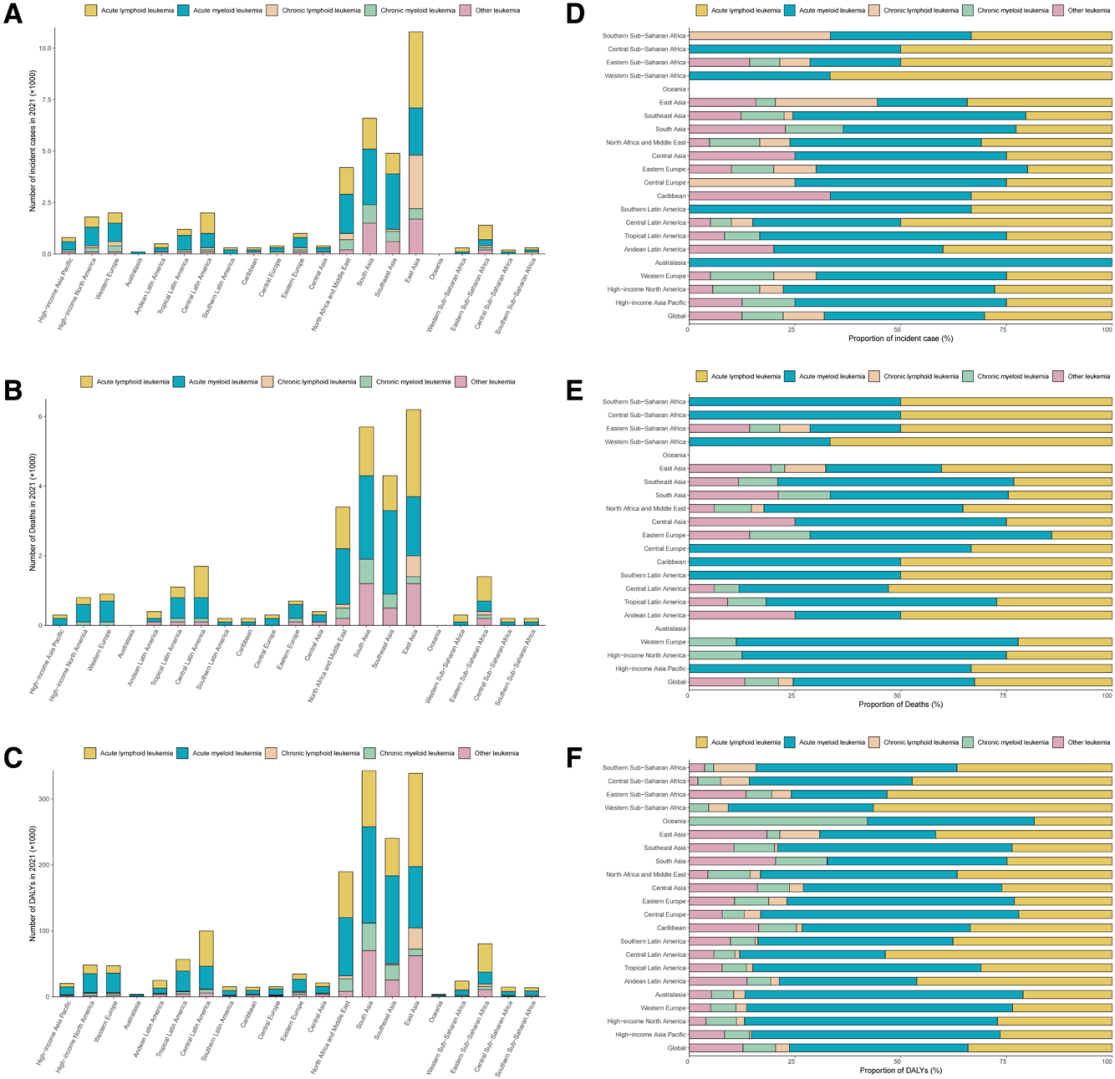
Numbers and proportions of incident cases, deaths, and DALYs contributed by 21 GBD regions, for 5 leukemia subtypes among WCBA, in 2021. Numbers of incident cases (A), deaths (B), and DALYs (C) of each subtype. Proportions of incident cases (D), deaths (E), and DALYs (F) accounted for by each subtype. DALYs = disability-adjusted life years, GBD = global burden of disease.

In 2021, the global burden of leukemia subtypes among WCBA revealed significant heterogeneity. ALL accounted for 12,100 cases (95% UI 7200, 14,200), corresponding to an ASIR of 0.6 per 100,000 (0.4–0.7). AML accounted for 15,200 estimated cases (12,000–19,800; ASIR 0.8, 0.6–1.0), representing 37.8% of total incidence (15.200/40.200). CLL and CML each contributed 3900 cases, though CLL exhibited wider uncertainty intervals (2000–5400 vs CML 2800–5200) despite identical ASIRs (0.2 per 100,000). The mortality pattern is as follows: AML caused 12,400 deaths (9900–16,200; ASDR 1.4, 1.1–1.6), constituting 42.8% of leukemia-related mortality (12,400/29,000), followed by ALL (9400 deaths; 5600–11,200; ASDR 0.7, 0.4–0.9), while CML showed the lowest fatal burden (2300 deaths; 1600–3300) (Table 1, Fig. 1A–C). Notably, these 4 leukemia subtypes present higher incidence rates, death rates, and DALYs among WCBA in East Asia, South Asia, and Southeast Asia (Fig. 3A–C). Subtype-specific DALY proportions were: ALL 34.1% (562,800/1651,000), CML 7.7% (127,600/1651,000), CLL 3.3% (53,800/1651,000), and AML 42.2% (697,000/165,1000) (Fig. 3D–F).

In 2021, the highest ASIRs per 100,000 population were observed as follows: AML in Southeast Asia (1.5, 95% UI 1.1–1.9), ALL in Central Latin America (1.5, 95% UI 1.3–1.7), CML in Oceania (1.0, 95% UI 0.6–1.5), and CLL in East Asia (0.8, 95% UI 0.3–1.2). The highest ASDRs per 100,000 population were reported in High-income North America for AML (2.2, 95% UI 2.0–2.4), Andean Latin America for ALL (1.8, 95% UI 1.1–2.2), Oceania for CML (1.6, 95% UI 0.9–2.4), and Southern Sub-Saharan Africa for CLL (1.2, 95% UI 0.5–1.6). Notably, the highest age-standardized DALY rates per 100,000 population were concentrated in Southeast Asia for AML (79.8, 95% UI 58.3–107.8), Andean Latin America for ALL (92.8, 95% UI 58.6–118.3), Oceania for CML (59.4, 95% UI 33.8–90.3), and Southern Sub-Saharan Africa for CLL (25.8, 95% UI 9.8–34.4) (Tables S2-S5, Supplemental Digital Content, https://links.lww.com/MD/R200; Fig. 1A-C).

From 1990 to 2021, the global ASIRs for leukemia subtypes among WCBA exhibited substantial declines: ALL decreased by 31.1% (95% UI: −55.6% to − 4.0%), AML by 5.3% (95% UI: −20.5% to 13.0%), CLL by 13.8% (95% UI: −27.6% to − 1.9%), and CML by 54.4% (95% UI: −62.3% to − 43.3%). Corresponding reductions were observed in ASDRs and DALY rates, with ALL showing declines of 48.3% (95% UI: −64.7% to − 31.7%) in ASDR and 54.8% (95% UI: −70.3% to − 37.0%) in DALY rates, AML decreasing by 11.7% (95% UI: −26.1% to 6.1%) in ASDR and 23.7% (95% UI: −41.3% to 0.0%) in DALY rates, CLL declining by 37.3% (95% UI: −45.3% to − 29.3%) in ASDR and 38.4% (95% UI: −49.2% to − 25.8%) in DALY rates, and CML demonstrating the most pronounced reductions at 61.9% (95% UI: −69.3% to − 51.4%) in ASDR and 63.7% (95% UI: −72.4% to − 50.9%) in DALY rates (Table 1; Fig. 1D–F).

Among the 21 regions analyzed from 1990 to 2021, heterogeneous trends in ASIRs were observed across leukemia subtypes. For ALL, ASIRs increased in 4 regions, with the most pronounced rise in Southern Sub-Saharan Africa (21.5%, 95% UI: −25.1% to 63.9%). AML exhibited ASIR increases in 11 regions, notably in Australasia (25.0%, 95% UI: 6.9% to 47.2%). CLL showed ASIR elevations in 14 regions, with the largest increase in Central Europe (90.5%, 95% UI: 65.8% to 121.0%). Similarly, ASDRs increased for ALL in 1 region (Southern Sub-Saharan Africa: 20.9%, 95% UI: −26.0% to 62.9%), for AML in 8 regions (Andean Latin America: 17.8%, 95% UI: −13.9 to 63.7%), and for CLL in 8 regions (Southern Sub-Saharan Africa: 31.4%, 95% UI: −14.0 to 83.5%). Age-standardized DALY rates increased for ALL in 1 region (Southern Sub-Saharan Africa: 11.0%, 95% UI: −30.1% to 52.4%), for AML in 1 region (Andean Latin America: 5.4%, 95% UI: −27.4% to 56.5%), and for CLL in 5 regions (Southern Sub-Saharan Africa: 27.6%, 95% UI: −3.0% to 872.5%). In contrast, the age-standardized incidence, mortality, and DALY rates for CML decreased across all regions (Tables S2–S5, Supplemental Digital Content, https://links.lww.com/MD/R200; Fig. 1).

At the national level in 2021, the highest ASIRs for leukemia subtypes were observed in Monaco for ALL (9.2, 95% UI: 3.9–16.1) and AML (6.0, 95% UI: 3.7–8.2), Slovenia for CLL (5.3, 95% UI: 4.0–7.0), and Samoa for CML (3.2, 95% UI: 2.4–6.0). The highest ASDRs were recorded in Afghanistan for ALL (4.1, 95% UI: 1.3–9.3), the United Arab Emirates for AML (5.7, 95% UI: 3–8.6) and CLL (2.6, 95% UI: 1.2–5.1), and Samoa for CML (3.0, 95% UI: 1.8–4.2). Age-standardized DALY rates peaked in Afghanistan for ALL (200.6, 95% UI: 67.0–433.0), Tokelau for AML (169.7, 95% UI: 86.3–275.8), Ethiopia for CLL (38.3, 95% UI: 17.4–63.4), and Samoa for CML (92.3, 95% UI: 58.2–133.3) (Tables S6–S9, Supplemental Digital Content, https://links.lww.com/MD/R200, Figures S1–S4, Supplemental Digital Content, https://links.lww.com/MD/R201). From 1990 to 2021, the most pronounced increases in ASIRs, ASDRs, and DALY rates were observed in Zimbabwe (ALL ASIR: 123.1%, 95% UI: 31.9%–272.7%), Tokelau (ALL ASDR: 117.5%, 95% UI: 5.5%–321.5%), and Niue (ALL DALY: 244.7%, 95% UI: 123.8%–437.7%) for ALL; Georgia (AML ASIR: 163.1%, 95% UI: 106.3%–241.4%; AML ASDR: 164.6%, 95% UI: 106.1%–241.6%) and Tokelau (AML DALY: 153.6%, 95% UI: 40.1%–552.3%) for AML; Saudi Arabia (CLL ASIR: 193.7%, 95% UI: 45.0%–480.3%), Jordan (CLL ASDR: 175.2%, 95% UI: −38.5%–521.7%), and Egypt (CLL DALY: 105.8%, 95% UI: −19.7%–243.0%) for CLL; and Niue (CML ASIR: 30.8%, 95% UI: −17.8%–102.7%) and American Samoa (CML ASDR: 328.7%, 95% UI: −37.1%–2137.0%; CML DALY: 392.5%, 95% UI: −34.1%–2670.3%) for CML. (Tables S6–S9, Supplemental Digital Content, https://links.lww.com/MD/R200; Figures S1–S4, Supplemental Digital Content, https://links.lww.com/MD/R201).

### 3.3. Age-group disparties in the burden of 4 leukemias

**Figure 4.**
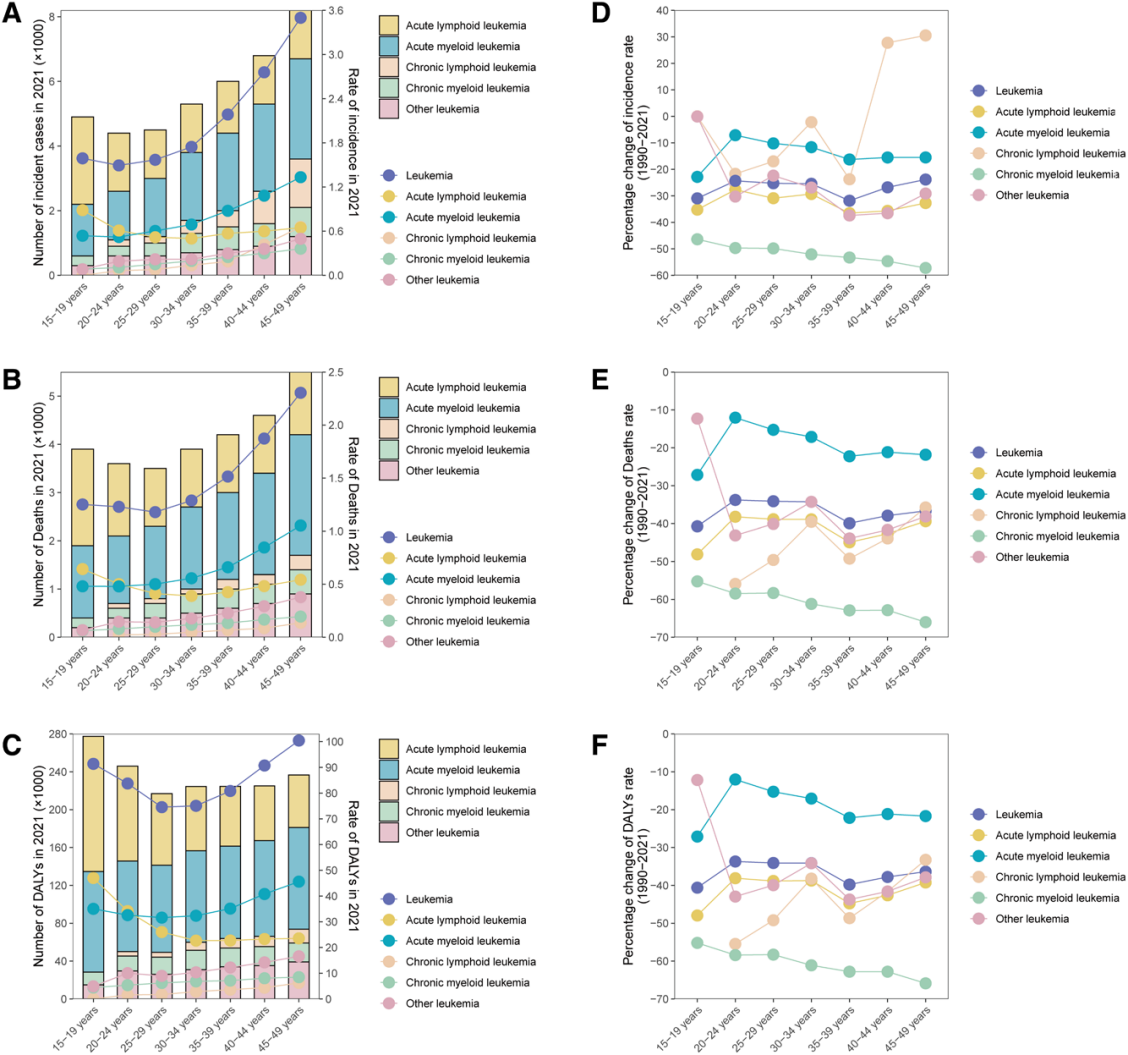
The cross sectional (2021) and longitudinal trends (1990–2021) of incidence rate, deaths rate, and DALY rate of 5 leukemia subtypes throughout WCBA. Numbers and rates of incident cases (A), deaths (B), and DALYs (C) of 5 leukemia subtypes. Percentage changes of incidence rate (D), deaths rate (E), and DALY rate (F) of 5 leukemia subtypes. DALYs = disability-adjusted life years, WCBA = women of child-bearing age.

Among WCBA, the incidence, death rates, and DALYs for the 4 leukemia subtypes and overall female leukemia show a consistent pattern across different age groups. In 2021, the incidence, death, and DALY rates for AML, CLL, and CML increased with age, peaking in the 45 to 49 age group. For ALL, these metrics initially decrease with age before increasing again, peaking at ages 15 to 19. In addition, except for the 15 to 19 years age group, the absolute incidence, death, and DALY numbers and rates for AML are the highest among all other age groups, followed by ALL, CML, and CLL. Notably, in the 15 to 19 years age group, the absolute incidence numbers and DALY numbers, and rates for ALL are the highest, differing from the pattern observed in other age groups (Fig. 4A–C).

From 1990 to 2021, the incidence rate, death rate, and DALY rate for CML exhibited a fluctuating downward trend. Conversely, the percentage changes in the incidence rate, death rate, and DALY rate for ALL, AML, CLL, and the overall burden of leukemia showed a fluctuating upward trend with increasing age in females. It is also noteworthy that, except for CLL, the incidence rate, death rate, and DALY rate for other leukemia subtypes experienced negative growth across all age groups of WCBA from 1990 to 2021. Only the incidence rate of CLL displayed positive growth in the 40 to 44 and 45 to 49 age groups of WCBA. These patterns suggest that CLL is gradually becoming more prevalent among middle-aged women (Fig. 4D–F).

### 3.4. The association between ASR and SDI

**Figure 5.**
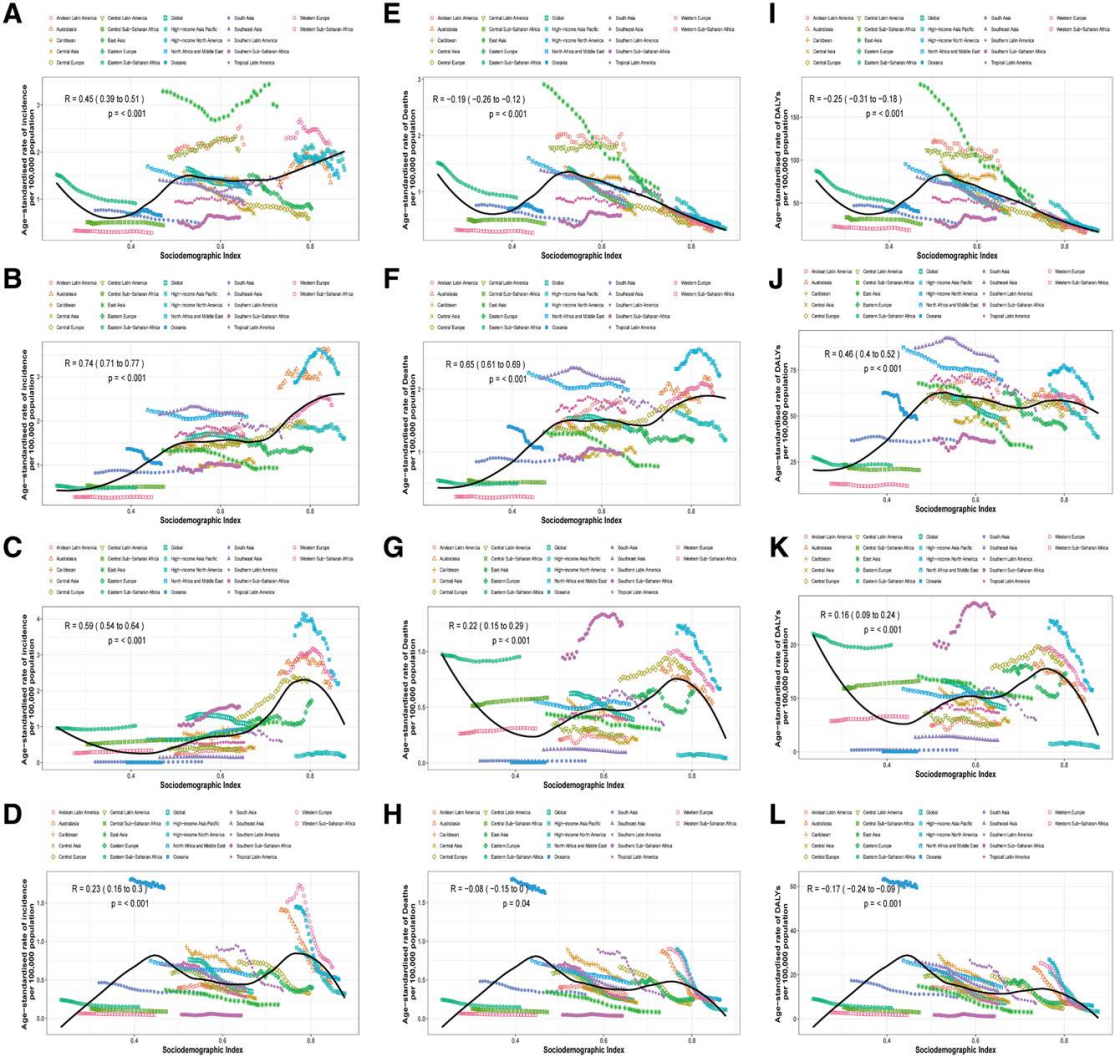
Age-standardized rates of incidence, deaths, and DALYs of 4 leukemia subtypes in WCBA, globally and for 21 GBD regions, by SDI (2021), from 1990 to 2021. Age-standardized incidence rates of ALL (A), AML (B), CLL (C), and CML (D), by SDI. Age-standardized death rates of ALL (E), AML (F), CLL (G), and CML (H), by SDI. Age-standardized DALY rates of ALL (I), AML (J), CLL (K), and CML (L), by SDI. ALL = acute lymphoblastic leukemia, AML = acute myeloid leukemia, CLL = chronic lymphocytic leukemia, CML = chronic myeloid leukemia, DALYs = disability-adjusted life years, GBD = global burden of disease, SDI = socio-demographic index, WCBA = women of child-bearing age.

Between 1990 and 2021, the ASIR of leukemia among WCBA exhibited a strong positive correlation with the SDI (*R* = 0.73, *P* < .001) (Fig. S5A, Supplemental Digital Content, https://links.lww.com/MD/R201). Subtype-specific analyses revealed distinct patterns: ALL showed moderate correlation (*R* = 0.45, *P* < .001), AML demonstrated the strongest linear association (*R* = 0.74, *P* < .001), and CLL displayed a moderate positive trend (*R* = 0.59, *P* < .001) (Fig. 5A–C). In contrast, CML incidence followed a significant nonlinear quadratic relationship, peaking at SDI = 0.45 (Fig. 5D).

For mortality trends, the overall ASDR showed a weak positive correlation with SDI (*R* = 0.33, *P* < .001) (Fig. S5B, Supplemental Digital Content, https://links.lww.com/MD/R201). AML mortality maintained a moderate linear association (*R* = 0.65, *P* < .001), while ALL, CLL, and CML exhibited inverted U-shaped patterns, peaking at SDI = 0.53, 0.75, and 0.45, respectively (Fig. 5E–H). Additionally, the overall age-standardized DALY rate of leukemia in WCBA initially increases and then decreases with the increase of the SDI, but no consistent correlation is evident between the two (Figure S5C, Supplemental Digital Content, https://links.lww.com/MD/R201). Specifically, the age-standardized DALY rate of AML increases with the rise of the SDI, maintaining slight fluctuations after the SDI reaches 0.5, and exhibits a relatively strong positive correlation (*R* = 0.46, *P* < .001) (Fig. 5J). However, for the other 3 subtypes of leukemia, the age-standardized DALY rates generally first increase and then decrease with the rise of the SDI, but the correlations are very weak. ALL peaks at an SDI of 0.53, CLL peaks at an SDI of 0.75, and CML peaks at an SDI of 0.45 (Fig. 5I–L).

### 3.5. Leukemia burden attributable to risk factors

**Figure 6.**
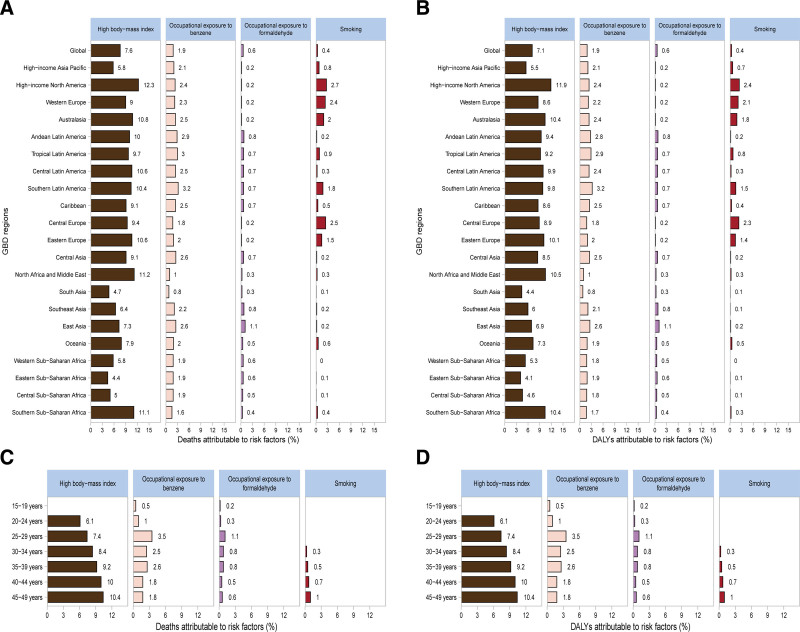
The percentage of deaths (A) and DALYs (B) due to leukemia among WCBA attributable to each risk factor for the 21 global burden of disease regions in 2021. The percentage of deaths (C) and DALYs (D) due to leukemia among WCBA attributable to each risk factor, by age, in 2021. DALYs = disability-adjusted life years, WCBA = women of child-bearing age.

The proportion of death cases and DALYs attributable to individual risk factors for leukemia in WCBA varies across GBD regions. High body mass index (BMI), occupational exposure to benzene or formaldehyde, and smoking are the most common risk factors, with high BMI being the most significant (Fig. 6A–B). In 2021, High-income North America had the highest proportion of death cases and DALYs due to high BMI, accounting for 12.3% and 11.9%, respectively. In contrast, Eastern Sub-Saharan Africa had the lowest percentages, with death cases at 4.4% and DALYs at 4.1% (Fig. 6A–B). Furthermore, the proportion of death cases and DALYs due to leukemia in WCBA attributable to individual risk factors varies by age group. The proportion attributable to high BMI increases with age, peaking at 10.4% in the 45 to 49 years age group. The proportion attributable to occupational exposure to benzene or formaldehyde peaks in the 25 to 29 years age group, at 3.5% for death cases and 1.1% for DALYs (Fig. 6C–D). The patterns for different subtypes of leukemia follow a similar trend across GBD regions and age groups (Figures S6–S9, Supplemental Digital Content, https://links.lww.com/MD/R201).

### 3.6. Sensitivity analysis

The sensitivity analysis, which employed alternative standardization using the WHO 2000 standard population, confirmed that the overall trends in ASRs for incidence, mortality, and DALYs were robust and consistent with the primary findings based on the GBD standard population.

## 4. Discussion

The incidence of the 4 common types of leukemia is consistently higher in males than in females. As a result, epidemiological data on leukemia in females, particularly among WCBA, remain relatively limited.^[6–9]^ This study represents the 1st comprehensive global assessment of the disease burden associated with 4 types of leukemia in WCBA. The main findings are as follows: 1st, in 2021, leukemia accounted for 40,200 prevalent cases, 29,000 deaths, and 1.651 million DALYs among WCBA globally. While the age-standardized point prevalence, mortality, and DALY rates associated with leukemia have declined over the past 31 years, the absolute numbers of cases, deaths, and DALYs have continued to rise. However, these trends varied by region and country. Second, in 2021, the ASIR, ASDR and age-standardized DALY rate of the 4 leukemias were ranked as follows: AML, ALL, CLL, and CML. Third, the age distribution of numbers and rates in incidence, death, and DALYs for overall leukemia exhibited consistent patterns. In 2021, the incidence, death, and DALY numbers and rates of ALL peaked at ages 15 to 19 years. In contrast, these metrics for the other 3 types of leukemia: AML, CLL, and CML, increased with age. The percentage change of disease burden of overall leukemia, AML, ALL and CLL fluctuated, while that of CML showed a declining trend. Fourth, the disease burden of overall leukemia among WCBA exhibited an initial decline followed by a subsequent increase. The impact of age on the disease burden varied across the 4 leukemia subtypes. Among these subtypes, the burden of ALL peaked in the 15 to 19-year age group, after which it initially decreased and then gradually increased with advancing age. For AML, the incidence, mortality, and DALYs rose significantly after the age of 30 to 34 years. In contrast, the disease burden of chronic leukemias demonstrated a consistent upward trend, with both subtypes showing a gradual increase with age. Lastly, high BMI, occupational exposure to benzene or formaldehyde, and smoking were identified as the significant potential risk factors associated with leukemia in WCBA, with high BMI emerging as the most critical factor.

According to the GBD 2021 data, the disease burden of leukemia, both overall and across its 4 subtypes, varies significantly across regions and countries. Several factors may contribute to this phenomenon, including local perceptions of the disease, economic constraints, and limited access to diagnostic tools and treatment options related to leukemia.^[16]^ Research has identified smoking, high BMI, and occupational exposure to benzene or formaldehyde as the most prevalent potential risk factors associated with AML.^[6]^ In our study, we observed that the proportion of leukemia-related deaths and DALY rates attributable to individual risk factors varied among WCBA across different age groups. Notably, the proportion of deaths and DALYs associated with high BMI increased with age, peaking in the 45 to 49 age group. Conversely, the 25 to 29 age group exhibited the highest proportion of deaths and DALYs related to occupational exposure to benzene or formaldehyde. Furthermore, regional differences in leukemia risk factors were evident, suggesting that high BMI may be a more significant risk factor for leukemia in economically developed areas. A similar composition of risk factors was observed across the 4 types of leukemia. Furthermore, the impact of high BMI on DALY and death among patients with these subtypes became more pronounced with increasing age. A meta-analysis examining the associations between 4 types of leukemia and obesity revealed that the over associated risks for CLL, ALL, AML, and CML were 1.25 (95% CI, 1.11–1.41),1.65 (95% CI, 1.16–2.35),1,52 (95%CI, 1.19–1.95), and 1.26 (95% CI, 1.09–1.46), respectively.^[17,18]^ There is insufficient evidence to establish a direct causal relationship between formaldehyde and benzene exposure and the development of myeloid or lymphoid leukemia.^[19]^ Collectively, these findings underscore the importance of implementing tailored dietary regimens and weight management strategies as integral components of treatment for WCBA leukemia.

The age of onset varies significantly across different types of leukemia. ALL is more prevalent in children and young adults, whereas CML and CLL are less common among individuals under 20 years of age.^[7–9]^ AML can occur in infants, adults, and the elderly, but it is relatively rare during adolescence.^[6]^ Consistent with prior literature, our results indicated that among WCBA, the age-standardized incidence, mortality, and DALY rates for AML, CLL, and CML in 2021 were positively correlated with age, peaking in the 45 to 49 age group. In contrast, the disease burden of ALL decreased with age, reaching its apex in the 15 to 19 age group. In addition to influencing the occurrence and progression of leukemia, age significantly affects treatment choice for this disease. For instance, in elderly patients with AML who are unable to tolerate high-dose or standard-dose chemotherapy, treatment options may include a combination of low-dose chemotherapy, hypomethylating agents, venetoclax, and other therapeutic agents.^[20]^ Furthermore, in economically underdeveloped regions, the availability and sustainability of treatment options may also pose significant challenge.^[16]^

Although treatment and survival outcomes for female and male patients with leukemia in the same age group are comparable, women experience unique treatment-related adverse effects-particularly those impacting reproductive health-when subjected to the same chemotherapy regimens as men. For instance, premenopausal women undergoing chemotherapy may encounter abnormal uterine bleeding as a result of thrombocytopenia.^[21]^ Furthermore, long-term chemotherapy may lead to hair loss.^[22]^ Moreover, a small subset of patients is diagnosed with leukemia during pregnancy, necessitating treatment plans that consider both maternal and fetal health.^[23]^ This situation requires collaborative discussions among hematologists, obstetricians, pediatricians, and the patient’s family to formulate appropriate management strategies. There is a notable lack of extensive prospective studies, as well as standardized prevention, detection methods, and treatment guidelines for these conditions.

Our study also has several limitations. First, while the GBD 2021 database encompasses leukemia data from 21 regions and 204 countries, the completeness and accuracy of these data may vary significantly across different regions. Additionally, the definitions and classification of leukemia in the GBD database may not fully align with those established by the WHO, the European LeukemiaNet (ELN), and other guidelines. These discrepancies could introduce potential biases into the data. Second, the global COVID-19 pandemic has significantly impacted the medical resources of various countries, leading to delays in the diagnosis and treatment of leukemia patients. This situation has exacerbated disability and mortality rates among leukemia patients, thereby skewing the data collected during this period. Third, the range of disease-related risk factors included in the data is relatively limited, which restricts our ability to conduct nuanced investigations across different genders, occupations, and age groups. Finally, the GBD dataset primarily focuses on the epidemiological data of overall leukemia and common leukemia subtypes, which limits the ability to conduct in-depth analyses of rare leukemia subtypes or specific patient populations, such as pregnant individuals with leukemia.

In summary, leukemia among WCBA has emerged as a significant global public health challenge, despite a gradual decline in its overall burden over the past 3 decades. However, disparities in medical resources across countries and persistent biases against women in certain regions complicate efforts to deliver timely and personalized leukemia diagnosis and treatment. Addressing this issue requires the collaborative efforts of physicians, obstetricians and gynecologists, nutritionists, epidemiologists, and healthcare policymakers worldwide.

## Acknowledgments

We thank the Institute for Health Metrics and Evaluation (University of Washington), the GBD Collaborators, and all staff who provide that data necessary for this study.

## Author contributions

**Conceptualization:** Zi-Yuan Lu, Xing-Biao Chen, Zhe-Han Yang, Qi-Jian Feng.

**Formal analysis:** Zi-Yuan Lu, Xing-Biao Chen.

**Funding acquisition:** Zi-Yuan Lu.

**Investigation:** Zi-Yuan Lu.

**Methodology:** Zi-Yuan Lu, Wei-Jun Ling.

**Validation:** Zi-Yuan Lu, Xing-Biao Chen, Zhe-Han Yang.

**Visualization:** Zi-Yuan Lu, Xing-Biao Chen, Wei-Jun Ling.

**Writing – original draft:** Zi-Yuan Lu, Xing-Biao Chen.

**Writing – review & editing:** Zi-Yuan Lu, Xing-Biao Chen, Wei-Jun Ling.

**Data curation:** Xing-Biao Chen, Qi-Jian Feng.

**Project administration:** Xing-Biao Chen, Wei-Jun Ling, Qi-Jian Feng.

**Supervision:** Xing-Biao Chen, Zhe-Han Yang.

**Software:** Zhe-Han Yang, Qi-Jian Feng.

**Resources:** Qi-Jian Feng.

## Supplementary Material




