# Supplementary material for: Global burden of leukemia in women of child-bearing age, 1990 to 2021: An update from the Global Burden of Disease Study 2021
Source: Medicine (Baltimore). 2026 Jan 23;105(4):e47217. doi: 10.1097/MD.0000000000047217 (PMC12851764; doi:10.1097/MD.0000000000047217)
Supplement: Supplementary file 1 [file medi-105-e47217-s001.pdf]

Table S1 Age-standardized incidence rates, death rates, and DALY rates in 1990 and 2021, and the percentage change in the age-standardized rates from 1990 to 2021 for leukemia among WCBA (15–49 years), by country.

	Incidence (95% UI)			Deaths (95% UI)			DALYs (95% UI)		
	ASRs per 100000 (95% UI), 1990	ASRs per 100000 (95% UI), 2021	Percentage changes in ASRs from 1990 to 2021	ASRs per 100000 (95% UI), 1990	ASRs per 100000 (95% UI), 2021	Percentage changes in ASRs from 1990 to 2021	ASRs per 100000 (95% UI), 1990	ASRs per 100000 (95% UI), 2021	Percentage changes in ASRs from 1990 to 2021
Global	5.9 (5,6.6)	4.6 (3.8,5.1)	-22.4 (-31.2,-11.4)	4.8 (4,5.4)	3.2 (2.6,3.5)	-34.2 (-40.5,-25.7)	202.5 (156,237.2)	115.3 (92.4,127.6)	-43.1 (-50.6,-30.6)
High-income North America	10 (9.4,10.4)	7.5 (6.9,7.9)	-25 (-28.4,-21.9)	5.4 (5,5.6)	3.8 (3.4,4.1)	-28.5 (-31.8,-25.9)	165.6 (159.6,170.1)	102.7 (96.4,107.2)	-38 (-40.2,-35.9)
Canada	10.4 (9.3,11.6)	7.6 (6.5,8.7)	-26.6 (-39.4,-13)	5.1 (4.6,5.5)	3.3 (2.8,3.8)	-34.3 (-43,-25.2)	151.2 (139.8,163.1)	86.9 (75.9,98.7)	-42.5 (-50.4,-34)
Greenland	2.8 (2,3.4)	1.6 (1.2,2.1)	-43.1 (-56.1,-21.9)	2.9 (2.1,3.5)	1.5 (1.1,2)	-47 (-58.3,-27.1)	97.7 (65.7,117.4)	46.1 (34.5,61.2)	-52.9 (-63.7,-30)
United States of America	10 (9.4,10.4)	7.5 (6.8,7.9)	-24.8 (-28,-22.1)	5.4 (5,5.6)	3.9 (3.5,4.1)	-27.8 (-31.3,-25.2)	167.2 (161,171.6)	104.5 (98,108.8)	-37.5 (-39.7,-35.5)
Australasia	8.5 (7.8,9.1)	7.8 (6.8,8.9)	-8 (-19.8,6.7)	4.8 (4.4,5.1)	3.4 (2.9,3.9)	-29.2 (-37.9,-18.4)	147.9 (139.3,156.2)	90.2 (79.9,102.4)	-39 (-45.8,-30.6)
Australia	8.3 (7.6,9)	7.9 (6.8,9.2)	-4.5 (-18.3,12.8)	4.8 (4.4,5.1)	3.5 (2.9,4.1)	-27.5 (-37.6,-15.1)	146.9 (138.5,155.9)	90.7 (79.3,104)	-38.3 (-46.1,-29)
New Zealand	9.3 (8.4,10.1)	7 (6.2,7.8)	-24.1 (-33,-14.6)	4.9 (4.5,5.3)	3 (2.6,3.3)	-38.3 (-44.4,-31.3)	153 (142.1,162.6)	87.3 (79.6,95.1)	-43 (-48.7,-37.1)
High-income Asia Pacific	5.4 (4.9,5.9)	4.2 (3.7,4.6)	-21.7 (-30.7,-12.4)	3.6 (3.3,3.7)	2.1 (1.8,2.3)	-40.7 (-46.7,-36.4)	152.1 (134.7,160.3)	69.9 (62.7,75.5)	-54 (-57.4,-48.8)
Brunei Darussalam	6.2 (4.5,7.6)	4.5 (3,5.6)	-26.9 (-42.8,-3.5)	6 (4.3,7.4)	4 (2.7,4.9)	-33.2 (-46.2,-12.1)	227.1 (167.2,283.3)	138.9 (98,171.8)	-38.9 (-50.2,-21.9)
Japan	5.7 (5.2,6.3)	4.3 (3.9,4.6)	-24.6 (-34.1,-15.1)	3.3 (3.1,3.4)	2.1 (1.9,2.3)	-35.2 (-40.6,-31.5)	134.4 (130.9,137.3)	69.2 (64.6,72.4)	-48.5 (-51.1,-46.2)
Singapore	4.7 (4.2,5.2)	4.6 (3.9,5.4)	-2.2 (-17.8,15.9)	3.9 (3.5,4.3)	2.2 (1.9,2.5)	-43.8 (-51.4,-34.3)	150.9 (138.5,165)	82.3 (72.1,94.3)	-45.5 (-52.9,-36.6)
Republic of Korea	4.6 (3.6,5.6)	3.9 (2.2,4.9)	-15.6 (-46.3,9.3)	4.1 (3.4,5.2)	1.9 (1.1,2.3)	-53.6 (-70.1,-44)	190.4 (131.8,220.5)	67.8 (43,80.8)	-64.4 (-73.3,-53.2)
Western Europe	8.8 (8.4,9.2)	7.7 (7.1,8.3)	-12.1 (-18.2,-6.7)	4.8 (4.5,5)	3.4 (3,3.7)	-28.2 (-33,-23.3)	155.7 (150.7,160.2)	92.6 (86.6,98.2)	-40.5 (-43.8,-37.3)

Andorra	6.4 (4,10.7)	4.4 (2.8,6.4)	-30.5 (-62.6,20.6)	2.6 (1.7,3.8)	1.6 (1,2.3)	-39.8 (-60.7,-7.3)	90.1 (59.6,130.4)	48.5 (32.5,68.2)	-46.2 (-66.9,-14.1)
Austria	8.1 (7.3,9.1)	7.4 (6.2,8.6)	-8.5 (-24.3,9.2)	4.6 (4.3,5)	3.3 (2.8,3.8)	-28.4 (-39,-17.2)	143.3 (133,155.9)	85.6 (74,97.3)	-40.3 (-49.2,-30.6)
Belgium	9.3 (8.1,10.8)	8 (6.6,9.4)	-14.4 (-30.2,3.3)	5.4 (4.8,6.1)	3.8 (3.1,4.6)	-29.2 (-41.2,-15.5)	165.2 (150,181.6)	105.1 (90.5,121.2)	-36.4 (-46,-25.7)
Cyprus	7.5 (5.8,11.3)	8.2 (5.3,10.6)	8.8 (-36.6,57.3)	6 (4.5,9.6)	3.8 (2.5,4.8)	-37 (-61.8,-11.1)	152.5 (123,225.2)	90.2 (62,112)	-40.8 (-61.5,-23.8)
Denmark	9 (8.3,9.8)	9 (7.8,10.3)	0.4 (-14.7,17.9)	5.7 (5.3,6.1)	4.1 (3.5,4.7)	-28.7 (-38.5,-18.2)	175.1 (163.5,187.3)	104.4 (92.4,117)	-40.4 (-48.1,-31.7)
Finland	6.1 (5.6,6.6)	6.5 (5.7,7.4)	6.5 (-6.6,23.2)	3.8 (3.5,4.1)	2.7 (2.4,3.1)	-28.5 (-36.9,-19.3)	118.7 (110.6,127.5)	80 (70.7,90.2)	-32.6 (-40.9,-23.3)
France	8.4 (7.6,9.3)	8.2 (7,9.4)	-2.4 (-17.3,14.8)	5.1 (4.7,5.5)	3.6 (3.1,4.2)	-28.6 (-37.9,-18)	157.1 (146.8,167.8)	94.3 (83,106.4)	-40 (-47.2,-32.1)
Germany	7.9 (7.1,8.5)	7.8 (6.7,8.8)	-0.9 (-14.4,15.3)	4.8 (4.5,5.2)	3.6 (3.1,4.1)	-25.2 (-34.2,-15)	154.9 (146,163.9)	95.7 (85.5,106.8)	-38.2 (-45.7,-30.2)
Greece	10.7 (9.6,12)	9.4 (8.4,10.4)	-12.3 (-24.7,0.9)	5.5 (5.2,5.9)	4.8 (4.3,5.3)	-13.5 (-22.1,-4.3)	164.9 (156.8,174)	126.7 (115.9,137.8)	-23.2 (-30.1,-15.5)
Iceland	7.8 (6.7,9)	7.7 (6.6,9)	-1.3 (-19.7,21.8)	3.9 (3.3,4.4)	3.1 (2.6,3.6)	-21.4 (-35.7,-4)	122.7 (107.5,138.2)	85.5 (74.4,96.9)	-30.3 (-42,-16.5)
Ireland	7.8 (7,8.7)	6.1 (5.2,7.2)	-21.5 (-34.7,-6.8)	4.8 (4.3,5.1)	2.6 (2.3,3)	-45.1 (-53.6,-36.9)	138.5 (125.8,151.2)	69.5 (60.6,79.7)	-49.9 (-57.8,-42.1)
Israel	9.4 (8.4,10.6)	8 (6.8,9.2)	-15.4 (-29.7,0.2)	6.2 (5.6,6.8)	4 (3.4,4.6)	-35.5 (-44.3,-25.4)	193.1 (176,210)	101.6 (89.6,113.9)	-47.4 (-55,-39.1)
Italy	11.3 (10.2,12.5)	9.1 (8.2,10)	-18.9 (-29.5,-7.9)	5.3 (5,5.5)	3.6 (3.1,4)	-32.2 (-37.7,-27.4)	183.1 (176,189.7)	102.1 (93,109.5)	-44.2 (-47.9,-40.6)
Luxembourg	9.6 (8.7,10.5)	8.3 (7.2,9.6)	-13.6 (-26.1,2.4)	6.1 (5.6,6.5)	4.1 (3.6,4.7)	-32.4 (-41.5,-21.7)	192.4 (177.6,207.5)	101.8 (89.8,115.4)	-47.1 (-54,-38.9)
Malta	6.2 (5.4,7.2)	6.5 (5.4,7.7)	4.8 (-18.2,29.2)	4.2 (3.7,4.8)	3.1 (2.6,3.6)	-26.5 (-39.6,-10.6)	129 (113.9,147.8)	90.1 (77.3,104.1)	-30.2 (-42.8,-16.2)
Monaco	22 (13.3,33.4)	22.8 (14.5,32.9)	3.4 (-34.1,69.1)	7.5 (5.2,10.5)	8.4 (5.9,11)	12 (-22.5,65.3)	273.1 (193.5,372.8)	271.8 (194.7,352.9)	-0.5 (-27.3,42.9)
Netherlands	8.4 (7.5,9.3)	6.6 (5.7,7.7)	-21.7 (-33.1,-7.8)	4.3 (3.9,4.7)	3 (2.6,3.4)	-31.2 (-39.8,-20.4)	135.6 (126.6,144.4)	81.3 (72.6,91.7)	-40.1 (-46.5,-32.2)
Norway	6.4 (5.9,6.9)	7 (6.2,7.6)	9.5 (-1,21.2)	4 (3.7,4.2)	3.4 (3,3.7)	-13.3 (-20.5,-5.3)	125.7 (119.4,132.4)	90.9 (83.8,98.2)	-27.6 (-33.3,-21.1)

Portugal	6.7 (6.1,7.5)	6.7 (5.6,7.9)	-0.3 (-16.3,21)	4.8 (4.4,5.1)	3.1 (2.6,3.6)	-35.2 (-44.8,-25.1)	175.6 (162.2,189)	89.6 (79.4,100.6)	-49 (-55.2,-41.2)
San Marino	17.5 (11.8,25.2)	10.3 (6.3,15.2)	-41.4 (-65,0)	5.6 (4.2,7.5)	2.9 (1.7,4.2)	-48.2 (-68,-20.5)	179.2 (137.8,236.7)	94.3 (61,138.1)	-47.4 (-65.5,-20.2)
Spain	8.7 (7.9,10)	7.8 (6.7,9)	-10.7 (-26.2,7)	4.6 (4.3,4.9)	2.9 (2.5,3.3)	-37.2 (-45.1,-29.2)	162.5 (152.4,172.7)	85 (75.2,95.7)	-47.7 (-54.3,-40.3)
Sweden	9 (7.9,10.3)	6.9 (5.9,8.2)	-23.1 (-36.2,-4.7)	4.1 (3.7,4.5)	3 (2.5,3.5)	-26.8 (-38.1,-14.6)	132.5 (121.1,144.3)	79.1 (67.5,92)	-40.4 (-49.4,-29.7)
Switzerland	9.5 (8.4,10.7)	8.3 (7,9.8)	-11.9 (-27.4,4.2)	4.1 (3.7,4.4)	2.9 (2.4,3.4)	-29 (-39.1,-17.8)	132.1 (121.2,142)	81.1 (70.5,94.3)	-38.6 (-46.8,-28.8)
United Kingdom	8.8 (8.4,9.1)	6.7 (6.2,7)	-23.9 (-27.6,-20.1)	4.1 (3.9,4.2)	3.3 (3,3.4)	-20.5 (-24.5,-17.5)	131.2 (127.5,134.1)	86.2 (81.7,89.8)	-34.3 (-36.7,-32.1)
Southern Latin America	5.1 (4.8,5.4)	4.3 (3.8,4.9)	-15.9 (-26,-5.1)	4.8 (4.5,5.1)	3.4 (3.1,3.8)	-27.8 (-36.1,-18.9)	186.5 (176.2,196.8)	120.6 (107.8,135.5)	-35.3 (-43,-26.9)
Argentina	5.2 (4.9,5.6)	4.2 (3.7,4.8)	-20.3 (-30.3,-8.8)	4.9 (4.6,5.2)	3.5 (3.2,4)	-28 (-36.1,-18.9)	195 (183.4,205.9)	125.8 (111.3,142.7)	-35.5 (-43.7,-26.1)
Chile	4.6 (4.2,4.9)	4.3 (3.7,4.9)	-6.2 (-19,7.5)	4.2 (3.9,4.6)	3 (2.7,3.4)	-29.3 (-38.2,-19.8)	162.6 (152.2,172.6)	102 (90.8,114.9)	-37.3 (-45,-28.6)
Uruguay	6 (5.4,6.5)	6 (5.1,6.9)	0.2 (-15.1,18)	5.2 (4.7,5.7)	4.5 (3.8,5.1)	-13.7 (-26.4,0.7)	194 (178.3,212.7)	145.3 (124.6,166.2)	-25.1 (-36.8,-12)
Eastern Europe	5.3 (5.1,5.5)	4.8 (4.3,5.3)	-9.5 (-17.9,0.7)	4.2 (4.1,4.4)	3 (2.7,3.4)	-28 (-35.2,-19.9)	190.5 (184.7,196.8)	102.1 (91.7,113.9)	-46.4 (-51.8,-40.2)
Belarus	6.1 (5.5,6.7)	6.3 (5,7.8)	2.3 (-20.3,30.6)	4.8 (4.5,5.2)	3.5 (2.8,4.3)	-27.4 (-42,-9.5)	199.7 (182.4,218.5)	116.7 (94.6,142.5)	-41.6 (-53,-27.7)
Estonia	8.1 (7.3,8.9)	7 (5.8,8.4)	-13.4 (-29.9,5.5)	5.8 (5.3,6.3)	3.5 (3,4.2)	-38.4 (-49,-26.7)	218 (198.9,239.9)	112.9 (95.8,130.7)	-48.2 (-57.2,-38.6)
Latvia	6.9 (6.3,7.7)	6.4 (5.3,7.7)	-8 (-24.9,14.3)	5.3 (4.8,5.8)	3.6 (3,4.3)	-31.8 (-43.6,-16.7)	201.9 (184.8,221.1)	116 (98,136.6)	-42.5 (-52.1,-30.5)
Lithuania	7.7 (6.9,8.4)	6.4 (5.4,7.5)	-16.5 (-31.1,2.1)	5.5 (5,5.9)	4.2 (3.5,4.9)	-23.7 (-36.5,-9.5)	206.6 (186.8,226.3)	130.1 (109.2,151.8)	-37 (-48.2,-24.2)
Republic of Moldova	4.6 (4.3,5)	2.8 (2.5,3.3)	-38.7 (-47.5,-27.8)	4.2 (3.9,4.5)	2.1 (1.9,2.4)	-48.8 (-56.4,-40.9)	191.5 (177.1,208.8)	79.7 (69.4,92)	-58.4 (-64.9,-50.9)
Russian Federation	4.7 (4.6,4.8)	4.8 (4.3,5.3)	2.5 (-8.2,12.3)	3.8 (3.7,3.9)	3 (2.7,3.3)	-20.4 (-28.7,-12.9)	169.8 (166.1,174.5)	97.9 (88.6,106.9)	-42.3 (-48.3,-37.1)
Ukraine	6.5 (6,7.1)	4.4 (3,6.2)	-33 (-55.2,-3)	5.1 (4.7,5.5)	3 (2,4.2)	-41.9 (-62.3,-17)	246.6 (228.1,267.4)	114.5 (79.3,159.2)	-53.6 (-69.3,-33.4)

Central Europe	5.2 (4.9,5.5)	5.6 (5,6.1)	7.5 (-1.5,17.2)	4.6 (4.3,4.8)	3.5 (3.2,3.8)	-22.4 (-28.4,-16.1)	161.2 (153.1,169.8)	101.9 (93.1,111.4)	-36.8 (-42,-30.9)
Albania	3.2 (2.5,4.4)	3.1 (2.1,4.4)	-4.3 (-36.1,45.7)	3.2 (2.5,4.5)	2.4 (1.6,3.3)	-25.6 (-48.5,9.3)	135.5 (105.1,187.2)	85.2 (57.2,120.1)	-37.2 (-57.7,-4.8)
Bosnia and Herzegovina	3 (2.5,4)	3.7 (2.3,4.9)	22 (-19.8,70)	2.9 (2.4,3.7)	2.7 (1.8,3.5)	-3.9 (-33.9,30.6)	97.5 (80.1,123)	81.2 (51.8,104.1)	-16.7 (-40.5,10.6)
Bulgaria	4.2 (3.7,4.7)	4.8 (3.7,6)	15.9 (-11.5,54.5)	3.8 (3.3,4.3)	4 (3.1,4.9)	5 (-18.3,38.1)	150.8 (131.7,171.6)	133 (103.6,167.5)	-11.8 (-32.1,17.8)
Croatia	5.9 (4.9,7.1)	7.5 (6.2,9.2)	26.3 (-2.8,66.9)	4.5 (3.7,5.5)	3.8 (3,4.8)	-16.1 (-37.3,14.3)	134 (111.2,159.2)	105.9 (85.1,132.3)	-20.9 (-41.1,8.2)
Czechia	6.1 (5.6,6.5)	7.1 (5.8,8.6)	15.9 (-6.3,43.7)	5.3 (4.9,5.7)	4.1 (3.5,5)	-21.7 (-34.6,-3.7)	168.3 (156.6,180.8)	109.8 (91.6,132)	-34.8 (-46.1,-21)
Hungary	6.5 (6,7)	6.4 (5.4,7.5)	-1.4 (-18.8,17.2)	5.4 (5.1,5.9)	3.8 (3.2,4.5)	-30 (-41.7,-17.1)	184.8 (169.5,199.8)	113.4 (94.4,136.3)	-38.7 (-50.1,-25.6)
Montenegro	4.7 (3.4,6.1)	5.1 (3.6,6.9)	9 (-25.7,52.8)	3.5 (2.7,4.5)	3.3 (2.5,4.2)	-6.5 (-36.5,27.8)	133.6 (102.3,171.3)	97.7 (74,121.2)	-26.8 (-46.2,-3.8)
North Macedonia	4.3 (3.5,5.6)	4.4 (2.9,5.8)	3.4 (-23.8,38.6)	4 (3.3,5.2)	3.4 (2.5,4.3)	-14.9 (-35.2,11.8)	154 (124.8,191.6)	95.4 (66.5,123.6)	-38 (-53.4,-14.9)
Poland	5.9 (5.5,6.3)	5.8 (5.1,6.5)	-1.3 (-13.7,12.4)	5.2 (4.8,5.7)	3.6 (3.2,4)	-30.9 (-39,-21.8)	173.3 (166,181.4)	96.6 (86.2,106.6)	-44.3 (-50.3,-37.2)
Romania	3.7 (3.5,4)	4.5 (3.7,5.2)	19.7 (-3.1,42.2)	3.4 (3.2,3.6)	3.2 (2.7,3.7)	-6 (-21.5,10.4)	154 (141.4,166.3)	103.2 (86.3,121.6)	-32.9 (-45.7,-20.1)
Serbia	4.8 (3.5,6.2)	4.9 (3.4,6.5)	3.2 (-25.6,40.4)	4.3 (3.2,5.7)	3.2 (2.2,4.2)	-25 (-43.3,1.7)	151.7 (110.7,200.9)	91.3 (64.6,117.4)	-39.8 (-56.4,-16.7)
Slovakia	5.4 (4.3,6.8)	5.5 (3.8,7.5)	2.2 (-27.7,46.9)	4.6 (3.8,5.9)	3.5 (2.4,4.6)	-24.1 (-44.8,7)	157.3 (128.1,202.7)	105.7 (74.1,141.8)	-32.8 (-50.8,-6.3)
Slovenia	6.1 (5.4,6.9)	8.8 (7.1,11)	44.3 (12.3,84.8)	4.2 (3.8,4.6)	3.2 (2.6,3.9)	-24 (-38.8,-6.6)	136.5 (123.2,151)	87.1 (71,106.8)	-36.2 (-49,-20.5)
Central Asia	4.1 (3.9,4.3)	2.9 (2.6,3.3)	-28.3 (-36.7,-19.2)	3.7 (3.5,3.9)	2.5 (2.2,2.8)	-32.8 (-40.6,-24.1)	181.4 (171.6,193.3)	108.8 (94.3,126.8)	-40 (-47.9,-30.9)
Armenia	5.4 (5.1,5.7)	3.5 (3.1,4)	-34.4 (-43.3,-23.7)	4.5 (4.3,4.8)	2.6 (2.3,3)	-43.2 (-51,-33.4)	212.4 (199.8,225.5)	92.2 (81.5,104.3)	-56.6 (-62.2,-50)
Azerbaijan	4.1 (3.3,5.2)	3 (2.2,4.3)	-25.8 (-45.4,-0.5)	3.8 (3,4.8)	2.6 (1.8,3.6)	-32.6 (-49.4,-11.7)	201.1 (159.2,249.3)	124.2 (88.3,176.4)	-38.3 (-53.1,-18.1)
Georgia	5.3 (4.8,5.7)	4.4 (3.9,4.9)	-16 (-26.2,-2.9)	4.1 (3.8,4.5)	3.6 (3.2,4.1)	-12.4 (-22.8,-1.5)	208.3 (192.3,224.2)	130.5 (114.6,146.3)	-37.3 (-44.7,-29.5)

Kazakhstan	3.8 (3.6,4.1)	2.9 (2.5,3.3)	-24.7 (-34.5,-13.1)	3.5 (3.3,3.7)	2.3 (2,2.6)	-33.9 (-42.6,-24.5)	164 (154.6,173.8)	88.4 (78.5,99.3)	-46.1 (-52.5,-39.3)
Kyrgyzstan	3.7 (3.3,4)	2.4 (2,2.8)	-35.5 (-46.9,-21.9)	3.3 (2.9,3.6)	2 (1.6,2.3)	-39.8 (-50.5,-27.1)	165.6 (148.2,182.3)	87 (72.7,103.3)	-47.5 (-56.8,-35.8)
Mongolia	3.3 (2.4,4.8)	2.2 (1.5,2.9)	-34.8 (-55.6,-5.6)	3.3 (2.4,4.6)	2 (1.4,2.7)	-38 (-57.4,-9.3)	165.4 (117.6,240.2)	89.6 (61.5,124.5)	-45.8 (-64.1,-19.6)
Tajikistan	3.4 (2.4,4.5)	2.2 (1.4,3.4)	-36.8 (-60.9,3.3)	3.2 (2.2,4.2)	2 (1.2,3.1)	-38.8 (-61.5,-2.1)	169.9 (114.9,219)	99 (60.1,166.9)	-41.7 (-64.4,0.8)
Turkmenistan	3.4 (3.1,3.7)	2.7 (2.2,3.4)	-20 (-36.8,4)	3.1 (2.9,3.4)	2.3 (1.8,3)	-25.9 (-41,-3.5)	163.4 (150.4,178.1)	112.4 (90.4,139.9)	-31.2 (-44.7,-12.3)
Uzbekistan	4.1 (3.7,4.5)	2.8 (2.3,3.4)	-32.8 (-46.1,-15.1)	3.8 (3.4,4.1)	2.3 (1.9,2.9)	-37.4 (-49.1,-21.3)	187.4 (167.9,208.3)	116.1 (95.2,143.9)	-38 (-50.1,-21.4)
Central Latin America	4.7 (4.5,4.8)	4.6 (4,5.2)	-2 (-14.9,11.1)	4.5 (4.4,4.7)	3.9 (3.4,4.5)	-13.1 (-24,-2.3)	212.5 (205.2,220.7)	168.8 (147.9,193.4)	-20.6 (-31.2,-9.6)
Colombia	5.1 (4.7,5.4)	4.7 (3.9,5.7)	-6.9 (-23.8,14.6)	4.9 (4.5,5.2)	3.8 (3.1,4.4)	-22.9 (-35.7,-8.6)	213.5 (199.1,230.8)	153.6 (128.3,184.1)	-28.1 (-40.3,-13.8)
Costa Rica	5.6 (5.1,6)	6.2 (5.3,7.1)	11.9 (-4.4,31)	4.9 (4.5,5.3)	4.6 (4,5.2)	-6 (-19.7,8.1)	190.4 (177.5,203.6)	178.9 (157.5,200.3)	-6 (-18.2,7.5)
El Salvador	4.8 (4.1,6.1)	5 (3.5,6.5)	4.6 (-22.7,35.4)	4.7 (4,6.1)	4.3 (3,5.5)	-7.6 (-33,21)	231.9 (191.5,293.2)	184.6 (130.4,236.2)	-20.4 (-40.5,5.5)
Guatemala	3.5 (3.3,3.8)	4 (3.4,4.6)	12.4 (-4.9,31.7)	3.6 (3.4,3.8)	3.8 (3.2,4.3)	5.1 (-11.6,22.2)	173.7 (161.2,186.5)	172 (146.7,200.8)	-0.9 (-16.8,16.6)
Honduras	5.6 (4.2,6.9)	4.9 (3.2,6.9)	-12.4 (-39.3,26)	5.5 (4.2,6.8)	4.8 (3.2,6.7)	-13.3 (-40.3,23.1)	281.8 (207.2,351.4)	181.6 (117.2,274.1)	-35.6 (-55.5,-4)
Mexico	4.7 (4.5,4.9)	4.6 (4,5.2)	-2.1 (-16,11.7)	4.6 (4.4,4.7)	4 (3.4,4.5)	-12.7 (-24.9,-1)	217 (208.4,227.9)	175.1 (151.9,196.6)	-19.3 (-30.1,-8.5)
Nicaragua	3.9 (3.3,5.1)	3.3 (2.4,4.2)	-15.2 (-36,10.4)	3.7 (3,5)	2.8 (2,3.6)	-23.2 (-42.7,0)	198.2 (159.7,264.3)	120.7 (85.6,151)	-39.1 (-56,-16.8)
Panama	4.1 (3.8,4.5)	5.1 (4.1,6.1)	23.7 (1.3,49.8)	3.8 (3.5,4.1)	3.9 (3.2,4.7)	3.8 (-15.5,24.5)	182.3 (169.2,198.2)	171.5 (141.8,206.1)	-5.9 (-22.8,12.4)
Venezuela (Bolivarian Republic of)	4.3 (4.1,4.6)	4.5 (3.3,6)	4.8 (-24.1,40.4)	4.1 (3.9,4.3)	3.9 (2.8,5)	-6.1 (-30.7,24.1)	191.3 (180.7,201.6)	168.3 (121.3,224.1)	-12 (-36.6,17.8)
Andean Latin America	5.1 (4.3,6.4)	5.4 (3.9,6.7)	5.8 (-21.2,36.8)	5.1 (4.3,6.3)	4.7 (3.4,5.8)	-8.2 (-30.3,16.8)	235.3 (191.9,303.4)	193.2 (137.9,237.6)	-17.9 (-41.5,6.2)

Bolivia (Plurinational State of)	7.9 (4.9,11.1)	6.6 (4.3,9.2)	-16.5 (-44.1,25.1)	8 (5.1,11.3)	6.4 (4.2,9.1)	-19.8 (-45.8,17.9)	372.1 (220.3,524)	258.1 (171,362.8)	-30.6 (-56.6,16.9)
Ecuador	4.4 (4.1,4.8)	5.3 (4.3,6.4)	20.9 (-4.1,50)	4.4 (4.1,4.8)	4.8 (3.9,5.9)	8.5 (-13.8,33.6)	187.4 (174.1,202.9)	194.2 (159,235.8)	3.7 (-16.7,26.7)
Peru	4.6 (3.8,6.1)	5.2 (3,6.8)	11.1 (-32.1,55.4)	4.6 (3.7,5.9)	4.1 (2.3,5.5)	-9.3 (-43.8,27.1)	215.6 (174.3,294.1)	172.1 (96.8,229.8)	-20.2 (-53.7,12.3)
Caribbean	5.5 (4.6,6.5)	4.8 (3.9,5.9)	-13.3 (-26.6,0.7)	5 (4.2,6)	4 (3.2,5.1)	-19.4 (-32,-6.1)	224.8 (166.4,291.3)	176.5 (124.9,242.8)	-21.5 (-36.7,-4.7)
Antigua and Barbuda	4.2 (3.8,4.7)	4.7 (4.3,5.1)	10.6 (-3.2,27.3)	3.9 (3.5,4.3)	3.8 (3.5,4.1)	-1.5 (-12.5,12.2)	149.6 (135.7,165.2)	130.5 (119.4,142.4)	-12.8 (-22.9,-0.2)
Barbados	4.9 (4.5,5.3)	5.3 (4.2,6.6)	8.8 (-15.6,34.9)	4.6 (4.3,5)	4.7 (3.7,5.7)	0.6 (-22.2,23)	187.9 (172,204.6)	165 (127.9,205)	-12.2 (-32.4,8.1)
Belize	3.2 (2.9,3.5)	3 (2.6,3.4)	-4.6 (-18,10.6)	2.9 (2.6,3.2)	2.7 (2.3,3)	-7.6 (-20.8,7.4)	134.9 (122.5,149.1)	98.6 (86.3,112.4)	-26.9 (-37.9,-15.3)
Bermuda	4.6 (3.9,5.3)	4 (3,5.6)	-12.1 (-37.3,25.1)	4.3 (3.8,5)	2.2 (1.8,2.9)	-48.1 (-61.1,-31.7)	150.7 (130.1,175.1)	79 (61.3,102.4)	-47.6 (-61,-30.2)
Bahamas	3.8 (3.4,4.2)	3.7 (2.9,4.5)	-3 (-25.4,25.8)	3.3 (2.9,3.6)	2.9 (2.3,3.6)	-11.3 (-30.4,15.2)	133.8 (121.4,147.4)	108.5 (84.8,135.5)	-19 (-38.2,6.5)
Cuba	5.5 (5.1,5.9)	4.6 (3.8,5.5)	-16.9 (-32.5,1.4)	4.6 (4.3,4.9)	3.2 (2.6,3.8)	-31 (-42.8,-17)	175.8 (163.7,188.5)	112.1 (92.9,134)	-36.2 (-48,-22.9)
Dominica	4.9 (3.9,6.2)	5.6 (4,7.4)	13.9 (-14,52)	4.9 (3.9,6.1)	5.3 (3.8,6.9)	7.3 (-19.5,43.4)	180.3 (146.2,223.5)	212 (151.8,282.7)	17.5 (-13.6,55.3)
Dominican Republic	3.4 (2.8,4.3)	2.8 (2,3.8)	-19.6 (-39.1,5.1)	3.3 (2.7,4.2)	2.5 (1.9,3.5)	-23.3 (-41.1,-1)	163.8 (128.9,206.1)	104.2 (76,148.7)	-36.4 (-52.4,-14.6)
Grenada	5.9 (5.3,6.7)	6 (5.2,6.8)	1.1 (-14.9,20.2)	5.4 (4.8,6)	4.8 (4.2,5.5)	-10.8 (-24.4,5.1)	234.9 (207.2,265.5)	176.9 (153.4,202.4)	-24.7 (-36.7,-10.6)
Guyana	3.1 (2.7,3.5)	3.1 (2.3,4)	0.9 (-28.2,35.4)	3 (2.6,3.5)	2.9 (2.1,3.7)	-4.7 (-31.7,27.5)	129.1 (108.9,148.3)	118.2 (87.4,156.7)	-8.4 (-34.9,21.5)
Haiti	8.1 (3.8,12.8)	6.3 (3.4,10.4)	-21.6 (-47,17.6)	8.2 (3.9,13)	6.3 (3.4,10.4)	-22.4 (-47.1,16)	407.9 (162.1,675.5)	295.1 (140.2,497.6)	-27.6 (-52,16.1)
Jamaica	3.5 (3.1,4)	4 (3,5.2)	13.5 (-13.4,50.8)	3.2 (2.9,3.6)	3.5 (2.7,4.5)	8.6 (-19,42.5)	138.7 (124.3,156.8)	130.7 (99.8,169.9)	-5.8 (-30.4,24.9)
Puerto Rico	5.4 (4.9,6)	5 (4,6)	-7.7 (-26.9,13.4)	4.6 (4.1,5)	3.3 (2.7,4)	-27.2 (-41.7,-9.7)	167.4 (152.7,184.4)	107 (86.1,129.4)	-36.1 (-48.1,-20.9)

Saint Kitts and Nevis	5.3 (4.8,5.9)	3.4 (2.8,4.1)	-36.8 (-49,-21.1)	5.3 (4.8,5.8)	3.1 (2.6,3.6)	-42.1 (-52.7,-29.9)	218 (198.1,240.2)	109.4 (91.1,130.5)	-49.8 (-59.1,-37.3)
Saint Lucia	4.8 (4.4,5.3)	3.5 (2.8,4.3)	-27.4 (-41.4,-9.3)	4.7 (4.3,5.1)	3 (2.4,3.6)	-36.3 (-48.5,-21.5)	177.4 (162,194.5)	115.2 (94,141.1)	-35.1 (-48.3,-18.1)
Saint Vincent and the Grenadines	5.7 (5.1,6.2)	4.6 (3.9,5.4)	-20.1 (-32.9,-4.4)	5.3 (4.8,5.8)	4 (3.5,4.7)	-23.8 (-36.3,-9)	215.8 (193.5,237.6)	171.8 (147.2,200.2)	-20.4 (-33.7,-4.9)
Suriname	3.6 (2.5,4.3)	3.3 (2.4,4.2)	-7.6 (-28.9,23.1)	3.5 (2.4,4.2)	3 (2.1,3.8)	-13.2 (-33.3,14.6)	150.9 (92.7,181.4)	131.3 (92,165.9)	-13 (-34.2,16.3)
Trinidad and Tobago	4.6 (4.2,5)	4 (3,5.2)	-13.3 (-34.7,11.9)	4.5 (4.1,4.8)	3.5 (2.7,4.5)	-22 (-40.9,1.2)	182.1 (167.5,197.4)	137 (104.8,176.6)	-24.8 (-43,-3.3)
United States Virgin Islands	4.1 (3.2,5.2)	2.2 (1.5,3)	-47.2 (-62.5,-25.1)	3.7 (2.9,4.7)	1.7 (1.2,2.3)	-54.3 (-66.8,-35.3)	139 (106.6,174.3)	61.9 (43.3,85.4)	-55.5 (-68.1,-38)
Tropical Latin America	4.1 (3.9,4.3)	3.7 (3.4,4)	-9.1 (-14.4,-2.8)	4 (3.8,4.2)	3.3 (3,3.5)	-17.3 (-22.3,-11.8)	165.9 (157.9,174.1)	120.5 (112.8,128.4)	-27.3 (-32,-21.9)
Brazil	4.1 (3.9,4.3)	3.7 (3.4,3.9)	-9.7 (-14.8,-3.5)	4 (3.8,4.2)	3.3 (3,3.5)	-17.8 (-22.8,-12.1)	165.8 (157.6,174.2)	119.6 (112.5,127)	-27.8 (-32.3,-22.6)
Paraguay	4 (3.2,5.1)	4.5 (3,6.2)	11.2 (-21.3,56.1)	3.9 (3.1,4.9)	3.9 (2.6,5.3)	0.8 (-29.9,40.5)	169.6 (129.3,210.4)	147.8 (102.4,200.4)	-12.8 (-37.2,21.9)
East Asia	6.6 (4.5,8.1)	5.9 (3.5,7.6)	-10.8 (-42.4,26)	5.9 (4.1,7.3)	2.7 (1.8,3.5)	-53.8 (-66,-36.5)	317.9 (211.1,398.4)	121.2 (80.5,152.5)	-61.9 (-72.6,-44.1)
China	6.7 (4.6,8.2)	5.9 (3.4,7.7)	-11.4 (-43.2,26.5)	6 (4.2,7.4)	2.7 (1.8,3.5)	-54.8 (-67.1,-37.2)	323.5 (213.3,406.2)	120.7 (79.3,152.5)	-62.7 (-73.5,-45)
Democratic People's Republic of Korea	4.7 (3.2,6.7)	4.3 (3,6.1)	-8.2 (-38.3,34.1)	4.4 (3,6.2)	3.7 (2.5,5.2)	-17.2 (-43.4,22.8)	220.5 (146.2,312.4)	167.2 (112.2,241.8)	-24.2 (-49.9,15.8)
Taiwan (Province of China)	2.8 (2.5,3)	4.2 (3.7,4.8)	52.3 (27.4,80.4)	2.3 (2.1,2.5)	2.5 (2.2,2.8)	9.9 (-4.4,24.7)	97.2 (90.4,104.3)	93.7 (83.1,104.6)	-3.6 (-16.2,10.4)
Southeast Asia	5.1 (3.7,6.5)	4.2 (3.2,5.2)	-17.5 (-31.9,3.6)	5 (3.7,6.4)	3.9 (3,4.9)	-22.6 (-35.7,-4.2)	217.7 (146.5,290.7)	155.8 (117.3,192.4)	-28.4 (-43.3,-2.7)
Cambodia	6.9 (3.9,9.9)	5.4 (3.7,7.8)	-21.5 (-46.5,26.6)	7 (4,10)	5.3 (3.6,7.6)	-24.4 (-48,18.8)	304.8 (145.8,456)	208.3 (136.3,300.8)	-31.7 (-55.8,25.9)
Indonesia	5.1 (3.4,7.1)	4.5 (3.3,6.4)	-12 (-34,26.2)	5.1 (3.4,7.1)	4.4 (3.2,6.2)	-14.4 (-35.7,19.5)	224.7 (138.3,319.2)	170.7 (126.3,232.3)	-24.1 (-44.7,18.6)

Lao People's Democratic Republic	7.2 (3.6,10.6)	5 (3.4,6.9)	-30 (-53.8,23.1)	7.3 (3.7,10.8)	5 (3.4,6.8)	-32 (-54.9,16.7)	318.6 (141.4,496.8)	206.6 (129.7,290.2)	-35.2 (-60.1,29.8)
Malaysia	4.9 (3.9,6.4)	4.3 (3.6,6.3)	-12.8 (-29,8.9)	4.8 (3.9,6.5)	3.9 (3.2,6)	-18.5 (-33.9,0.9)	203.3 (148.8,253.4)	145.5 (119.9,193.8)	-28.4 (-42.6,-10.7)
Maldives	4.4 (2.1,6.6)	2 (1.5,2.7)	-53.8 (-72.9,15.2)	4.4 (2.1,6.6)	1.7 (1.2,2.1)	-62.2 (-77.6,-11)	181.3 (76.6,279.9)	59.2 (42.8,77.8)	-67.3 (-81.2,-13.2)
Mauritius	4.2 (3.9,4.5)	3.2 (2.9,3.5)	-23.8 (-32.8,-15.2)	3.7 (3.4,4)	2.8 (2.6,3.1)	-23.6 (-32.2,-14.8)	145.7 (135.8,155.8)	100.8 (91.8,108.1)	-30.8 (-38.3,-24)
Myanmar	8 (4.5,11.6)	4.9 (3.5,6.5)	-38.2 (-57.1,0.8)	8 (4.6,11.7)	4.8 (3.4,6.3)	-40.7 (-58.9,-4.9)	370 (175.5,567)	204 (140.7,275.5)	-44.9 (-63.3,0.8)
Philippines	4.7 (3.5,5.7)	4.1 (3.2,5.1)	-13.9 (-31.6,5.9)	4.7 (3.6,5.7)	3.9 (3.1,4.9)	-17.6 (-34.8,1.6)	207.9 (154.5,259.6)	165.1 (123.9,204.3)	-20.6 (-37.3,2.4)
Sri Lanka	4.8 (3.6,5.8)	3 (2,4.3)	-36.4 (-58.7,-7.6)	4.7 (3.6,5.8)	2.6 (1.8,3.7)	-44.8 (-63.7,-20.1)	194.7 (138.8,240.6)	95.3 (62.9,136.6)	-51 (-68.1,-26)
Seychelles	5.9 (4.6,7.2)	4.9 (3.8,6)	-18.2 (-34.7,0.6)	5.6 (4.4,6.8)	4.2 (3.2,5.2)	-25.9 (-41.2,-8.5)	218.6 (171.3,269.9)	149.5 (119,187.3)	-31.6 (-45.3,-16.5)
Thailand	5.7 (3.7,6.9)	5.3 (3,6.7)	-7.2 (-32,27.3)	5.5 (3.5,6.7)	4.3 (2.3,5.6)	-22.3 (-44.6,7.1)	213.8 (145.2,266.8)	157.8 (92.3,201.5)	-26.2 (-46.8,-0.8)
Timor-Leste	5.4 (3.2,7.8)	4.4 (3.2,5.9)	-18.8 (-42.8,22.1)	5.5 (3.3,7.8)	4.3 (3.1,5.9)	-21 (-44.1,17.4)	244.9 (121.3,372.1)	176.4 (123.2,242.5)	-28 (-51,25.2)
Viet Nam	2.8 (2.1,3.7)	2.1 (1.6,3)	-24.4 (-46.6,2.5)	2.8 (2.2,3.8)	1.8 (1.3,2.7)	-35.6 (-54.8,-10.5)	108.3 (81.8,142.6)	71.3 (50.7,97.6)	-34.2 (-53.4,-8.6)
Oceania	3.9 (2.2,5.2)	3.4 (2,4.5)	-12.9 (-28.4,9)	4 (2.2,5.3)	3.4 (2,4.4)	-15.2 (-30.6,6)	168.5 (86.6,232.4)	148.6 (85.8,206.3)	-11.9 (-30.4,12.5)
American Samoa	4 (3.1,5.2)	4.3 (3.2,5.5)	7.9 (-20.9,42.5)	4.1 (3.1,5.3)	4.1 (3.1,5.2)	0.5 (-26.8,33)	151.5 (114.2,196.9)	152.5 (112.9,199.7)	0.7 (-25.8,35.8)
Cook Islands	1.6 (1,2.1)	1.1 (0.7,1.5)	-34.7 (-60.7,16.1)	1.6 (1,2)	0.8 (0.6,1.1)	-49.8 (-66.3,-16.6)	58.9 (37.7,78)	29.8 (20.4,44.5)	-49.5 (-69.3,-2.9)
Micronesia (Federated States of)	5 (3.2,6.7)	4.2 (2.7,5.7)	-16.8 (-37.6,14.1)	5.2 (3.3,7)	4.1 (2.7,5.6)	-19.8 (-40.1,10.2)	205.5 (131.6,273.6)	158.9 (103.7,217)	-22.7 (-44.2,6.9)
Fiji	4.7 (2.2,6.3)	4.1 (1.9,5.8)	-12.5 (-36.8,23.3)	4.8 (2.2,6.3)	4.1 (1.9,5.8)	-13.2 (-37.5,21.5)	200.7 (94.8,267.3)	171.4 (77.4,242.2)	-14.6 (-38.9,20.5)
Guam	3.9 (3,4.7)	2.5 (2,3.3)	-35.4 (-46.8,-22.2)	3.7 (2.9,4.6)	1.8 (1.5,2.3)	-50.5 (-59.1,-40)	132 (100.5,157.7)	71.4 (57.3,90.4)	-45.9 (-54.3,-35)

Kiribati	3.3 (2.6,4.6)	3.1 (2.2,4.4)	-5.8 (-29.2,25.9)	3.4 (2.6,4.8)	3.2 (2.3,4.5)	-6.2 (-29.4,24.8)	155.3 (121.9,202.5)	136.1 (94.4,191.9)	-12.4 (-36.8,24.3)
Marshall Islands	3.8 (2.5,5)	3.9 (2.3,5.7)	2.3 (-29.2,38.3)	3.9 (2.6,5.1)	3.9 (2.3,5.6)	-0.9 (-31.9,33.5)	151.5 (97.3,197.2)	158 (92.3,228.4)	4.3 (-28.7,39)
Nauru	5.8 (3.7,7.7)	5.3 (3,7.6)	-8.8 (-34.1,23.3)	5.9 (3.7,7.8)	5.1 (3,7.4)	-12.6 (-37.2,16.5)	233.6 (144.2,311.8)	209.8 (118.7,308.8)	-10.2 (-34.8,21.3)
Niue	3.6 (2.3,4.9)	6.7 (4.4,8.4)	85.3 (38.4,154.7)	3.6 (2.3,4.9)	5.7 (3.8,7.2)	58.5 (18.3,114.4)	141.8 (90.4,195.3)	337.4 (220.8,440.9)	138.1 (76.7,231.1)
Northern Mariana Islands	4.5 (2.8,5.9)	3 (2.3,3.7)	-33.3 (-51.9,-3.5)	4.3 (2.7,5.7)	2.6 (2.1,3.3)	-38.9 (-55.4,-11.3)	148.9 (92.3,200.3)	88.6 (68.5,113.3)	-40.5 (-58.3,-10.8)
Palau	3.6 (2.5,4.9)	3.4 (2.5,4.3)	-5.3 (-29.6,29.7)	3.5 (2.5,4.8)	3.2 (2.4,4.1)	-9.7 (-34.3,25.1)	138.8 (98.3,192.2)	121.4 (90.9,155.6)	-12.5 (-35.4,22.6)
Papua New Guinea	3.7 (2,5.4)	3.3 (1.9,4.6)	-12.4 (-35,22.9)	3.8 (2,5.4)	3.2 (1.9,4.5)	-14.2 (-36.1,19.9)	167.4 (80.6,249.1)	147.4 (83.3,216.1)	-11.9 (-36,27.3)
Samoa	5.2 (3.5,6.8)	5.3 (3.6,7.2)	2 (-26.5,38.2)	5.3 (3.5,6.9)	5.1 (3.4,6.9)	-4.2 (-31.6,28.9)	188.3 (135.7,247.7)	176.4 (121.7,236.6)	-6.3 (-31.4,24.5)
Solomon Islands	3.8 (1.8,5.5)	3.9 (2.5,5.4)	1.6 (-26.6,56.7)	4 (1.9,5.6)	3.9 (2.5,5.4)	-1.6 (-29,49.5)	154.2 (70.5,225.4)	156.2 (99.1,216.9)	1.3 (-26.9,58.4)
Tokelau	4.4 (2.8,5.8)	7.1 (4.4,10.7)	63.3 (12.2,156)	4.4 (2.8,5.9)	6.2 (3.9,8.9)	39.6 (0.6,112.1)	181 (112.9,243.4)	370.5 (219.8,588)	104.6 (35.7,235.8)
Tonga	1.5 (1.1,1.9)	1.4 (1,1.9)	-6.1 (-33.5,31.7)	1.5 (1.1,1.9)	1.3 (0.9,1.8)	-11.3 (-37.9,24.1)	58.3 (41.5,73)	51.5 (34.9,71)	-11.6 (-39.1,27.9)
Tuvalu	4.7 (2.9,6.4)	3.6 (2.5,4.9)	-22.3 (-41.2,7.5)	4.8 (3,6.4)	3.6 (2.4,4.9)	-25.6 (-43.4,2.1)	194.6 (118.6,273.5)	136.8 (90,186.9)	-29.7 (-48.5,6.1)
Vanuatu	3.2 (1.9,4.4)	3.1 (2,4.1)	-2.7 (-27.5,30.4)	3.3 (2,4.5)	3.2 (2,4.1)	-5.1 (-28.6,25.9)	127.4 (76.6,178)	124.6 (78.8,166.5)	-2.2 (-28.1,31.3)
North Africa and Middle East	5.8 (4.3,6.9)	5.2 (3.5,6.1)	-10.2 (-25.4,13.5)	5.6 (4.2,6.8)	4.3 (3,5)	-23.5 (-36.1,-7)	233.9 (164.8,288.6)	156.7 (107.2,186.9)	-33 (-44.6,-14.9)
Afghanistan	12.7 (4.6,19.8)	12.1 (5.8,18.7)	-4.6 (-28.7,38.1)	12.9 (4.8,20)	12 (5.9,18.7)	-6.6 (-30,31.9)	516.9 (168.1,842.8)	463.3 (212.3,724.1)	-10.4 (-33.8,40.5)
Algeria	3 (2.1,3.8)	2.5 (1.7,3.2)	-17.8 (-36.8,9.7)	2.9 (2,3.6)	2.2 (1.5,2.8)	-25.7 (-42.9,-2.4)	114.5 (82.6,146.6)	72.1 (50.1,94.6)	-37 (-51.9,-12.8)
Bahrain	6.3 (4.2,7.9)	4.1 (3,5.2)	-35.6 (-51.1,-14.7)	6.3 (4.2,7.9)	3.5 (2.6,4.5)	-44.2 (-58.4,-26.1)	199.8 (134.9,248.7)	101.9 (76.4,132.4)	-49 (-61.2,-30.4)
Egypt	4.2 (3.4,8.1)	6.7 (4.4,8.4)	57.5 (-22,124.4)	4.3 (3.4,8.5)	6.2 (4.3,7.6)	43.6 (-31,102.2)	172.7 (140.8,316.1)	197.6 (127.6,249.6)	14.4 (-42,69)

Iran (Islamic Republic of)	7.2 (4.1,8.8)	5.5 (3.1,6.5)	-23.3 (-36.8,0.9)	6.7 (3.9,8.1)	4.1 (2.5,4.7)	-38.9 (-48.2,-21.4)	293.9 (159.8,361.7)	145.8 (87.6,169.3)	-50.4 (-59.6,-31.6)
Iraq	6 (4.7,8.9)	5.9 (4.2,7.8)	-1.5 (-30.5,41.6)	5.7 (4.5,8.3)	4.6 (3.5,6.1)	-18.9 (-40.1,13.5)	239.2 (182.7,354.4)	164.3 (122.5,221.6)	-31.3 (-51.1,3)
Jordan	7.8 (6,10)	5.9 (3.8,8.2)	-24.5 (-48.7,9.6)	7.6 (5.8,9.8)	4.5 (3,6.3)	-41.1 (-58.2,-16.9)	284.4 (216.9,362.3)	145.4 (99.4,203.6)	-48.9 (-64.2,-23.6)
Kuwait	5.6 (4.8,6.6)	3.2 (2.4,4)	-43.4 (-58,-26.3)	4.7 (4,5.4)	1.7 (1.4,2.1)	-63.2 (-70.9,-53.8)	160 (140.9,181.7)	59.8 (48.3,71.7)	-62.6 (-69.9,-53.4)
Lebanon	5.5 (3.8,7.9)	4.7 (3.3,6.5)	-13.7 (-40.7,47.1)	5.3 (3.7,7.6)	3.5 (2.6,4.6)	-34.4 (-52.4,7.3)	178.4 (117.8,269.9)	104.5 (78.6,144.9)	-41.4 (-59.1,-0.7)
Libya	7.3 (5.5,9.4)	8 (5.5,10.8)	9.4 (-27.9,57.6)	7 (5.2,9.1)	6.7 (4.7,8.9)	-4.3 (-35.8,35.9)	261.8 (203.4,339.9)	242.2 (163.1,328.4)	-7.5 (-39.4,31.4)
Morocco	0.9 (0.6,1.1)	0.9 (0.5,1.1)	-8.1 (-30.7,24.6)	0.9 (0.6,1.1)	0.8 (0.5,1)	-15.3 (-36.6,14.9)	38.4 (27,49.3)	27.5 (18.8,38.2)	-28.3 (-50.9,4.3)
Palestine	7.2 (5.2,9.6)	5.6 (4.3,7.2)	-21.9 (-42.2,7.6)	6.7 (4.9,8.9)	4.3 (3.4,5.5)	-35.5 (-51.7,-13.4)	239.1 (173.3,328.7)	138.7 (109.9,180.1)	-42 (-57.5,-16.2)
Oman	3.6 (2.5,4.8)	2.6 (2,3.5)	-26.3 (-49.3,8.3)	3.5 (2.4,4.8)	2.3 (1.6,3)	-35.9 (-55.5,-5.9)	124.9 (86.9,166.5)	70.1 (52,94.2)	-43.8 (-61.6,-17)
Qatar	6.1 (3.8,7.8)	3.7 (2.6,5)	-39.6 (-56.1,-14.7)	6 (3.9,7.7)	2.9 (2,3.9)	-51.8 (-64.8,-33.3)	183 (119.5,231.8)	77.2 (55.2,103.2)	-57.8 (-69.3,-40.4)
Saudi Arabia	2.9 (2,5.7)	4 (2.9,6.5)	39 (-10.9,111.9)	2.8 (2,5.6)	3.1 (2.3,4.9)	10.5 (-31.7,65.4)	115 (80.4,215.4)	107.3 (77.5,166)	-6.7 (-38.1,37.5)
Sudan	6.7 (3.6,10.3)	5.6 (3.1,8.2)	-16.3 (-44.4,36.8)	6.7 (3.6,10.1)	5.2 (3,7.5)	-22.5 (-48.9,23.8)	306.6 (135.5,495.9)	210.7 (114,309.8)	-31.3 (-58.1,34.9)
Syrian Arab Republic	8.2 (5.6,10.2)	6.6 (4.6,8.8)	-19.4 (-41.8,13.9)	8 (5.5,9.9)	5.4 (3.9,7.2)	-31.8 (-50.6,-4.2)	315.7 (221.9,399.7)	178.7 (128,241.9)	-43.4 (-58.1,-19.4)
Tunisia	3.5 (2.5,4.3)	2.8 (1.9,4)	-17.9 (-41.6,18.6)	3.2 (2.3,3.9)	2.2 (1.5,3)	-31.8 (-52.1,-2.5)	130.3 (97.9,163.5)	75.9 (52.6,105.6)	-41.8 (-59.7,-9.5)
Turkey	7.9 (5.3,9.8)	5.5 (3.7,6.9)	-30.2 (-46.4,-1.9)	7.6 (5.1,9.5)	4.1 (2.8,5.2)	-46.4 (-58.6,-27.6)	322.4 (207,415.4)	135 (92.5,168.8)	-58.1 (-68.9,-36.6)
United Arab Emirates	10.7 (6.6,14.6)	13.1 (7,18.8)	21.9 (-23.6,93.8)	10.4 (6.3,14.4)	11.7 (6.2,16.5)	12.6 (-30.9,78.2)	328.8 (214.7,438.6)	263.6 (170.2,360.9)	-19.8 (-43.6,17.3)
Yemen	5.7 (3.1,8.7)	5.2 (2.8,7.7)	-7.5 (-35.2,36.2)	5.7 (3.2,8.6)	5 (2.7,7.3)	-11.5 (-38,29.9)	230.1 (113.6,370.7)	186.7 (97.3,277.4)	-18.9 (-45.9,36.3)
South Asia	2.9 (2.1,3.7)	2.4 (1.9,3)	-17.6 (-31,4.6)	2.9 (2.1,3.7)	2.3 (1.8,2.9)	-21 (-33.3,-2.5)	127.7 (86.7,170.5)	91.5 (72.3,114.9)	-28.4 (-42.4,2.4)

Bangladesh	3.2 (2.1,4.3)	2.2 (1.6,3.2)	-30.9 (-54.5,17.1)	3.3 (2.1,4.3)	2.1 (1.5,3)	-35.3 (-57,7.5)	153.5 (87.9,220.2)	91.6 (65,132.1)	-40.4 (-62.3,16.8)
Bhutan	3 (1.8,4.1)	2.5 (1.6,3.9)	-18.5 (-49.5,47.4)	3.1 (1.9,4.2)	2.4 (1.6,3.7)	-22.1 (-50.4,32)	132.1 (69.7,189)	92.9 (58.7,153.5)	-29.7 (-61.1,50)
India	2.8 (2,3.6)	2.2 (1.8,2.8)	-19.9 (-34.8,4.2)	2.8 (2,3.6)	2.1 (1.7,2.7)	-23.1 (-37.1,-1.9)	121.5 (81.5,163.7)	81.1 (64.9,103.9)	-33.3 (-47.5,-2.7)
Nepal	3.2 (2.1,4.2)	2.4 (1.8,3.4)	-23.8 (-49.2,17.6)	3.3 (2.1,4.3)	2.4 (1.8,3.4)	-25.5 (-50.2,12.2)	141.5 (80.8,200.4)	90.1 (65.4,129)	-36.3 (-60.5,11.8)
Pakistan	3.3 (2.3,4.3)	3.5 (2.3,5.1)	5.2 (-23.1,40.7)	3.4 (2.4,4.3)	3.4 (2.3,5)	2.1 (-25.6,38)	142.8 (93.7,192.7)	147.4 (96.8,218.5)	3.2 (-23.7,37.4)
Southern Sub-Saharan Africa	2.6 (1.9,3.3)	3.3 (2.2,3.8)	24.6 (-1,48.3)	2.5 (1.8,3.1)	2.9 (2,3.4)	16.4 (-9.5,40.3)	91.9 (69.9,110.8)	98.2 (69.5,118.5)	6.9 (-11.6,27.6)
Botswana	2.3 (1.5,3.3)	2.4 (1.6,3.3)	3.5 (-32.5,57.5)	2.3 (1.5,3.3)	2.2 (1.5,3.1)	-3 (-37,43.2)	76.3 (51.2,110.1)	75.1 (50.1,103.9)	-1.6 (-35.5,48.6)
Lesotho	1.9 (1.3,2.7)	3.5 (2.1,5.1)	87.6 (3.3,200.8)	1.9 (1.3,2.7)	3.5 (2.1,5)	83.4 (-0.1,195.5)	61.7 (41.7,91.5)	112.8 (67.7,164)	82.9 (3.8,185.3)
Namibia	1.9 (1.4,2.5)	1.9 (1.3,2.7)	2.5 (-32.9,48.4)	1.8 (1.4,2.5)	1.7 (1.2,2.4)	-6.2 (-37.5,33.8)	66.6 (48.2,85.4)	61.6 (40.9,90.6)	-7.5 (-39.1,37.7)
South Africa	2.7 (1.9,3.4)	3.2 (2,3.7)	16 (-5.4,39.3)	2.6 (1.8,3.3)	2.8 (1.8,3.2)	6.8 (-14.5,28.2)	97.6 (73.9,118.2)	88.5 (63.5,103.3)	-9.3 (-24.6,7.7)
Eswatini	2.7 (2,3.8)	3.3 (2,5.1)	22.2 (-24.3,84)	2.7 (2,3.9)	3.1 (1.9,4.8)	17.3 (-25.8,74)	90.2 (65.2,134.1)	103.7 (62.7,162)	15 (-27.6,75.1)
Zimbabwe	2.5 (1.9,3.4)	3.9 (2.5,5.2)	55.6 (0.1,115.7)	2.5 (1.9,3.4)	3.8 (2.5,5.1)	53.5 (-0.8,112.3)	81.8 (61.8,105.5)	139.4 (87.9,193.5)	70.4 (11.1,138.4)
Western Sub-Saharan Africa	0.9 (0.7,1.1)	0.9 (0.5,1.2)	-0.6 (-24.5,26.6)	0.9 (0.7,1.2)	0.9 (0.5,1.1)	-4.6 (-26,21)	44.8 (29.5,56)	38.5 (21.3,50)	-14.1 (-37,16.4)
Benin	1.1 (0.8,1.4)	1.2 (0.6,1.7)	15.4 (-33.8,61.6)	1.1 (0.8,1.4)	1.2 (0.6,1.6)	10.6 (-35.8,56)	51 (35.8,66.5)	51.9 (23.8,73.5)	1.8 (-42.8,52.5)
Burkina Faso	1.1 (0.8,1.5)	1.3 (0.6,1.8)	17.2 (-28.3,59.4)	1.1 (0.8,1.5)	1.3 (0.6,1.7)	14.2 (-28.6,56.1)	51.6 (33.6,69)	56.7 (27.8,77.6)	9.8 (-33,58.7)
Cameroon	1.2 (0.9,1.6)	1.4 (0.6,2)	12.5 (-37.8,62)	1.2 (0.9,1.6)	1.3 (0.6,1.9)	7.4 (-39.7,56.3)	54.4 (37.5,71.7)	55.1 (24.9,79)	1.3 (-42.3,51.5)
Cabo Verde	2.1 (1.6,3.3)	2.8 (1.8,3.7)	32.6 (-20.8,93.8)	2 (1.5,3.2)	2.3 (1.6,2.9)	15.2 (-29.4,67.7)	91.4 (67.7,144.5)	82.9 (54.4,109.7)	-9.3 (-46.2,30.7)
Chad	0.9 (0.6,1.2)	1.3 (0.7,1.9)	45.2 (-8.9,106.3)	0.9 (0.6,1.2)	1.3 (0.7,1.9)	42.8 (-11.2,100.9)	41.2 (29.4,56)	55.5 (29.6,80.4)	34.5 (-17.5,97.1)

CÃ´te d'Ivoire	0.7 (0.5,0.9)	0.8 (0.5,1)	2.4 (-25.8,42.4)	0.7 (0.5,0.9)	0.7 (0.4,1)	-3.7 (-29.8,36.3)	32.3 (21.3,42.3)	30.3 (17.4,44.1)	-6.2 (-35.1,41.8)
Gambia	0.6 (0.4,0.7)	0.6 (0.4,0.8)	5.7 (-23.7,44.1)	0.5 (0.4,0.7)	0.5 (0.4,0.7)	0.4 (-27.8,37.3)	24.8 (15.4,34.4)	22.1 (14,32.6)	-11 (-39.3,27.4)
Ghana	1.9 (0.9,2.6)	1 (0.7,1.5)	-47.9 (-67.7,11.9)	1.9 (0.9,2.5)	0.9 (0.6,1.5)	-50.6 (-69.4,7.4)	85.3 (40.8,113.3)	37.8 (24.9,59.5)	-55.7 (-73.2,-4.3)
Guinea	0.5 (0.3,0.6)	0.4 (0.2,0.6)	-13.3 (-41.8,34.4)	0.5 (0.3,0.6)	0.4 (0.2,0.6)	-15.5 (-43.6,30.5)	24.2 (14.8,32.5)	18 (9.1,29.9)	-25.5 (-54.4,26.6)
Guinea-Bissau	1.4 (0.9,1.9)	1.6 (0.8,2.1)	16.4 (-25,64.2)	1.4 (0.9,1.9)	1.6 (0.8,2.1)	13.8 (-26.4,59.5)	63.3 (41,89.5)	62 (34.4,85)	-2.1 (-37.7,44.5)
Liberia	1.2 (0.8,1.5)	1.4 (0.7,2)	21.2 (-26.6,81)	1.2 (0.8,1.6)	1.3 (0.6,1.9)	13.3 (-31.9,69.9)	56.1 (34.6,79.4)	55.6 (26.4,77.1)	-0.9 (-43.6,65.5)
Mali	1.3 (0.9,1.6)	1.1 (0.6,1.5)	-17.6 (-41,15.4)	1.3 (0.9,1.6)	1 (0.6,1.5)	-20.4 (-43.2,10.7)	63.1 (38.1,84.5)	44.1 (24.8,67)	-30.2 (-53.6,7.4)
Mauritania	1.1 (0.8,1.4)	1.3 (0.7,1.8)	19.7 (-29.7,74.7)	1.1 (0.8,1.4)	1.2 (0.6,1.6)	6.2 (-35.4,51.7)	45.9 (34.6,60)	44 (23.4,60.5)	-4 (-42.9,38.1)
Niger	1.2 (0.8,1.6)	1.2 (0.6,1.7)	2.4 (-39.9,60.2)	1.2 (0.8,1.6)	1.2 (0.6,1.7)	0.5 (-39.7,57)	59.4 (35.9,87.3)	48.7 (23.8,71.8)	-18 (-53.3,41.5)
Nigeria	0.7 (0.5,0.9)	0.7 (0.4,1)	0.7 (-35.1,63.6)	0.7 (0.5,1)	0.7 (0.4,1)	-4.4 (-37,52.2)	34.8 (23.7,47.6)	30.5 (17.4,44)	-12.4 (-45.7,51.5)
Sao Tome and Principe	1.1 (0.7,1.4)	1 (0.7,1.4)	-3.5 (-29.9,30.2)	1 (0.7,1.3)	0.9 (0.6,1.3)	-12.6 (-36.5,16.9)	50 (33.1,67.8)	35.5 (21.7,55.9)	-28.9 (-52.6,7.7)
Senegal	1.1 (0.8,1.4)	1.3 (0.7,1.8)	19.4 (-20.5,65.3)	1.1 (0.8,1.4)	1.2 (0.6,1.7)	13.9 (-23.1,58.5)	50.3 (34.6,66.6)	48.7 (24.7,71.4)	-3.2 (-36.9,45.6)
Sierra Leone	1 (0.7,1.3)	1.3 (0.6,1.8)	31.5 (-17.5,84)	1 (0.7,1.3)	1.3 (0.6,1.8)	26.1 (-20,79.3)	49.5 (30.6,67.8)	55 (25.5,77.4)	11.2 (-32.7,69.2)
Togo	1 (0.7,1.3)	1.3 (0.6,1.8)	26.3 (-22.8,74.2)	1 (0.7,1.3)	1.2 (0.6,1.7)	21.2 (-24.9,67.1)	45.7 (31.5,59.3)	48.8 (23.8,68.6)	6.8 (-35.3,50.7)
Eastern Sub-Saharan Africa	3.9 (2.4,5.3)	3.1 (2.1,4.1)	-20.8 (-43.6,25.5)	3.9 (2.5,5.3)	2.9 (2,3.9)	-24.3 (-45.8,18.4)	166.5 (101.1,239.8)	112.9 (77.9,149.9)	-32.2 (-53.7,17.1)
Burundi	2.2 (1.5,3.1)	2 (1.2,2.8)	-10.1 (-45.1,54.5)	2.3 (1.6,3.2)	2 (1.2,2.7)	-11.8 (-45.4,50.5)	103.5 (68.3,150.2)	79.7 (44.9,117.7)	-23 (-54.7,44.2)
Comoros	2.2 (1.5,2.8)	2.5 (1.7,3.3)	14.7 (-18.8,62.6)	2.1 (1.5,2.8)	2.3 (1.6,3.1)	9.6 (-21.9,54.4)	94 (63.9,127)	96.1 (64.1,137.3)	2.2 (-29.5,52.5)
Djibouti	1.7 (1.1,2.5)	2.1 (1.2,3.2)	20 (-19.5,82.6)	1.7 (1.1,2.5)	1.9 (1.1,3)	14.7 (-22,72.2)	73.4 (47.1,109.1)	72.2 (39.8,118.8)	-1.6 (-34.7,52.3)

Eritrea	2 (1.4,3.1)	2.4 (1.5,3.6)	20.8 (-19.6,92.5)	2 (1.4,3.1)	2.4 (1.5,3.5)	17.7 (-20.7,82)	87 (59.6,133.7)	93.3 (54.6,140.1)	7.2 (-35.4,85.6)
Ethiopia	9.4 (4.9,13.9)	6 (4,9)	-36.8 (-58.8,25)	9.6 (5.2,14.1)	5.7 (3.8,8.7)	-40.5 (-61,15.9)	389.2 (187,607)	215.3 (149.6,314.8)	-44.7 (-65.1,18.6)
Kenya	1.8 (1.3,2.6)	2 (1.4,2.9)	14.8 (-14.8,54.8)	1.7 (1.3,2.6)	1.9 (1.4,2.8)	11 (-17.9,47.6)	68 (49.4,95.5)	68 (46.8,98.4)	0 (-29.6,46.8)
Madagascar	1.9 (1.4,2.5)	1.9 (1.3,2.6)	2.6 (-28.6,45.2)	1.8 (1.4,2.6)	1.8 (1.2,2.5)	-0.5 (-30.3,38.7)	84.7 (62.8,121.6)	75.6 (48.8,103.3)	-10.8 (-40.1,35.1)
Malawi	0.8 (0.5,1.1)	0.7 (0.4,1.1)	-17.1 (-44.9,29.7)	0.8 (0.5,1)	0.6 (0.4,1)	-20.2 (-45.7,22.5)	41.7 (26,55)	27.2 (14.3,47.7)	-34.8 (-61.3,15.1)
Mozambique	3.3 (2.5,4.3)	3 (1.9,4.4)	-9.3 (-42.9,44.8)	3.4 (2.5,4.4)	3 (1.9,4.3)	-11.1 (-43.4,38)	159.7 (112.1,219.5)	114.7 (66.2,188.4)	-28.2 (-59.1,38.5)
Rwanda	2.8 (1.9,3.8)	2.3 (1.5,3.5)	-18.3 (-47.8,54)	2.8 (1.9,3.9)	2.2 (1.4,3.3)	-22.9 (-50.2,45.3)	127.7 (82.5,183.6)	83.9 (51,133.4)	-34.3 (-59.7,39.3)
Somalia	2.1 (1.3,3.3)	2.3 (1.5,3.4)	11.8 (-21.3,62.6)	2.1 (1.3,3.3)	2.4 (1.5,3.5)	11.4 (-21.8,61.5)	89 (52.3,144.7)	87.4 (54.5,128.1)	-1.9 (-34.1,49.8)
South Sudan	2 (1.3,3)	2.6 (1.6,3.7)	30.8 (-7.2,86.8)	1.9 (1.3,2.9)	2.4 (1.6,3.6)	26.1 (-12.1,80.2)	89.1 (55.3,140)	105 (63.9,157.7)	17.8 (-18.9,69.3)
United Republic of Tanzania	2.2 (1.7,2.9)	2.4 (1.5,3.3)	7 (-25.5,56.7)	2.2 (1.7,2.9)	2.2 (1.4,3)	1.6 (-29,48.7)	97.9 (75.5,132.9)	90.1 (54.7,132.7)	-8 (-39.2,43.2)
Uganda	1.2 (0.9,1.6)	1.4 (0.9,2)	17.8 (-19.2,71.5)	1.2 (0.9,1.6)	1.3 (0.8,1.9)	12.2 (-22.5,62.8)	53.4 (35.8,71.4)	56 (31.3,84.4)	4.8 (-30.3,62.9)
Zambia	2.3 (1.7,3.2)	2.3 (1.4,3.2)	-2.1 (-35.6,49.9)	2.3 (1.7,3.2)	2.2 (1.4,3)	-6.9 (-37.8,40.6)	108.8 (73.6,159)	86 (52.8,121.6)	-20.9 (-51,35.5)
Central Sub-Saharan Africa	1.7 (1.2,2.4)	1.7 (1,2.4)	-0.4 (-30.1,42.6)	1.7 (1.2,2.4)	1.7 (1,2.4)	-4.1 (-32.6,36.6)	75.5 (52.1,113.2)	63.8 (39.3,90.1)	-15.5 (-43.4,34.3)
Angola	1.8 (1.2,2.6)	1.7 (1,2.6)	-3.7 (-42.3,62.6)	1.8 (1.2,2.6)	1.7 (0.9,2.6)	-8 (-43.9,53.3)	81.9 (50.5,132.8)	65 (37.3,98.6)	-20.6 (-55.8,50.6)
Central African Republic	2 (1.4,2.9)	1.9 (1.1,2.9)	-4.8 (-33.7,35.2)	2 (1.4,3)	1.9 (1.2,2.9)	-5.2 (-34.8,33.4)	87 (55.4,136.7)	77.4 (47.8,125.5)	-11.1 (-40.4,36.9)
Congo	2 (1.4,2.8)	2.1 (1.3,2.9)	3.7 (-27.3,53.8)	2 (1.4,2.8)	2 (1.2,2.7)	-2.9 (-31.4,41.7)	79.7 (50.8,115)	73.2 (45,101.3)	-8.2 (-37.6,47.6)
Democratic Republic of the Congo	1.7 (1.1,2.3)	1.7 (1,2.5)	0.7 (-31.6,46.3)	1.7 (1.1,2.3)	1.6 (1,2.5)	-2.5 (-33.4,41.5)	72.8 (50,109.7)	61.9 (37.4,89.7)	-15 (-45.1,34.7)

Equatorial Guinea	1.8 (1.2,2.6)	1.8 (0.9,2.9)	-0.1 (-43.6,69)	1.8 (1.2,2.6)	1.6 (0.8,2.5)	-13 (-50.7,48.7)	77.1 (50.3,118.4)	59.2 (28.8,98)	-23.2 (-59.9,42.4)
Gabon	1.7 (1.2,2.2)	1.8 (1,2.7)	6.4 (-27.7,52.8)	1.7 (1.2,2.2)	1.7 (0.9,2.4)	-2.8 (-32.5,37.4)	65.7 (46.3,84.1)	58.7 (31.9,88.9)	-10.5 (-42.1,36.9)

Table S2 Cases and age-standardized rates of incidence, deaths, and DALYs in 2021, and the percentage change in the age-standardized rates from 1990 to 2021 for ALL in WCBA (15–49 years), globally and by 21 GBD regions.

	Incidence (95% UI)			Deaths (95% UI)			DALYs (95% UI)		
	No, in thousands (95% UI)	ASRs per 100000 (95% UI)	Percentage changes in ASRs from 1990 to 2021	No, in thousands (95% UI)	ASRs per 100000 (95% UI)	Percentage changes in ASRs from 1990 to 2021	No, in thousands (95% UI)	ASRs per 100000 (95% UI)	Percentage changes in ASRs from 1990 to 2021
Global	12.1 (7.2,14.2)	0.6 (0.4,0.7)	-31.1 (-55.6,-4)	9.4 (5.6,11.2)	0.7 (0.4,0.9)	-48.3 (-64.7,-31.7)	562.8 (332.1,662.7)	39.4 (24.7,46.4)	-54.8 (-70.3,-37)
**GBD regions**									
High-income Asia Pacific	0.2 (0.2,0.3)	0.6 (0.5,0.7)	-16.3 (-37.1,7.7)	0.1 (0.1,0.1)	0.3 (0.3,0.4)	-60.8 (-67.8,-55.7)	5.4 (4.4,6.2)	17.8 (15,20.1)	-65.4 (-71.9,-60.5)
High-income North America	0.5 (0.4,0.5)	0.5 (0.5,0.6)	-24.7 (-31.8,-15.8)	0.2 (0.2,0.2)	0.4 (0.4,0.4)	-41.3 (-43.8,-38.8)	13.1 (12.4,14)	19.8 (18.9,20.8)	-47.2 (-49.5,-44.5)
Western Europe	0.5 (0.5,0.6)	0.6 (0.5,0.6)	-15.1 (-26,-2.5)	0.2 (0.2,0.2)	0.3 (0.3,0.4)	-52.5 (-56,-48.9)	11.1 (10.5,11.8)	17.3 (16.2,18.7)	-57.4 (-60.6,-53.8)
Australasia	0 (0,0)	0.5 (0.4,0.6)	3.9 (-20.1,36.4)	0 (0,0)	0.3 (0.3,0.3)	-54.7 (-60.7,-47.6)	0.8 (0.7,1)	15.2 (13.3,17.1)	-56.8 (-62.7,-50)
Andean Latin America	0.2 (0.1,0.3)	1.2 (0.8,1.6)	6.9 (-37,54.6)	0.2 (0.1,0.3)	1.8 (1.1,2.2)	-11.2 (-45.2,21.6)	11.5 (7.4,15)	92.8 (58.6,118.3)	-23.2 (-55.5,11.6)
Tropical Latin America	0.3 (0.3,0.3)	0.5 (0.5,0.6)	-7.1 (-18.5,6.2)	0.3 (0.3,0.3)	0.7 (0.7,0.8)	-18.9 (-27.1,-10)	17.6 (16.4,18.7)	40.1 (36.5,44.3)	-29 (-38,-18.9)
Central Latin America	1 (0.9,1.1)	1.5 (1.3,1.7)	7.5 (-7.4,23.9)	0.9 (0.8,1)	1.7 (1.5,1.9)	-6.6 (-18.5,7)	53.6 (46.5,60.5)	90.4 (80,103.3)	-19.1 (-29.7,-6.5)
Southern Latin America	0.1 (0.1,0.1)	0.7 (0.6,0.8)	-0.3 (-20.8,26.1)	0.1 (0.1,0.1)	0.8 (0.7,0.9)	-23.5 (-35.3,-7.2)	5.8 (5.1,6.6)	42.2 (36.7,48.1)	-33.2 (-43.6,-19.2)
Caribbean	0.1 (0.1,0.1)	0.7 (0.4,1.1)	-14.7 (-35.7,10.7)	0.1 (0,0.1)	1.2 (0.8,1.9)	-16.2 (-37.3,10.3)	4.9 (3,8.1)	78.2 (44.7,133.7)	-19.9 (-41.3,8.8)
Central Europe	0.1 (0.1,0.1)	0.3 (0.2,0.3)	-40.1 (-49.5,-28.6)	0.1 (0.1,0.1)	0.4 (0.4,0.5)	-55.4 (-61.2,-49.1)	3.4 (2.9,3.9)	19.5 (17.2,22.2)	-62.7 (-68,-56.5)
Eastern Europe	0.2 (0.2,0.2)	0.4 (0.3,0.5)	-46.4 (-52.5,-39.4)	0.1 (0.1,0.2)	0.5 (0.5,0.6)	-58.7 (-63.5,-52.8)	8 (7,9.4)	24.5 (22.1,27.1)	-68.2 (-71.7,-64)

Central Asia	0.1 (0.1,0.1)	0.4 (0.4,0.5)	-47.7 (-57.7,-31.4)	0.1 (0.1,0.1)	0.6 (0.5,0.7)	-51.9 (-60.7,-37.2)	5.5 (4.6,6.7)	36.1 (30.1,44.5)	-54.9 (-63.9,-40.8)
North Africa and Middle East	1.3 (0.6,1.9)	0.8 (0.4,1.2)	-28.3 (-55.3,13.8)	1.2 (0.5,1.7)	1 (0.4,1.4)	-38.4 (-61.4,-10)	69.5 (30.4,101.1)	52.7 (25.1,71.2)	-44.7 (-65.9,-14.2)
South Asia	1.5 (0.8,2.1)	0.3 (0.2,0.4)	-36.7 (-59.8,-0.2)	1.4 (0.8,2)	0.5 (0.2,0.7)	-39.8 (-61.1,-8.5)	85.2 (47.3,125.3)	25.9 (15.3,36.6)	-44.9 (-66.1,-6.1)
Southeast Asia	1 (0.3,1.4)	0.6 (0.2,0.8)	-31.7 (-57.7,6.2)	1 (0.3,1.3)	0.8 (0.3,1.1)	-38.9 (-63.1,-7.3)	56.8 (17.4,77.5)	46.1 (17.8,60.2)	-42.4 (-68.5,-2.1)
East Asia	3.7 (1.5,5.1)	1.1 (0.4,1.6)	-9.7 (-62.5,55.7)	2.5 (1,3.5)	1.1 (0.5,1.4)	-63.5 (-82.3,-45.3)	141.2 (57,197.4)	57.6 (26.6,76.3)	-69.4 (-85.6,-52.6)
Oceania	0 (0,0)	0.3 (0.1,0.6)	-4.8 (-30.4,32.6)	0 (0,0)	0.6 (0.3,1.1)	-7.2 (-30.7,28.2)	0.7 (0.3,1.4)	39.9 (18.5,72.6)	-4.3 (-33.1,42)
Western Sub-Saharan Africa	0.2 (0.1,0.3)	0.2 (0.1,0.3)	-15.7 (-50,25.2)	0.2 (0.1,0.3)	0.3 (0.1,0.4)	-17.8 (-51.7,21.2)	13.5 (4.9,19.5)	18.3 (7.1,25.7)	-19.9 (-53.4,23.2)
Eastern Sub-Saharan Africa	0.7 (0.4,1)	0.7 (0.4,0.9)	-40.2 (-66.9,23)	0.7 (0.4,1)	0.9 (0.5,1.2)	-41.1 (-67.3,20.3)	42.8 (25.8,60.4)	50.2 (30.6,67.8)	-42.6 (-68,24.1)
Central Sub-Saharan Africa	0.1 (0.1,0.2)	0.3 (0.2,0.5)	-13.1 (-50.6,63)	0.1 (0.1,0.2)	0.4 (0.2,0.6)	-13.8 (-50.9,62.5)	7 (3.4,10.6)	25.4 (13.7,36.6)	-24 (-56.8,54.9)
Southern Sub-Saharan Africa	0.1 (0,0.1)	0.4 (0.2,0.7)	21.5 (-25.1,63.9)	0.1 (0,0.1)	0.6 (0.3,0.8)	20.9 (-26,62.9)	5.1 (2.6,7.8)	32.3 (17.9,45.7)	11 (-30.1,52.4)

Table S3 Cases and age-standardized rates of incidence, deaths, and DALYs in 2021, and the percentage change in the age-standardized rates from 1990 to 2021 for AML in WCBA (15–49 years), globally and by 21 GBD regions.

	Incidence (95% UI)			Deaths (95% UI)			DALYs (95% UI)		
	No, in thousands (95% UI)	ASRs per 100000 (95% UI)	Percentage changes in ASRs from 1990 to 2021	No, in thousands (95% UI)	ASRs per 100000 (95% UI)	Percentage changes in ASRs from 1990 to 2021	No, in thousands (95% UI)	ASRs per 100000 (95% UI)	Percentage changes in ASRs from 1990 to 2021
Global	15.2 (12,19.8)	0.8 (0.6,1)	-5.3 (-20.5,13)	12.4 (9.9,16.2)	1.4 (1.1,1.6)	-11.7 (-26.1,6.1)	697 (552.6,924.2)	46.6 (38,59.2)	-23.7 (-41.3,0)
**GBD regions**									
High-income Asia Pacific	0.4 (0.3,0.4)	1 (0.9,1.1)	-15.4 (-26.4,-6.1)	0.2 (0.2,0.2)	1.2 (1,1.3)	-27.3 (-37,-19.8)	12 (10.6,13.2)	38.3 (33.4,41.8)	-42.6 (-49.5,-34.5)
High-income North America	0.9 (0.9,1)	1.1 (1,1.1)	7.4 (2.4,11.6)	0.5 (0.5,0.6)	2.2 (2,2.4)	-4.2 (-8.7,-0.4)	28.9 (27.8,30.1)	59.8 (56.3,62.1)	-17.9 (-21,-14.9)
Western Europe	0.9 (0.8,0.9)	1 (0.9,1)	19.2 (10,27.9)	0.6 (0.5,0.6)	1.9 (1.7,2.1)	8.4 (-0.4,16.6)	29.6 (28.2,31)	52.6 (49.2,55.9)	-13.1 (-18.4,-7.4)
Australasia	0.1 (0.1,0.1)	1.4 (1.2,1.8)	25 (6.9,47.2)	0 (0,0.1)	2.1 (1.8,2.4)	6.6 (-8.6,25)	2.5 (2.1,2.9)	54.8 (48,62.4)	-11.1 (-23.2,3.1)
Andean Latin America	0.2 (0.1,0.2)	1 (0.6,1.3)	24.2 (-8.7,73.9)	0.1 (0.1,0.2)	1.6 (1.1,2.2)	17.8 (-13.9,63.7)	8.1 (5.3,11)	62.7 (41.2,81.1)	5.4 (-27.4,56.5)
Tropical Latin America	0.7 (0.6,0.7)	1.1 (1,1.1)	1.4 (-5.9,8.7)	0.6 (0.5,0.6)	1.6 (1.5,1.7)	-1.3 (-8.7,6.1)	30.6 (28.9,32.3)	57.5 (53.7,61.1)	-16.8 (-22.4,-10.5)
Central Latin America	0.7 (0.6,0.8)	1 (0.9,1.2)	14.6 (-2.5,31)	0.6 (0.5,0.7)	1.4 (1.2,1.6)	10.2 (-6.1,25.6)	34.4 (29.3,39.4)	55.2 (47.6,63.5)	-5.1 (-20.2,8.8)
Southern Latin America	0.2 (0.1,0.2)	0.9 (0.8,1)	-6 (-21.4,11.5)	0.1 (0.1,0.1)	1.5 (1.3,1.7)	-10.8 (-25.1,6.3)	7.1 (6.3,8)	52.8 (46.4,60.3)	-25.2 (-36.7,-11.9)
Caribbean	0.1 (0.1,0.2)	1 (0.8,1.3)	-3.6 (-18.7,11.7)	0.1 (0.1,0.1)	1.4 (1.1,1.7)	-6.8 (-21.8,8.4)	5.8 (4.3,7.6)	55.9 (42.4,75.4)	-14.8 (-30.6,2.4)
Central Europe	0.2 (0.2,0.3)	0.9 (0.8,1)	20.8 (8.2,33.7)	0.2 (0.2,0.2)	1.6 (1.5,1.8)	10.3 (-1.2,22.6)	9.4 (8.4,10.3)	50.5 (45.9,55.2)	-8.8 (-18.6,1.2)
Eastern Europe	0.5 (0.4,0.5)	1 (0.8,1.1)	-6.1 (-19.2,8.4)	0.4 (0.3,0.4)	1.2 (1.1,1.3)	-10.7 (-22.8,3.2)	18.6 (16.2,21.7)	43.6 (39.1,49)	-30.3 (-39.3,-19.3)

Central Asia	0.2 (0.2,0.2)	0.8 (0.7,1)	5.8 (-13.7,26)	0.2 (0.1,0.2)	1 (0.8,1.2)	3.6 (-15.2,23.8)	9.9 (8.2,11.7)	44.1 (37.3,51.8)	-7.2 (-25.1,11.8)
North Africa and Middle East	1.9 (1.3,2.7)	1.2 (0.8,1.7)	-6.7 (-26.3,22.2)	1.6 (1.1,2.3)	2 (1.4,2.8)	-12 (-30,15)	88.2 (60.7,129.7)	69.6 (47.9,100.3)	-20.3 (-39.4,7.4)
South Asia	2.7 (1.8,3.8)	0.5 (0.4,0.8)	5.8 (-25.3,67.2)	2.4 (1.7,3.5)	0.9 (0.6,1.3)	3.4 (-26.5,62.7)	145.7 (98.4,209)	35.9 (25.4,52)	-1.9 (-37.7,64.2)
Southeast Asia	2.7 (2,3.5)	1.5 (1.1,1.9)	0.3 (-21.9,30.6)	2.4 (1.8,3.1)	2.1 (1.5,2.8)	-2.7 (-23.7,26.8)	133.2 (97.5,176)	79.8 (58.3,107.8)	-8.2 (-32.3,26.9)
East Asia	2.3 (1.4,3.6)	0.7 (0.4,1.1)	-30 (-58.5,36.8)	1.7 (1.1,2.6)	0.8 (0.5,1.2)	-41.4 (-65,14.1)	93 (60.5,142.1)	33 (22.4,54)	-51.3 (-73,16.2)
Oceania	0 (0,0)	0.7 (0.3,1.1)	-25.2 (-46.6,23.6)	0 (0,0)	1 (0.5,1.5)	-25.6 (-46.5,24.5)	1.5 (0.7,2.2)	48.4 (22.5,70.7)	-22.5 (-46.2,33)
Western Sub-Saharan Africa	0.1 (0.1,0.2)	0.1 (0.1,0.2)	-2.5 (-27.1,26.5)	0.1 (0.1,0.2)	0.3 (0.2,0.4)	-2.8 (-27.2,25.8)	8.2 (5.1,12.7)	12 (7.5,16.8)	-9 (-36.1,26.1)
Eastern Sub-Saharan Africa	0.3 (0.2,0.5)	0.3 (0.2,0.4)	-8.7 (-44.7,70.1)	0.3 (0.2,0.5)	0.5 (0.3,0.7)	-9.4 (-45,65.2)	18.2 (9.5,28)	23.1 (12.1,35.3)	-16.3 (-56.1,99.4)
Central Sub-Saharan Africa	0.1 (0.1,0.2)	0.3 (0.2,0.5)	-5 (-39.9,43)	0.1 (0.1,0.2)	0.5 (0.3,0.8)	-5.7 (-41,43.7)	5.7 (3,9)	20.6 (11,31.2)	-12 (-48.1,42.7)
Southern Sub-Saharan Africa	0.1 (0.1,0.2)	0.6 (0.4,0.9)	4.1 (-23.1,32.7)	0.1 (0.1,0.2)	1 (0.5,1.5)	3.4 (-25.3,34.5)	6.6 (4.2,9.6)	36.1 (20.9,53.8)	-4.1 (-27.4,22.2)

Table S4 Cases and age-standardized rates of incidence, deaths, and DALYs in 2021, and the percentage change in the age-standardized rates from 1990 to 2021 for CLL in WCBA (15–49 years), globally and by 21 GBD regions.

	Incidence (95% UI)			Deaths (95% UI)			DALYs (95% UI)		
	No, in thousands (95% UI)	ASRs per 100000 (95% UI)	Percentage changes in ASRs from 1990 to 2021	No, in thousands (95% UI)	ASRs per 100000 (95% UI)	Percentage changes in ASRs from 1990 to 2021	No, in thousands (95% UI)	ASRs per 100000 (95% UI)	Percentage changes in ASRs from 1990 to 2021
Global	3.9 (2,5.4)	0.2 (0.1,0.3)	-13.8 (-27.6,-1.9)	1 (0.5,1.4)	0.4 (0.3,0.4)	-37.3 (-45.3,-29.3)	53.8 (24.6,73)	8.2 (5.6,9.5)	-38.4 (-49.2,-25.8)
**GBD regions**									
High-income Asia Pacific	0 (0,0)	0 (0,0)	-4.2 (-19.5,12.1)	0 (0,0)	0 (0,0.1)	-39.2 (-49.3,-31)	0.1 (0.1,0.1)	0.9 (0.7,1)	-42.4 (-50.3,-34.8)
High-income North America	0.1 (0.1,0.1)	0.1 (0.1,0.2)	-37.7 (-41.2,-34.4)	0 (0,0)	0.7 (0.6,0.7)	-42.7 (-46.7,-39.9)	0.9 (0.8,1)	11.6 (10.1,12.6)	-50 (-53.1,-47.6)
Western Europe	0.2 (0.2,0.2)	0.2 (0.2,0.3)	-6.1 (-14.5,3)	0 (0,0)	0.7 (0.5,0.7)	-34.1 (-41.6,-27.2)	1.2 (1,1.3)	11.6 (9.9,12.9)	-40 (-45.7,-33.7)
Australasia	0 (0,0)	0.2 (0.1,0.2)	-12.6 (-32.1,8.5)	0 (0,0)	0.5 (0.4,0.7)	-34.8 (-48.6,-19.4)	0.1 (0,0.1)	9.8 (7.6,12)	-39.8 (-52.9,-26.7)
Andean Latin America	0 (0,0)	0.1 (0.1,0.2)	67.4 (16.2,134.9)	0 (0,0)	0.2 (0.1,0.3)	6.3 (-24.4,48.7)	0.5 (0.2,0.7)	5.2 (2.8,7.3)	5.5 (-26.3,48.8)
Tropical Latin America	0 (0,0.1)	0.1 (0.1,0.1)	27.1 (14,40.7)	0 (0,0)	0.4 (0.3,0.4)	-2.1 (-12.7,8.3)	0.8 (0.7,1)	7.3 (6.5,8)	-6.9 (-16.2,2.8)
Central Latin America	0.1 (0,0.1)	0.1 (0.1,0.1)	3.9 (-10.3,19.6)	0 (0,0)	0.3 (0.2,0.3)	-25.2 (-35.5,-14.3)	1.1 (0.9,1.3)	5.2 (4.5,6)	-26.8 (-37,-15.1)
Southern Latin America	0 (0,0)	0.1 (0,0.1)	-18.9 (-35.4,3.4)	0 (0,0)	0.3 (0.3,0.4)	-41.5 (-52.7,-26.7)	0.1 (0.1,0.2)	5.9 (5,7)	-45.5 (-56.6,-30.8)
Caribbean	0 (0,0)	0.1 (0.1,0.1)	-9.9 (-25.5,7.3)	0 (0,0)	0.4 (0.3,0.5)	-31.7 (-43.1,-18.9)	0.2 (0.1,0.3)	8.1 (6.7,9.7)	-31.8 (-43.7,-18.5)
Central Europe	0.1 (0.1,0.1)	0.3 (0.2,0.3)	90.5 (65.8,121)	0 (0,0)	0.8 (0.7,0.9)	8.8 (-4.4,24.6)	0.6 (0.5,0.7)	16.4 (14.3,18.1)	5 (-8.1,20.1)
Eastern Europe	0.1 (0.1,0.2)	0.3 (0.2,0.3)	51.3 (27.7,87.5)	0 (0,0)	0.6 (0.6,0.7)	7.6 (-8.9,31.1)	1.5 (1.3,1.8)	14.6 (12.9,16.8)	-3.6 (-19.7,19.7)

Central Asia	0 (0,0)	0.2 (0.1,0.2)	-21.5 (-40.9,9.7)	0 (0,0)	0.2 (0.2,0.3)	-34.7 (-50.8,-9.3)	0.7 (0.6,1)	5.6 (4.6,6.8)	-42.1 (-56,-19.6)
North Africa and Middle East	0.3 (0.1,0.4)	0.2 (0,0.2)	44.9 (4.6,100.7)	0.1 (0,0.1)	0.5 (0.2,0.7)	-8.4 (-33.6,26)	4.6 (0.9,6.9)	10.5 (3.2,13.9)	-10.5 (-36.9,24.5)
South Asia	0 (0,0)	0 (0,0)	34.5 (-12.2,147.7)	0 (0,0)	0 (0,0)	2.5 (-32.7,93.3)	0.3 (0,0.5)	0.4 (0,0.5)	-1.3 (-34.6,81)
Southeast Asia	0.1 (0,0.1)	0 (0,0.1)	4.4 (-27.5,46)	0 (0,0.1)	0.1 (0,0.1)	-20.4 (-44.5,10)	1.8 (0.8,2.7)	2.1 (1,3.2)	-21.8 (-45.1,6)
East Asia	2.6 (1.1,3.9)	0.8 (0.3,1.2)	82.2 (7.7,241.2)	0.6 (0.2,0.9)	0.3 (0.1,0.4)	-31.7 (-58.4,24.2)	32 (12.6,48.5)	10 (4.1,14.8)	-29.5 (-58.9,36.3)
Oceania	0 (0,0)	0 (0,0)	13.5 (-42.2,105)	0 (0,0)	0 (0,0)	11.8 (-44.6,114.1)	0 (0,0)	0.1 (0,0.1)	-1.1 (-43.4,68)
Western Sub-Saharan Africa	0 (0,0)	0 (0,0)	28.8 (-6.6,67.1)	0 (0,0)	0.3 (0.1,0.4)	17.6 (-14.4,50.7)	1.1 (0.3,1.7)	6.5 (2.5,8.5)	13.7 (-17.8,49.5)
Eastern Sub-Saharan Africa	0.1 (0,0.2)	0.1 (0,0.2)	7.1 (-28,61.6)	0.1 (0,0.1)	1 (0.5,1.4)	-2.2 (-32.2,45.7)	3.7 (1.4,6.2)	20 (9.3,29.1)	-9.5 (-43,41.4)
Central Sub-Saharan Africa	0 (0,0)	0.1 (0,0.1)	25.4 (-14.2,79.5)	0 (0,0)	0.6 (0.2,0.9)	14.2 (-22,61.8)	1 (0.2,1.6)	13.3 (4.4,20.1)	12.2 (-25.1,64.1)
Southern Sub-Saharan Africa	0.1 (0,0.1)	0.2 (0.1,0.4)	48.9 (10.2,106.1)	0 (0,0)	1.2 (0.5,1.6)	31.4 (-1.4,83.5)	1.4 (0.4,2.5)	25.8 (9.8,34.4)	27.6 (-3.8,72.5)

Table S5 Cases and age-standardized rates of incidence, deaths, and DALYs in 2021, and the percentage change in the age-standardized rates from 1990 to 2021 for CML in WCBA (15–49 years), globally and by 21 GBD regions.

	Incidence (95% UI)			Deaths (95% UI)			DALYs (95% UI)		
	No, in thousands (95% UI)	ASRs per 100000 (95% UI)	Percentage changes in ASRs from 1990 to 2021	No, in thousands (95% UI)	ASRs per 100000 (95% UI)	Percentage changes in ASRs from 1990 to 2021	No, in thousands (95% UI)	ASRs per 100000 (95% UI)	Percentage changes in ASRs from 1990 to 2021
Global	3.9 (2.8,5.2)	0.2 (0.1,0.3)	-54.4 (-62.3,-43.3)	2.3 (1.6,3.3)	0.2 (0.2,0.3)	-61.9 (-69.3,-51.4)	127.6 (87.8,183.2)	7.3 (5.4,10)	-63.7 (-72.4,-50.9)
**GBD regions**									
High-income Asia Pacific	0.1 (0.1,0.2)	0.3 (0.2,0.4)	-65.1 (-71.2,-55.3)	0 (0,0)	0.1 (0.1,0.1)	-80.6 (-83.9,-75.5)	1.2 (1,1.6)	3.4 (2.8,4.2)	-84.3 (-86.8,-79)
High-income North America	0.2 (0.2,0.3)	0.3 (0.3,0.3)	-64.9 (-67.2,-62.4)	0.1 (0.1,0.1)	0.2 (0.2,0.3)	-72.7 (-74.9,-70.8)	3.5 (3.2,3.8)	5.9 (5.4,6.3)	-78 (-79.3,-76.5)
Western Europe	0.3 (0.3,0.4)	0.4 (0.3,0.4)	-56 (-61.6,-48.5)	0.1 (0,0.1)	0.2 (0.2,0.3)	-75.8 (-79.3,-71.6)	2.8 (2.6,3.2)	4.9 (4.4,5.6)	-80.5 (-82.8,-77.3)
Australasia	0 (0,0)	0.3 (0.2,0.5)	-67.2 (-73.9,-58.6)	0 (0,0)	0.2 (0.1,0.2)	-78.1 (-83,-72.2)	0.2 (0.2,0.3)	4.4 (3.5,5.4)	-80.9 (-84.6,-76.6)
Andean Latin America	0 (0,0.1)	0.2 (0.1,0.3)	-10.3 (-42.5,44.8)	0 (0,0)	0.3 (0.2,0.4)	-25.8 (-52.2,15.7)	1.4 (0.9,2.1)	9.2 (5.8,12.8)	-35.8 (-59.4,1.8)
Tropical Latin America	0.1 (0.1,0.1)	0.2 (0.1,0.2)	-59.4 (-64.6,-53.4)	0.1 (0.1,0.1)	0.3 (0.2,0.3)	-63.5 (-68.3,-58)	3.3 (3,3.8)	6.8 (6,7.8)	-69.5 (-73.2,-65.2)
Central Latin America	0.1 (0.1,0.2)	0.2 (0.2,0.2)	-44.1 (-54.1,-33.8)	0.1 (0.1,0.1)	0.3 (0.2,0.3)	-53.4 (-61.8,-45.1)	5 (4.2,5.8)	8.2 (7.1,9.5)	-57.4 (-65.3,-49.4)
Southern Latin America	0 (0,0)	0.2 (0.1,0.2)	-61.2 (-73.4,-46)	0 (0,0)	0.3 (0.2,0.3)	-69.1 (-79.2,-56.3)	0.9 (0.7,1.1)	6.9 (5.7,8.4)	-74 (-82.5,-63.1)
Caribbean	0 (0,0)	0.3 (0.2,0.4)	-35.7 (-50.2,-18.2)	0 (0,0)	0.5 (0.4,0.6)	-45.2 (-57.3,-29.9)	1.3 (0.8,2.1)	15.1 (10.7,24.1)	-47.7 (-63.1,-28.5)
Central Europe	0 (0,0.1)	0.2 (0.1,0.2)	-55.3 (-63.8,-42.1)	0 (0,0)	0.2 (0.2,0.2)	-71.2 (-76.7,-62.3)	0.8 (0.7,1)	4.8 (4.2,5.6)	-75.3 (-80,-67.1)
Eastern Europe	0.1 (0.1,0.1)	0.2 (0.2,0.3)	-28.7 (-50.7,-5.7)	0.1 (0,0.1)	0.3 (0.2,0.3)	-46.5 (-63.7,-29.3)	2.8 (2.2,3.6)	7.2 (6,8.9)	-56.3 (-69.7,-41.5)

Central Asia	0 (0,0.1)	0.2 (0.1,0.2)	-42.8 (-61.5,-9.4)	0 (0,0)	0.2 (0.2,0.3)	-50.2 (-66.4,-21.3)	1.6 (1.2,2.2)	7.6 (5.7,10.2)	-54.5 (-68.9,-27.9)
North Africa and Middle East	0.5 (0.2,0.7)	0.3 (0.1,0.4)	-25 (-54.2,18.9)	0.3 (0.1,0.5)	0.5 (0.2,0.6)	-39.4 (-63.9,-8.1)	19 (7.9,27)	14.4 (6.6,19.6)	-45.4 (-67.5,-11.6)
South Asia	0.9 (0.6,1.5)	0.2 (0.1,0.3)	-30.7 (-60,15.7)	0.7 (0.5,1.2)	0.3 (0.2,0.5)	-35.1 (-62.6,7.8)	41.7 (28,68.4)	9.7 (6.7,15.1)	-44.1 (-66.6,-1.7)
Southeast Asia	0.5 (0.4,0.9)	0.3 (0.2,0.5)	-40.4 (-58.2,2.1)	0.4 (0.3,0.7)	0.4 (0.2,0.6)	-46.2 (-62.3,-9.5)	23 (15,37.5)	12.6 (8.4,20.9)	-51.6 (-67.8,-11.6)
East Asia	0.5 (0.3,0.8)	0.2 (0.1,0.3)	-51.5 (-73.3,-2.5)	0.2 (0.1,0.3)	0.1 (0.1,0.1)	-75.8 (-86.9,-49.6)	10.2 (6.5,16.9)	3.1 (2,5.2)	-79.5 (-89.2,-54.8)
Oceania	0 (0,0.1)	1 (0.6,1.5)	-6.2 (-28.7,25.2)	0 (0,0)	1.6 (0.9,2.4)	-9.8 (-31.2,19.9)	1.6 (0.9,2.4)	59.4 (33.8,90.3)	-5.8 (-30.8,30.1)
Western Sub-Saharan Africa	0 (0,0.1)	0 (0,0)	-33.1 (-66.9,17.8)	0 (0,0.1)	0 (0,0.1)	-34.5 (-66.9,16.5)	1.1 (0.4,3)	1.6 (0.7,3.9)	-45.8 (-78.3,11.1)
Eastern Sub-Saharan Africa	0.1 (0,0.3)	0.1 (0,0.2)	-38.1 (-69.6,13.6)	0.1 (0,0.2)	0.1 (0.1,0.4)	-39.4 (-69.7,9.9)	4.9 (1.7,13.9)	5 (1.9,14.9)	-44.6 (-76.8,10)
Central Sub-Saharan Africa	0 (0,0)	0.1 (0,0.1)	-31.3 (-68.1,26.3)	0 (0,0)	0.1 (0,0.2)	-32.3 (-68.3,21)	0.8 (0.3,2.1)	3 (1,8)	-42.4 (-79.2,23.2)
Southern Sub-Saharan Africa	0 (0,0)	0 (0,0.1)	-23.7 (-64.2,40.5)	0 (0,0)	0 (0,0.1)	-27.6 (-67.6,39.1)	0.3 (0.1,0.6)	1.2 (0.6,2.2)	-32.5 (-64.9,19.2)

Table S6 Age-standardized incidence rates, death rates, and DALY rates in 1990 and 2021, and the percentage change in the age-standardized rates for ALL among WCBA (15–49 years), by country.

	Incidence (95% UI)			Deaths (95% UI)			DALYs (95% UI)		
	ASRs per 100000 (95% UI), 1990	ASRs per 100000 (95% UI), 2021	Percentage changes in ASRs from 1990 to 2021	ASRs per 100000 (95% UI), 1990	ASRs per 100000 (95% UI), 2021	Percentage changes in ASRs from 1990 to 2021	ASRs per 100000 (95% UI), 1990	ASRs per 100000 (95% UI), 2021	Percentage changes in ASRs from 1990 to 2021
Global	1.6 (1.2,2.2)	1.1 (0.7,1.4)	-31.1 (-55.6,-4)	1.4 (1,1.9)	0.7 (0.4,0.9)	-48.3 (-64.7,-31.7)	87.2 (59.1,120.7)	39.4 (24.7,46.4)	-54.8 (-70.3,-37)
High-income North America	1.9 (1.8,2.1)	1.5 (1.4,1.6)	-24.7 (-31.8,-15.8)	0.7 (0.7,0.7)	0.4 (0.4,0.4)	-41.3 (-43.8,-38.8)	37.5 (36.7,38.3)	19.8 (18.9,20.8)	-47.2 (-49.5,-44.5)
Canada	2.4 (1.8,3.3)	1.6 (1.3,2)	-34.2 (-56.7,-5.4)	0.7 (0.6,0.7)	0.3 (0.3,0.4)	-54.5 (-62.1,-46.4)	35.6 (31.8,39.4)	15.3 (12.9,18.3)	-56.9 (-64.7,-47.4)
Greenland	0.7 (0.2,0.9)	0.3 (0.1,0.5)	-61.1 (-78.7,-16.4)	0.7 (0.2,0.9)	0.2 (0.1,0.4)	-69.9 (-82.8,-34.3)	31.8 (12.5,43.4)	9.1 (5,15.7)	-71.3 (-84.4,-35.6)
United States of America	1.9 (1.8,2)	1.4 (1.3,1.6)	-23.4 (-30.7,-14.7)	0.7 (0.7,0.7)	0.4 (0.4,0.5)	-39.8 (-42.3,-37.3)	37.7 (36.9,38.4)	20.3 (19.4,21.3)	-46.2 (-48.4,-43.7)
Australasia	1.3 (1.1,1.5)	1.4 (1.1,1.7)	3.9 (-20.1,36.4)	0.7 (0.6,0.7)	0.3 (0.3,0.3)	-54.7 (-60.7,-47.6)	35.2 (32.6,37.9)	15.2 (13.3,17.1)	-56.8 (-62.7,-50)
Australia	1.3 (1.1,1.6)	1.4 (1.1,1.8)	5.1 (-21.2,42.5)	0.7 (0.6,0.7)	0.3 (0.3,0.4)	-54.1 (-60.6,-45.8)	35.1 (32.1,38.3)	15.1 (13,17.4)	-56.9 (-63.5,-49.1)
New Zealand	1.2 (1,1.4)	1.1 (0.9,1.4)	-3.3 (-29.5,27.3)	0.6 (0.6,0.7)	0.3 (0.2,0.3)	-58.5 (-63,-53)	35.4 (32.8,38.5)	15.3 (13.9,16.9)	-56.8 (-62,-51)
High-income Asia Pacific	1.9 (1.6,2.4)	1.6 (1.3,1.9)	-16.3 (-37.1,7.7)	0.9 (0.7,1)	0.3 (0.3,0.4)	-60.8 (-67.8,-55.7)	51.4 (42.6,59.8)	17.8 (15,20.1)	-65.4 (-71.9,-60.5)
Brunei Darussalam	1.5 (0.8,2.3)	1 (0.4,1.3)	-35.2 (-61.1,-3.9)	1.4 (0.7,2.1)	0.8 (0.3,1.1)	-44 (-66.7,-15.2)	74.4 (37.6,109.6)	39.5 (17.8,53)	-47 (-68.3,-20.3)
Japan	2.2 (1.8,2.9)	1.7 (1.5,1.9)	-24.3 (-43.9,0.6)	0.7 (0.7,0.7)	0.3 (0.3,0.4)	-52.5 (-55,-50.3)	42 (41,43)	17.7 (16.9,18.6)	-57.7 (-59.7,-55.6)
Singapore	1.3 (1.1,1.6)	2 (1.5,2.5)	47.5 (7.6,94.6)	0.9 (0.8,1)	0.4 (0.4,0.5)	-50.6 (-57.7,-42.8)	48.9 (44.5,54)	24.7 (21.4,28.8)	-49.4 (-57.7,-40.6)
Republic of Korea	1.4 (0.8,2.1)	1.4 (0.6,2.3)	-1.2 (-59.1,64.7)	1.2 (0.7,1.7)	0.3 (0.1,0.5)	-74.8 (-88.3,-63.9)	72.4 (42.1,101.1)	16.6 (7.7,23.4)	-77 (-89.2,-66.8)
Western Europe	2.3 (2.1,2.5)	1.9 (1.8,2.2)	-15.1 (-26,-2.5)	0.7 (0.7,0.7)	0.3 (0.3,0.4)	-52.5 (-56,-48.9)	40.8 (39.1,42.2)	17.3 (16.2,18.7)	-57.4 (-60.6,-53.8)

Andorra	2.7 (1.1,5.9)	1.4 (0.7,2.4)	-49.7 (-79.9,62.8)	0.4 (0.2,0.7)	0.2 (0.1,0.3)	-54.3 (-74.8,-15.2)	26.1 (12.7,44.5)	10.6 (5.4,17.3)	-59.3 (-78.8,-13.2)
Austria	1.7 (1.2,2.4)	1.3 (1,1.6)	-26.3 (-51.4,15.3)	0.5 (0.5,0.6)	0.2 (0.2,0.3)	-55.6 (-63.6,-46.5)	29.3 (26,33.2)	11.3 (9.4,13.5)	-61.2 (-68.6,-51.9)
Belgium	2.5 (1.8,3.6)	1.9 (1.4,2.5)	-25.7 (-51.2,11.9)	0.7 (0.7,0.8)	0.3 (0.3,0.4)	-53.6 (-62.5,-43.9)	42 (37,48)	17.7 (15,20.8)	-57.9 (-65.8,-48.6)
Cyprus	1.5 (0.9,2.5)	1.7 (0.9,2.6)	13 (-44.7,100.9)	1 (0.6,1.6)	0.4 (0.2,0.6)	-58.6 (-79.9,-33.6)	41.9 (26.3,64.1)	17.9 (9.5,25.5)	-57.3 (-76.3,-35.2)
Denmark	1.2 (0.9,1.4)	1.6 (1.2,2.1)	38 (-2.9,88.6)	0.5 (0.5,0.6)	0.3 (0.3,0.4)	-41.8 (-51.1,-29.6)	27 (24.1,30.3)	15.3 (13.1,18)	-43.4 (-53.2,-31.6)
Finland	1.1 (0.9,1.3)	1.7 (1.3,2.2)	57.2 (13.3,114.1)	0.6 (0.5,0.6)	0.4 (0.3,0.4)	-34.8 (-44.3,-23.2)	27.4 (24.6,30.6)	18.3 (15.7,21.3)	-33.1 (-43.9,-19.4)
France	1.9 (1.5,2.7)	2 (1.5,2.5)	2.6 (-33.5,46.4)	0.7 (0.6,0.8)	0.3 (0.3,0.4)	-53.6 (-59.8,-45.8)	38.4 (35.3,41.6)	16.1 (13.9,18.9)	-58.2 (-64.9,-50.1)
Germany	1.7 (1.3,2.3)	2 (1.6,2.5)	15 (-19.8,62.5)	0.6 (0.6,0.7)	0.3 (0.3,0.4)	-49.5 (-57.6,-38.1)	36.1 (31.5,40.6)	17.1 (15.1,19.1)	-52.6 (-60.4,-43.3)
Greece	3.1 (2.3,4.2)	2.3 (1.7,2.7)	-27.8 (-51.9,4.6)	0.8 (0.7,0.8)	0.4 (0.4,0.5)	-41.7 (-47.7,-35)	41.4 (38.7,44.3)	22 (20.1,24.3)	-47 (-53.2,-40.7)
Iceland	2.2 (1.6,2.8)	2.2 (1.7,2.7)	1.8 (-27.2,45.6)	0.6 (0.5,0.7)	0.4 (0.3,0.4)	-39.8 (-51.1,-24.7)	31.2 (26.9,35.8)	18.2 (15.3,21.4)	-41.5 (-52.5,-25.7)
Ireland	1.8 (1.4,2.5)	1.2 (1,1.6)	-30.9 (-51.8,-1.9)	0.7 (0.7,0.8)	0.3 (0.2,0.3)	-64.3 (-70.6,-56.3)	37.5 (33.4,41.9)	11.9 (10.1,14)	-68.2 (-74.5,-61.5)
Israel	2 (1.5,2.9)	1.4 (1,1.8)	-31.4 (-55.4,3.8)	0.8 (0.7,0.9)	0.3 (0.3,0.4)	-59.5 (-66.1,-50.7)	46.6 (40.8,53.2)	15.7 (13.1,18.8)	-66.4 (-73.5,-57.2)
Italy	3.7 (2.9,4.9)	2.9 (2.4,3.3)	-23.1 (-44.4,5.9)	0.9 (0.9,0.9)	0.5 (0.4,0.5)	-48.7 (-52.3,-44.4)	53.9 (51.6,56.4)	24.3 (22.5,26.6)	-54.8 (-58.2,-50.7)
Luxembourg	2 (1.5,2.6)	1.1 (0.9,1.4)	-45.1 (-60.8,-19.2)	0.8 (0.7,0.8)	0.2 (0.2,0.2)	-71.6 (-75.6,-66.6)	45.3 (40.3,49.9)	9.9 (8.6,11.7)	-78.1 (-81.7,-73.6)
Malta	1.7 (1.3,2.2)	2.1 (1.6,2.7)	24.5 (-17.3,76.2)	0.7 (0.7,0.8)	0.4 (0.3,0.4)	-50.6 (-60.6,-39)	39 (33.3,44.7)	19.3 (15.9,22.8)	-50.5 (-60,-39.4)
Monaco	12 (4.6,22.5)	9.2 (3.9,16.1)	-23.5 (-62.8,59)	1.4 (0.8,2.1)	1.2 (0.6,1.8)	-15.6 (-43.7,22.8)	95.1 (51.5,144.4)	71 (37,105.1)	-25.4 (-50.6,11.9)
Netherlands	2.2 (1.6,2.8)	1.4 (1.1,1.8)	-34.8 (-55.8,-2.4)	0.5 (0.5,0.6)	0.3 (0.2,0.3)	-49.1 (-56.3,-40.4)	29.4 (26.9,32.1)	12.5 (11.1,14.4)	-57.4 (-64.2,-49.7)
Norway	1.4 (1.1,1.8)	1.6 (1.3,1.9)	16.4 (-11.6,54.2)	0.5 (0.4,0.5)	0.3 (0.3,0.3)	-31.1 (-36.9,-24.3)	25.2 (24,26.6)	16.7 (15.4,18.1)	-33.7 (-39.5,-27)

Portugal	2 (1.6,2.6)	2 (1.5,2.7)	2.2 (-31.6,48.5)	1 (0.9,1.1)	0.4 (0.3,0.4)	-62.8 (-68.1,-56.3)	60.4 (55,66.4)	19.5 (16.8,22.6)	-67.8 (-72.9,-61.5)
San Marino	10.9 (5.6,17.9)	6 (3.4,9.7)	-45.3 (-72.4,15.4)	1.8 (1.2,2.6)	0.9 (0.5,1.4)	-46.8 (-65.9,-19.2)	92.7 (60.7,131.7)	47.4 (28.6,72.4)	-48.9 (-67.7,-17.5)
Spain	2.9 (2.2,4)	2.8 (2.1,3.5)	-4.7 (-34.7,34.5)	0.9 (0.8,1)	0.4 (0.3,0.4)	-59.2 (-65.2,-52.3)	53.6 (48.6,58.8)	20.9 (17.9,24.9)	-61 (-67.5,-52.7)
Sweden	3 (2.2,4)	1.8 (1.5,2.3)	-40.1 (-57.3,-8.8)	0.5 (0.5,0.6)	0.3 (0.2,0.3)	-53.8 (-62,-43.8)	34.4 (30.8,38.2)	14.2 (12.1,17.3)	-58.6 (-66.3,-49.1)
Switzerland	2.8 (2.1,3.7)	2.1 (1.7,2.7)	-25.6 (-47.9,8)	0.6 (0.5,0.6)	0.3 (0.2,0.3)	-53.4 (-61.9,-42)	32.7 (28.5,36.7)	15 (12.8,17.7)	-54.1 (-62.1,-42.8)
United Kingdom	2 (1.8,2.2)	1.2 (1.1,1.3)	-36.8 (-45.9,-27.5)	0.6 (0.5,0.6)	0.3 (0.3,0.3)	-51.9 (-54.3,-49.1)	33.3 (32,34.6)	14.4 (13.8,15)	-56.8 (-59.4,-54.1)
Southern Latin America	1.2 (1.1,1.4)	1.2 (1,1.5)	-0.3 (-20.8,26.1)	1.1 (1,1.2)	0.8 (0.7,0.9)	-23.5 (-35.3,-7.2)	63.2 (57.4,68.9)	42.2 (36.7,48.1)	-33.2 (-43.6,-19.2)
Argentina	1.3 (1.1,1.5)	1.2 (1,1.5)	-7.1 (-29.5,22.8)	1.1 (1,1.3)	0.9 (0.8,1)	-23 (-36.4,-3.2)	66.5 (58.8,74)	45 (38.7,51.7)	-32.4 (-44.3,-15.6)
Chile	1.2 (1.1,1.3)	1.4 (1.1,1.8)	17.2 (-8.8,50.1)	1 (1,1.1)	0.8 (0.7,0.9)	-27.6 (-38.8,-15.8)	59.1 (54.3,64.1)	36.6 (31.7,41.9)	-38.1 (-47.3,-27.1)
Uruguay	1 (0.8,1.1)	0.9 (0.8,1.2)	-1 (-24,31.6)	0.8 (0.7,0.9)	0.6 (0.5,0.7)	-16.9 (-32,3.4)	46.8 (40.4,54.4)	32.4 (27.3,38)	-30.8 (-43.8,-13.7)
Eastern Europe	1.5 (1.4,1.6)	0.8 (0.7,0.9)	-46.4 (-52.5,-39.4)	1.3 (1.2,1.3)	0.5 (0.5,0.6)	-58.7 (-63.5,-52.8)	77.1 (72.1,82.4)	24.5 (22.1,27.1)	-68.2 (-71.7,-64)
Belarus	1.6 (1.3,1.9)	0.6 (0.5,0.8)	-60 (-71,-44.6)	1.3 (1.1,1.6)	0.4 (0.3,0.5)	-71.3 (-78.7,-62.1)	79.8 (67.6,94.5)	18.1 (14.3,22.1)	-77.4 (-83.1,-70.5)
Estonia	1.2 (1,1.5)	0.7 (0.5,0.9)	-47.1 (-62.2,-28.7)	1 (0.9,1.1)	0.3 (0.2,0.4)	-70.2 (-75.7,-64)	60.9 (53.1,70.4)	14.7 (12.4,17.6)	-75.9 (-80.5,-70.3)
Latvia	1.1 (1,1.4)	0.5 (0.4,0.6)	-58.1 (-68.1,-43.8)	1 (0.8,1.1)	0.3 (0.3,0.4)	-67 (-73.8,-58.8)	56 (48.6,64.8)	15.3 (13,18.3)	-72.7 (-78,-66.3)
Lithuania	1.1 (0.9,1.4)	0.5 (0.4,0.7)	-54.6 (-65.4,-40.1)	0.9 (0.8,1.1)	0.3 (0.3,0.4)	-61.2 (-69.6,-51.7)	55.4 (46.5,65)	16.8 (13.5,20.5)	-69.6 (-76.2,-61.7)
Republic of Moldova	1.9 (1.7,2.1)	0.7 (0.6,0.9)	-61.6 (-69.6,-49.8)	1.7 (1.5,1.9)	0.5 (0.5,0.6)	-69.1 (-73.7,-63.2)	101.5 (91.6,113)	26.2 (22,31.1)	-74.2 (-78.4,-68.8)
Russian Federation	1.3 (1.2,1.4)	0.8 (0.7,0.9)	-38.5 (-44.3,-30.5)	1.1 (1,1.2)	0.5 (0.5,0.5)	-54.8 (-59.4,-48)	66.5 (60.2,70.6)	22.7 (20.9,24.7)	-65.8 (-68.9,-61.6)
Ukraine	2.1 (1.8,2.5)	0.9 (0.7,1.3)	-55.9 (-70.2,-34.9)	1.7 (1.4,1.9)	0.6 (0.5,0.9)	-61.3 (-74.2,-43.7)	109.2 (93.4,124.3)	34.4 (25.5,46)	-68.5 (-78.3,-55)

Central Europe	1 (0.9,1.1)	0.6 (0.5,0.7)	-40.1 (-49.5,-28.6)	0.9 (0.8,1)	0.4 (0.4,0.5)	-55.4 (-61.2,-49.1)	52.3 (47.1,58.2)	19.5 (17.2,22.2)	-62.7 (-68,-56.5)
Albania	1.1 (0.6,1.6)	1.1 (0.5,1.9)	-0.8 (-46.9,76.6)	1 (0.6,1.5)	0.6 (0.3,1)	-45.2 (-68.2,-7.4)	64.4 (38.5,95.6)	32.7 (16.1,58)	-49.2 (-70.8,-9.4)
Bosnia and Herzegovina	0.6 (0.3,0.9)	0.4 (0.2,0.7)	-26.9 (-54.3,14.4)	0.6 (0.3,0.9)	0.4 (0.2,0.6)	-35.6 (-60.5,-1.4)	30 (18.7,41.7)	17.2 (8.1,26.6)	-42.7 (-63.5,-13.9)
Bulgaria	1 (0.8,1.2)	0.4 (0.3,0.6)	-55 (-67.4,-38.9)	0.9 (0.8,1.1)	0.4 (0.3,0.5)	-59.5 (-70,-45.8)	54 (44.1,65.2)	20 (15.2,25.5)	-62.9 (-72.3,-48.8)
Croatia	1 (0.8,1.2)	0.7 (0.5,1)	-28.9 (-51,5.7)	0.9 (0.7,1.1)	0.3 (0.3,0.4)	-60.8 (-70.4,-46.9)	38.3 (31.8,45.7)	16.9 (13.7,21.1)	-55.9 (-66.6,-40.7)
Czechia	1.2 (1.1,1.4)	0.7 (0.5,0.9)	-44.8 (-58.9,-26.4)	1.2 (1,1.3)	0.4 (0.4,0.5)	-62.8 (-70.5,-52.8)	53.8 (47.7,60.7)	19 (15.6,23.2)	-64.7 (-71.9,-55.2)
Hungary	1.1 (1,1.2)	0.6 (0.4,0.8)	-46.5 (-60.2,-27.5)	1 (0.9,1.1)	0.4 (0.3,0.5)	-59.5 (-69.7,-47.8)	55.5 (48.3,63.8)	18.6 (14.5,24)	-66.5 (-75.4,-56.1)
Montenegro	1.1 (0.6,1.7)	0.6 (0.3,0.9)	-43.9 (-65.9,-9.9)	0.8 (0.5,1.1)	0.4 (0.3,0.7)	-43.4 (-62.5,-19.3)	45.5 (28.4,66.5)	20.2 (11.5,30.4)	-55.6 (-70.9,-32.5)
North Macedonia	1.2 (0.7,1.7)	0.6 (0.3,0.9)	-52.6 (-71.1,-28.9)	1.1 (0.7,1.6)	0.5 (0.3,0.8)	-52.6 (-69.9,-29)	64.5 (42.2,88.8)	21.7 (11.5,33)	-66.4 (-81.2,-47.9)
Poland	0.9 (0.7,1)	0.5 (0.4,0.7)	-37.9 (-52.4,-15.5)	0.8 (0.6,0.9)	0.4 (0.3,0.4)	-55.2 (-62.8,-43.3)	47 (37.9,52.7)	17.5 (15.7,19.3)	-62.9 (-69.1,-50.8)
Romania	1.1 (0.9,1.2)	0.7 (0.6,0.9)	-35.3 (-51,-15.6)	1 (0.9,1.1)	0.5 (0.4,0.6)	-45.8 (-56.6,-32.5)	64.4 (56.7,72.6)	25.8 (21.3,30.7)	-59.9 (-68.7,-49.9)
Serbia	0.9 (0.5,1.4)	0.4 (0.2,0.7)	-54.1 (-74.4,-24.5)	0.9 (0.4,1.3)	0.3 (0.1,0.5)	-60.3 (-75.6,-40.7)	49.9 (27.1,73.9)	14.2 (6.5,20.8)	-71.5 (-84.4,-52.1)
Slovakia	0.9 (0.5,1.2)	0.5 (0.2,0.8)	-39.2 (-64.7,-5.8)	0.8 (0.4,1.1)	0.4 (0.2,0.6)	-54.1 (-72.3,-28)	43.2 (24.6,57.7)	18.6 (9.5,28.1)	-57 (-73.8,-34.9)
Slovenia	1.1 (1,1.3)	1 (0.7,1.3)	-14.1 (-39.5,18.1)	1 (0.8,1.1)	0.4 (0.3,0.5)	-55.8 (-66.1,-43.2)	48.7 (42.5,55.4)	19.6 (15.2,24.3)	-59.8 (-69.1,-47.6)
Central Asia	1.3 (1.2,1.5)	0.7 (0.6,0.8)	-47.7 (-57.7,-31.4)	1.2 (1.1,1.4)	0.6 (0.5,0.7)	-51.9 (-60.7,-37.2)	80 (70.8,91.6)	36.1 (30.1,44.5)	-54.9 (-63.9,-40.8)
Armenia	2.2 (1.9,2.6)	0.7 (0.6,0.9)	-67 (-74.2,-57.7)	2.1 (1.8,2.3)	0.6 (0.5,0.6)	-73 (-78,-66.4)	121.7 (106.5,137)	27.7 (23.9,31.6)	-77.2 (-80.9,-72.1)
Azerbaijan	1.5 (1,1.9)	0.9 (0.6,1.3)	-39.5 (-59.3,-11.6)	1.4 (0.9,1.8)	0.7 (0.5,1.1)	-46.6 (-64.1,-22.4)	95.2 (61.4,121.7)	48.4 (31.1,69.4)	-49.1 (-65.5,-26.3)
Georgia	1.8 (1.6,2)	0.5 (0.5,0.6)	-69.3 (-74.6,-62.6)	1.5 (1.4,1.7)	0.5 (0.4,0.5)	-69.2 (-73.7,-63.1)	104 (92.9,116.5)	23.7 (20.9,27.3)	-77.2 (-80.6,-72.4)

Kazakhstan	1.1 (1,1.3)	0.5 (0.5,0.7)	-49.8 (-60.5,-34.5)	1 (0.9,1.2)	0.4 (0.4,0.5)	-56.2 (-64.9,-42.9)	64.2 (56.1,74.7)	23.3 (20,27.2)	-63.7 (-70.6,-54.1)
Kyrgyzstan	1.2 (1.1,1.4)	0.5 (0.4,0.6)	-62.4 (-69.6,-53.4)	1.2 (1,1.3)	0.4 (0.3,0.5)	-66.7 (-72.9,-59.1)	78.7 (69.4,88.8)	23.2 (19.3,27.7)	-70.5 (-76.5,-63.2)
Mongolia	1.3 (0.7,2.1)	0.6 (0.3,0.8)	-55.6 (-78.1,-26.7)	1.3 (0.7,2)	0.5 (0.3,0.8)	-58 (-79.4,-29.6)	80.4 (45.6,136.5)	29.9 (16.1,44.1)	-62.8 (-82.4,-37.6)
Tajikistan	1.2 (0.7,1.8)	0.7 (0.3,1.2)	-47 (-71.5,-5.5)	1.2 (0.7,1.7)	0.6 (0.3,1.1)	-48.9 (-72.7,-8.7)	80.6 (46.6,116)	39.7 (19.8,74.2)	-50.8 (-74.5,-5.5)
Turkmenistan	1.1 (0.9,1.3)	0.7 (0.5,0.9)	-36.8 (-51.8,-14.1)	1 (0.8,1.3)	0.6 (0.5,0.8)	-40.7 (-54.9,-18.3)	69.7 (55.4,86.2)	38.1 (29.9,49.3)	-45.3 (-57.9,-25.9)
Uzbekistan	1.3 (1,1.6)	0.8 (0.6,1)	-40.4 (-57,-14.3)	1.2 (0.9,1.5)	0.7 (0.5,0.8)	-45.6 (-60.6,-21.5)	77.6 (61.2,95)	42.6 (33.8,53.9)	-45.2 (-60.1,-21.2)
Central Latin America	1.9 (1.8,2)	2 (1.8,2.3)	7.5 (-7.4,23.9)	1.8 (1.7,1.9)	1.7 (1.5,1.9)	-6.6 (-18.5,7)	111.7 (106,117.8)	90.4 (80,103.3)	-19.1 (-29.7,-6.5)
Colombia	1.8 (1.6,2)	1.9 (1.6,2.4)	7.7 (-15.4,38.8)	1.7 (1.5,1.8)	1.4 (1.2,1.7)	-17.2 (-31.9,0.3)	104 (93.8,115.4)	75.4 (61.9,90.9)	-27.4 (-41.2,-10.9)
Costa Rica	1.8 (1.5,2)	2.2 (1.9,2.7)	26.8 (0.9,60.3)	1.5 (1.3,1.6)	1.5 (1.3,1.7)	3.4 (-12.4,24.1)	80.5 (72.1,88.2)	76.3 (66.8,85.8)	-5.2 (-19.3,12.2)
El Salvador	2.1 (1.4,2.8)	1.9 (0.9,2.6)	-9.2 (-48.2,32.2)	1.9 (1.4,2.6)	1.5 (0.8,2)	-21.3 (-54.1,10.9)	127.5 (92.8,169.8)	85.7 (45.8,114.3)	-32.8 (-60.5,-1.5)
Guatemala	1.5 (1.2,1.7)	1.9 (1.6,2.2)	27.3 (2.3,59.9)	1.4 (1.2,1.7)	1.7 (1.5,2)	20 (-2.7,49.4)	93.4 (79.7,108.1)	96.6 (81.8,113.3)	3.3 (-16.3,27.1)
Honduras	2.6 (1.8,3.6)	1.8 (1,2.8)	-29.9 (-58.8,7.6)	2.6 (1.8,3.5)	1.8 (0.9,2.8)	-29.2 (-57.2,7)	162.5 (109.3,224.4)	84.4 (45.5,137.4)	-48.1 (-70.8,-13.4)
Mexico	2 (1.9,2.1)	2.2 (1.9,2.5)	10.3 (-3.9,25.8)	1.9 (1.8,2)	1.9 (1.6,2.1)	-2.6 (-15.1,11)	118.4 (112.3,125.9)	100.1 (87.3,111.8)	-15.5 (-26,-3.7)
Nicaragua	2 (1.5,2.8)	1.4 (0.7,1.9)	-30.9 (-62.9,3.2)	1.8 (1.4,2.5)	1.2 (0.6,1.5)	-35.9 (-64.4,-5.9)	123.5 (89.2,165.7)	63.2 (35.2,83.6)	-48.8 (-72.8,-22.9)
Panama	1.6 (1.5,1.9)	2.2 (1.7,2.8)	33.7 (3.6,75.5)	1.4 (1.3,1.6)	1.5 (1.3,1.8)	4.9 (-13.3,30.1)	90.6 (81.4,99.4)	82.8 (69.8,98.1)	-8.5 (-23.9,12.7)
Venezuela (Bolivarian Republic of)	1.5 (1.4,1.6)	1.8 (1.3,2.5)	21 (-14.5,66.4)	1.4 (1.3,1.5)	1.4 (1,1.9)	2.5 (-25.5,38.6)	88.4 (80.8,94.9)	82.9 (59.8,110.6)	-6.3 (-32.7,27.3)
Andean Latin America	2 (1.5,2.8)	2.2 (1.3,2.8)	6.9 (-37,54.6)	2 (1.5,2.7)	1.8 (1.1,2.2)	-11.2 (-45.2,21.6)	120.8 (90.5,170.1)	92.8 (58.6,118.3)	-23.2 (-55.5,11.6)

Bolivia (Plurinational State of)	3.3 (1.7,5.4)	2.4 (1.3,3.5)	-28.4 (-63.8,31.5)	3.3 (1.8,5.4)	2.3 (1.2,3.4)	-31.4 (-64,20.2)	202.2 (101.5,325.5)	119.5 (66.5,173.3)	-40.9 (-71.5,17.8)
Ecuador	1.7 (1.5,1.8)	2.4 (1.9,2.9)	45 (13.9,79.1)	1.6 (1.5,1.8)	2.1 (1.7,2.6)	28.6 (2.4,60.5)	92.8 (84.3,101.9)	104.9 (86.1,126.5)	13 (-9.7,37.4)
Peru	1.8 (1.3,2.6)	2 (0.8,2.9)	10.9 (-55.3,84.6)	1.7 (1.3,2.5)	1.4 (0.6,2)	-17.8 (-65.3,25.8)	108.4 (79.8,161)	77.8 (33.9,111.9)	-28.2 (-71,14.1)
Caribbean	1.6 (1,2.4)	1.3 (0.9,2.1)	-14.7 (-35.7,10.7)	1.4 (0.9,2.3)	1.2 (0.8,1.9)	-16.2 (-37.3,10.3)	97.7 (58.3,161.7)	78.2 (44.7,133.7)	-19.9 (-41.3,8.8)
Antigua and Barbuda	0.9 (0.8,1.1)	1 (0.8,1.2)	3.9 (-18.6,32.7)	0.8 (0.7,0.9)	0.7 (0.6,0.7)	-20.1 (-31.7,-7.3)	48.2 (42.5,53.8)	34.9 (31.4,38.6)	-27.7 (-38.6,-16.1)
Barbados	0.6 (0.5,0.8)	0.4 (0.3,0.6)	-33.6 (-53.5,-3.2)	0.5 (0.4,0.6)	0.3 (0.2,0.4)	-45 (-58,-27.8)	34.3 (28.7,40.7)	16 (12.3,20.6)	-53.4 (-65.2,-36.2)
Belize	1.2 (1,1.4)	0.8 (0.7,0.9)	-33.3 (-44.9,-19.3)	1 (0.9,1.2)	0.7 (0.6,0.8)	-32.7 (-43.2,-19.7)	72 (63.6,81.2)	36.9 (32.1,42.3)	-48.8 (-57,-39)
Bermuda	0.9 (0.6,1.3)	1.7 (1,3)	87 (-4.1,284.3)	0.7 (0.5,0.9)	0.3 (0.2,0.4)	-57.1 (-70.7,-32.5)	33.8 (23.2,45.8)	17.1 (12.7,22.7)	-49.4 (-66.2,-17.9)
Bahamas	0.4 (0.4,0.5)	0.3 (0.2,0.4)	-29.7 (-48.6,-2.9)	0.4 (0.3,0.4)	0.3 (0.2,0.3)	-33.2 (-50.7,-9.1)	25.5 (21.5,29.3)	15.5 (11.8,20.2)	-39.3 (-56,-15.2)
Cuba	1.3 (1.1,1.5)	1 (0.8,1.3)	-22.8 (-45.4,7.8)	1 (0.9,1.1)	0.6 (0.5,0.7)	-40.3 (-53,-25.7)	58.6 (52,66.8)	30.8 (25.3,37.5)	-47.4 (-59.1,-33.9)
Dominica	1.1 (0.6,1.5)	1.5 (0.8,2.3)	35 (-5.8,97.1)	1.1 (0.6,1.5)	1.3 (0.7,2)	22.6 (-12.6,77.3)	59.2 (34.3,78.6)	76 (44.6,113)	28.3 (-12.2,87.7)
Dominican Republic	1 (0.6,1.5)	0.6 (0.3,1)	-39.1 (-67.2,4.7)	0.9 (0.6,1.4)	0.5 (0.3,0.9)	-43 (-66.5,-10.1)	68.9 (43.6,101)	33.3 (18.6,56.2)	-51.7 (-73.8,-14.1)
Grenada	1 (0.8,1.3)	0.8 (0.7,1)	-19.4 (-36.7,8.7)	1 (0.8,1.2)	0.7 (0.6,0.8)	-27.4 (-42.7,-4.1)	68.3 (54.6,81.7)	42.8 (36.4,49.7)	-37.4 (-50.2,-17)
Guyana	1.1 (0.9,1.3)	0.9 (0.7,1.3)	-17.6 (-40.8,10.9)	1.1 (0.9,1.3)	0.9 (0.7,1.2)	-20.6 (-42.8,6.1)	69.1 (55,82.3)	52.3 (39.7,69.8)	-24.3 (-45.6,2.9)
Haiti	3.3 (1.1,6.8)	2.5 (1.1,4.8)	-24.9 (-51.9,31.7)	3.3 (1.1,6.7)	2.4 (1.1,4.7)	-25.4 (-51.7,30.9)	220.6 (64.6,467)	156.2 (62,313.2)	-29.2 (-54.7,32.1)
Jamaica	1 (0.8,1.2)	0.7 (0.5,1)	-26 (-48.1,7)	0.7 (0.6,0.9)	0.5 (0.4,0.7)	-27.8 (-46.2,-1.9)	53.2 (44.6,63)	31.2 (24.2,39.8)	-41.3 (-56.7,-20.9)
Puerto Rico	0.9 (0.8,1.1)	1.1 (0.8,1.6)	21.6 (-14.4,74.8)	0.7 (0.6,0.8)	0.6 (0.5,0.7)	-14.8 (-34.6,8.2)	39.5 (33.9,45.5)	24.6 (19.9,30)	-37.8 (-52.3,-20.8)

Saint Kitts and Nevis	1.3 (1.1,1.5)	0.9 (0.7,1.2)	-27 (-46,-0.6)	1.2 (1,1.4)	0.7 (0.6,0.8)	-42.5 (-54.9,-27.4)	83.6 (70.5,97.7)	45.9 (37.5,56.1)	-45.1 (-57.1,-30.6)
Saint Lucia	0.7 (0.6,0.9)	0.5 (0.4,0.6)	-34.7 (-51.6,-13.1)	0.7 (0.6,0.8)	0.4 (0.3,0.5)	-45.2 (-57.1,-28.1)	42.6 (35.9,49.8)	23.6 (18.9,29.8)	-44.6 (-57.7,-26.6)
Saint Vincent and the Grenadines	0.8 (0.7,0.9)	1 (0.8,1.2)	24.4 (0.8,57)	0.7 (0.6,0.8)	0.9 (0.7,1)	18.3 (-2.1,47)	51.3 (44,59.8)	57.3 (47.7,67.8)	11.6 (-9.6,40.6)
Suriname	0.8 (0.4,1)	0.7 (0.3,1)	-11.1 (-38.9,44)	0.7 (0.3,1)	0.6 (0.3,0.9)	-16.8 (-42.6,35.4)	50.2 (23.6,67.7)	40.6 (21.8,56.7)	-19.1 (-45,38.8)
Trinidad and Tobago	1.1 (1,1.3)	1 (0.7,1.3)	-13.9 (-36.9,17.3)	1.1 (0.9,1.2)	0.8 (0.6,1)	-24.3 (-43.3,1)	65.9 (56.6,74.5)	44.5 (34.8,56.5)	-32.5 (-49.8,-8.3)
United States Virgin Islands	0.6 (0.3,0.9)	0.3 (0.1,0.6)	-45.4 (-78.5,2.2)	0.5 (0.3,0.8)	0.2 (0.1,0.3)	-63.5 (-84.7,-37.5)	31.8 (17.2,45.9)	10.5 (3.7,19)	-66.9 (-86.5,-41)
Tropical Latin America	0.9 (0.9,1)	0.9 (0.8,1)	-7.1 (-18.5,6.2)	0.9 (0.8,0.9)	0.7 (0.7,0.8)	-18.9 (-27.1,-10)	56.4 (50.9,62)	40.1 (36.5,44.3)	-29 (-38,-18.9)
Brazil	0.9 (0.9,1)	0.9 (0.8,1)	-7.7 (-18.9,4.6)	0.9 (0.8,0.9)	0.7 (0.6,0.8)	-19.4 (-27.5,-10.8)	55.6 (50,61.4)	39.2 (35.8,43.1)	-29.5 (-38.3,-19.6)
Paraguay	1.4 (0.9,2)	1.4 (0.7,2.2)	0.3 (-34.4,50.8)	1.3 (0.9,1.8)	1.2 (0.6,1.7)	-11.3 (-40.8,25.7)	83.9 (55.3,112.9)	64.2 (35.8,91.4)	-23.5 (-49.4,9.7)
East Asia	3.3 (2,4.6)	3 (1.3,4.3)	-9.7 (-62.5,55.7)	2.9 (1.8,4)	1.1 (0.5,1.4)	-63.5 (-82.3,-45.3)	188.1 (116.2,265.5)	57.6 (26.6,76.3)	-69.4 (-85.6,-52.6)
China	3.4 (2,4.7)	3 (1.3,4.4)	-9.9 (-63.1,55.9)	3 (1.8,4.1)	1.1 (0.5,1.4)	-64.3 (-82.8,-45.7)	192.2 (118.6,271.4)	57.7 (26.3,77.2)	-70 (-86.1,-53)
Democratic People's Republic of Korea	2.2 (1.1,3.3)	2 (0.8,3)	-10.2 (-44.3,45.9)	2 (0.9,3.1)	1.6 (0.7,2.5)	-18.7 (-48.6,28.2)	115 (56.6,181.4)	84.8 (35.6,129.1)	-26.3 (-55.1,22.6)
Taiwan (Province of China)	0.7 (0.5,1)	1.2 (0.9,1.6)	68.2 (3.9,206.8)	0.5 (0.3,0.6)	0.4 (0.3,0.4)	-20.9 (-44.7,22.9)	27.3 (18.9,35.7)	19.8 (17,23)	-27.6 (-48.2,13)
Southeast Asia	1.4 (0.6,2.2)	1 (0.4,1.2)	-31.7 (-57.7,6.2)	1.4 (0.6,2.2)	0.8 (0.3,1.1)	-38.9 (-63.1,-7.3)	80.1 (33.5,130.1)	46.1 (17.8,60.2)	-42.4 (-68.5,-2.1)
Cambodia	1.8 (0.5,3.7)	1.1 (0.3,1.8)	-40.1 (-71.6,39.9)	1.8 (0.5,3.8)	1 (0.2,1.8)	-43.2 (-73.9,28.9)	109.4 (30,227.3)	56.2 (14.9,94)	-48.6 (-77,41.9)
Indonesia	1.3 (0.4,2.2)	0.9 (0.3,1.3)	-28.7 (-63.9,29)	1.3 (0.4,2.2)	0.9 (0.2,1.3)	-32 (-67.3,19.2)	77.4 (26,134.3)	45.9 (13.9,65)	-40.7 (-72.3,16.9)

Lao People's Democratic Republic	1.9 (0.5,4.2)	1.1 (0.3,1.8)	-43 (-70.5,47.8)	1.9 (0.5,4.3)	1.1 (0.3,1.7)	-45.2 (-72.3,40.9)	116.4 (30.4,251.8)	60.8 (17.8,102)	-47.8 (-74.6,63.1)
Malaysia	1.4 (0.6,2)	1 (0.4,1.3)	-28.9 (-53.2,4.7)	1.3 (0.6,1.9)	0.8 (0.4,1.1)	-36.7 (-57.7,-10.5)	73 (32.3,109.1)	40.4 (17.4,53.7)	-44.7 (-64.2,-14.9)
Maldives	0.9 (0.3,1.9)	0.4 (0.2,0.7)	-52.4 (-81.8,88.1)	0.9 (0.3,1.9)	0.2 (0.1,0.3)	-73.5 (-89.3,-6.9)	57.7 (17.9,119.8)	14.1 (7.3,21)	-75.6 (-90.1,-3.1)
Mauritius	0.6 (0.6,0.7)	0.6 (0.5,0.7)	-8.2 (-24,13.6)	0.6 (0.5,0.6)	0.4 (0.4,0.5)	-22.8 (-33,-10.9)	34.1 (30.5,37.7)	22.7 (20.4,25)	-33.3 (-42.9,-21.6)
Myanmar	2.2 (0.6,4.8)	1.2 (0.3,1.8)	-47.9 (-72.4,18.6)	2.2 (0.6,4.7)	1.1 (0.3,1.6)	-52.1 (-75.8,10.4)	138.9 (38.3,309.2)	63.7 (19.9,98.6)	-54.1 (-77.7,24)
Philippines	1.4 (0.8,2)	1 (0.5,1.4)	-25.8 (-51.6,7.8)	1.4 (0.7,1.9)	1 (0.5,1.3)	-29.6 (-53.7,-0.5)	83.5 (42.6,121.1)	55.2 (26.5,74.6)	-33.9 (-60,2.9)
Sri Lanka	0.8 (0.5,1.5)	0.6 (0.3,1)	-23.7 (-70.7,68.6)	0.8 (0.4,1.5)	0.4 (0.2,0.7)	-41.4 (-77.4,18.5)	43.1 (25.1,84)	22.1 (9.1,33.9)	-48.9 (-81.6,13)
Seychelles	0 (0,0)	0 (0,0)	-20.3 (-49.1,32.7)	0 (0,0)	0 (0,0)	-20.4 (-49.7,22.3)	0 (0,0)	0 (0,0)	-24.8 (-51.8,23.4)
Thailand	1.4 (0.7,2.3)	1.1 (0.5,1.6)	-24.4 (-57.8,27.3)	1.4 (0.6,2.1)	0.7 (0.3,1)	-52.1 (-73.2,-19)	71.6 (35.4,112.8)	34.6 (15.4,49.6)	-51.7 (-73.7,-24.5)
Timor-Leste	1.6 (0.4,3.2)	1 (0.3,1.6)	-35.5 (-69.2,46.9)	1.6 (0.4,3.2)	1 (0.3,1.6)	-37.8 (-71.9,36.3)	99.4 (27.9,207.1)	55.6 (16.9,89.3)	-44 (-75.1,50.9)
Viet Nam	1.2 (0.6,1.7)	0.9 (0.5,1.3)	-26 (-55.1,13.5)	1.2 (0.6,1.7)	0.7 (0.3,1)	-42.6 (-64.4,-15.2)	57.3 (34,82.5)	33.1 (16.3,46.8)	-42.3 (-64.9,-9.7)
Oceania	0.7 (0.3,1.2)	0.6 (0.3,1.1)	-4.8 (-30.4,32.6)	0.7 (0.4,1.2)	0.6 (0.3,1.1)	-7.2 (-30.7,28.2)	41.7 (18.1,78.6)	39.9 (18.5,72.6)	-4.3 (-33.1,42)
American Samoa	0.3 (0.2,0.7)	0.4 (0.2,0.6)	21.2 (-37.7,124.3)	0.3 (0.2,0.6)	0.4 (0.2,0.6)	18.1 (-32,99.5)	14.3 (8.7,32)	15.6 (7.6,26.8)	9.1 (-48.3,114.9)
Cook Islands	0.3 (0.1,0.4)	0.2 (0.1,0.4)	-19.7 (-70.7,97.4)	0.3 (0.1,0.4)	0.1 (0.1,0.2)	-49.6 (-73.8,-6.3)	11.9 (6.4,19.7)	5.3 (2,9.9)	-55.7 (-82.6,3.1)
Micronesia (Federated States of)	0.8 (0.4,1.3)	0.6 (0.3,0.9)	-20.7 (-50.6,28.9)	0.8 (0.5,1.4)	0.7 (0.3,1)	-20.1 (-49.2,26.1)	44.7 (23.3,74.9)	30.7 (15.2,47.6)	-31.2 (-59.5,26)
Fiji	0.4 (0.1,0.6)	0.3 (0.1,0.4)	-29.2 (-56.1,29.1)	0.4 (0.1,0.6)	0.3 (0.1,0.4)	-27.8 (-54.8,29.5)	22.3 (5.3,34.8)	12.7 (4.4,21.2)	-42.8 (-66.5,12.7)
Guam	0.5 (0.3,0.8)	0.3 (0.2,0.5)	-37.1 (-69.7,12.5)	0.5 (0.3,0.8)	0.2 (0.1,0.3)	-62.8 (-76.6,-44.6)	19.3 (12,33.8)	8.7 (4.7,13.8)	-54.8 (-73,-30.1)

Kiribati	0.7 (0.4,1.1)	0.6 (0.4,0.9)	-9.5 (-42.1,69.2)	0.7 (0.4,1.1)	0.7 (0.4,1)	-7.7 (-39.8,70.1)	45.6 (27,75.8)	37.2 (20.7,57.1)	-18.5 (-51.5,64.9)
Marshall Islands	0.6 (0.3,0.9)	0.7 (0.3,1)	13.4 (-25.3,65.1)	0.6 (0.4,0.9)	0.7 (0.4,1)	11.2 (-24.1,60.5)	30.3 (17,46.3)	34.2 (17.2,53.2)	12.8 (-26.1,72.1)
Nauru	0.9 (0.5,1.4)	0.9 (0.4,1.4)	-3.1 (-33.8,41)	0.9 (0.5,1.5)	0.9 (0.4,1.4)	-5.9 (-37.1,34.2)	48.9 (26.6,80.5)	45.5 (21.3,75.4)	-6.9 (-38.7,42.5)
Niue	0.6 (0.3,0.9)	2 (1,3)	246.8 (128.1,457.6)	0.6 (0.3,0.9)	1.5 (0.8,2.2)	155.4 (68.7,283.4)	29.9 (17.7,46.9)	103.1 (54.8,156.5)	244.7 (123.8,437.7)
Northern Mariana Islands	1.7 (0.8,2.3)	1.1 (0.6,1.4)	-33.4 (-53.8,0.6)	1.6 (0.8,2.4)	1 (0.6,1.3)	-37.2 (-55.6,-3.2)	68 (29.9,98.6)	42.1 (24.4,53.8)	-38 (-58,-2)
Palau	0.8 (0.5,1.1)	0.8 (0.5,1.2)	-0.8 (-31.5,43.6)	0.8 (0.5,1.2)	0.8 (0.5,1.2)	-1.2 (-33.2,44.1)	34.7 (22.8,58.3)	34.3 (22,63.8)	-1 (-33.1,42.6)
Papua New Guinea	0.8 (0.3,1.5)	0.7 (0.3,1.3)	-6.2 (-36.9,40.7)	0.8 (0.3,1.4)	0.7 (0.3,1.2)	-7.5 (-36.8,39.3)	47.8 (18.8,98.1)	44.1 (18.8,84)	-7.9 (-39.8,51.9)
Samoa	0.7 (0.4,1.1)	0.7 (0.4,1)	1.2 (-34.8,48.7)	0.7 (0.4,1.2)	0.7 (0.4,1)	-0.4 (-32.9,45.8)	34.7 (22.7,60.2)	29.6 (15.8,41.9)	-14.8 (-45,31)
Solomon Islands	0.6 (0.3,1.1)	0.6 (0.3,1)	-0.6 (-32.4,45.1)	0.7 (0.3,1.1)	0.7 (0.3,1)	-0.5 (-30.8,43.5)	35.8 (17.3,61)	33.5 (16.3,50.9)	-6.5 (-39.3,41.5)
Tokelau	0.7 (0.4,1)	2.1 (0.8,4.4)	198.3 (27.1,567)	0.7 (0.4,1)	1.6 (0.7,2.9)	117.5 (5.5,321.5)	38.7 (19.7,58.5)	111.9 (45.8,226.2)	189.4 (21.2,520.4)
Tonga	0.2 (0.2,0.4)	0.2 (0.1,0.4)	2.9 (-35.3,68.8)	0.2 (0.2,0.4)	0.2 (0.1,0.3)	-1.6 (-36.3,57.1)	12.1 (7.3,19.2)	11 (5.5,16.7)	-8.8 (-46.7,61.7)
Tuvalu	0.8 (0.4,1.5)	0.6 (0.3,0.8)	-28.7 (-59.2,35.5)	0.8 (0.4,1.5)	0.6 (0.3,0.8)	-28.5 (-57.7,27)	46.3 (20.3,90.3)	27.7 (13.5,40.1)	-40.2 (-68.4,36.8)
Vanuatu	0.5 (0.3,0.8)	0.5 (0.3,0.7)	-0.1 (-33.3,48)	0.5 (0.3,0.9)	0.5 (0.3,0.8)	0.2 (-30.6,48.1)	27.3 (14.6,43.3)	26.3 (12.7,39.4)	-3.5 (-37.4,47.7)
North Africa and Middle East	1.7 (0.9,2.7)	1.2 (0.6,1.6)	-28.3 (-55.3,13.8)	1.6 (0.8,2.5)	1 (0.4,1.4)	-38.4 (-61.4,-10)	95.2 (50.7,149.3)	52.7 (25.1,71.2)	-44.7 (-65.9,-14.2)
Afghanistan	4.7 (1,11.7)	4.1 (1.3,9.2)	-13.9 (-38,63.5)	4.8 (1.1,11.7)	4.1 (1.3,9.3)	-14 (-38.5,53.1)	242.7 (50.6,567.2)	200.6 (67,433)	-17.4 (-41.8,66.1)
Algeria	0.4 (0.2,0.6)	0.2 (0.1,0.4)	-35.1 (-69.4,15.7)	0.4 (0.2,0.6)	0.2 (0.1,0.3)	-52.4 (-76.8,-17.5)	24.3 (12.7,39)	9.9 (4.6,15.7)	-59.1 (-80.9,-23)
Bahrain	0.6 (0.3,1)	0.4 (0.2,0.7)	-30.8 (-60.5,14.2)	0.6 (0.2,0.9)	0.3 (0.1,0.4)	-54.2 (-73.2,-26.7)	30.2 (14.3,46.3)	13.4 (6.4,19.4)	-55.6 (-74.4,-29.3)
Egypt	1.4 (0.8,2.3)	1.6 (0.7,2.3)	15.9 (-57.4,91.5)	1.4 (0.8,2.3)	1.5 (0.5,2.3)	8.8 (-60.5,75.1)	77.6 (48.1,141.1)	65.3 (26.5,93)	-15.8 (-73.6,46)

Iran (Islamic Republic of)	2.2 (1,3.2)	1.3 (0.6,1.9)	-41.7 (-67.2,0.6)	2 (0.9,2.7)	0.8 (0.4,1.3)	-57.1 (-73.2,-38)	123.2 (56.3,172.1)	44.4 (22.1,62.5)	-63.9 (-77.9,-43.5)
Iraq	1.3 (0.6,2.4)	1 (0.5,1.6)	-23.4 (-61.2,103.7)	1.2 (0.6,2.2)	0.7 (0.4,1.1)	-43.2 (-69.5,37.2)	78.8 (37.4,132.7)	39.1 (19.8,59.8)	-50.4 (-74.8,24.5)
Jordan	1.2 (0.6,1.7)	0.8 (0.3,1.3)	-36.4 (-70.4,15.5)	1.1 (0.5,1.6)	0.4 (0.2,0.6)	-64.9 (-82.4,-43.2)	63 (31.9,90.8)	21.6 (9.7,32.4)	-65.6 (-82.5,-44.1)
Kuwait	1.4 (1.1,1.8)	1.3 (0.8,1.9)	-10.3 (-44.4,40.2)	0.9 (0.8,1.1)	0.4 (0.3,0.4)	-60.9 (-70.1,-49.5)	48.4 (41.3,55.9)	19.6 (15.5,23.5)	-59.5 (-69.1,-48)
Lebanon	1.2 (0.6,2)	0.9 (0.4,1.5)	-27.1 (-69.6,65.5)	1.1 (0.6,1.9)	0.5 (0.2,0.8)	-54.8 (-78.9,-8.5)	54.2 (30.4,93.2)	22.9 (11.5,35.4)	-57.8 (-80.6,-9.2)
Libya	2 (1,2.9)	2.7 (1.1,4.1)	30.3 (-18.2,113.3)	1.9 (0.8,2.6)	2.1 (0.7,3.2)	9.5 (-30.5,83.7)	99.7 (50.6,139.3)	100.6 (41.1,146.7)	0.8 (-33.1,61.3)
Morocco	0.3 (0.1,0.5)	0.2 (0.1,0.3)	-30.6 (-62.2,20.8)	0.3 (0.1,0.5)	0.2 (0.1,0.3)	-35.1 (-65.3,6.9)	17.9 (9.3,29.4)	9.5 (3.5,14.8)	-46.7 (-73.6,7)
Palestine	1.5 (0.9,2.4)	1.1 (0.6,1.5)	-29.8 (-57.2,24)	1.4 (0.8,2.1)	0.7 (0.4,1)	-45.5 (-65.4,-10.8)	84.7 (51.1,131.8)	41.5 (26.6,54.9)	-51 (-69.4,-15.2)
Oman	0.6 (0.3,1)	0.4 (0.2,0.7)	-25.8 (-62.9,35.7)	0.5 (0.2,0.8)	0.2 (0.1,0.4)	-55.1 (-74.8,-21.2)	30.1 (14.7,48.2)	12.2 (5.8,18.1)	-59.6 (-78.2,-27.5)
Qatar	0.6 (0.2,0.8)	0.3 (0.2,0.6)	-39.7 (-69.3,10)	0.5 (0.2,0.8)	0.2 (0.1,0.3)	-68.3 (-82.2,-46.6)	25.1 (11.3,36.6)	7.3 (2.9,11.3)	-71 (-84.4,-49.9)
Saudi Arabia	0.8 (0.5,1.6)	0.8 (0.4,1.2)	-6.3 (-50.9,66.3)	0.8 (0.5,1.5)	0.6 (0.3,1)	-22.9 (-56.6,27.6)	45.5 (28.6,83.1)	27.8 (16.9,43.7)	-39 (-66.8,5.1)
Sudan	2.6 (0.7,5.7)	1.7 (0.6,3)	-31.9 (-65,106.6)	2.5 (0.7,5.6)	1.6 (0.5,2.8)	-36.4 (-67.6,83.7)	152.8 (41.5,341.3)	87.8 (33.7,145.7)	-42.5 (-71.5,85.9)
Syrian Arab Republic	2.5 (1.1,3.9)	1.6 (0.7,2.4)	-35.3 (-58.1,5.2)	2.4 (1,3.8)	1.3 (0.5,1.8)	-47.6 (-67,-18.8)	131.3 (63.8,206.1)	58.2 (27.4,82.5)	-55.7 (-72.1,-29.3)
Tunisia	1.1 (0.5,1.6)	0.7 (0.2,1.2)	-35.5 (-69.8,19)	1 (0.4,1.3)	0.5 (0.1,0.8)	-49.4 (-75.6,-16.7)	57 (26.3,80.8)	23.9 (7.7,38)	-58 (-80.4,-22)
Turkey	2.2 (1.1,3.6)	1.2 (0.6,1.9)	-43 (-72,27.6)	2 (1,3.3)	0.7 (0.3,0.9)	-66 (-81.9,-35.6)	125.9 (61.8,211.3)	36.3 (16.8,50.2)	-71.2 (-85.2,-38.4)
United Arab Emirates	1.1 (0.5,1.9)	0.7 (0.3,1.1)	-35.6 (-66.4,20.5)	1 (0.5,1.8)	0.6 (0.3,1.1)	-38.4 (-67.9,21.7)	53.7 (28.3,90.8)	24.1 (12.6,38.1)	-55.1 (-76.7,-20.8)
Yemen	1.9 (0.6,4.5)	1.5 (0.5,2.7)	-23.2 (-56.7,69.2)	1.9 (0.6,4.4)	1.4 (0.4,2.6)	-25 (-58.4,63)	106 (34,235.6)	72.8 (24.4,130.5)	-31.4 (-63.7,64.7)
South Asia	0.8 (0.4,1.2)	0.5 (0.3,0.7)	-36.7 (-59.8,-0.2)	0.8 (0.4,1.2)	0.5 (0.2,0.7)	-39.8 (-61.1,-8.5)	47.1 (24.7,81.2)	25.9 (15.3,36.6)	-44.9 (-66.1,-6.1)

Bangladesh	0.9 (0.4,1.8)	0.5 (0.3,0.8)	-42.1 (-72.5,48)	0.9 (0.4,1.8)	0.5 (0.3,0.7)	-47.7 (-74.7,29.7)	60.5 (22.8,125.3)	29.9 (18,42.5)	-50.6 (-76.7,36.2)
Bhutan	0.7 (0.3,1.3)	0.5 (0.3,0.8)	-31.4 (-72.4,71.5)	0.7 (0.3,1.3)	0.5 (0.2,0.8)	-36.8 (-74.1,54.3)	43.6 (16.1,84.3)	25.6 (13.4,42.7)	-41.3 (-78.5,61)
India	0.7 (0.4,1.2)	0.4 (0.2,0.6)	-43.9 (-66.2,-7.4)	0.7 (0.4,1.2)	0.4 (0.2,0.5)	-46.6 (-67.1,-16.1)	44.7 (24,76)	20.7 (11.4,27.1)	-53.7 (-73.5,-17.1)
Nepal	0.8 (0.3,1.6)	0.4 (0.2,0.7)	-45.8 (-74.8,22.3)	0.8 (0.3,1.6)	0.4 (0.2,0.6)	-47.4 (-75.2,12.9)	50.7 (19,107)	22.7 (12.5,34.3)	-55.2 (-80.3,18.6)
Pakistan	0.8 (0.4,1.3)	0.8 (0.4,1.4)	2.9 (-28.1,47)	0.8 (0.4,1.3)	0.8 (0.4,1.4)	0.2 (-30.8,42.5)	48.9 (23.1,83.7)	48.8 (26.4,81.3)	-0.3 (-33.5,49.5)
Southern Sub-Saharan Africa	0.5 (0.3,0.8)	0.6 (0.3,0.9)	21.5 (-25.1,63.9)	0.5 (0.3,0.8)	0.6 (0.3,0.8)	20.9 (-26,62.9)	29.1 (20.2,47.9)	32.3 (17.9,45.7)	11 (-30.1,52.4)
Botswana	0.4 (0.2,0.7)	0.4 (0.2,0.7)	8.3 (-39.7,92.5)	0.4 (0.2,0.7)	0.4 (0.2,0.6)	2.7 (-43.9,82.9)	22.8 (13.6,38)	24.3 (14,35.8)	6.7 (-39,84.2)
Lesotho	0.3 (0.2,0.6)	0.7 (0.4,1.2)	113.1 (14.2,316.3)	0.3 (0.2,0.6)	0.7 (0.4,1.2)	113.1 (13.8,320.4)	19 (11.6,32.1)	37.3 (21.3,57.1)	96.6 (13.3,252.9)
Namibia	0.4 (0.2,0.6)	0.4 (0.2,0.6)	3.3 (-41.7,73.4)	0.4 (0.2,0.6)	0.4 (0.2,0.6)	-3.5 (-45,62.7)	21.6 (13.3,33.3)	20.7 (10.6,33.8)	-4.2 (-46.9,68)
South Africa	0.5 (0.4,0.9)	0.5 (0.3,0.8)	-2.7 (-44.8,40.9)	0.5 (0.4,0.9)	0.5 (0.3,0.8)	-4.5 (-47.3,37)	31.8 (21,55.5)	26.3 (15,40)	-17.5 (-51.8,21.3)
Eswatini	0.5 (0.3,0.9)	0.6 (0.3,1)	26.3 (-29.6,107)	0.5 (0.3,0.9)	0.6 (0.3,1)	25.4 (-30.1,102.9)	28.4 (17.7,52.4)	33.1 (18.4,51.7)	16.7 (-35.2,98.2)
Zimbabwe	0.4 (0.3,0.7)	1 (0.5,1.4)	123.1 (31.9,272.7)	0.4 (0.2,0.7)	0.9 (0.5,1.4)	123.1 (30.5,282.4)	23.6 (14.3,34.4)	53.2 (28.1,78.1)	125.4 (39.4,271.3)
Western Sub-Saharan Africa	0.3 (0.2,0.5)	0.3 (0.1,0.4)	-15.7 (-50,25.2)	0.3 (0.2,0.5)	0.3 (0.1,0.4)	-17.8 (-51.7,21.2)	22.8 (12.3,33.2)	18.3 (7.1,25.7)	-19.9 (-53.4,23.2)
Benin	0.4 (0.2,0.5)	0.4 (0.1,0.6)	3 (-52.4,68.5)	0.4 (0.2,0.5)	0.4 (0.1,0.6)	0.3 (-54.8,65.6)	25.8 (14.9,39)	25 (8.3,39.1)	-3.1 (-56.6,68.1)
Burkina Faso	0.4 (0.2,0.6)	0.4 (0.1,0.6)	11.3 (-39.9,72)	0.4 (0.2,0.6)	0.4 (0.1,0.6)	9.8 (-41.7,70)	25.1 (13.8,40.5)	27.2 (10.1,41.6)	8.4 (-43.1,78)
Cameroon	0.4 (0.2,0.6)	0.4 (0.1,0.6)	1.1 (-51.4,57.2)	0.4 (0.2,0.5)	0.4 (0.1,0.6)	-0.8 (-54.4,56.2)	25.9 (14.5,38.4)	25 (7.9,38.2)	-3.2 (-55.5,59.4)
Cabo Verde	0.6 (0.4,0.9)	0.5 (0.2,0.8)	-18 (-73,34.9)	0.6 (0.3,0.8)	0.4 (0.1,0.7)	-25 (-75.1,23.3)	39 (24.2,57.6)	26.9 (9.6,43.9)	-31 (-77.7,17.9)
Chad	0.3 (0.2,0.5)	0.4 (0.2,0.7)	37.6 (-20.8,105.9)	0.3 (0.2,0.5)	0.4 (0.2,0.7)	37.2 (-24.2,106.7)	20.6 (11.8,34)	27.5 (11.4,44.4)	33.4 (-26.2,114.3)

CÃ´te d'Ivoire	0.2 (0.1,0.3)	0.2 (0.1,0.3)	-14.2 (-50,41.1)	0.2 (0.1,0.3)	0.2 (0.1,0.3)	-4.7 (-42.5,60.5)	14.6 (7.3,21)	13.7 (5.6,23.4)	-5.9 (-45,66.4)
Gambia	0.2 (0.1,0.3)	0.2 (0.1,0.4)	-1.6 (-40.2,66)	0.2 (0.1,0.3)	0.1 (0.1,0.3)	-16.1 (-50.1,34.9)	12.5 (5.7,18.4)	9.7 (4.3,17.9)	-22.2 (-57,36.4)
Ghana	0.7 (0.2,0.9)	0.3 (0.1,0.4)	-60.7 (-81.6,-18)	0.6 (0.2,0.9)	0.2 (0.1,0.4)	-61.8 (-82.5,-17.2)	41.6 (14.4,60.2)	15.6 (7.6,27.7)	-62.4 (-83,-21.9)
Guinea	0.2 (0.1,0.3)	0.1 (0,0.3)	-25.3 (-61.2,45.8)	0.2 (0.1,0.3)	0.1 (0,0.2)	-26.1 (-61.9,44)	13.1 (6.5,20.2)	9.1 (3.2,17.4)	-30.5 (-66.1,43.3)
Guinea-Bissau	0.5 (0.3,0.8)	0.5 (0.2,0.7)	-6.9 (-50.3,55.8)	0.5 (0.3,0.8)	0.4 (0.2,0.7)	-7.4 (-51.3,57.7)	32 (17,52.9)	27.2 (10.6,41.8)	-14.8 (-56.3,54.9)
Liberia	0.4 (0.2,0.7)	0.4 (0.1,0.6)	0.7 (-51.8,91.8)	0.4 (0.2,0.7)	0.4 (0.1,0.6)	-1.9 (-54.1,88.6)	28.9 (14.3,49.6)	25.9 (9.1,39.3)	-10.1 (-58.5,90.1)
Mali	0.5 (0.2,0.7)	0.3 (0.1,0.6)	-31.3 (-61.1,19.1)	0.5 (0.2,0.7)	0.3 (0.1,0.5)	-32.3 (-62,16.3)	32.6 (15.1,49.9)	21 (8.9,36.2)	-35.7 (-65.2,20)
Mauritania	0.3 (0.2,0.4)	0.3 (0.1,0.5)	-7.3 (-60.1,51.2)	0.3 (0.2,0.4)	0.3 (0.1,0.5)	-13.3 (-63.3,42.6)	20.8 (12.6,30.2)	17.3 (6.5,29)	-17.2 (-64.7,39.3)
Niger	0.5 (0.2,0.8)	0.4 (0.1,0.6)	-20.5 (-62.3,57.9)	0.5 (0.2,0.8)	0.4 (0.1,0.6)	-21 (-62.9,58)	33 (15.8,60.7)	23.8 (8.7,37.9)	-27.8 (-66.1,56.5)
Nigeria	0.3 (0.1,0.4)	0.2 (0.1,0.4)	-12.2 (-54.3,68)	0.2 (0.1,0.4)	0.2 (0.1,0.3)	-15.9 (-55.6,58.9)	18.2 (10.5,29.4)	15.1 (6,23.7)	-17.1 (-56.1,62)
Sao Tome and Principe	0.4 (0.2,0.5)	0.2 (0.1,0.4)	-37.6 (-74.3,13.2)	0.3 (0.2,0.5)	0.2 (0.1,0.4)	-39.5 (-75,5.9)	24.3 (13,36)	12.8 (4.2,26.3)	-47.3 (-80,1.6)
Senegal	0.4 (0.2,0.5)	0.3 (0.1,0.6)	-10.1 (-57.6,48.4)	0.4 (0.2,0.5)	0.3 (0.1,0.6)	-11.6 (-58.8,46.3)	25.7 (13.9,37.7)	20.9 (7.3,36.2)	-18.6 (-61.8,42.5)
Sierra Leone	0.4 (0.2,0.6)	0.4 (0.1,0.6)	14.1 (-42.4,92.9)	0.4 (0.2,0.6)	0.4 (0.1,0.6)	12.4 (-44.5,86.3)	25.9 (13.4,43.3)	26.9 (8.8,40.4)	3.9 (-49.2,84.5)
Togo	0.3 (0.2,0.5)	0.3 (0.1,0.6)	3.6 (-49.4,62.2)	0.3 (0.2,0.5)	0.3 (0.1,0.5)	2.5 (-50.9,57.5)	22.2 (11.8,32.1)	21.2 (7.1,34.2)	-4.2 (-54.5,55.9)
Eastern Sub-Saharan Africa	1.5 (0.8,2.7)	0.9 (0.5,1.2)	-40.2 (-66.9,23)	1.5 (0.8,2.7)	0.9 (0.5,1.2)	-41.1 (-67.3,20.3)	87.5 (42.6,152.9)	50.2 (30.6,67.8)	-42.6 (-68,24.1)
Burundi	0.7 (0.4,1.3)	0.6 (0.3,0.8)	-23 (-59.2,76.5)	0.7 (0.4,1.2)	0.6 (0.3,0.8)	-23 (-59.4,76.2)	47.7 (24.5,83)	33.3 (16.2,52.8)	-30.2 (-64.8,68.4)
Comoros	0.6 (0.4,1)	0.7 (0.4,1)	5 (-37,73.8)	0.6 (0.4,0.9)	0.7 (0.4,1)	3.2 (-37.5,67.2)	40.4 (25.8,61.4)	39.8 (22.4,61.9)	-1.6 (-41.3,67.9)
Djibouti	0.5 (0.3,0.8)	0.5 (0.2,0.9)	2 (-47.2,68.6)	0.5 (0.3,0.7)	0.5 (0.2,0.8)	0.8 (-47.8,62.1)	30 (17.3,49.6)	26.8 (12.7,50.1)	-10.6 (-52.5,53.3)

Eritrea	0.6 (0.3,1.1)	0.6 (0.3,1)	6.4 (-48,125.9)	0.6 (0.3,1.1)	0.6 (0.3,1)	5.6 (-47.2,119.4)	37.8 (21.3,68.2)	37.3 (18.1,59.6)	-1.2 (-55.8,122.7)
Ethiopia	4.2 (1.8,8.1)	2.1 (1.3,3.1)	-49 (-72.7,21.7)	4.2 (1.8,8.2)	2.1 (1.2,3.1)	-50 (-73.4,18)	223.9 (85.8,429.7)	111.5 (72.1,163.7)	-50.2 (-72.7,27.9)
Kenya	0.4 (0.3,0.7)	0.4 (0.2,0.6)	-9.9 (-48.5,51)	0.4 (0.3,0.6)	0.4 (0.2,0.6)	-9.8 (-48.1,48.6)	26.2 (15.8,41.3)	21 (11.4,30.4)	-19.7 (-55,48.5)
Madagascar	0.6 (0.4,0.9)	0.5 (0.3,0.8)	-7.6 (-47.6,63.2)	0.6 (0.4,0.9)	0.5 (0.3,0.8)	-7.8 (-47.8,61.8)	38 (22.8,61.7)	31.8 (18.9,45.8)	-16.3 (-53.8,53.9)
Malawi	0.3 (0.2,0.4)	0.2 (0.1,0.4)	-35.9 (-69.2,35.2)	0.3 (0.2,0.4)	0.2 (0.1,0.3)	-36.6 (-69.3,29.3)	21 (12.7,30)	11.5 (4.5,22.9)	-45 (-75.8,22.8)
Mozambique	1.1 (0.7,1.7)	0.7 (0.4,1.2)	-33.9 (-68.7,51.2)	1.1 (0.6,1.7)	0.7 (0.4,1.1)	-33.9 (-68.9,48)	75.5 (43.6,125.2)	42 (20.6,77)	-44.4 (-76.6,45.3)
Rwanda	0.9 (0.4,1.5)	0.5 (0.3,0.9)	-38.1 (-71.4,67.8)	0.9 (0.4,1.5)	0.5 (0.3,0.9)	-39.7 (-72.2,62.8)	56.9 (30,94.2)	31.7 (15.9,55.2)	-44.3 (-74.9,59.5)
Somalia	0.6 (0.3,1.2)	0.7 (0.3,1.1)	1.6 (-40,71)	0.6 (0.3,1.2)	0.7 (0.3,1.2)	2.4 (-39.8,72.7)	40.1 (17.6,78.1)	36.2 (19.3,60.1)	-9.7 (-50.2,67.5)
South Sudan	0.6 (0.3,1.1)	0.8 (0.4,1.3)	30.5 (-22.1,100.1)	0.6 (0.3,1.1)	0.8 (0.4,1.3)	28.9 (-23,95.7)	42.1 (20.9,76.5)	51.3 (25.6,81.7)	22 (-26.8,94.8)
United Republic of Tanzania	0.7 (0.5,1)	0.6 (0.3,0.9)	-7.5 (-52.5,69.1)	0.6 (0.4,1)	0.6 (0.3,0.9)	-9.6 (-53.9,59.5)	43.2 (28.3,68.8)	36.2 (19.7,55.7)	-16.3 (-59.4,58.2)
Uganda	0.3 (0.1,0.4)	0.3 (0.1,0.5)	-3.8 (-55.1,65)	0.3 (0.1,0.4)	0.3 (0.1,0.4)	-6.2 (-55.5,58.9)	20.3 (10.8,30.4)	17.9 (6,31.6)	-11.8 (-61.3,57.4)
Zambia	0.8 (0.5,1.2)	0.5 (0.2,0.8)	-34.7 (-71.1,25.4)	0.7 (0.5,1.2)	0.5 (0.2,0.7)	-36.2 (-71.6,20.1)	49.8 (30,82)	29.2 (14,45.8)	-41.4 (-76.1,23.6)
Central Sub-Saharan Africa	0.5 (0.3,0.9)	0.4 (0.2,0.6)	-13.1 (-50.6,63)	0.5 (0.3,0.9)	0.4 (0.2,0.6)	-13.8 (-50.9,62.5)	33.3 (17.6,62)	25.4 (13.7,36.6)	-24 (-56.8,54.9)
Angola	0.6 (0.3,1.1)	0.4 (0.2,0.7)	-22.1 (-63.8,81.5)	0.6 (0.3,1.1)	0.4 (0.2,0.7)	-23.5 (-64.8,81.2)	38.4 (17.7,78.3)	26.1 (13.7,39.5)	-32 (-68.6,75.9)
Central African Republic	0.6 (0.3,1.1)	0.6 (0.3,1.1)	-3.3 (-41.4,74.8)	0.6 (0.3,1.1)	0.6 (0.3,1.1)	-3.1 (-41.5,76.2)	38.6 (18.2,76.9)	34.6 (17.4,67.2)	-10.4 (-46.5,72.7)
Congo	0.5 (0.3,0.9)	0.5 (0.2,0.7)	-3.9 (-46,80.8)	0.5 (0.3,0.8)	0.5 (0.2,0.7)	-5.1 (-47.3,79.9)	30 (16.5,50.7)	26 (12.2,38)	-13.3 (-52.1,76.8)
Democratic Republic of the Congo	0.5 (0.3,0.9)	0.4 (0.2,0.6)	-12 (-48.8,69.8)	0.5 (0.3,0.8)	0.4 (0.2,0.7)	-12.2 (-49.7,67.1)	32 (16.8,60.4)	24.5 (12.9,37.3)	-23.5 (-55.8,54.9)

Equatorial Guinea	0.5 (0.3,0.9)	0.4 (0.1,0.7)	-25.6 (-71.3,75.8)	0.5 (0.3,0.9)	0.4 (0.1,0.7)	-30.5 (-74.4,63.2)	33.9 (17,63.6)	21.1 (7.7,38)	-37.7 (-77.9,62.6)
Gabon	0.4 (0.2,0.6)	0.4 (0.1,0.6)	-3.8 (-54,66.7)	0.4 (0.2,0.5)	0.4 (0.1,0.6)	-6.8 (-55.9,61.2)	23.1 (14.6,33.3)	19.5 (7.5,33.9)	-15.4 (-60.5,62)

Table S7 Age-standardized incidence rates, death rates, and DALY rates in 1990 and 2021, and the percentage change in the age-standardized rates for AML among WCBA (15–49 years), by country.

	Incidence (95% UI)			Deaths (95% UI)			DALYs (95% UI)		
	ASRs per 100000 (95% UI), 1990	ASRs per 100000 (95% UI), 2021	Percentage changes in ASRs from 1990 to 2021	ASRs per 100000 (95% UI), 1990	ASRs per 100000 (95% UI), 2021	Percentage changes in ASRs from 1990 to 2021	ASRs per 100000 (95% UI), 1990	ASRs per 100000 (95% UI), 2021	Percentage changes in ASRs from 1990 to 2021
Global	1.6 (1.3,2)	1.5 (1.3,1.8)	-5.3 (-20.5,13)	1.5 (1.2,1.9)	1.4 (1.1,1.6)	-11.7 (-26.1,6.1)	61 (43.3,78.9)	46.6 (38,59.2)	-23.7 (-41.3,0)
High-income North America	2.9 (2.7,3)	3.1 (2.8,3.2)	7.4 (2.4,11.6)	2.3 (2.2,2.4)	2.2 (2,2.4)	-4.2 (-8.7,-0.4)	72.8 (70.2,74.8)	59.8 (56.3,62.1)	-17.9 (-21,-14.9)
Canada	2.4 (2.2,2.6)	2.6 (2.2,3)	6.6 (-9,24.6)	2 (1.9,2.2)	1.9 (1.6,2.2)	-6.3 (-19.7,8.4)	62.1 (57.3,67.6)	50 (43.4,57)	-19.5 (-31.1,-7.1)
Greenland	1.7 (1.1,2.2)	1.1 (0.7,1.5)	-35.2 (-51.3,-10.4)	1.7 (1.1,2.2)	1.1 (0.6,1.4)	-38.3 (-53.7,-14.6)	54.8 (34.3,69.3)	31.5 (19.2,41.6)	-42.5 (-57.6,-21.9)
United States of America	2.9 (2.7,3)	3.1 (2.8,3.3)	7.7 (2.4,12.1)	2.4 (2.2,2.4)	2.3 (2,2.4)	-3.8 (-8.5,0.2)	73.9 (71.4,75.8)	60.9 (57.2,63.3)	-17.6 (-20.9,-14.6)
Australasia	2.8 (2.5,3)	3.4 (2.9,4)	25 (6.9,47.2)	1.9 (1.8,2.1)	2.1 (1.8,2.4)	6.6 (-8.6,25)	61.6 (57.8,65.7)	54.8 (48,62.4)	-11.1 (-23.2,3.1)
Australia	2.8 (2.5,3)	3.7 (3.2,4.4)	34.1 (13,60.3)	1.9 (1.7,2)	2.2 (1.8,2.5)	16.4 (-2,38.8)	59.6 (55.6,64.2)	56 (48.5,64.7)	-5.9 (-19.2,10.4)
New Zealand	2.6 (2.3,2.8)	1.9 (1.7,2.1)	-27.1 (-37.2,-16.3)	2.3 (2.1,2.6)	1.6 (1.4,1.8)	-33.5 (-42.6,-22.9)	71.5 (64.8,77.1)	47.7 (42.9,52.8)	-33.3 (-41.4,-24.2)
High-income Asia Pacific	1.9 (1.7,2.1)	1.6 (1.4,1.8)	-15.4 (-26.4,-6.1)	1.6 (1.5,1.8)	1.2 (1,1.3)	-27.3 (-37,-19.8)	66.8 (57.3,74.5)	38.3 (33.4,41.8)	-42.6 (-49.5,-34.5)
Brunei Darussalam	2.9 (2,4)	2.1 (1.5,2.9)	-27 (-46.9,2.1)	2.9 (1.9,4)	2 (1.4,2.8)	-29.5 (-48.9,-1.9)	108.1 (72.9,153.6)	70.8 (50.8,98)	-34.6 (-52.4,-8.7)
Japan	1.8 (1.7,1.9)	1.5 (1.3,1.6)	-17.3 (-24.6,-10.6)	1.5 (1.4,1.6)	1.1 (1,1.2)	-26.3 (-32.9,-21.9)	59.2 (57.5,60.6)	35.3 (33,37)	-40.4 (-43.6,-37.8)
Singapore	2.2 (2,2.4)	2 (1.7,2.3)	-10.5 (-24,6.6)	2.1 (1.9,2.3)	1.5 (1.3,1.7)	-28.1 (-38.5,-14.3)	74.3 (67.5,82.5)	51 (44.3,58.6)	-31.4 (-41.9,-19.5)
Republic of Korea	2 (1.4,2.7)	1.8 (0.9,2.2)	-11.3 (-54.6,24.7)	1.9 (1.4,2.6)	1.3 (0.7,1.7)	-31.1 (-66.3,-2.8)	84.1 (50.7,112.6)	43.8 (24.1,54.5)	-47.8 (-68.1,-25.3)
Western Europe	2 (1.9,2.1)	2.4 (2.1,2.5)	19.2 (10,27.9)	1.8 (1.7,1.9)	1.9 (1.7,2.1)	8.4 (-0.4,16.6)	60.5 (57.9,62.7)	52.6 (49.2,55.9)	-13.1 (-18.4,-7.4)

Andorra	1.3 (0.8,2)	1.1 (0.7,1.6)	-17.7 (-49.8,36.4)	1.2 (0.8,1.8)	0.9 (0.5,1.3)	-28 (-55.6,20.3)	40.8 (24.2,61.7)	26.5 (17,38.1)	-35 (-62,8.8)
Austria	1.9 (1.7,2.1)	2.2 (1.9,2.5)	14.5 (-2.2,33.5)	1.8 (1.6,1.9)	1.8 (1.5,2.1)	2.8 (-12,19.3)	59.3 (54.7,64.5)	49.2 (43.1,55.6)	-17 (-28,-3.7)
Belgium	2.1 (1.9,2.3)	2.7 (2.2,3.2)	27.6 (4.8,53.7)	1.9 (1.7,2.2)	2.2 (1.8,2.7)	14.4 (-6.8,37.8)	62.7 (57,69.1)	61.8 (53.9,70.8)	-1.6 (-16.5,15.5)
Cyprus	2.4 (1.5,4)	2.2 (1.4,2.9)	-7.7 (-49.8,35.6)	2.5 (1.5,4)	1.9 (1.1,2.5)	-22.1 (-58.2,17.3)	66.4 (45.4,103.2)	47.5 (30.2,62.3)	-28.5 (-57.2,0.4)
Denmark	2.7 (2.5,2.9)	2.2 (1.9,2.5)	-19.4 (-31.3,-5.7)	2.7 (2.5,2.9)	2.1 (1.8,2.4)	-22.2 (-33.2,-8.8)	89 (82.6,95.6)	55.8 (49,63.4)	-37.2 (-46.5,-27)
Finland	1.8 (1.6,2)	2.1 (1.8,2.4)	16 (-1.9,36.2)	1.5 (1.3,1.6)	1.4 (1.2,1.6)	-7.2 (-21.3,10.1)	50.1 (45.7,54.8)	42 (36.5,48.3)	-16.2 (-28.2,-1.3)
France	1.9 (1.7,2)	2.3 (2,2.7)	25.8 (6.1,46.9)	1.8 (1.6,1.9)	2 (1.6,2.3)	11.3 (-5.7,29.5)	58 (53.8,62.2)	52.1 (44.9,59.4)	-10.3 (-23.4,4.1)
Germany	1.9 (1.6,2.1)	2.5 (2.1,2.8)	29.7 (10.6,58.3)	1.8 (1.5,2)	2.1 (1.8,2.4)	17.2 (-1.1,43)	61.7 (54.4,68.2)	55.6 (49,62.2)	-9.9 (-23.1,6.7)
Greece	1.9 (1.8,2)	2.8 (2.5,3.1)	48.7 (33.3,66.3)	1.7 (1.6,1.9)	2.4 (2.2,2.7)	40.8 (25.8,58)	54.4 (51.2,58.2)	66.9 (61.2,72.7)	23.1 (11.3,36.1)
Iceland	2 (1.7,2.3)	2.3 (1.9,2.7)	14 (-7.5,40.4)	1.8 (1.6,2.1)	1.8 (1.5,2.1)	-1 (-20,22.2)	57.6 (50.3,65.4)	49.3 (42.5,56.4)	-14.5 (-29.7,4.5)
Ireland	1.7 (1.5,1.9)	1.9 (1.6,2.2)	9.8 (-7.7,31.7)	1.6 (1.5,1.8)	1.5 (1.3,1.8)	-8 (-22.8,10.5)	49.1 (44.6,53.9)	40 (34.1,46.1)	-18.5 (-31.3,-2.8)
Israel	2.7 (2.4,3)	2.4 (2.1,2.8)	-9.3 (-23.9,8.7)	2.6 (2.3,2.9)	2.1 (1.8,2.5)	-18 (-31.2,-1.6)	86.2 (76.9,95.6)	57.7 (50.3,64.6)	-33 (-43,-22.1)
Italy	1.8 (1.7,1.9)	2.4 (2.1,2.6)	32.1 (19.9,43.6)	1.6 (1.5,1.7)	1.9 (1.7,2.1)	17.3 (6.8,26.7)	58.7 (56.2,60.8)	53.6 (49,57.4)	-8.6 (-15.2,-2.1)
Luxembourg	2.5 (2.3,2.7)	2.9 (2.6,3.3)	16.1 (0.3,33.6)	2.4 (2.2,2.6)	2.4 (2.1,2.7)	2.5 (-12.2,18.5)	81.8 (75.7,88.5)	64.4 (57.3,72.3)	-21.2 (-31.9,-8.8)
Malta	1.8 (1.6,2.1)	2.3 (2,2.7)	29.7 (4.1,58.8)	1.8 (1.5,2)	2 (1.7,2.3)	12.7 (-8.1,38.1)	52.3 (46.3,59.7)	55.7 (48.1,64.5)	6.4 (-12.4,28.6)
Monaco	4 (2.5,6.5)	6 (3.7,8.2)	49.3 (-8.6,135.6)	3.5 (2.2,5.6)	4.8 (3,6.7)	35.8 (-15.7,114.9)	122.1 (77.4,192.1)	150.8 (97.1,206.6)	23.4 (-19.8,88.1)
Netherlands	2.1 (1.9,2.3)	2.3 (2,2.7)	9.3 (-4.6,27.1)	1.9 (1.8,2.1)	1.9 (1.6,2.2)	-3.2 (-14.9,12.5)	64.3 (59.7,68.9)	51.4 (45.8,57.8)	-20.1 (-29.5,-9.8)
Norway	2.2 (2.1,2.3)	2.4 (2.1,2.6)	8.8 (-2.3,20)	2.1 (2,2.2)	2.1 (1.9,2.3)	1 (-9.5,12.2)	69.3 (65.9,73.4)	55.6 (51.1,60.4)	-19.8 (-27,-12.4)

Portugal	1.8 (1.7,2)	2 (1.7,2.4)	11.6 (-5.8,30.5)	1.8 (1.6,1.9)	1.7 (1.5,2)	-1 (-16.6,16)	67.9 (62.2,74.5)	51.1 (45.1,57.7)	-24.7 (-35.4,-12.8)
San Marino	1.5 (0.9,2.3)	1 (0.5,1.6)	-32.3 (-61.9,16.6)	1.3 (0.8,2.1)	0.8 (0.4,1.3)	-39.9 (-66,3.6)	37 (23.5,60.1)	23.2 (12.2,38.7)	-37.3 (-62.5,7.1)
Spain	1.6 (1.5,1.7)	1.9 (1.6,2.2)	20.2 (3.4,37)	1.4 (1.3,1.6)	1.5 (1.3,1.7)	5.8 (-8.5,21.4)	53.2 (49.5,57.1)	43.7 (38.5,49)	-17.9 (-28.7,-5.1)
Sweden	2.1 (1.9,2.3)	2.2 (1.9,2.6)	6.9 (-11.1,27)	1.9 (1.7,2.1)	1.8 (1.5,2.2)	-3.5 (-20.3,14)	61.6 (56.1,67.2)	47.7 (40.5,56.2)	-22.6 (-34.5,-7.2)
Switzerland	2.3 (2,2.6)	2.8 (2.4,3.3)	24.4 (1.6,51.1)	1.7 (1.5,1.9)	1.6 (1.3,1.9)	-3.7 (-20.9,14.8)	55.8 (49.4,61.9)	46.2 (39.9,53.6)	-17.1 (-30.5,-1.1)
United Kingdom	2.5 (2.3,2.5)	2.5 (2.3,2.6)	2.2 (-3.2,6.7)	2 (1.9,2.1)	2.1 (1.9,2.2)	3.6 (-2,8.3)	63 (61.1,65)	54.2 (51.4,56.2)	-14 (-17.6,-10.8)
Southern Latin America	1.7 (1.5,1.9)	1.6 (1.4,1.8)	-6 (-21.4,11.5)	1.7 (1.5,1.9)	1.5 (1.3,1.7)	-10.8 (-25.1,6.3)	70.5 (63.6,77)	52.8 (46.4,60.3)	-25.2 (-36.7,-11.9)
Argentina	1.8 (1.5,2)	1.6 (1.4,1.9)	-9.7 (-26.3,8.9)	1.8 (1.5,2)	1.5 (1.3,1.8)	-12.6 (-28.6,6.4)	74.4 (65.3,83.7)	54.4 (47.3,62.6)	-26.8 (-39.7,-12.4)
Chile	1.5 (1.3,1.6)	1.5 (1.3,1.7)	2.8 (-12.5,19.2)	1.4 (1.3,1.6)	1.3 (1.2,1.5)	-6.8 (-21,7.5)	58.6 (53.4,63.6)	45.5 (40.2,51.4)	-22.5 (-33.9,-9.5)
Uruguay	1.9 (1.7,2.2)	2.1 (1.8,2.4)	8.2 (-11.7,34.6)	1.9 (1.7,2.1)	1.9 (1.6,2.2)	3.2 (-15,26.7)	80.8 (72.8,89.5)	70 (59.8,80.9)	-13.4 (-28.7,4.6)
Eastern Europe	1.4 (1.3,1.6)	1.4 (1.2,1.5)	-6.1 (-19.2,8.4)	1.3 (1.2,1.4)	1.2 (1.1,1.3)	-10.7 (-22.8,3.2)	62.5 (57.5,67.6)	43.6 (39.1,49)	-30.3 (-39.3,-19.3)
Belarus	1.2 (0.9,1.4)	2 (1.5,2.5)	70.4 (21.8,138.1)	1.1 (0.9,1.3)	1.7 (1.3,2.1)	54.1 (10.3,116)	47.1 (37.8,58.1)	61.8 (48.6,76.1)	31.2 (-3.8,79.4)
Estonia	1.6 (1.3,1.8)	2 (1.6,2.3)	25.2 (0,58.7)	1.4 (1.2,1.7)	1.6 (1.3,1.9)	11.6 (-10.9,41.4)	66.4 (57.1,76.7)	60.3 (51.1,70.3)	-9.2 (-26.7,13.4)
Latvia	1.9 (1.6,2.2)	1.7 (1.4,2)	-10.4 (-29.4,13.7)	1.7 (1.5,2)	1.5 (1.2,1.7)	-16.6 (-34.1,5.8)	76.7 (67.1,87.1)	55 (45.8,65.7)	-28.3 (-43.1,-9.6)
Lithuania	1.6 (1.3,1.8)	2.2 (1.8,2.7)	41.4 (12.4,79.8)	1.5 (1.2,1.7)	2 (1.6,2.4)	36.6 (9.1,74.4)	64.4 (54.4,73.5)	69.9 (58,82.8)	8.6 (-12.8,38.8)
Republic of Moldova	1.5 (1.3,1.6)	1.1 (1,1.3)	-22.6 (-34.6,-8.7)	1.4 (1.3,1.5)	1 (0.9,1.2)	-26.8 (-38.2,-13.7)	60.6 (55.3,67.1)	38.3 (33,44.4)	-36.7 (-47.8,-24.5)
Russian Federation	1.3 (1.2,1.5)	1.3 (1.2,1.5)	0 (-15.6,15.9)	1.2 (1.1,1.4)	1.2 (1,1.3)	-5.4 (-19.9,9.5)	57.8 (52.4,63.9)	41.7 (37.1,45.8)	-27.9 (-39.1,-16.5)
Ukraine	1.8 (1.5,2.1)	1.2 (0.8,1.8)	-30.3 (-55,4.6)	1.6 (1.4,1.9)	1.1 (0.7,1.6)	-32 (-56.1,0.4)	78.6 (67.1,94)	44.8 (29.8,63.6)	-43 (-62.9,-16.1)

Central Europe	1.5 (1.4,1.7)	1.9 (1.7,2)	20.8 (8.2,33.7)	1.5 (1.4,1.6)	1.6 (1.5,1.8)	10.3 (-1.2,22.6)	55.4 (51.1,61.6)	50.5 (45.9,55.2)	-8.8 (-18.6,1.2)
Albania	1.5 (0.9,2.5)	1.4 (0.9,2.3)	-2.6 (-38.8,60.7)	1.5 (0.9,2.5)	1.3 (0.8,2.1)	-11.1 (-43.6,44.8)	53.8 (33.5,87.4)	41.7 (25.6,65.7)	-22.4 (-53.4,32.7)
Bosnia and Herzegovina	1.3 (0.9,2.1)	1.5 (0.9,2.2)	16.6 (-26.1,88.6)	1.3 (0.9,2.1)	1.4 (0.8,2.1)	7.7 (-31.6,75.7)	44.3 (30.1,66.2)	43.3 (27.7,61.9)	-2.3 (-37.4,54.7)
Bulgaria	1.4 (1.3,1.6)	2.6 (2,3.2)	85.4 (40.7,134.7)	1.3 (1.2,1.5)	2.4 (1.8,3)	77.1 (35.7,124.9)	54.5 (48.1,63.5)	84.1 (64.5,104.6)	54.4 (15.5,101.6)
Croatia	1.5 (1.1,1.9)	2.3 (1.6,3.1)	53.7 (2,135.2)	1.4 (1,1.8)	1.9 (1.3,2.6)	33.5 (-11,104)	43.6 (33.5,54.5)	55.7 (41.4,73.8)	27.8 (-12.7,89.7)
Czechia	2 (1.8,2.2)	2.2 (1.8,2.7)	12.1 (-10.9,39)	1.9 (1.7,2.1)	1.9 (1.5,2.3)	-1.1 (-21.7,24.6)	60.8 (56.2,66.2)	51.7 (41.5,62.8)	-15 (-32.3,5.1)
Hungary	1.9 (1.8,2.1)	2.2 (1.9,2.6)	13.4 (-6,34.9)	1.9 (1.7,2)	1.9 (1.6,2.2)	1.3 (-15.3,21)	64.8 (60.5,69.5)	59.1 (49.1,70.9)	-8.7 (-25.2,10)
Montenegro	1.9 (1.3,2.8)	2 (1.3,2.8)	8.2 (-27.8,56.4)	1.7 (1.2,2.5)	1.7 (1.1,2.4)	3.1 (-32.2,50.3)	61 (43.3,91.6)	53.4 (36,72.7)	-12.4 (-37.7,22.3)
North Macedonia	2.2 (1.7,3.2)	2.1 (1.4,2.9)	-3.6 (-30.9,35.5)	2.2 (1.6,3.2)	2 (1.2,2.7)	-9.5 (-36.5,26.3)	76.9 (55.7,111.5)	58.3 (38.5,80.1)	-24.3 (-45.5,8.9)
Poland	1.6 (1.4,1.9)	1.7 (1.5,1.9)	9.8 (-10.9,32.5)	1.5 (1.3,1.8)	1.5 (1.4,1.7)	1.6 (-17.9,22.2)	56.5 (51.3,66.7)	44.9 (39.8,49.9)	-20.6 (-34,-6.4)
Romania	1.1 (1,1.2)	1.6 (1.3,1.9)	50.7 (21.3,84.1)	1 (0.9,1.2)	1.4 (1.2,1.7)	40.6 (14.2,69.5)	47.2 (41.7,54.1)	47.9 (39.4,56.4)	1.5 (-18.7,25.4)
Serbia	1.7 (1.2,2.5)	1.8 (1.1,2.4)	6.9 (-26.4,56.8)	1.7 (1.2,2.4)	1.6 (1,2.1)	-4 (-34.8,40.7)	58.5 (40.9,85.3)	47.9 (31.5,64.7)	-18.2 (-46.1,21.1)
Slovakia	1.9 (1.4,3)	1.9 (1.2,2.7)	0 (-34.6,60.2)	1.8 (1.3,2.8)	1.7 (1,2.4)	-8.7 (-40.7,48.6)	66.5 (48.6,100.8)	55.2 (34.8,76.8)	-16.9 (-44,28.6)
Slovenia	1.1 (0.9,1.2)	1.3 (1.1,1.7)	27 (-2.5,61.2)	1 (0.9,1.1)	1 (0.8,1.2)	4.6 (-19,33.7)	32.1 (28.5,36.5)	29.5 (23.7,35.9)	-8 (-28.8,16.5)
Central Asia	1 (0.9,1.2)	1.1 (0.9,1.2)	5.8 (-13.7,26)	1 (0.8,1.1)	1 (0.8,1.2)	3.6 (-15.2,23.8)	47.5 (39,56)	44.1 (37.3,51.8)	-7.2 (-25.1,11.8)
Armenia	0.7 (0.6,0.9)	1.1 (0.9,1.3)	45.6 (3.7,107.7)	0.7 (0.5,0.9)	1 (0.8,1.2)	40.8 (0.9,98)	30.3 (24.1,39.1)	35.4 (29.6,42)	16.7 (-16.5,61.6)
Azerbaijan	1.2 (0.7,2)	1.1 (0.6,1.8)	-6.4 (-44.7,60.7)	1.1 (0.7,1.9)	1 (0.6,1.7)	-9.5 (-46.3,58.8)	57.4 (35.6,97.8)	48.4 (29.9,80.8)	-15.6 (-47.1,38.9)
Georgia	0.6 (0.5,0.7)	1.6 (1.4,1.8)	163.1 (106.3,241.4)	0.6 (0.4,0.7)	1.5 (1.3,1.7)	164.6 (106.1,241.6)	30.1 (23.1,37.1)	58.3 (50.5,66.3)	93.5 (51.9,155.2)

Kazakhstan	1.2 (1,1.3)	1 (0.8,1.3)	-9.6 (-31.5,16.3)	1.1 (0.9,1.3)	1 (0.8,1.2)	-13.2 (-34.2,11.1)	52.6 (43.8,60.3)	38.3 (31.1,46.8)	-27.1 (-45,-8.3)
Kyrgyzstan	0.5 (0.4,0.5)	0.9 (0.8,1.1)	107.4 (65.1,156.8)	0.4 (0.4,0.5)	0.9 (0.7,1)	101 (60.4,149.5)	22.4 (19.4,25.5)	39.6 (32.3,47.1)	76.7 (38.8,120.7)
Mongolia	1.2 (0.6,2)	1.1 (0.7,1.6)	-7.3 (-50.5,94.3)	1.2 (0.6,2)	1.1 (0.6,1.6)	-9.7 (-51.3,88)	54.8 (27.1,96)	45.7 (27.1,68.6)	-16.6 (-57.2,89.4)
Tajikistan	0.9 (0.5,1.5)	0.8 (0.4,1.3)	-12.4 (-54.1,71.7)	0.8 (0.4,1.4)	0.7 (0.4,1.2)	-13.3 (-53.5,71.4)	42.7 (20.5,73.9)	36 (17,65.7)	-15.7 (-57.8,71.8)
Turkmenistan	1 (0.8,1.2)	1 (0.7,1.3)	-0.3 (-29,35.2)	1 (0.8,1.1)	0.9 (0.7,1.2)	-3.2 (-31.4,31.5)	50.1 (37.4,58.5)	45.3 (33.2,59.8)	-9.5 (-37.1,21.2)
Uzbekistan	1.1 (0.9,1.4)	1 (0.8,1.2)	-12.7 (-36.7,26.9)	1.1 (0.9,1.4)	0.9 (0.8,1.2)	-14.2 (-37.5,24.7)	54.2 (43.4,69.7)	45.9 (36.8,57.6)	-15.3 (-38.9,22.5)
Central Latin America	1.3 (1.2,1.3)	1.5 (1.3,1.7)	14.6 (-2.5,31)	1.3 (1.2,1.3)	1.4 (1.2,1.6)	10.2 (-6.1,25.6)	58.2 (55.3,61.5)	55.2 (47.6,63.5)	-5.1 (-20.2,8.8)
Colombia	1.3 (1.2,1.4)	1.6 (1.3,1.9)	22.6 (0,49.4)	1.3 (1.2,1.4)	1.5 (1.2,1.7)	15.7 (-5.8,39.1)	54.9 (50.3,59.4)	54.3 (44.9,66.1)	-1.1 (-20.3,19.7)
Costa Rica	1.6 (1.5,1.9)	2.2 (1.8,2.5)	32 (7.5,59.4)	1.6 (1.4,1.8)	2 (1.7,2.2)	23.3 (-0.1,49.6)	62.2 (55.5,70)	74 (63.9,84.3)	18.9 (-0.8,41.9)
El Salvador	1.1 (0.7,1.8)	1.4 (0.8,1.9)	21 (-14.7,68.8)	1.1 (0.7,1.7)	1.3 (0.8,1.8)	14.6 (-19,61)	54 (31.3,86)	54.2 (31.3,78.3)	0.3 (-30.3,50.7)
Guatemala	0.8 (0.6,0.9)	1.1 (0.9,1.3)	41 (5.2,76.6)	0.8 (0.7,0.9)	1 (0.9,1.2)	36.1 (2.2,70)	37.8 (29.5,46.5)	45.6 (38.3,53.5)	20.8 (-10.8,59.8)
Honduras	1.5 (0.8,2.2)	1.6 (0.9,2.5)	6.3 (-31.4,59.8)	1.5 (0.8,2.2)	1.6 (0.9,2.5)	6.4 (-31,59.7)	69.5 (32,112.4)	59.1 (31.5,94.1)	-14.9 (-47.4,37.5)
Mexico	1.3 (1.3,1.4)	1.4 (1.2,1.7)	8 (-8.5,24.9)	1.3 (1.3,1.4)	1.4 (1.2,1.6)	3.8 (-12.2,19.5)	61.4 (59.3,64.2)	55.5 (47.3,63.2)	-9.7 (-23.7,3.9)
Nicaragua	0.8 (0.5,1.3)	0.9 (0.5,1.3)	3.7 (-23.9,40.7)	0.8 (0.5,1.3)	0.8 (0.5,1.3)	0.1 (-26.7,36.5)	41.4 (24.9,67.2)	34.5 (21.9,54.5)	-16.7 (-44,21.3)
Panama	1.3 (1.1,1.4)	1.7 (1.3,2)	31.5 (2.1,64)	1.2 (1.1,1.4)	1.5 (1.2,1.9)	24.5 (-4.2,54.3)	57.9 (51.9,65.7)	64.4 (51.4,78.2)	11.2 (-14.2,38.4)
Venezuela (Bolivarian Republic of)	1.3 (1.2,1.5)	1.5 (1.1,2.1)	20.1 (-18.6,67)	1.3 (1.1,1.4)	1.5 (1.1,2)	17.7 (-18.7,63.7)	58.5 (53.3,66.1)	59.7 (41.9,82.6)	2.1 (-33.5,43.3)
Andean Latin America	1.4 (1,1.9)	1.7 (1.1,2.2)	24.2 (-8.7,73.9)	1.4 (1,1.9)	1.6 (1.1,2.2)	17.8 (-13.9,63.7)	59.5 (39.5,82.9)	62.7 (41.2,81.1)	5.4 (-27.4,56.5)

Bolivia (Plurinational State of)	1.9 (1,3.3)	2.1 (1.2,3.3)	11.5 (-29.2,77.2)	2 (1.1,3.4)	2.1 (1.2,3.3)	9.7 (-29.8,75.9)	83.3 (43.1,155.7)	82 (45.5,124.3)	-1.7 (-45.1,74.3)
Ecuador	1.3 (1.1,1.4)	1.8 (1.4,2.2)	41.7 (10.1,82.9)	1.3 (1.1,1.5)	1.7 (1.4,2.2)	34.7 (4.4,74.5)	50.7 (45.6,57.3)	62.8 (50.2,78.5)	23.8 (-4.3,58.1)
Peru	1.3 (0.8,1.9)	1.5 (0.8,2.2)	22 (-22.5,90.4)	1.3 (0.8,1.9)	1.5 (0.8,2.1)	13.6 (-26.8,78.4)	56.2 (33,84.5)	56.6 (29.4,81.7)	0.7 (-39.2,68.8)
Caribbean	1.5 (1.3,1.9)	1.5 (1.2,1.8)	-3.6 (-18.7,11.7)	1.5 (1.3,1.8)	1.4 (1.1,1.7)	-6.8 (-21.8,8.4)	65.6 (51.6,91.2)	55.9 (42.4,75.4)	-14.8 (-30.6,2.4)
Antigua and Barbuda	1.4 (1.3,1.6)	1.9 (1.8,2.1)	35.5 (14.3,59.9)	1.4 (1.2,1.6)	1.8 (1.7,2)	33.2 (11.8,56.9)	57.7 (51.1,65.2)	66.6 (59.9,72.8)	15.4 (-2.5,34.9)
Barbados	2.1 (1.9,2.4)	2.9 (2.2,3.6)	37.3 (5.2,73.8)	2 (1.8,2.3)	2.7 (2.1,3.3)	33.9 (1.9,67.3)	95.2 (83.9,106.9)	108.3 (82.5,135.1)	13.8 (-13.3,44)
Belize	0.4 (0.3,0.4)	0.5 (0.4,0.6)	39.5 (0.2,95)	0.3 (0.3,0.4)	0.5 (0.4,0.6)	36.5 (-2.3,88.8)	16.3 (13.2,19.9)	18.6 (15.4,21.8)	13.7 (-18.8,53.8)
Bermuda	2.4 (2,2.8)	1.6 (1.2,2.1)	-33.7 (-51.4,-12)	2.3 (2,2.7)	1.3 (1,1.7)	-43.6 (-58.6,-25.3)	84.6 (69.5,102.9)	46.5 (35.5,60)	-45.1 (-61.8,-27.2)
Bahamas	1.2 (1,1.4)	1.4 (1.1,1.7)	13.8 (-16.1,52.8)	1.1 (1,1.3)	1.3 (1,1.6)	11.4 (-17.3,49.9)	55.9 (48.4,64)	57.2 (43.5,74.2)	2.3 (-25.2,37.5)
Cuba	1.6 (1.5,1.8)	1.5 (1.2,1.8)	-8 (-25.6,13.7)	1.5 (1.4,1.7)	1.3 (1.1,1.6)	-13.9 (-30.7,6.2)	63.6 (57.1,70)	50.7 (41.5,62)	-20.2 (-36.2,-1.4)
Dominica	2.7 (2,3.7)	2.9 (2,4.1)	10.1 (-21.5,51.4)	2.7 (2.1,3.8)	2.9 (2,4.1)	7.8 (-23.5,48)	95.3 (72.4,134.9)	112.7 (77.4,162.5)	18.3 (-17.1,63.4)
Dominican Republic	1.1 (0.8,1.6)	1 (0.7,1.6)	-8.2 (-36.4,28.4)	1.1 (0.8,1.6)	1 (0.7,1.6)	-10.1 (-38,25.3)	58.2 (35.7,79.1)	43.9 (30.2,63.8)	-24.7 (-49.5,11.8)
Grenada	1 (0.8,1.3)	1.3 (1.1,1.5)	20.9 (-10,62.8)	1 (0.8,1.2)	1.2 (1,1.4)	18.6 (-12,59.4)	53.5 (42.1,67.1)	53.3 (45.2,62.3)	-0.3 (-26.1,33.6)
Guyana	0.2 (0.2,0.3)	0.5 (0.3,0.6)	88.2 (19.6,195.5)	0.2 (0.2,0.3)	0.4 (0.3,0.6)	85 (18,189)	10.8 (8.3,13.8)	19.6 (13.8,26)	81.6 (12.9,185.8)
Haiti	1.6 (0.8,3.2)	1.5 (0.7,2.5)	-9.2 (-44.3,49.2)	1.6 (0.8,3.2)	1.5 (0.7,2.5)	-9.5 (-44.1,47.7)	74.4 (29.2,173.8)	63.9 (29.7,115.6)	-14.2 (-48.4,45.7)
Jamaica	0.6 (0.5,0.8)	1.2 (0.9,1.6)	92.4 (27.5,182.7)	0.6 (0.5,0.7)	1.1 (0.8,1.5)	87.3 (24.1,173.4)	30.4 (24.6,38.1)	46.7 (34,63)	53.7 (2,122.2)
Puerto Rico	2.2 (2,2.4)	2.1 (1.7,2.6)	-3.4 (-23.7,22.1)	2.1 (1.9,2.3)	1.8 (1.5,2.2)	-11.8 (-30,10.8)	84.5 (76.7,93.8)	61.8 (49.4,74.7)	-26.9 (-43,-8.9)

Saint Kitts and Nevis	0 (0,0)	0 (0,0)	-22 (-41.4,0.9)	0 (0,0)	0 (0,0)	-22.3 (-41.8,0)	0 (0,0)	0 (0,0)	-33.3 (-51.6,-14)
Saint Lucia	1.4 (1.2,1.6)	1.3 (1.1,1.6)	-5.1 (-30.1,23.3)	1.4 (1.2,1.6)	1.3 (1,1.5)	-8.6 (-32.9,18.4)	60.9 (53.3,71.3)	56 (45,69)	-8 (-32.3,19.9)
Saint Vincent and the Grenadines	1.4 (1.2,1.5)	1.1 (0.9,1.3)	-20.5 (-35.4,-2.6)	1.3 (1.2,1.5)	1 (0.9,1.2)	-22.6 (-37.2,-4.6)	66.1 (59.2,73.9)	49.9 (41.5,59.1)	-24.5 (-39.3,-7.8)
Suriname	0.9 (0.6,1.3)	1 (0.6,1.5)	12.8 (-24.5,68.3)	0.9 (0.6,1.3)	1 (0.6,1.4)	10.2 (-25.6,63.2)	42.4 (23,58.4)	45.9 (28.6,67.7)	8.2 (-29.3,65.7)
Trinidad and Tobago	1.4 (1.2,1.7)	1.6 (1.2,2.1)	15.6 (-20.4,58.8)	1.4 (1.2,1.6)	1.6 (1.1,2)	11.1 (-23.3,51.3)	59.7 (52.1,70.1)	61.4 (45.1,80.4)	3 (-30.3,42.6)
United States Virgin Islands	1.7 (1.2,2.3)	1 (0.7,1.4)	-41.5 (-59.1,-15.3)	1.6 (1.1,2.2)	0.9 (0.6,1.3)	-43.9 (-61.4,-19.3)	68.8 (47.4,93.5)	37.1 (25.7,52.9)	-46.1 (-62.7,-22.1)
Tropical Latin America	1.6 (1.6,1.7)	1.7 (1.5,1.8)	1.4 (-5.9,8.7)	1.6 (1.6,1.7)	1.6 (1.5,1.7)	-1.3 (-8.7,6.1)	69.1 (65.9,72.3)	57.5 (53.7,61.1)	-16.8 (-22.4,-10.5)
Brazil	1.7 (1.6,1.7)	1.7 (1.5,1.8)	0.9 (-6.6,8.1)	1.7 (1.6,1.7)	1.6 (1.5,1.7)	-1.8 (-9.4,5.3)	69.6 (66.6,72.9)	57.6 (53.9,60.9)	-17.3 (-22.9,-10.9)
Paraguay	1.2 (0.8,1.8)	1.5 (0.9,2.2)	27.2 (-17.5,88.5)	1.2 (0.8,1.8)	1.5 (0.9,2.1)	24.4 (-20.3,87)	48.9 (31.7,74.5)	53.6 (32.8,77.9)	9.7 (-26.6,65.9)
East Asia	1.3 (0.7,2)	0.9 (0.6,1.5)	-30 (-58.5,36.8)	1.3 (0.7,1.9)	0.8 (0.5,1.2)	-41.4 (-65,14.1)	67.8 (30.4,108.4)	33 (22.4,54)	-51.3 (-73,16.2)
China	1.3 (0.7,2)	0.9 (0.6,1.5)	-31.9 (-60.7,37)	1.3 (0.7,1.9)	0.7 (0.5,1.2)	-43.3 (-66.8,13.8)	68 (29.8,110)	32.1 (21.1,52.7)	-52.8 (-74.6,16.2)
Democratic People's Republic of Korea	1.4 (0.8,2.5)	1.2 (0.7,2.4)	-13.2 (-46.6,35)	1.4 (0.9,2.5)	1.2 (0.7,2.2)	-16.2 (-47.9,32.9)	68.6 (36.9,123.4)	51.5 (31.8,96.3)	-24.9 (-55.5,32)
Taiwan (Province of China)	1.4 (1.2,1.7)	2.1 (1.9,2.4)	51.2 (17,87.2)	1.3 (1.1,1.6)	1.7 (1.5,1.9)	32.7 (3.3,64)	53.3 (45.2,66.9)	61.8 (54.4,69.2)	16 (-11,43.8)
Southeast Asia	2.1 (1.4,3.2)	2.2 (1.5,2.8)	0.3 (-21.9,30.6)	2.2 (1.5,3.2)	2.1 (1.5,2.8)	-2.7 (-23.7,26.8)	86.9 (55.9,135.4)	79.8 (58.3,107.8)	-8.2 (-32.3,26.9)
Cambodia	2.6 (1.3,4.4)	2.5 (1.5,3.8)	-4.3 (-41.6,71.8)	2.7 (1.3,4.6)	2.5 (1.5,3.8)	-5.9 (-41.9,67.1)	107.1 (48.7,191.8)	96.8 (57.3,151.4)	-9.6 (-50.1,73.6)
Indonesia	2.2 (1.3,3.6)	2.3 (1.6,3.5)	6.2 (-26.2,54.5)	2.2 (1.4,3.6)	2.3 (1.6,3.5)	5.2 (-26.3,48.8)	91.5 (54.9,153.3)	89.3 (62.1,135.1)	-2.4 (-34.2,48.9)

Lao People's Democratic Republic	2.5 (1.1,4.8)	2.3 (1.4,3.5)	-7.4 (-42.8,69.7)	2.6 (1.2,4.9)	2.4 (1.4,3.6)	-8.9 (-43.7,66.3)	102.4 (41.6,212.4)	93.7 (54.4,144.8)	-8.4 (-48.7,83.6)
Malaysia	2 (1.3,2.7)	2 (1.3,2.9)	-1.9 (-28.3,35.9)	2 (1.3,2.8)	1.9 (1.3,2.8)	-5.4 (-31.8,30.1)	80.9 (50.7,110)	69.6 (48.3,96.6)	-13.9 (-37.2,22.6)
Maldives	0.9 (0.4,1.8)	0.7 (0.4,1)	-30.6 (-65.5,67.2)	1 (0.4,1.8)	0.6 (0.4,0.9)	-35.5 (-67.3,50.1)	38.7 (16.5,80.7)	23.2 (15.8,35.6)	-40.1 (-73.3,50.3)
Mauritius	1.1 (1,1.3)	1 (0.9,1.1)	-10.2 (-24.2,4.4)	1.1 (1,1.2)	1 (0.9,1.1)	-12.2 (-26.1,1.9)	48 (43.4,52.8)	35.1 (31.3,38.1)	-26.7 (-38.4,-15.6)
Myanmar	3.1 (1.6,5.4)	2.4 (1.5,3.5)	-22.5 (-50.7,33.1)	3.1 (1.7,5.5)	2.4 (1.5,3.6)	-23.6 (-51.2,27.8)	130.1 (58.8,259)	96.4 (60,150.8)	-25.9 (-54.7,39)
Philippines	2.2 (1.5,3)	2.1 (1.6,3)	-3.7 (-25.2,22.4)	2.2 (1.6,3.1)	2.1 (1.6,3.1)	-6.3 (-27.2,19.3)	86.6 (61,120.6)	81.9 (58.8,114.8)	-5.4 (-27.4,20.7)
Sri Lanka	3.1 (1.9,3.9)	1.7 (1,2.4)	-47.4 (-68,-14.4)	3.2 (1.9,3.9)	1.5 (1,2.3)	-50.9 (-69.3,-20.7)	126.5 (71.6,160)	55.6 (35.8,81.5)	-56.1 (-72.7,-20.8)
Seychelles	2.2 (1.2,2.9)	1.7 (1,2.3)	-18.9 (-39,8.4)	2.1 (1.2,2.8)	1.7 (1,2.3)	-21.3 (-40.3,4.9)	95.9 (59.4,126.8)	69.1 (44.2,90.5)	-27.9 (-46.1,-1.8)
Thailand	2.5 (1.5,3.5)	3.1 (1.5,4.2)	23.4 (-21,89.6)	2.5 (1.5,3.6)	2.9 (1.4,3.9)	14.1 (-28.8,77.1)	94.4 (58.7,135.7)	100.4 (50.9,134.7)	6.4 (-30,64.1)
Timor-Leste	2 (1,3.4)	2 (1.2,2.9)	0.2 (-36.9,70.3)	2.1 (1,3.5)	2 (1.3,3)	-1.8 (-38.1,65.8)	81 (36.5,150)	78.7 (51.7,115.9)	-2.9 (-43.2,86.3)
Viet Nam	0.9 (0.5,1.4)	0.8 (0.5,1.3)	-16.1 (-46.9,41.7)	1 (0.6,1.4)	0.7 (0.5,1.3)	-22.7 (-50.6,30.3)	31.8 (17.9,49.4)	26.7 (17.1,42.9)	-16.1 (-48.9,42.4)
Oceania	1.4 (0.5,2.1)	1 (0.5,1.5)	-25.2 (-46.6,23.6)	1.4 (0.5,2.2)	1 (0.5,1.5)	-25.6 (-46.5,24.5)	62.5 (19.6,100.6)	48.4 (22.5,70.7)	-22.5 (-46.2,33)
American Samoa	3.1 (1.3,4.3)	1.5 (1,2.9)	-51.1 (-72.3,36.3)	3.2 (1.3,4.4)	1.5 (1,2.9)	-52.5 (-73,31.7)	122.1 (54.6,167.3)	66 (44.1,120)	-45.9 (-68.1,33.5)
Cook Islands	0.6 (0.2,0.9)	0.3 (0.2,0.6)	-43.4 (-71,76.1)	0.6 (0.2,0.9)	0.3 (0.2,0.5)	-48.1 (-72.6,58.8)	24.3 (7.6,37.1)	13.5 (6.5,23.8)	-44.6 (-72.3,94.7)
Micronesia (Federated States of)	1.5 (0.4,2.6)	1.1 (0.6,1.7)	-29.5 (-55,67.1)	1.6 (0.4,2.7)	1.1 (0.6,1.7)	-30.4 (-55.1,65.7)	67.3 (17.4,115.4)	47.3 (27.2,71.6)	-29.8 (-55.2,59.7)
Fiji	3.2 (1.5,4.4)	3.1 (1.4,4.5)	-2.9 (-30.9,40.7)	3.3 (1.5,4.4)	3.1 (1.4,4.5)	-3.3 (-31.2,40.2)	142.9 (68.2,194.4)	137.1 (60.8,197.1)	-4 (-33.7,40.1)
Guam	1.2 (0.4,1.7)	0.3 (0.2,0.7)	-74.9 (-85.9,28)	1.2 (0.4,1.7)	0.3 (0.2,0.7)	-75.9 (-86.3,8.8)	52.6 (17.9,70)	13.5 (9.3,29.5)	-74.3 (-84.8,7.5)

Kiribati	1 (0.3,1.7)	0.8 (0.5,1.3)	-16.6 (-47.3,98.1)	1 (0.3,1.7)	0.8 (0.5,1.4)	-16.6 (-46.2,92.7)	50.2 (13.9,87.7)	40.9 (22.3,65.1)	-18.5 (-50.3,108.4)
Marshall Islands	1.1 (0.3,1.7)	0.9 (0.5,1.4)	-15.6 (-48.7,111.1)	1.1 (0.3,1.8)	0.9 (0.5,1.4)	-16.6 (-49,102.2)	48 (11.7,73.6)	43.4 (23,61.9)	-9.4 (-44.7,113.6)
Nauru	1.7 (0.4,3)	1.2 (0.6,2)	-28.6 (-55.2,69.8)	1.8 (0.5,3)	1.3 (0.6,2.1)	-29.7 (-56.2,62.2)	77.8 (20.9,130.6)	58.9 (30,96.5)	-24.2 (-52.1,64.4)
Niue	1.3 (0.3,2)	2.4 (1.4,3.3)	91.4 (18.9,330.6)	1.3 (0.4,2)	2.3 (1.3,3.2)	79.5 (11.1,306.4)	56.2 (15.7,86.9)	153.7 (89.3,223.3)	173.4 (64.9,482.8)
Northern Mariana Islands	0.5 (0.1,0.9)	0.1 (0.1,0.5)	-73.1 (-91,103.7)	0.5 (0.1,0.9)	0.1 (0.1,0.4)	-74.3 (-91.4,99.5)	19.6 (2.7,31.8)	5 (2.3,15.6)	-74.6 (-91.6,88)
Palau	0.6 (0.2,0.9)	0.6 (0.2,0.9)	-2.9 (-38.1,56.9)	0.6 (0.2,0.9)	0.6 (0.2,0.9)	-5 (-39.9,53.9)	31.7 (10.9,48.7)	30.4 (11.9,49.8)	-4.2 (-43.5,58.1)
Papua New Guinea	1 (0.2,1.9)	0.8 (0.3,1.2)	-23.5 (-54.5,102.1)	1 (0.2,1.9)	0.8 (0.3,1.3)	-24.3 (-55,108)	49.6 (8.7,95.5)	40.4 (16.7,65.5)	-18.4 (-51.4,125.9)
Samoa	1.7 (0.5,2.7)	1.4 (0.8,2.1)	-21.1 (-50,86.5)	1.8 (0.6,2.8)	1.4 (0.8,2.1)	-22.7 (-51.1,76)	67.7 (20.4,106.8)	52.8 (30.9,79.5)	-22.1 (-50.4,66.9)
Solomon Islands	1 (0.2,1.9)	0.9 (0.4,1.4)	-15 (-47.2,145)	1.1 (0.2,2)	0.9 (0.5,1.4)	-16.3 (-48.4,136.7)	45.7 (8.7,84.2)	41.5 (20.5,63.9)	-9.2 (-43.8,170.8)
Tokelau	1.4 (0.4,2.2)	2.6 (1.4,4.1)	82.9 (6.1,371.1)	1.5 (0.4,2.3)	2.5 (1.3,3.8)	71.5 (-0.5,340.3)	66.9 (18.5,103.9)	169.7 (86.3,275.8)	153.6 (40.1,552.3)
Tonga	0.5 (0.1,0.7)	0.4 (0.3,0.6)	-17.8 (-50.3,92.7)	0.5 (0.1,0.7)	0.4 (0.3,0.6)	-19.4 (-50.3,84.8)	22.5 (6.9,32.2)	19.1 (11.5,28.2)	-15.1 (-50,85.4)
Tuvalu	1.2 (0.3,2.1)	0.9 (0.5,1.3)	-28.6 (-55.9,85.4)	1.3 (0.3,2.2)	0.9 (0.5,1.3)	-29.9 (-56.3,78.2)	56.4 (14.5,99)	39.7 (20.8,56.4)	-29.5 (-58.4,89.1)
Vanuatu	0.9 (0.2,1.5)	0.8 (0.4,1.1)	-19.4 (-50.8,87.7)	1 (0.2,1.6)	0.8 (0.4,1.1)	-20.3 (-50.2,93.2)	40.6 (9.7,66.3)	35 (19,51.2)	-13.7 (-46.7,98.8)
North Africa and Middle East	2.2 (1.5,3.2)	2.1 (1.5,2.9)	-6.7 (-26.3,22.2)	2.3 (1.5,3.2)	2 (1.4,2.8)	-12 (-30,15)	87.3 (54.3,126.9)	69.6 (47.9,100.3)	-20.3 (-39.4,7.4)
Afghanistan	4.5 (1.3,8.8)	4.8 (2,9.2)	7.3 (-27.2,89.1)	4.6 (1.4,8.9)	4.8 (2.2,9.2)	6.1 (-27.9,82.1)	158.1 (46.9,348.8)	169 (72.6,344.7)	6.9 (-27,88.3)
Algeria	0.9 (0.5,1.3)	0.8 (0.5,1.2)	-8.5 (-42.1,35.7)	0.9 (0.4,1.3)	0.8 (0.4,1.1)	-14.2 (-46,28.2)	39.9 (21.7,61.6)	29.9 (18.4,42.8)	-25 (-52.8,18.4)
Bahrain	2.2 (1.1,3.2)	1.6 (0.9,2.2)	-28.4 (-54.4,12)	2.3 (1.1,3.3)	1.5 (0.8,2.1)	-34.9 (-58.7,2)	76.2 (41.2,111.6)	46 (29.3,66.1)	-39.6 (-61.4,-5)
Egypt	1.7 (1.1,3.4)	3.1 (1.9,4.3)	76.3 (-9,174.1)	1.8 (1.1,3.6)	3 (1.8,4.3)	70.9 (-12.4,161.4)	65 (37.8,119.4)	97.7 (59.4,137.9)	50.3 (-22.6,151.1)

Iran (Islamic Republic of)	2.5 (1.6,3.5)	2 (1.3,3)	-20.6 (-37.7,6.6)	2.5 (1.6,3.6)	1.8 (1.3,2.8)	-26.4 (-42.5,-0.7)	102.9 (60.6,143.5)	65.5 (43.5,96.8)	-36.3 (-52.5,-8.2)
Iraq	1.5 (0.7,3.4)	1.5 (0.9,3.1)	1 (-42.1,111.9)	1.5 (0.7,3.3)	1.4 (0.9,3)	-4.8 (-44.2,96.6)	60.4 (28.6,132)	54.3 (33.7,109.3)	-10.1 (-50.4,84.6)
Jordan	4.4 (2.9,5.8)	2.9 (2,4.1)	-34 (-53.7,0.1)	4.3 (2.9,5.7)	2.7 (1.8,3.9)	-38.6 (-57.3,-5.4)	163.5 (112.7,218.2)	92.7 (64.4,131.4)	-43.3 (-61.7,-11.9)
Kuwait	1.5 (1.3,1.8)	1 (0.8,1.1)	-37.7 (-51.3,-21.7)	1.4 (1.3,1.7)	0.8 (0.6,1)	-44.8 (-57.2,-29.3)	47.7 (41.4,54.7)	26.2 (21,31.5)	-45.1 (-57.1,-32)
Lebanon	2.3 (1.5,3.8)	2.1 (1.5,3)	-10.4 (-42.1,44.7)	2.3 (1.6,3.9)	1.9 (1.4,2.7)	-19.2 (-47.6,32.1)	78 (50.7,133.9)	58.3 (42,90.5)	-25.3 (-54,25)
Libya	3.4 (2.3,5.1)	3.3 (2.1,5.5)	-2.7 (-43.1,49.8)	3.4 (2.4,5.1)	3.2 (2,5.3)	-6.9 (-44.8,42.7)	118.9 (81.8,183.1)	107.8 (63.9,183.3)	-9.4 (-46.9,42.1)
Morocco	0.4 (0.2,0.5)	0.4 (0.2,0.6)	5.5 (-28.9,55.8)	0.4 (0.2,0.6)	0.4 (0.2,0.6)	1.5 (-32.1,50.5)	14 (7.6,21.9)	13.4 (8.1,20.5)	-3.7 (-36.6,47.5)
Palestine	0.7 (0.4,1.1)	0.6 (0.4,1.1)	-8.2 (-43.3,62.7)	0.6 (0.4,1.1)	0.6 (0.3,1)	-13 (-45.3,52)	26.2 (14.7,45.2)	21 (12.8,35.4)	-19.8 (-50.7,41.6)
Oman	1.2 (0.6,1.9)	1.1 (0.7,1.6)	-11.8 (-45.8,57.6)	1.2 (0.6,1.9)	1 (0.6,1.4)	-19.3 (-50.6,48.3)	45.5 (22.3,71.8)	33.7 (22.8,46.6)	-25.9 (-54.4,30.8)
Qatar	2.5 (1.2,3.6)	1.6 (0.9,2.2)	-36.2 (-57.9,-3.1)	2.5 (1.2,3.7)	1.4 (0.8,2)	-44.3 (-63.8,-13.8)	81.3 (45.1,119.7)	40.1 (25.3,56.4)	-50.6 (-68.3,-23.4)
Saudi Arabia	0.9 (0.5,1.9)	1.5 (0.9,2.6)	64.5 (-0.4,171.1)	0.9 (0.5,1.9)	1.3 (0.9,2.3)	49.3 (-7.7,144.2)	34.8 (20.2,71)	46.9 (29.7,84.4)	34.8 (-17.5,130.1)
Sudan	2.5 (0.9,4.9)	2.5 (1.3,4)	1.2 (-42.1,126.2)	2.5 (0.9,4.9)	2.5 (1.3,3.9)	-2 (-43.4,115.6)	95.8 (31.9,207.5)	90.9 (47,152.4)	-5.1 (-51.8,120.9)
Syrian Arab Republic	3.6 (1.9,5.2)	2.9 (2,4.6)	-19.3 (-45,26.8)	3.6 (1.9,5.3)	2.7 (1.9,4.3)	-24 (-48.8,19)	131.6 (72.1,200.9)	89.6 (60.8,140.3)	-31.9 (-53.7,12.7)
Tunisia	1.4 (0.9,2.1)	1.3 (0.8,2)	-12 (-42,30.1)	1.4 (0.9,2.1)	1.2 (0.7,1.9)	-19.3 (-46.3,17.6)	52.6 (35.8,81.6)	39.9 (25.8,63.3)	-24.1 (-50.2,14.6)
Turkey	3.5 (2.3,4.7)	2.4 (1.6,3.2)	-32.6 (-53.7,4.9)	3.5 (2.2,4.8)	2.2 (1.6,2.9)	-37.9 (-58,-3.6)	143.5 (84.4,206.8)	73.9 (51.6,98.2)	-48.5 (-66.2,-14.6)
United Arab Emirates	4.7 (2.4,6.7)	5.6 (3,8.4)	19.9 (-28,112.2)	4.7 (2.4,6.9)	5.7 (3,8.6)	21.4 (-30.7,125.3)	158.5 (92,229.1)	141.3 (89.1,204.7)	-10.8 (-41.2,44.1)
Yemen	2.2 (0.8,3.6)	2.3 (1,3.5)	6 (-32.4,86.6)	2.2 (0.8,3.8)	2.3 (1,3.6)	4.2 (-33.2,82.8)	76.3 (29.4,140.7)	78.8 (35,125.4)	3.2 (-36.4,87.3)
South Asia	0.8 (0.5,1.3)	0.9 (0.6,1.3)	5.8 (-25.3,67.2)	0.8 (0.5,1.3)	0.9 (0.6,1.3)	3.4 (-26.5,62.7)	36.7 (19.5,60.2)	35.9 (25.4,52)	-1.9 (-37.7,64.2)

Bangladesh	0.9 (0.5,1.4)	0.8 (0.5,1.3)	-9 (-50.5,63)	0.9 (0.5,1.5)	0.8 (0.5,1.3)	-12.1 (-51.6,58.5)	42.7 (18.5,76.7)	36.1 (22,56.6)	-15.5 (-60,73)
Bhutan	0.9 (0.4,1.7)	1 (0.6,1.6)	6.7 (-47.9,120.7)	0.9 (0.4,1.8)	1 (0.6,1.7)	4.1 (-48.9,116.1)	39.9 (14.5,80.3)	38.7 (23.1,70.6)	-3 (-60.5,137.3)
India	0.8 (0.5,1.2)	0.8 (0.6,1.2)	4.9 (-27.3,70.3)	0.8 (0.5,1.3)	0.8 (0.6,1.2)	2.7 (-28.6,67.5)	34.5 (18.8,57.4)	32.5 (22,45.6)	-5.9 (-42.5,63.9)
Nepal	0.9 (0.4,1.5)	0.9 (0.6,1.4)	1.6 (-36.6,73.2)	0.9 (0.5,1.6)	0.9 (0.6,1.5)	-0.4 (-38.1,70.8)	39.9 (16.8,76.8)	36.3 (23.8,58)	-9.2 (-51.9,85.1)
Pakistan	1 (0.6,1.7)	1.2 (0.7,2.2)	18.8 (-20.8,85)	1.1 (0.6,1.8)	1.2 (0.8,2.2)	17 (-21.6,81)	45 (24.3,78.8)	54.2 (32.7,95.6)	20.5 (-21.1,88)
Southern Sub-Saharan Africa	0.9 (0.6,1.3)	1 (0.5,1.5)	4.1 (-23.1,32.7)	0.9 (0.6,1.3)	1 (0.5,1.5)	3.4 (-25.3,34.5)	37.6 (24.7,52)	36.1 (20.9,53.8)	-4.1 (-27.4,22.2)
Botswana	0.8 (0.4,1.3)	0.8 (0.4,1.2)	-3.5 (-40.8,60.4)	0.8 (0.4,1.3)	0.8 (0.4,1.3)	-4.8 (-42,56.4)	29.3 (16.7,48.1)	28.6 (14.6,44.2)	-2.5 (-40.6,63.4)
Lesotho	0.6 (0.4,1.1)	1 (0.5,2.2)	65.3 (-23.8,181.5)	0.7 (0.4,1.1)	1.1 (0.5,2.2)	63.7 (-24.4,178.3)	23.3 (14,38.8)	38.3 (19.1,76.6)	64.1 (-16.9,169.2)
Namibia	0.6 (0.4,1)	0.6 (0.3,1.1)	-7.4 (-42.6,43.8)	0.7 (0.4,1)	0.6 (0.3,1.1)	-9.2 (-43.9,40)	25.8 (15.6,38)	23.5 (13.6,38.8)	-8.8 (-44.1,47.6)
South Africa	1 (0.6,1.4)	0.9 (0.5,1.4)	-4.4 (-29.9,23.4)	1 (0.6,1.4)	0.9 (0.5,1.5)	-4.8 (-33,24.3)	40.3 (25.3,55.5)	33.8 (17.7,49)	-16.1 (-41.5,10.6)
Eswatini	0.9 (0.6,1.6)	1 (0.5,2)	11.8 (-33.6,76.9)	0.9 (0.6,1.7)	1 (0.5,2)	10.4 (-33.8,77.5)	34.2 (21,60.3)	37.7 (19.1,71.6)	10.1 (-34.6,75.5)
Zimbabwe	0.9 (0.6,1.3)	1.2 (0.7,2.1)	37.6 (-20.2,118.4)	0.9 (0.6,1.4)	1.2 (0.7,2.1)	35.7 (-21.2,113.8)	32.7 (21.3,47.9)	47.1 (25.8,76.3)	44.2 (-10.6,125.3)
Western Sub-Saharan Africa	0.3 (0.2,0.4)	0.3 (0.2,0.4)	-2.5 (-27.1,26.5)	0.3 (0.2,0.4)	0.3 (0.2,0.4)	-2.8 (-27.2,25.8)	13.2 (7,19.5)	12 (7.5,16.8)	-9 (-36.1,26.1)
Benin	0.3 (0.2,0.5)	0.4 (0.2,0.5)	12 (-31.4,67.8)	0.3 (0.2,0.5)	0.4 (0.2,0.5)	11.3 (-31.2,67.5)	15.4 (8.1,25.7)	16.4 (8.3,23.5)	6.4 (-39.5,67.7)
Burkina Faso	0.3 (0.2,0.5)	0.4 (0.2,0.6)	15.6 (-24.1,71.7)	0.3 (0.2,0.5)	0.4 (0.2,0.6)	15.2 (-23.9,72.4)	15.2 (7.3,25.3)	17.4 (8,26)	14.6 (-28.8,77)
Cameroon	0.4 (0.2,0.6)	0.4 (0.2,0.6)	7.3 (-35.1,70.2)	0.4 (0.2,0.6)	0.4 (0.2,0.7)	6.4 (-35.5,69.7)	17.2 (9.2,27.9)	17.9 (8.5,27)	3.8 (-39.1,62.3)
Cabo Verde	0.7 (0.5,1.4)	0.9 (0.5,1.5)	17.1 (-28.2,79.1)	0.7 (0.5,1.4)	0.8 (0.5,1.5)	15 (-29.4,75.6)	35.9 (23.3,62.3)	35.1 (21.1,62.5)	-2 (-43,59.3)
Chad	0.3 (0.1,0.4)	0.4 (0.2,0.6)	37.8 (-8.6,107.8)	0.3 (0.2,0.4)	0.4 (0.2,0.6)	38.1 (-7.6,110.1)	11.7 (6.2,18.5)	15.4 (7.4,23.6)	31.6 (-14.7,99.1)

CÃ´te d'Ivoire	0.2 (0.1,0.3)	0.2 (0.1,0.3)	-5.2 (-34.8,37.5)	0.2 (0.2,0.4)	0.2 (0.1,0.3)	-6.8 (-36.6,37)	10.7 (6.3,15.8)	9.8 (5.7,14.6)	-8.6 (-38.9,36.4)
Gambia	0.2 (0.1,0.2)	0.2 (0.1,0.3)	4 (-35,60.8)	0.2 (0.1,0.2)	0.2 (0.1,0.3)	2.8 (-35.4,58.3)	7.4 (3.8,12)	7.1 (4.3,12)	-3.7 (-43.8,61.9)
Ghana	0.6 (0.3,0.8)	0.3 (0.2,0.7)	-46 (-70.6,37.8)	0.6 (0.3,0.9)	0.3 (0.2,0.7)	-46.5 (-71.1,35.3)	27.2 (12.2,37.8)	13.7 (7.6,26.6)	-49.7 (-72.8,30.9)
Guinea	0.1 (0.1,0.2)	0.1 (0.1,0.2)	-12.5 (-47.7,36.3)	0.1 (0.1,0.2)	0.1 (0.1,0.2)	-12.7 (-48.4,35.2)	6.8 (3,12.5)	5.4 (2.8,9)	-20.3 (-57.5,31.2)
Guinea-Bissau	0.4 (0.2,0.6)	0.5 (0.3,0.7)	17.8 (-24.7,81.7)	0.4 (0.2,0.6)	0.5 (0.3,0.7)	17.7 (-23.8,83.4)	18 (8.7,31.8)	19.3 (10.8,29.6)	7.1 (-33.4,72.2)
Liberia	0.3 (0.2,0.6)	0.4 (0.2,0.6)	18.6 (-30.2,87.5)	0.4 (0.2,0.6)	0.4 (0.2,0.7)	16.7 (-31,86.5)	15.9 (6.9,31.5)	17.2 (8.1,26.2)	8.1 (-41.8,83.7)
Mali	0.4 (0.2,0.6)	0.3 (0.2,0.5)	-17.9 (-46.5,31.8)	0.4 (0.2,0.6)	0.3 (0.2,0.5)	-18.1 (-47.1,30)	17.8 (7.3,31.4)	13.3 (7.5,21.4)	-25.6 (-54.6,31.5)
Mauritania	0.4 (0.2,0.6)	0.4 (0.2,0.6)	12.7 (-29.7,72.6)	0.4 (0.2,0.6)	0.4 (0.2,0.6)	9.9 (-31.4,69.7)	14.6 (8.2,24.1)	15.4 (8.5,23.1)	5.2 (-35.4,70.4)
Niger	0.3 (0.2,0.5)	0.3 (0.2,0.5)	4.6 (-37.4,61.4)	0.3 (0.2,0.5)	0.3 (0.2,0.5)	4.9 (-37.1,62.3)	15.6 (6.8,29.2)	14 (6.3,21.7)	-9.8 (-50.8,52.3)
Nigeria	0.2 (0.1,0.3)	0.2 (0.1,0.3)	0.2 (-35.7,51.1)	0.2 (0.1,0.3)	0.2 (0.1,0.3)	-0.4 (-35.5,50.8)	9.8 (5.4,16)	9.4 (5.6,13.9)	-4.7 (-42.6,45.9)
Sao Tome and Principe	0.3 (0.2,0.5)	0.3 (0.2,0.5)	-5.2 (-37.5,48.5)	0.4 (0.2,0.5)	0.3 (0.2,0.5)	-7.1 (-38.9,43.5)	16.9 (9.9,24.2)	13.9 (8.4,23.9)	-17.8 (-50.2,48.5)
Senegal	0.3 (0.2,0.5)	0.4 (0.2,0.6)	19.9 (-21.4,83.2)	0.3 (0.2,0.5)	0.4 (0.2,0.6)	19 (-22.1,83.7)	15.3 (8.2,22.7)	16.5 (9.6,24.2)	8.2 (-34.6,72.1)
Sierra Leone	0.3 (0.2,0.4)	0.4 (0.2,0.5)	29.1 (-18.8,101.8)	0.3 (0.2,0.4)	0.4 (0.2,0.6)	27.6 (-19.9,100)	14.2 (6.5,25.5)	16.5 (8,23.6)	16.7 (-31.7,93.2)
Togo	0.3 (0.2,0.4)	0.4 (0.2,0.6)	22.2 (-21.2,85.5)	0.3 (0.2,0.5)	0.4 (0.2,0.6)	21.4 (-21.4,85.1)	14 (7.8,20.6)	15.7 (8.3,22.6)	12.3 (-31.7,74)
Eastern Sub-Saharan Africa	0.5 (0.2,0.8)	0.5 (0.3,0.7)	-8.7 (-44.7,70.1)	0.5 (0.2,0.9)	0.5 (0.3,0.7)	-9.4 (-45,65.2)	27.6 (11,48.8)	23.1 (12.1,35.3)	-16.3 (-56.1,99.4)
Burundi	0.4 (0.2,0.8)	0.4 (0.2,0.5)	-16.2 (-55.9,54.2)	0.4 (0.2,0.8)	0.4 (0.2,0.6)	-16.5 (-57.1,55.8)	23.8 (7.8,43.2)	17.8 (7.1,27.2)	-25.5 (-65.2,50.3)
Comoros	0.5 (0.2,0.7)	0.5 (0.3,0.8)	9.2 (-36.7,80.2)	0.5 (0.2,0.7)	0.5 (0.3,0.8)	8.4 (-37.6,77.5)	24.2 (9.3,36.5)	24.9 (12.4,40.2)	3 (-43.7,100.2)
Djibouti	0.4 (0.2,0.6)	0.4 (0.2,0.7)	3.4 (-34.3,60.5)	0.4 (0.2,0.6)	0.4 (0.2,0.7)	3.1 (-34.1,60.2)	20.4 (7.9,32)	18.1 (7.9,31.6)	-11.2 (-44.3,47.5)

Eritrea	0.4 (0.2,0.6)	0.4 (0.2,0.7)	18.6 (-34.9,108.2)	0.4 (0.2,0.6)	0.5 (0.2,0.7)	18.4 (-35.8,103.9)	19 (7.4,30.3)	21.3 (9.3,35.8)	12.3 (-46.2,132.2)
Ethiopia	0.9 (0.3,1.8)	0.7 (0.4,1.2)	-23.1 (-62.7,106.6)	0.9 (0.3,1.8)	0.7 (0.4,1.2)	-24.3 (-63.3,102.6)	43.4 (14.2,102.6)	31 (16.3,53.2)	-28.5 (-68.9,154.7)
Kenya	0.4 (0.2,0.5)	0.5 (0.3,0.7)	28.9 (-13.3,90.6)	0.4 (0.2,0.5)	0.5 (0.3,0.7)	27.9 (-13.1,88.6)	15.7 (9,24.3)	18.9 (11.5,29.9)	20.6 (-26.7,89.2)
Madagascar	0.4 (0.2,0.5)	0.4 (0.2,0.5)	-7.5 (-37.8,35.5)	0.4 (0.2,0.6)	0.4 (0.2,0.5)	-7.9 (-38.7,35.6)	21 (9,30.8)	17.7 (7.9,26.9)	-15.8 (-48.5,35.9)
Malawi	0.2 (0.1,0.3)	0.1 (0.1,0.2)	-24.1 (-56.5,50.2)	0.2 (0.1,0.3)	0.1 (0.1,0.2)	-24.7 (-56.7,48.1)	10.6 (2.8,18)	6.9 (2.6,12.6)	-35.4 (-68.1,64.8)
Mozambique	0.7 (0.3,1.1)	0.6 (0.3,1)	-15 (-52.9,58.4)	0.7 (0.3,1.1)	0.6 (0.3,1)	-14.9 (-53.3,61.5)	42.3 (13.4,71)	31.6 (13.3,59.9)	-25.2 (-66.2,98.5)
Rwanda	0.6 (0.2,1)	0.5 (0.2,0.7)	-19.2 (-59,64.7)	0.6 (0.2,1)	0.5 (0.2,0.7)	-19.9 (-59.4,65.4)	30.2 (11.5,53.1)	21.8 (10.7,34.6)	-27.7 (-66.8,85.4)
Somalia	0.4 (0.1,0.6)	0.4 (0.1,0.6)	-1.7 (-32.8,51.8)	0.4 (0.1,0.7)	0.4 (0.1,0.6)	-1.1 (-31.8,50.9)	18 (5.9,33.3)	15.7 (6.4,25.6)	-12.7 (-44.8,47.6)
South Sudan	0.4 (0.2,0.7)	0.4 (0.2,0.8)	8.3 (-29.7,58.6)	0.4 (0.2,0.7)	0.4 (0.2,0.8)	7.9 (-29.7,60)	21.4 (8.1,40.1)	20.6 (8.7,39.9)	-3.7 (-38.4,46.2)
United Republic of Tanzania	0.5 (0.2,0.7)	0.5 (0.2,0.7)	1.7 (-36,60.3)	0.5 (0.2,0.7)	0.5 (0.2,0.7)	0.8 (-36.2,57.9)	25.6 (11.5,39.7)	24.2 (11.7,38)	-5.6 (-46.4,80.1)
Uganda	0.2 (0.1,0.4)	0.3 (0.2,0.5)	22.3 (-21.8,106.6)	0.2 (0.1,0.4)	0.3 (0.2,0.5)	20.8 (-23.3,99.4)	13.6 (7.1,20.6)	15.5 (7.9,25.1)	14.2 (-32,122.5)
Zambia	0.5 (0.2,0.8)	0.5 (0.3,0.9)	8.6 (-45.6,105.9)	0.5 (0.2,0.8)	0.5 (0.3,0.9)	7.4 (-46.5,106.7)	27.5 (11.5,43.9)	26.3 (12.2,43)	-4.5 (-56.7,106.6)
Central Sub-Saharan Africa	0.5 (0.3,0.8)	0.5 (0.3,0.8)	-5 (-39.9,43)	0.5 (0.3,0.8)	0.5 (0.3,0.8)	-5.7 (-41,43.7)	23.4 (10.6,34.5)	20.6 (11,31.2)	-12 (-48.1,42.7)
Angola	0.5 (0.3,0.8)	0.5 (0.3,0.8)	-3.4 (-45.4,71.4)	0.5 (0.3,0.8)	0.5 (0.3,0.8)	-4.4 (-46.5,69.4)	24 (9.2,39.4)	21.4 (10.7,33.6)	-10.6 (-53.4,76)
Central African Republic	0.6 (0.3,0.9)	0.5 (0.3,0.8)	-10.1 (-42.7,33)	0.6 (0.3,0.9)	0.5 (0.3,0.9)	-10 (-42.8,34)	25.5 (10.3,40.2)	22.1 (10.7,33.9)	-13.4 (-46.4,31.6)
Congo	0.7 (0.3,1)	0.6 (0.3,1)	-2.4 (-39.5,53.4)	0.7 (0.3,1)	0.6 (0.4,1)	-4.3 (-41.4,47.9)	27.3 (12.3,42.7)	25.7 (13.8,39.1)	-6 (-44.4,56.1)
Democratic Republic of the Congo	0.5 (0.3,0.8)	0.5 (0.2,0.8)	-5.4 (-43.4,51.4)	0.5 (0.3,0.8)	0.5 (0.3,0.8)	-5.7 (-43.6,50)	22.8 (10.5,34.2)	19.7 (10.4,31.4)	-13.3 (-52.5,47.4)

Equatorial Guinea	0.5 (0.3,0.8)	0.5 (0.3,0.9)	0.6 (-43,88.4)	0.6 (0.3,0.8)	0.5 (0.3,0.9)	-2.8 (-45.5,83.4)	23.6 (10.1,35.9)	22 (10.6,38.2)	-6.7 (-54.9,85.2)
Gabon	0.6 (0.3,0.8)	0.6 (0.3,0.9)	-3.2 (-41.6,52.6)	0.6 (0.3,0.8)	0.6 (0.3,0.9)	-5.2 (-43.9,47.4)	24.3 (12.8,34.7)	21.9 (11.1,34.3)	-9.7 (-50.3,46.3)

Table S8 Age-standardized incidence rates, death rates, and DALY rates in 1990 and 2021, and the percentage change in the age-standardized rates for CLL among WCBA (15–49 years), by country.

	Incidence (95% UI)			Deaths (95% UI)			DALYs (95% UI)		
	ASRs per 100000 (95% UI), 1990	ASRs per 100000 (95% UI), 2021	Percentage changes in ASRs from 1990 to 2021	ASRs per 100000 (95% UI), 1990	ASRs per 100000 (95% UI), 2021	Percentage changes in ASRs from 1990 to 2021	ASRs per 100000 (95% UI), 1990	ASRs per 100000 (95% UI), 2021	Percentage changes in ASRs from 1990 to 2021
Global	1.2 (1.1,1.3)	1.1 (0.8,1.2)	-13.8 (-27.6,-1.9)	0.6 (0.5,0.7)	0.4 (0.3,0.4)	-37.3 (-45.3,-29.3)	13.3 (10.6,15.6)	8.2 (5.6,9.5)	-38.4 (-49.2,-25.8)
High-income North America	3.5 (3.2,3.7)	2.2 (1.9,2.4)	-37.7 (-41.2,-34.4)	1.2 (1,1.2)	0.7 (0.6,0.7)	-42.7 (-46.7,-39.9)	23.1 (21.2,24.6)	11.6 (10.1,12.6)	-50 (-53.1,-47.6)
Canada	3.7 (3.2,4.1)	2.6 (2.1,3.1)	-29.8 (-43.1,-14.6)	1.2 (1,1.3)	0.7 (0.5,0.8)	-43.7 (-52.4,-35)	23.2 (20.7,25.7)	11.8 (9.7,13.8)	-49.2 (-57.2,-39.9)
Greenland	0 (0,0)	0 (0,0)	40.2 (-40.8,169.4)	0 (0,0)	0 (0,0)	15.8 (-54.5,125.8)	0 (0,0)	0 (0,0)	-1.6 (-59.1,89.7)
United States of America	3.5 (3.2,3.7)	2.2 (1.9,2.3)	-38.6 (-42.3,-35.6)	1.2 (1,1.2)	0.7 (0.5,0.7)	-42.6 (-46.7,-39.9)	23.1 (21.2,24.6)	11.5 (10,12.5)	-50.2 (-53.2,-47.9)
Australasia	2.5 (2.2,2.8)	2.2 (1.7,2.6)	-12.6 (-32.1,8.5)	0.8 (0.7,0.9)	0.5 (0.4,0.7)	-34.8 (-48.6,-19.4)	16.3 (14.4,18.1)	9.8 (7.6,12)	-39.8 (-52.9,-26.7)
Australia	2.4 (2.1,2.7)	2.1 (1.6,2.6)	-12.5 (-34.4,10.4)	0.8 (0.7,0.9)	0.5 (0.4,0.7)	-35.5 (-50.5,-18.3)	15.9 (14,18)	9.4 (7.1,11.8)	-41.1 (-54.5,-27.4)
New Zealand	2.8 (2.4,3.2)	2.4 (1.9,2.9)	-12.3 (-32.2,7.8)	0.9 (0.8,1.1)	0.6 (0.5,0.8)	-30.5 (-44.9,-14.5)	18 (15.5,20.7)	12 (9.6,14.5)	-33.2 (-47,-18.3)
High-income Asia Pacific	0.2 (0.2,0.2)	0.2 (0.1,0.2)	-4.2 (-19.5,12.1)	0.1 (0.1,0.1)	0 (0,0.1)	-39.2 (-49.3,-31)	1.5 (1.3,1.8)	0.9 (0.7,1)	-42.4 (-50.3,-34.8)
Brunei Darussalam	0.5 (0.2,0.8)	0.5 (0.2,0.9)	6.5 (-52.4,140.8)	0.4 (0.2,0.6)	0.3 (0.1,0.5)	-18 (-60.2,78.3)	8.3 (3.5,13.6)	6.5 (2.1,10.6)	-21.4 (-64.2,77.1)
Japan	0.2 (0.2,0.2)	0.2 (0.2,0.2)	-7.6 (-18.9,4.1)	0.1 (0.1,0.1)	0 (0,0)	-36.2 (-44.9,-30.9)	1.4 (1.3,1.5)	0.9 (0.7,1)	-39 (-45.2,-34.2)
Singapore	0.2 (0.2,0.3)	0.2 (0.2,0.3)	-0.1 (-20.6,25.9)	0.1 (0.1,0.1)	0.1 (0,0.1)	-55.9 (-64.7,-45.6)	2.6 (2.2,3)	1.2 (1,1.4)	-54.7 (-63.1,-44.3)
Republic of Korea	0.1 (0.1,0.3)	0.2 (0.1,0.3)	23.6 (-30.7,104.9)	0.1 (0.1,0.2)	0 (0,0.1)	-53.2 (-73.5,-20.8)	2 (1,3.5)	0.9 (0.4,1.5)	-55.2 (-74.7,-25.7)
Western Europe	2.5 (2.3,2.6)	2.3 (2,2.6)	-6.1 (-14.5,3)	1 (0.9,1.1)	0.7 (0.5,0.7)	-34.1 (-41.6,-27.2)	19.3 (17.7,20.7)	11.6 (9.9,12.9)	-40 (-45.7,-33.7)

Andorra	1.5 (0.6,2.5)	1.4 (0.7,2.3)	-3.5 (-49.1,70.7)	0.5 (0.2,0.9)	0.3 (0.2,0.6)	-37.5 (-63.6,6.8)	10.3 (4.5,17.3)	6.6 (3.1,11.1)	-36.5 (-64.3,12.8)
Austria	2.6 (2.3,2.9)	2.9 (2.2,3.5)	11.7 (-13.9,39)	1.1 (1,1.2)	0.8 (0.6,1)	-29.3 (-43.9,-13.7)	21.2 (18.8,23.7)	14.3 (11.1,17.3)	-32.6 (-46.8,-17.9)
Belgium	2.3 (1.9,2.7)	2.2 (1.7,2.8)	-3.5 (-28.1,26.7)	1 (0.8,1.2)	0.6 (0.5,0.8)	-36.4 (-51.6,-18.3)	18.7 (15.7,22.3)	11.6 (8.9,14.4)	-38.1 (-52.8,-19.9)
Cyprus	2.6 (1.5,4.7)	3.5 (1.7,5.1)	33.6 (-23.9,162.4)	1.7 (1,3.3)	1.1 (0.6,1.5)	-34.8 (-66.1,25.8)	27.5 (15.8,51.2)	18.4 (9.2,25.7)	-33.2 (-63,26.1)
Denmark	3.3 (2.8,3.8)	3.8 (3,4.6)	14.3 (-12.5,42.9)	1.3 (1.2,1.5)	1 (0.8,1.2)	-24.9 (-40.4,-6.1)	26.7 (23.5,30.7)	18.3 (14.8,22.2)	-31.5 (-46,-15)
Finland	1.8 (1.6,2)	1.8 (1.5,2.2)	2 (-17.4,25.8)	0.9 (0.8,1)	0.6 (0.4,0.6)	-39.2 (-50.2,-26.1)	17.9 (15.9,20.2)	10.3 (8.4,12.1)	-42.3 (-52.5,-29.5)
France	2.5 (2.2,2.8)	2.6 (2,3.2)	3.2 (-18.7,27.5)	1.2 (1,1.3)	0.7 (0.6,0.9)	-37.1 (-49.7,-23.8)	22 (19.4,24.6)	12.9 (10.1,16.2)	-41 (-53.1,-27.6)
Germany	2.2 (1.8,2.7)	2.2 (1.8,2.7)	-0.9 (-25,30.5)	1 (0.8,1.2)	0.7 (0.5,0.8)	-33 (-47.6,-12)	19.8 (15.8,23.1)	11.6 (9.4,14)	-41.5 (-54.2,-23.6)
Greece	2.4 (2.1,2.7)	2.3 (1.9,2.6)	-5.8 (-20.6,15.2)	0.9 (0.8,1)	0.8 (0.6,0.9)	-18.3 (-29.9,-4.4)	17.3 (15.3,19.1)	13.1 (11.2,15.1)	-24.7 (-35.4,-11.5)
Iceland	2.4 (2,2.9)	2.4 (1.9,3)	1.3 (-23,35.5)	0.8 (0.6,0.9)	0.6 (0.4,0.7)	-27.9 (-44.9,-1.1)	15.8 (12.9,18.7)	10.7 (8.5,13.4)	-32.7 (-47.8,-11)
Ireland	2.6 (2.3,3)	2.2 (1.7,2.7)	-14.8 (-35.5,8)	1.2 (1,1.3)	0.5 (0.4,0.6)	-55.6 (-64.7,-44.9)	22.2 (19.5,25)	10.1 (7.9,12.6)	-54.4 (-64.2,-44)
Israel	3.3 (2.8,3.9)	3.4 (2.8,4.2)	3.3 (-21.1,31.5)	1.8 (1.4,2.1)	1.2 (0.9,1.4)	-32.7 (-48.4,-16.9)	32.7 (27.3,38.1)	20.3 (16.3,24)	-38 (-50.7,-22.8)
Italy	2.9 (2.6,3.2)	2.7 (2.3,3.1)	-6 (-18.7,7)	1.1 (0.9,1.1)	0.7 (0.6,0.8)	-33.4 (-41.8,-26.5)	21.7 (19.9,23.2)	12.9 (10.8,14.7)	-40.6 (-47.4,-34.7)
Luxembourg	3 (2.7,3.4)	3.2 (2.6,3.8)	4.5 (-16.1,29.9)	1.5 (1.3,1.6)	0.9 (0.7,1)	-40.3 (-49.4,-29.8)	27.5 (24.5,30.8)	15.8 (13.2,18.6)	-42.5 (-52.3,-30.4)
Malta	1.2 (1,1.5)	1.2 (0.9,1.5)	0.6 (-27.4,36.7)	0.6 (0.5,0.7)	0.3 (0.3,0.4)	-41.5 (-56.8,-21.9)	10.9 (9,13.2)	6.4 (4.9,8)	-41.1 (-56.4,-20.6)
Monaco	4.2 (2,7.4)	5.1 (1.2,9.6)	21.8 (-62,132.9)	1.5 (0.7,2.5)	1.3 (0.4,2.4)	-9.8 (-70.6,65.4)	28.3 (13.8,48.1)	25.3 (6.4,45.3)	-10.6 (-71.2,67)
Netherlands	2.5 (2.1,2.9)	1.8 (1.4,2.2)	-28.3 (-44.2,-7.1)	1 (0.8,1.1)	0.4 (0.3,0.5)	-58.3 (-67.1,-48.2)	17.8 (15.2,20.1)	8.1 (6.2,10)	-54.7 (-64.7,-42.9)
Norway	1.7 (1.5,1.9)	2.2 (1.9,2.6)	29.8 (8.8,52.5)	0.7 (0.6,0.8)	0.6 (0.5,0.7)	-13.1 (-24.1,-0.5)	13.8 (12.5,14.9)	10.9 (9.2,12.4)	-20.7 (-31.1,-9.8)

Portugal	1.5 (1.3,1.7)	1.8 (1.4,2.2)	21.3 (-5.9,54.2)	0.9 (0.8,1)	0.6 (0.4,0.7)	-33.1 (-48.5,-16.6)	16.5 (14.3,18.8)	10.3 (8,12.7)	-37.5 (-52,-22.4)
San Marino	2.9 (1.3,5)	2 (0.8,3.5)	-31.3 (-67,37.5)	1 (0.4,1.7)	0.5 (0.2,0.8)	-50.7 (-75.8,-1.8)	18.4 (7.6,31)	9.5 (3.7,16)	-48.4 (-75.4,2.3)
Spain	2.1 (1.8,2.4)	2.1 (1.6,2.6)	-2 (-22.7,23.1)	0.9 (0.8,1)	0.5 (0.4,0.7)	-39.7 (-51.9,-26)	17.6 (15.5,19.6)	9.8 (7.7,11.9)	-44.3 (-54.9,-31)
Sweden	2.6 (2.3,3)	2.1 (1.7,2.6)	-20.1 (-39.7,2.1)	1 (0.9,1.1)	0.6 (0.5,0.8)	-39.4 (-52.2,-24.4)	18.3 (15.9,21)	10.2 (8.2,12.5)	-44.4 (-57,-30)
Switzerland	2.5 (2,3.2)	2.4 (1.8,3)	-5.6 (-31.8,29.3)	0.8 (0.7,1)	0.6 (0.4,0.7)	-30.4 (-46.8,-7.6)	17 (13.6,20.4)	10.8 (8.4,13.7)	-36.5 (-52.2,-13.6)
United Kingdom	2.8 (2.6,2.9)	2.1 (1.9,2.2)	-26.1 (-31.6,-19.5)	0.7 (0.7,0.8)	0.5 (0.4,0.6)	-29 (-34.3,-23.3)	14.6 (13.4,15.6)	9.3 (8.2,10.2)	-36.4 (-40.7,-30.8)
Southern Latin America	0.7 (0.6,0.9)	0.6 (0.5,0.7)	-18.9 (-35.4,3.4)	0.6 (0.5,0.7)	0.3 (0.3,0.4)	-41.5 (-52.7,-26.7)	10.9 (9,12.7)	5.9 (5,7)	-45.5 (-56.6,-30.8)
Argentina	0.7 (0.5,0.8)	0.5 (0.4,0.6)	-30.7 (-47.8,-5.5)	0.5 (0.4,0.7)	0.3 (0.2,0.4)	-46 (-59,-28.3)	10.3 (8.1,12.5)	5.2 (4.2,6.2)	-49.5 (-62.3,-31.9)
Chile	0.6 (0.5,0.7)	0.6 (0.5,0.7)	-3.3 (-27.9,28.3)	0.5 (0.4,0.6)	0.3 (0.2,0.3)	-42.6 (-57.2,-23.3)	8.9 (7.4,10.6)	4.8 (3.8,6)	-46.2 (-59.7,-28.3)
Uruguay	1.5 (1.3,1.8)	1.9 (1.5,2.2)	23.3 (-3.6,61.2)	1.1 (0.9,1.3)	1 (0.8,1.2)	-7 (-26.7,17.8)	21.2 (18,24.9)	18.4 (15.2,22.3)	-13 (-31.9,11.9)
Eastern Europe	1.1 (1,1.3)	1.7 (1.5,1.9)	51.3 (27.7,87.5)	0.6 (0.5,0.7)	0.6 (0.6,0.7)	7.6 (-8.9,31.1)	15.2 (12.8,17.3)	14.6 (12.9,16.8)	-3.6 (-19.7,19.7)
Belarus	1.7 (1.2,2.2)	2.9 (2.1,4)	71.8 (8.6,176.4)	0.9 (0.7,1.2)	0.9 (0.7,1.2)	1.5 (-32.5,53.8)	22 (16.4,28.7)	21.9 (15.5,29.7)	-0.5 (-36.4,57.6)
Estonia	3.7 (3.1,4.5)	3.6 (2.8,4.5)	-2.2 (-29.1,31.7)	1.9 (1.6,2.3)	1.1 (0.9,1.4)	-40.8 (-54.1,-23.6)	46.5 (38.3,55.9)	24.4 (19,29.9)	-47.5 (-60.2,-31.3)
Latvia	2.5 (1.9,3.1)	3.4 (2.6,4.3)	35.7 (-1.2,97.4)	1.3 (1,1.7)	1.3 (1,1.6)	-5.7 (-30.3,31.7)	32 (24.2,39.8)	28.8 (21.9,36.4)	-10.3 (-34.9,28)
Lithuania	3.2 (2.6,3.9)	2.7 (2.1,3.3)	-17.8 (-37.1,6.4)	1.6 (1.3,1.9)	1.1 (0.8,1.3)	-34.2 (-49.5,-15.9)	39.3 (32.3,46.7)	22.9 (18.3,28.2)	-41.8 (-54.8,-25.8)
Republic of Moldova	0.5 (0.5,0.6)	0.6 (0.5,0.7)	12.9 (-12.2,50.1)	0.3 (0.3,0.4)	0.3 (0.2,0.3)	-25.5 (-41.5,-4.1)	8.4 (7,9.7)	6.2 (5.2,7.4)	-26.4 (-42.3,-4.5)
Russian Federation	1 (0.8,1.1)	1.7 (1.5,1.9)	77.4 (49.7,114)	0.5 (0.5,0.6)	0.6 (0.6,0.7)	22.8 (4.7,47)	13.4 (11.5,15.7)	14.3 (12.8,16)	6.3 (-10.9,28.7)
Ukraine	1.2 (0.9,1.6)	1.3 (0.8,2)	8 (-39.6,84.9)	0.6 (0.4,0.8)	0.5 (0.3,0.8)	-12.1 (-50.6,46.4)	15.7 (11.5,20.5)	13.4 (8,20.5)	-14.6 (-53.7,48.6)

Central Europe	1.2 (1.1,1.4)	2.3 (2,2.5)	90.5 (65.8,121)	0.7 (0.7,0.8)	0.8 (0.7,0.9)	8.8 (-4.4,24.6)	15.7 (14,17.6)	16.4 (14.3,18.1)	5 (-8.1,20.1)
Albania	0 (0,0)	0 (0,0)	50.9 (-35.2,287.3)	0 (0,0)	0 (0,0)	-3.2 (-60.4,155.3)	0 (0,0)	0 (0,0)	-11.6 (-62.4,128.6)
Bosnia and Herzegovina	0.5 (0.2,0.9)	1.1 (0.4,2.1)	127.5 (-0.7,427.8)	0.3 (0.2,0.6)	0.5 (0.1,0.8)	39.4 (-34.7,201.6)	7.2 (3.2,13.3)	9.6 (2.9,17.1)	34.1 (-42.4,205.8)
Bulgaria	0.5 (0.4,0.6)	0.9 (0.6,1.3)	78.5 (4.3,197.2)	0.3 (0.2,0.4)	0.4 (0.3,0.6)	39.6 (-15.9,128)	6.7 (5,8.7)	8.6 (5.6,12.6)	29.3 (-24.6,116.6)
Croatia	2 (1.6,2.5)	3.8 (3,4.7)	88.9 (40.5,156.2)	0.9 (0.8,1.1)	1.1 (0.9,1.3)	18.7 (-7.4,58.3)	19.9 (16.4,24.2)	21.9 (17.8,26.5)	10.3 (-15.6,48.1)
Czechia	1.2 (1,1.5)	2.9 (2.2,3.8)	134.9 (66.6,231.6)	0.7 (0.6,0.8)	0.9 (0.7,1.1)	27.6 (-4.3,74.7)	13.9 (11.6,16.6)	17.3 (13.4,22.2)	24.7 (-9.6,77.1)
Hungary	1.9 (1.7,2.1)	2.7 (2.2,3.1)	39.1 (16.1,65)	1.2 (1.1,1.3)	0.9 (0.7,1)	-26.2 (-37.6,-11.4)	24.1 (21.8,26.5)	18.7 (16,21.8)	-22.2 (-33.9,-8.4)
Montenegro	0.9 (0.4,1.7)	1.8 (0.6,3.4)	93.5 (-16.3,337.6)	0.4 (0.2,0.7)	0.6 (0.2,1.2)	57.2 (-31.8,250.3)	8.7 (3.3,16)	12.6 (4.1,22.8)	44.7 (-35.7,221.8)
North Macedonia	0.9 (0.4,1.5)	1.7 (0.5,3.2)	99.1 (7.5,269)	0.6 (0.3,1.1)	0.8 (0.3,1.5)	35.3 (-22.3,141.6)	12.6 (5.1,21.1)	15.5 (4.7,27.4)	23 (-31.7,122.6)
Poland	1.5 (1.3,1.8)	2.8 (2.4,3.2)	80.6 (46.9,121.7)	1 (0.8,1.2)	1 (0.9,1.2)	4 (-14.4,25.8)	21.3 (17.9,25)	20.5 (18,23)	-3.8 (-21.3,16.6)
Romania	0.5 (0.4,0.6)	1.4 (1.1,1.7)	174.9 (107.5,263.7)	0.3 (0.3,0.4)	0.5 (0.4,0.6)	58.1 (21.6,110.8)	8 (6.5,9.4)	11.7 (9.5,14)	45.4 (13.2,89.6)
Serbia	1 (0.6,1.6)	1.9 (0.9,3)	92.7 (22.1,206.5)	0.7 (0.4,1)	0.7 (0.3,1.1)	2.8 (-33.3,54.2)	13.5 (7.7,21.3)	14.4 (6.7,22.6)	6.9 (-29.5,66.7)
Slovakia	1.4 (0.6,2.4)	2.3 (1.1,3.9)	64.4 (-10.1,253)	0.8 (0.4,1.3)	0.8 (0.4,1.3)	3.2 (-43.7,104.5)	17.7 (7.6,29.8)	17.4 (8.1,29.1)	-1.6 (-46.9,110.9)
Slovenia	2.5 (2,3)	5.3 (4,7)	117 (55.2,201.1)	1.1 (0.9,1.4)	1.2 (0.9,1.5)	5.3 (-20.1,38.2)	23.3 (19.4,28.1)	24.9 (18.8,31.9)	6.8 (-22.1,44.5)
Central Asia	0.5 (0.4,0.6)	0.4 (0.3,0.5)	-21.5 (-40.9,9.7)	0.3 (0.3,0.4)	0.2 (0.2,0.3)	-34.7 (-50.8,-9.3)	9.7 (7.9,11.5)	5.6 (4.6,6.8)	-42.1 (-56,-19.6)
Armenia	1.2 (1,1.5)	0.9 (0.8,1.1)	-23.8 (-41.3,0.7)	0.7 (0.6,0.8)	0.4 (0.3,0.5)	-45.2 (-56.6,-28.9)	21.4 (18.1,25)	10 (8.7,11.7)	-53 (-62.8,-40.6)
Azerbaijan	0.3 (0.1,0.5)	0.3 (0.1,0.6)	-3.4 (-65.2,168.3)	0.2 (0.1,0.4)	0.1 (0.1,0.3)	-27.7 (-72.9,95.3)	5.7 (1.9,11.8)	3.8 (1.4,7.9)	-34.5 (-76.4,79.4)
Georgia	1.3 (0.9,1.6)	0.9 (0.7,1.2)	-28.5 (-50.4,9.8)	0.7 (0.5,0.8)	0.4 (0.3,0.6)	-33.9 (-52.8,-2.3)	21.4 (15.7,27)	11.8 (9.1,15)	-45 (-61,-16.3)

Kazakhstan	0.4 (0.2,0.6)	0.5 (0.3,0.8)	29.4 (-34.9,162.3)	0.3 (0.1,0.4)	0.2 (0.1,0.4)	-8.6 (-55.3,84.1)	7.3 (4.3,11.1)	6.1 (3.8,9.1)	-16.9 (-55.9,63.5)
Kyrgyzstan	0.6 (0.5,0.8)	0.3 (0.3,0.4)	-48.3 (-62.1,-30.1)	0.4 (0.4,0.5)	0.2 (0.1,0.2)	-61.6 (-72.1,-48)	14.2 (12,16.8)	4.9 (3.9,6.2)	-65.5 (-74.4,-52.7)
Mongolia	0.1 (0,0.1)	0.1 (0,0.1)	14.8 (-46.1,165.1)	0.1 (0,0.1)	0 (0,0.1)	-14.9 (-58.9,97.6)	1.6 (0.6,3.1)	1.2 (0.4,2.2)	-20.5 (-62,84.3)
Tajikistan	0.2 (0.1,0.4)	0.1 (0,0.3)	-31.9 (-76.6,86.7)	0.2 (0.1,0.3)	0.1 (0,0.2)	-39.1 (-79.3,67.1)	4.8 (1.6,9.9)	2.8 (0.9,6.4)	-41.7 (-81.8,66)
Turkmenistan	0.3 (0.2,0.4)	0.3 (0.2,0.4)	12.5 (-40.6,108.4)	0.2 (0.1,0.2)	0.2 (0.1,0.2)	-13.8 (-54.7,52.1)	5.6 (3.9,7.9)	4.9 (3.1,7.8)	-13.2 (-54.2,61.3)
Uzbekistan	0.3 (0.2,0.5)	0.3 (0.2,0.4)	-15.4 (-51.9,50.1)	0.2 (0.2,0.3)	0.2 (0.1,0.2)	-28.4 (-58.5,26.5)	7.3 (5,10.2)	4.9 (3.2,6.9)	-32.6 (-61.1,17.6)
Central Latin America	0.4 (0.4,0.4)	0.4 (0.3,0.5)	3.9 (-10.3,19.6)	0.3 (0.3,0.4)	0.3 (0.2,0.3)	-25.2 (-35.5,-14.3)	7.1 (6.6,7.6)	5.2 (4.5,6)	-26.8 (-37,-15.1)
Colombia	0.6 (0.5,0.6)	0.5 (0.4,0.6)	-18.6 (-37.3,4.8)	0.5 (0.4,0.5)	0.2 (0.2,0.3)	-47.8 (-59.4,-32.1)	10.1 (8.6,11.7)	5.1 (4,6.3)	-49.8 (-61.1,-35.3)
Costa Rica	0.8 (0.6,0.9)	0.9 (0.8,1.2)	25.7 (-5,71.8)	0.5 (0.4,0.6)	0.5 (0.4,0.6)	-10.3 (-33,19.6)	10.4 (8.2,12.6)	9.5 (7.6,11.6)	-8.9 (-29.3,20.8)
El Salvador	0.3 (0.1,0.4)	0.5 (0.2,0.7)	71.5 (13.7,163.4)	0.2 (0.1,0.4)	0.3 (0.1,0.4)	12.8 (-24.5,72.1)	5.5 (2.4,8.2)	6.1 (2.8,9.3)	11.2 (-25.4,65.2)
Guatemala	0.2 (0.1,0.2)	0.2 (0.2,0.2)	12.2 (-16.2,50.7)	0.2 (0.1,0.2)	0.1 (0.1,0.2)	-14.4 (-35.7,13)	3.5 (2.9,4.3)	3.2 (2.7,3.7)	-10 (-32.5,20.3)
Honduras	0.3 (0.1,0.5)	0.5 (0.2,0.9)	60.7 (-24.7,236.8)	0.3 (0.1,0.4)	0.4 (0.2,0.7)	41.5 (-33.6,192)	6.3 (2.7,10.6)	8.2 (3.5,16.1)	30 (-39.3,160.8)
Mexico	0.4 (0.3,0.4)	0.4 (0.3,0.4)	3.8 (-12.5,20.2)	0.3 (0.3,0.3)	0.2 (0.2,0.3)	-24.2 (-35.9,-13)	6.4 (6.1,6.7)	4.9 (4.2,5.7)	-23.7 (-35.6,-11.4)
Nicaragua	0.3 (0.2,0.5)	0.5 (0.2,0.7)	47.2 (-2.1,110.9)	0.3 (0.1,0.4)	0.3 (0.2,0.4)	6.7 (-27.6,57.9)	6.3 (3.4,10.1)	6.6 (3.2,9.6)	4.1 (-28.9,48.8)
Panama	0.3 (0.3,0.3)	0.5 (0.4,0.6)	63.8 (26.7,111)	0.2 (0.2,0.3)	0.3 (0.2,0.3)	12.8 (-11.6,44.6)	5 (4.4,5.6)	5.6 (4.4,6.9)	12 (-12.6,44.2)
Venezuela (Bolivarian Republic of)	0.4 (0.3,0.4)	0.4 (0.3,0.6)	9.4 (-26.1,64.1)	0.3 (0.3,0.4)	0.3 (0.2,0.4)	-13.9 (-40.5,26.5)	7 (5.8,8.1)	5.6 (4,7.7)	-19.4 (-46.1,19.8)
Andean Latin America	0.2 (0.1,0.3)	0.4 (0.2,0.6)	67.4 (16.2,134.9)	0.2 (0.1,0.3)	0.2 (0.1,0.3)	6.3 (-24.4,48.7)	4.9 (3,6.8)	5.2 (2.8,7.3)	5.5 (-26.3,48.8)

Bolivia (Plurinational State of)	0.3 (0.1,0.5)	0.4 (0.2,0.7)	30.1 (-24.7,148.3)	0.3 (0.1,0.5)	0.3 (0.1,0.6)	6.7 (-37.8,105.2)	7 (2.9,12.9)	7.1 (2.8,12.7)	1.6 (-43,102.4)
Ecuador	0.3 (0.3,0.4)	0.3 (0.3,0.5)	12.5 (-18.2,53.4)	0.3 (0.2,0.3)	0.2 (0.2,0.3)	-22.6 (-42.9,3.6)	6.4 (5.3,7.7)	5 (3.8,6.5)	-22.2 (-43.6,5.1)
Peru	0.2 (0.1,0.3)	0.4 (0.1,0.6)	130.4 (30.3,296)	0.2 (0.1,0.3)	0.2 (0.1,0.3)	30.6 (-24.6,130)	3.7 (1.7,6.2)	4.8 (1.6,7.8)	29.6 (-26.6,128.1)
Caribbean	0.8 (0.7,0.9)	0.7 (0.6,0.9)	-9.9 (-25.5,7.3)	0.6 (0.5,0.6)	0.4 (0.3,0.5)	-31.7 (-43.1,-18.9)	11.9 (10.5,13.5)	8.1 (6.7,9.7)	-31.8 (-43.7,-18.5)
Antigua and Barbuda	0.6 (0.5,0.7)	0.8 (0.7,0.9)	25.6 (2.4,55.6)	0.5 (0.4,0.6)	0.5 (0.4,0.6)	3 (-16.3,26.6)	10 (8.5,11.7)	9.4 (8.4,10.4)	-6.1 (-23.5,13.8)
Barbados	0.4 (0.3,0.4)	0.7 (0.5,0.9)	89.5 (42.1,144.1)	0.3 (0.2,0.3)	0.4 (0.3,0.5)	49.4 (15.1,90.4)	5.6 (4.9,6.4)	8.1 (6.3,10.1)	44.8 (10.8,86.3)
Belize	0.4 (0.3,0.4)	0.5 (0.4,0.6)	33.3 (5.5,68.8)	0.3 (0.3,0.4)	0.3 (0.3,0.4)	10.9 (-12,38.6)	7 (6,8.1)	7.7 (6.5,9.2)	9.8 (-13.4,41.8)
Bermuda	0 (0,0)	0 (0,0)	-28 (-47.6,1.9)	0 (0,0)	0 (0,0)	-60.4 (-71.1,-46.1)	0 (0,0)	0 (0,0)	-60.7 (-71,-45.9)
Bahamas	1.3 (1,1.5)	1.5 (1.2,1.8)	15.9 (-14.2,62.8)	0.9 (0.8,1.1)	0.9 (0.7,1.1)	-1.2 (-25,37.6)	23 (18.5,27.2)	21 (16.8,26.1)	-8.9 (-32.7,30.3)
Cuba	1.1 (1,1.3)	1 (0.8,1.2)	-11.7 (-32.2,13.6)	0.7 (0.6,0.8)	0.4 (0.4,0.5)	-37.3 (-51,-21.1)	15.1 (13.2,16.9)	9.2 (7.5,11.5)	-38.6 (-52.3,-22.1)
Dominica	0.6 (0.2,0.9)	0.6 (0.3,1)	14.3 (-34.2,113.6)	0.5 (0.2,0.8)	0.5 (0.2,0.8)	2.7 (-40.9,92.6)	9.5 (3.5,14.8)	9.8 (4.2,15.5)	2.2 (-39.6,90.5)
Dominican Republic	0.4 (0.1,0.5)	0.4 (0.2,0.6)	1.9 (-41.7,81.9)	0.3 (0.1,0.5)	0.3 (0.1,0.4)	-18.1 (-53.3,40.4)	6.8 (2.5,10.1)	5.7 (2.8,9.3)	-16.2 (-53.4,53.3)
Grenada	1.6 (1.3,2.1)	2.4 (2,2.8)	45.6 (8.7,86.4)	1.4 (1.1,1.7)	1.6 (1.4,1.8)	17 (-8.8,48.4)	32.7 (26.6,41.6)	35.3 (30,41.2)	8 (-18.4,39.8)
Guyana	0.6 (0.5,0.7)	0.6 (0.4,0.8)	-1.1 (-32.3,46.2)	0.6 (0.5,0.7)	0.5 (0.4,0.6)	-12.9 (-40.1,27.7)	13.2 (10.8,16)	11.3 (8.1,15.2)	-14.2 (-41.5,27)
Haiti	0.4 (0.1,0.7)	0.3 (0.2,0.6)	-3 (-44.6,86.2)	0.4 (0.2,0.7)	0.3 (0.2,0.6)	-8 (-47.4,73)	8.2 (3.4,16.5)	7.3 (3.2,14)	-10.7 (-48.6,70)
Jamaica	0.2 (0.2,0.3)	0.4 (0.3,0.5)	83.4 (15.9,180.1)	0.2 (0.1,0.2)	0.2 (0.2,0.3)	48.6 (-3.9,123.5)	3.3 (2.6,4.2)	5 (3.5,6.7)	51.8 (-1.4,132.4)
Puerto Rico	1.3 (1.1,1.5)	1.1 (0.8,1.4)	-12.7 (-38.2,16.6)	0.9 (0.7,1)	0.4 (0.3,0.6)	-48.1 (-62.1,-31.7)	16.5 (14,19.3)	8.8 (6.6,11.1)	-46.7 (-61.7,-29.4)
Saint Kitts and Nevis	0 (0,0)	0 (0,0)	-7.3 (-37.3,29.9)	0 (0,0)	0 (0,0)	-26.9 (-50.1,1.6)	0 (0,0)	0 (0,0)	-37.1 (-58.1,-11.3)

Saint Lucia	0.6 (0.5,0.7)	0.7 (0.6,0.9)	23.4 (-8.8,66.3)	0.5 (0.4,0.6)	0.5 (0.4,0.6)	-6.3 (-30.3,24.9)	10.7 (9,12.7)	10.1 (8,12.4)	-5.9 (-30.2,28.2)
Saint Vincent and the Grenadines	1.3 (1.1,1.5)	0.7 (0.6,0.8)	-47 (-58.3,-32.1)	1.1 (0.9,1.3)	0.5 (0.4,0.6)	-55 (-65.1,-43.8)	23.9 (20.4,27.8)	10.5 (8.7,12.5)	-56 (-65.9,-43.4)
Suriname	0.3 (0.2,0.5)	0.4 (0.2,0.6)	11.5 (-34.4,98.6)	0.3 (0.1,0.5)	0.3 (0.1,0.5)	-3.7 (-42.5,70.2)	6.7 (3.1,10.2)	6.5 (2.8,10.6)	-3 (-41.9,67.6)
Trinidad and Tobago	0.4 (0.3,0.5)	0.4 (0.3,0.5)	3.8 (-25.7,47.1)	0.3 (0.3,0.4)	0.3 (0.2,0.3)	-21.4 (-44,9.6)	7 (5.8,8.2)	5.6 (4.2,7.3)	-19.9 (-42.7,13.4)
United States Virgin Islands	1.1 (0.5,1.5)	0.6 (0.3,0.9)	-49.6 (-71.2,-11.9)	0.8 (0.4,1.1)	0.3 (0.2,0.5)	-60.3 (-75.8,-36.9)	17.4 (8.3,23.9)	6.8 (3.3,11.2)	-60.7 (-77.4,-32.2)
Tropical Latin America	0.4 (0.4,0.5)	0.6 (0.5,0.6)	27.1 (14,40.7)	0.4 (0.3,0.4)	0.4 (0.3,0.4)	-2.1 (-12.7,8.3)	7.9 (7.2,8.5)	7.3 (6.5,8)	-6.9 (-16.2,2.8)
Brazil	0.4 (0.4,0.5)	0.6 (0.5,0.6)	26.4 (13.5,40.4)	0.4 (0.3,0.4)	0.4 (0.3,0.4)	-2.7 (-12.9,8)	7.9 (7.2,8.5)	7.3 (6.4,7.9)	-7.6 (-17.1,2.8)
Paraguay	0.5 (0.2,0.7)	0.7 (0.4,1.1)	61.5 (2.1,157.1)	0.4 (0.2,0.6)	0.5 (0.2,0.8)	27.3 (-21.1,97.5)	7.8 (4.1,12.2)	9.9 (4.6,14.7)	26.8 (-21.6,101.8)
East Asia	0.6 (0.3,0.9)	1.1 (0.5,1.6)	82.2 (7.7,241.2)	0.4 (0.2,0.6)	0.3 (0.1,0.4)	-31.7 (-58.4,24.2)	14.1 (6.7,21.7)	10 (4.1,14.8)	-29.5 (-58.9,36.3)
China	0.6 (0.3,0.9)	1.2 (0.5,1.7)	82 (7.2,243)	0.5 (0.2,0.7)	0.3 (0.1,0.4)	-32.2 (-59.1,24.3)	14.5 (6.8,22.4)	10.2 (4.2,15.2)	-30 (-59.5,36.5)
Democratic People's Republic of Korea	0.3 (0.1,0.5)	0.4 (0.2,0.8)	42 (-20.5,165.5)	0.2 (0.1,0.4)	0.2 (0.1,0.4)	0.6 (-40.5,77.7)	6.9 (2.9,12.6)	7.3 (2.8,12.4)	5.8 (-40.5,107.3)
Taiwan (Province of China)	0.2 (0.1,0.3)	0.4 (0.3,0.5)	150.4 (45.6,327.9)	0.1 (0.1,0.1)	0.1 (0.1,0.1)	26 (-24.6,108.2)	2 (1.3,3.2)	2.9 (2.3,3.5)	41.8 (-15.8,135.4)
Southeast Asia	0.1 (0.1,0.2)	0.1 (0.1,0.2)	4.4 (-27.5,46)	0.1 (0.1,0.2)	0.1 (0,0.1)	-20.4 (-44.5,10)	2.7 (1.2,3.8)	2.1 (1,3.2)	-21.8 (-45.1,6)
Cambodia	0.1 (0.1,0.3)	0.1 (0.1,0.3)	6.6 (-48.6,110.1)	0.1 (0.1,0.3)	0.1 (0,0.2)	-11.8 (-56.9,72.2)	3 (1.3,6.3)	2.5 (1,4.7)	-15.4 (-61.6,68.7)
Indonesia	0.1 (0.1,0.2)	0.1 (0.1,0.3)	25.6 (-17.1,95.2)	0.1 (0.1,0.2)	0.1 (0.1,0.2)	6.3 (-28.6,61.4)	2.3 (1.2,4.1)	2.3 (1.2,4.5)	0.1 (-35.2,58)
Lao People's Democratic Republic	0.1 (0.1,0.2)	0.1 (0.1,0.2)	-8.1 (-51.7,87.6)	0.1 (0.1,0.2)	0.1 (0,0.2)	-20.4 (-57.5,57.6)	2.9 (1.2,5.9)	2.3 (1,4.1)	-22.6 (-61.3,57)

Malaysia	0.1 (0,0.2)	0.1 (0.1,0.2)	33.5 (-19.6,111.7)	0.1 (0,0.2)	0.1 (0,0.2)	0.8 (-39.4,64.7)	2 (0.9,3.1)	1.8 (0.8,3)	-8.3 (-43.6,47.4)
Maldives	0.1 (0,0.2)	0.1 (0,0.1)	-11.9 (-64.7,130.8)	0.1 (0,0.2)	0 (0,0.1)	-48.2 (-76.1,28.5)	1.5 (0.6,3.7)	0.7 (0.3,1.6)	-54.1 (-82.4,21.2)
Mauritius	1.2 (1,1.3)	0.1 (0.1,0.1)	-89.9 (-92.1,-86.7)	0.8 (0.7,0.9)	0.1 (0.1,0.1)	-91.7 (-93.5,-89.4)	20.2 (17.2,23.7)	1.5 (1.3,1.8)	-92.6 (-94.2,-90.5)
Myanmar	0.2 (0.1,0.3)	0.1 (0.1,0.2)	-21.3 (-64.3,54.9)	0.2 (0.1,0.3)	0.1 (0,0.2)	-34.2 (-69.5,31.4)	3.5 (1.8,6.9)	2.2 (1,4.1)	-37.1 (-72.3,28.1)
Philippines	0.2 (0,0.2)	0.2 (0,0.3)	-5.2 (-29.3,37.1)	0.2 (0,0.2)	0.1 (0,0.2)	-19.9 (-39.9,18.5)	3.4 (0.8,4.5)	3 (0.7,4.5)	-12.5 (-34.8,24.5)
Sri Lanka	0 (0,0.1)	0.1 (0,0.2)	119.4 (-11.8,895.1)	0 (0,0.1)	0 (0,0.1)	30.8 (-43.9,479.8)	0.8 (0.2,1.7)	1.1 (0.4,2)	32.9 (-45,491.7)
Seychelles	0.6 (0.2,0.9)	0.6 (0.2,0.9)	9 (-29,68.6)	0.5 (0.2,0.7)	0.4 (0.1,0.6)	-13.7 (-45.1,29.7)	10.5 (3.7,15.9)	8.6 (3.3,12.7)	-18.5 (-47.3,25.6)
Thailand	0.3 (0.1,0.5)	0.3 (0.1,0.5)	-15.3 (-53.6,74)	0.3 (0.1,0.4)	0.1 (0,0.2)	-49.1 (-71.5,1.1)	5.8 (1.6,9)	3.1 (1.1,4.8)	-47.4 (-70.8,8.2)
Timor-Leste	0.1 (0,0.2)	0.1 (0,0.2)	4.4 (-39.6,96.7)	0.1 (0,0.2)	0.1 (0,0.2)	-9.1 (-47.4,70.6)	2.2 (1.1,4.2)	2 (0.9,3.8)	-8.6 (-49.3,70.7)
Viet Nam	0 (0,0)	0 (0,0)	-5.9 (-51.1,75.6)	0 (0,0)	0 (0,0)	-39.3 (-67,14.7)	0.5 (0.1,0.7)	0.3 (0.1,0.5)	-35.4 (-67,20.7)
Oceania	0 (0,0)	0 (0,0)	13.5 (-42.2,105)	0 (0,0)	0 (0,0)	11.8 (-44.6,114.1)	0.1 (0,0.1)	0.1 (0,0.1)	-1.1 (-43.4,68)
American Samoa	0 (0,0)	0 (0,0)	344 (-30.2,1969.2)	0 (0,0)	0 (0,0)	315.1 (-37.6,2051.5)	0.1 (0,0.2)	0.2 (0,0.4)	339.8 (-33.8,1793.4)
Cook Islands	0 (0,0)	0 (0,0)	14.5 (-64,331.5)	0 (0,0)	0 (0,0)	-4.5 (-68.7,266.9)	0 (0,0.1)	0 (0,0.1)	-4.6 (-68.8,243.5)
Micronesia (Federated States of)	0 (0,0)	0 (0,0)	43.6 (-44.2,369.6)	0 (0,0)	0 (0,0)	39.2 (-48.9,410.3)	0.1 (0,0.2)	0.1 (0,0.2)	38.2 (-47.5,339.9)
Fiji	0 (0,0)	0 (0,0)	8.4 (-66.5,218.1)	0 (0,0)	0 (0,0)	6.3 (-68.7,233.7)	0 (0,0)	0 (0,0)	3.7 (-65.5,180.4)
Guam	0 (0,0)	0 (0,0)	17.5 (-94.1,1083.7)	0 (0,0)	0 (0,0)	-7.1 (-95.3,896.1)	0 (0,0.1)	0 (0,0)	22.7 (-94.1,1143.7)
Kiribati	0 (0,0)	0 (0,0)	84.6 (-29.7,616.3)	0 (0,0)	0 (0,0)	79.7 (-29.7,625.9)	0 (0,0.1)	0.1 (0,0.1)	75.6 (-27.7,535.1)
Marshall Islands	0 (0,0)	0 (0,0)	46.7 (-41.1,409.1)	0 (0,0)	0 (0,0)	44.3 (-42.8,431.2)	0 (0,0.1)	0.1 (0,0.1)	44.5 (-39,386.7)

Nauru	0 (0,0)	0 (0,0)	23.5 (-60.9,388.6)	0 (0,0)	0 (0,0)	19.6 (-63,374.4)	0.1 (0,0.2)	0.1 (0,0.2)	19.5 (-59.2,321.5)
Niue	0 (0,0)	0 (0,0)	47.5 (-50.1,460.9)	0 (0,0)	0 (0,0)	37 (-53.8,459.8)	0 (0,0.1)	0.1 (0,0.1)	37.9 (-51.2,378.3)
Northern Mariana Islands	0 (0,0)	0 (0,0)	-16.1 (-68.7,230.8)	0 (0,0)	0 (0,0)	-21.4 (-68.5,222.7)	0 (0,0.1)	0 (0,0)	-15.7 (-65.9,234)
Palau	0.3 (0.1,0.6)	0.4 (0.1,0.7)	23.3 (-27.3,101.9)	0.2 (0.1,0.4)	0.3 (0.1,0.4)	8.2 (-34.4,83.7)	7.2 (1.2,13)	7.3 (1.5,13)	1.5 (-42.1,70.5)
Papua New Guinea	0 (0,0)	0 (0,0)	26.8 (-58.8,337.4)	0 (0,0)	0 (0,0)	25.5 (-58.8,360.3)	0 (0,0.1)	0 (0,0.1)	25.8 (-55.2,299)
Samoa	0 (0,0)	0 (0,0)	65.5 (-37.7,586.5)	0 (0,0)	0 (0,0)	56.3 (-44.5,594.6)	0.1 (0,0.2)	0.1 (0,0.2)	56 (-39.6,529.7)
Solomon Islands	0 (0,0)	0 (0,0)	43 (-44.8,385.2)	0 (0,0)	0 (0,0)	39.1 (-46.8,401.5)	0 (0,0.1)	0.1 (0,0.1)	40.3 (-45.2,356.4)
Tokelau	0 (0,0)	0 (0,0)	37.5 (-54.3,338.4)	0 (0,0)	0 (0,0)	27.1 (-56.7,328.5)	0.1 (0,0.1)	0.1 (0,0.1)	27.9 (-54.1,296.1)
Tonga	0 (0,0)	0 (0,0)	65.7 (-35.5,525.4)	0 (0,0)	0 (0,0)	57.7 (-41.2,518.6)	0 (0,0.1)	0 (0,0.1)	55.3 (-38.4,464.1)
Tuvalu	0 (0,0)	0 (0,0)	49.4 (-47.2,436.8)	0 (0,0)	0 (0,0)	42.6 (-49.5,431.8)	0.1 (0,0.2)	0.1 (0,0.2)	40.9 (-48.4,392.3)
Vanuatu	0 (0,0)	0 (0,0)	37 (-48.8,397.2)	0 (0,0)	0 (0,0)	36.2 (-49.9,427.2)	0 (0,0.1)	0.1 (0,0.1)	35.8 (-48.7,370.1)
North Africa and Middle East	0.6 (0.2,0.9)	0.9 (0.3,1.2)	44.9 (4.6,100.7)	0.6 (0.2,0.8)	0.5 (0.2,0.7)	-8.4 (-33.6,26)	11.8 (3.8,16.9)	10.5 (3.2,13.9)	-10.5 (-36.9,24.5)
Afghanistan	1.1 (0.2,2.4)	1.3 (0.2,2.6)	17.8 (-35.5,120.3)	1.1 (0.2,2.4)	1.2 (0.2,2.4)	7.5 (-40.8,96.1)	23 (3.8,54.6)	25.1 (3.9,56.1)	9.5 (-42.9,110.5)
Algeria	0.1 (0,0.2)	0.2 (0,0.3)	34 (-24.1,144.9)	0.1 (0,0.2)	0.1 (0,0.2)	-6.4 (-49,70.8)	1.9 (0.4,3.1)	1.8 (0.3,3.1)	-6.1 (-46.5,70)
Bahrain	0.2 (0,0.3)	0.2 (0,0.3)	3.3 (-43,91.6)	0.2 (0,0.3)	0.1 (0,0.2)	-37.9 (-66.9,12)	3.1 (0.5,5)	1.8 (0.4,3.1)	-41 (-67.9,7.7)
Egypt	0.4 (0.1,0.7)	1.2 (0.2,2.1)	182 (8.7,386.7)	0.4 (0.1,0.7)	0.9 (0.2,1.5)	105.7 (-19.6,243.1)	8.1 (2.6,12.7)	16.7 (2.9,28.1)	105.8 (-19.7,243)
Iran (Islamic Republic of)	0.9 (0.3,1.3)	1.2 (0.4,1.7)	30.7 (-4.1,80.5)	0.7 (0.3,1)	0.6 (0.2,0.8)	-22.6 (-40.2,2.2)	16.3 (5.4,22)	12.1 (4,16.6)	-26 (-44.5,-0.6)
Iraq	0.3 (0.1,0.5)	0.5 (0.1,0.8)	58.3 (-23.6,251.8)	0.2 (0.1,0.4)	0.3 (0.1,0.4)	4.9 (-46.9,130.9)	5.6 (1.8,9.8)	5.7 (1.6,9.5)	1.9 (-50,119.5)

Jordan	0.3 (0.1,0.9)	1.3 (0.4,2.2)	338.1 (-8.9,925)	0.2 (0.1,0.7)	0.6 (0.2,1.1)	175.2 (-38.5,521.7)	4.9 (2.2,14.4)	12.6 (3.6,21.8)	156.8 (-46.1,498.4)
Kuwait	0 (0,0)	0.2 (0.1,0.3)	244493.3 (173442.9,347037.8)	0 (0,0)	0.1 (0,0.1)	140811.7 (99587.5,200703.4)	0 (0,0)	1.3 (1,1.7)	149977.2 (107066.8,212061.1)
Lebanon	0.7 (0.4,1.2)	1 (0.4,1.8)	45.2 (-35.2,215.8)	0.6 (0.3,0.9)	0.5 (0.2,0.8)	-17.4 (-59.7,69.3)	11 (5.3,18.9)	8.8 (3.2,14.8)	-20.3 (-65,69.6)
Libya	0.9 (0.1,1.6)	1.3 (0.2,2.2)	33.1 (-25.7,149)	0.8 (0.1,1.3)	0.8 (0.1,1.3)	0.3 (-43.1,82.7)	15.2 (2.4,25.8)	15.3 (2.8,26.3)	0.8 (-44.4,87.4)
Morocco	0.1 (0,0.2)	0.1 (0,0.2)	33.2 (-25.6,159.9)	0.1 (0,0.2)	0.1 (0,0.2)	0.5 (-42.5,87.8)	1.9 (0.2,3.4)	1.9 (0.3,3.4)	2.1 (-42.5,96.5)
Palestine	1.3 (0.7,2.4)	1.6 (0.8,2.5)	20.6 (-32.3,125.8)	1 (0.5,1.8)	0.8 (0.5,1.4)	-18.5 (-54.6,51)	22.9 (11.8,42.3)	18.3 (10,29.2)	-19.9 (-55.5,51.6)
Oman	0 (0,0)	0 (0,0)	36.8 (-36.3,205.1)	0 (0,0)	0 (0,0)	-13.5 (-58.6,99.7)	0 (0,0)	0 (0,0)	-21 (-61.8,79)
Qatar	0.4 (0.1,0.7)	0.4 (0.1,0.7)	-1.2 (-52,101.8)	0.3 (0.1,0.5)	0.1 (0,0.2)	-50.7 (-76.7,3.4)	5.4 (1.1,10.4)	2.7 (0.7,4.7)	-51.2 (-75.7,4.7)
Saudi Arabia	0.3 (0.1,0.6)	0.7 (0.2,1.6)	193.7 (45,480.3)	0.2 (0.1,0.6)	0.3 (0.1,0.7)	52.4 (-20.2,185.3)	4.6 (1.3,12.1)	7.4 (1.4,15.4)	61.6 (-21.6,229.6)
Sudan	0.6 (0.1,1.1)	0.7 (0.1,1.3)	21.2 (-35,173.4)	0.6 (0.1,1.1)	0.6 (0.1,1)	-5 (-49.6,96.2)	12 (1.5,24)	11.7 (1.7,21.4)	-3.3 (-51.7,113.2)
Syrian Arab Republic	1 (0.2,1.9)	1.4 (0.3,2.3)	33.5 (-25.4,136.5)	0.9 (0.1,1.7)	0.8 (0.2,1.4)	-10.9 (-52.3,55.3)	17.9 (2.8,32.4)	15 (2.8,25.3)	-16.2 (-53,52)
Tunisia	0.5 (0.1,0.8)	0.6 (0.1,1)	22.3 (-33.4,109.6)	0.4 (0,0.7)	0.3 (0,0.5)	-24 (-60.1,34.6)	6.9 (0.9,12.1)	5.5 (0.8,10.3)	-19.9 (-55.2,40.7)
Turkey	1 (0.4,1.6)	1.2 (0.5,1.8)	19.9 (-25.4,99.1)	0.9 (0.4,1.4)	0.6 (0.3,0.9)	-30.5 (-56.2,14.3)	18.9 (7.2,29.1)	11.8 (5.1,17.1)	-37.4 (-61.8,2.7)
United Arab Emirates	1.9 (0.7,3.5)	4.1 (1.9,8)	114.8 (-5.6,482.1)	1.5 (0.6,2.7)	2.6 (1.2,5.1)	72.1 (-26.8,357.1)	33.9 (12.3,60.8)	46.7 (22.3,90.1)	37.8 (-39.8,266.5)
Yemen	0.6 (0.1,1.1)	0.7 (0.1,1.4)	22.7 (-41.2,144.5)	0.6 (0.1,1.2)	0.6 (0.1,1.2)	6.3 (-47.8,111.2)	12.1 (1.5,23.2)	13.1 (1.4,24.6)	8 (-50.3,127.3)
South Asia	0 (0,0)	0 (0,0)	34.5 (-12.2,147.7)	0 (0,0)	0 (0,0)	2.5 (-32.7,93.3)	0.4 (0,0.6)	0.4 (0,0.5)	-1.3 (-34.6,81)
Bangladesh	0 (0,0)	0 (0,0)	-12.9 (-59.2,101.1)	0 (0,0)	0 (0,0)	-32.3 (-67.4,48.6)	0.1 (0,0.2)	0.1 (0,0.2)	-35.6 (-71.4,50.7)
Bhutan	0 (0,0)	0 (0,0)	3.7 (-52.2,134.7)	0 (0,0)	0 (0,0)	-15.8 (-62,98.4)	0.1 (0,0.3)	0.1 (0.1,0.2)	-22.7 (-65.4,76.6)

India	0 (0,0)	0 (0,0)	35.6 (-12.7,153.2)	0 (0,0)	0 (0,0)	2 (-34.4,94.7)	0.4 (0,0.7)	0.4 (0,0.6)	-0.5 (-35.4,84.1)
Nepal	0 (0,0)	0 (0,0)	-5.9 (-51.8,105.4)	0 (0,0)	0 (0,0)	-20.7 (-60,70.9)	0.2 (0,0.3)	0.1 (0,0.2)	-24.5 (-61.8,64.3)
Pakistan	0 (0,0)	0 (0,0)	-5 (-47.7,65.5)	0 (0,0)	0 (0,0)	-13 (-52.6,52.9)	0.2 (0.1,0.3)	0.1 (0.1,0.3)	-14 (-52.2,51.4)
Southern Sub-Saharan Africa	1 (0.4,1.4)	1.6 (0.6,2)	48.9 (10.2,106.1)	0.9 (0.4,1.3)	1.2 (0.5,1.6)	31.4 (-1.4,83.5)	20.2 (7.6,26.9)	25.8 (9.8,34.4)	27.6 (-3.8,72.5)
Botswana	0.9 (0.5,1.5)	1.1 (0.4,1.6)	12.3 (-35.1,89.7)	0.9 (0.5,1.4)	0.9 (0.4,1.4)	1.1 (-40.1,70.4)	19.2 (9.4,29.9)	18.8 (7.1,28.5)	-1.9 (-43.8,69.9)
Lesotho	0.8 (0.4,1.1)	1.5 (0.7,2.5)	100.2 (13,266)	0.8 (0.4,1.1)	1.5 (0.8,2.3)	93 (9.5,246.4)	15.6 (7.9,23.7)	31.2 (14.5,51.5)	100.7 (8.8,272.1)
Namibia	0.7 (0.4,1)	0.8 (0.4,1.3)	14.9 (-27.3,76)	0.7 (0.4,1)	0.7 (0.4,1)	-1 (-37.2,50.8)	15.2 (8.1,22)	14.6 (7.3,23.1)	-3.9 (-40.9,52.2)
South Africa	1.1 (0.4,1.5)	1.6 (0.5,2.1)	49 (7.1,106.9)	0.9 (0.3,1.4)	1.2 (0.4,1.6)	29.1 (-7.1,81.2)	20.5 (7,28.4)	25.1 (8.6,34.1)	22.3 (-10.1,66.1)
Eswatini	1.1 (0.6,1.6)	1.4 (0.6,2.3)	32.9 (-16.5,115.2)	1.1 (0.6,1.5)	1.3 (0.6,2)	23.6 (-21.1,93.8)	22 (12,32.9)	27.6 (12.1,44.8)	25.7 (-23.9,103.6)
Zimbabwe	1.1 (0.6,1.5)	1.5 (0.8,2.2)	46 (-10.4,140.4)	1 (0.6,1.5)	1.4 (0.8,2.1)	44.1 (-9.1,132.4)	20.5 (11,30.1)	31.8 (15.7,48.8)	54.5 (-6.2,161.8)
Western Sub-Saharan Africa	0.3 (0.1,0.4)	0.3 (0.1,0.4)	28.8 (-6.6,67.1)	0.3 (0.1,0.3)	0.3 (0.1,0.4)	17.6 (-14.4,50.7)	5.7 (2.8,7.7)	6.5 (2.5,8.5)	13.7 (-17.8,49.5)
Benin	0.3 (0.2,0.4)	0.4 (0.1,0.6)	43.8 (-24,113.5)	0.3 (0.2,0.4)	0.4 (0.1,0.5)	31.2 (-29.2,94.6)	6.6 (3.5,9.5)	8.5 (2.6,12)	28.6 (-31.3,94.3)
Burkina Faso	0.3 (0.2,0.5)	0.5 (0.1,0.7)	35.4 (-27.9,105.9)	0.3 (0.2,0.5)	0.4 (0.1,0.6)	27.7 (-29.4,96.4)	7.2 (3.4,11)	9.1 (2.5,13.7)	25.8 (-31.3,90.8)
Cameroon	0.4 (0.2,0.6)	0.5 (0.2,0.9)	35.6 (-30.8,127.7)	0.4 (0.2,0.6)	0.5 (0.1,0.8)	23.1 (-36.9,99.7)	8.2 (3.8,12.6)	10.1 (2.9,16.9)	22.2 (-38.6,108.7)
Cabo Verde	0.6 (0.3,1)	1.3 (0.5,2)	112.8 (5.9,248.2)	0.5 (0.3,0.8)	0.9 (0.3,1.3)	68.5 (-16.6,175.4)	11.7 (6.4,18.7)	18.1 (7.2,27.9)	55 (-24.2,153.9)
Chad	0.3 (0.1,0.4)	0.5 (0.2,0.7)	70.1 (-1.2,153.2)	0.3 (0.1,0.4)	0.4 (0.2,0.7)	62.7 (-6.7,146.2)	6 (3,8.8)	9.7 (3.3,15.2)	63.1 (-6.8,147.1)
CÃ´te d'Ivoire	0.2 (0.1,0.3)	0.3 (0.1,0.4)	18 (-26.5,77.4)	0.2 (0.1,0.3)	0.3 (0.1,0.4)	4.8 (-33.7,56.7)	5 (2.3,7.4)	5.4 (2.1,8.7)	6.5 (-35,65.1)
Gambia	0.2 (0.1,0.2)	0.2 (0.1,0.4)	34.2 (-12.1,103.1)	0.2 (0.1,0.2)	0.2 (0.1,0.3)	20.9 (-19.9,79.7)	3.5 (1.6,5.2)	4.3 (1.9,6.9)	22.6 (-20.2,88.2)

Ghana	0.6 (0.2,0.8)	0.4 (0.2,0.6)	-32.1 (-58.9,50)	0.5 (0.2,0.8)	0.3 (0.2,0.5)	-39.2 (-63.4,32.3)	12.1 (3.7,17.6)	7.1 (3.6,11.7)	-41.3 (-65.4,29.3)
Guinea	0.1 (0.1,0.2)	0.1 (0.1,0.2)	13.5 (-26,87)	0.1 (0.1,0.2)	0.1 (0.1,0.2)	6.4 (-29.1,74.1)	2.6 (1.2,3.9)	2.7 (1.1,4.2)	5.8 (-32.2,76.2)
Guinea-Bissau	0.4 (0.2,0.6)	0.6 (0.2,0.9)	56.3 (-10,147.1)	0.4 (0.2,0.6)	0.6 (0.2,0.8)	46.8 (-14,127.7)	8.6 (4,14.3)	12.3 (4.3,18.9)	43.3 (-16.6,132.4)
Liberia	0.3 (0.2,0.5)	0.5 (0.2,0.8)	63.3 (-6.5,153.4)	0.3 (0.2,0.5)	0.5 (0.2,0.7)	40.7 (-19.4,114)	7.2 (3.4,10.8)	10.2 (3.2,15.9)	41.9 (-19.9,119.9)
Mali	0.3 (0.2,0.5)	0.4 (0.2,0.6)	9.8 (-25.7,66.5)	0.3 (0.2,0.5)	0.3 (0.2,0.5)	0.6 (-31.9,50.2)	7.8 (3.5,11.7)	7.7 (3.1,11.3)	-1 (-34.3,54.1)
Mauritania	0.4 (0.2,0.6)	0.6 (0.2,0.9)	56.7 (-12.8,150.5)	0.4 (0.2,0.5)	0.5 (0.2,0.7)	25.9 (-30.7,99.4)	8.3 (3.7,12.2)	10.2 (3.2,15.5)	23.2 (-32.7,98.6)
Niger	0.3 (0.2,0.4)	0.4 (0.1,0.7)	47.6 (-15.3,136.3)	0.3 (0.2,0.4)	0.4 (0.2,0.6)	39.4 (-19.7,122.9)	6.4 (3.2,9.7)	8.7 (3,14)	35.6 (-23,116.1)
Nigeria	0.2 (0.1,0.3)	0.3 (0.1,0.4)	28.4 (-20.3,96.5)	0.2 (0.1,0.3)	0.2 (0.1,0.3)	15.2 (-27,72.4)	4.2 (2,6)	4.7 (1.8,6.8)	13.1 (-32.7,77.9)
Sao Tome and Principe	0.3 (0.2,0.4)	0.4 (0.2,0.7)	43.1 (-3.9,97.9)	0.3 (0.2,0.4)	0.3 (0.2,0.5)	16.9 (-19.4,62.6)	6.3 (3.2,9.3)	7.4 (3.4,11.6)	17.9 (-22.5,63.8)
Senegal	0.3 (0.1,0.4)	0.5 (0.2,0.7)	64.2 (-3.8,146.6)	0.3 (0.2,0.4)	0.4 (0.2,0.6)	47.9 (-13.5,123.3)	6.5 (3.1,9.4)	9.5 (3.1,13.8)	45.7 (-16.1,120.3)
Sierra Leone	0.3 (0.1,0.4)	0.5 (0.1,0.7)	70.4 (-2.6,159)	0.3 (0.1,0.4)	0.4 (0.1,0.6)	55 (-10.7,136.6)	5.8 (2.4,8.4)	9.2 (2.7,13.6)	58 (-10.1,143.5)
Togo	0.3 (0.2,0.4)	0.5 (0.2,0.7)	63.7 (-8.5,137.6)	0.3 (0.2,0.4)	0.5 (0.2,0.7)	50.1 (-16,119.7)	6.8 (3.4,9.6)	10 (3,14.9)	47.3 (-19,115)
Eastern Sub-Saharan Africa	1 (0.5,1.4)	1 (0.5,1.5)	7.1 (-28,61.6)	1 (0.5,1.4)	1 (0.5,1.4)	-2.2 (-32.2,45.7)	22.1 (11.3,34.1)	20 (9.3,29.1)	-9.5 (-43,41.4)
Burundi	0.6 (0.2,0.9)	0.7 (0.3,1)	15.7 (-31.6,118)	0.6 (0.3,0.9)	0.6 (0.3,1)	10.5 (-34.4,104.3)	13 (5.6,20.3)	13.8 (5.1,22.2)	5.8 (-40.2,105.7)
Comoros	0.6 (0.3,0.9)	0.8 (0.4,1.3)	39.8 (-6.8,115.5)	0.6 (0.3,0.9)	0.8 (0.4,1.2)	28.6 (-13.7,96)	13.5 (7,20.2)	16.7 (7.7,25.9)	24.1 (-19,100.3)
Djibouti	0.5 (0.3,0.8)	0.8 (0.3,1.4)	56.1 (-0.4,152.5)	0.5 (0.3,0.7)	0.7 (0.3,1.2)	43.4 (-8.5,128.9)	11.1 (5.2,16.7)	15.5 (6.1,26.9)	39.1 (-12.2,130.1)
Eritrea	0.6 (0.3,0.8)	0.8 (0.4,1.3)	50.7 (-0.5,141.1)	0.6 (0.3,0.8)	0.8 (0.4,1.3)	43.1 (-6,124.1)	13 (6.8,20.1)	17.7 (7.3,28)	36 (-13.2,130)
Ethiopia	2.4 (1.1,3.9)	2 (0.9,3.3)	-15.1 (-54,54)	2.5 (1.2,4)	1.8 (0.9,3.1)	-25.7 (-58.1,33.3)	54.3 (25.9,95)	38.3 (17.4,63.4)	-29.4 (-65.3,35.4)

Kenya	0.5 (0.3,0.8)	0.7 (0.4,1)	39.5 (0.8,95.9)	0.5 (0.3,0.8)	0.6 (0.3,0.9)	29.3 (-6.6,81.8)	10 (5.7,16.9)	12.8 (6.9,19.1)	27.5 (-8.4,83.2)
Madagascar	0.5 (0.3,0.7)	0.6 (0.3,0.9)	28.1 (-14.8,94.5)	0.5 (0.3,0.7)	0.6 (0.3,0.9)	20.1 (-21.3,78.9)	11.1 (5.8,15.7)	13.1 (5.8,19.3)	17.5 (-23.6,82)
Malawi	0.2 (0.1,0.3)	0.2 (0.1,0.3)	21.6 (-20,88.3)	0.2 (0.1,0.3)	0.2 (0.1,0.3)	11.5 (-27,69.8)	4.2 (1.9,6.1)	4.5 (1.9,6.9)	8.4 (-29.8,72.2)
Mozambique	0.9 (0.4,1.2)	1.1 (0.5,1.6)	23.9 (-24.8,98.3)	0.9 (0.4,1.3)	1.1 (0.5,1.6)	17.9 (-28,90.1)	18.8 (8.5,26.5)	21.8 (9.9,32.9)	15.8 (-29.9,91.5)
Rwanda	0.7 (0.3,1.1)	0.9 (0.4,1.3)	17.4 (-27.3,149.4)	0.8 (0.4,1.1)	0.8 (0.3,1.2)	4.4 (-36.9,116.4)	17.1 (7.9,24.9)	16.5 (6.8,25.7)	-3.1 (-42.1,111.2)
Somalia	0.6 (0.3,0.9)	0.7 (0.4,1.2)	32.2 (-12,117.2)	0.6 (0.3,0.9)	0.8 (0.4,1.2)	30.4 (-13,114)	13.1 (6.2,21.8)	16.7 (7.8,26.9)	27.5 (-17.3,113.7)
South Sudan	0.5 (0.3,0.8)	0.8 (0.4,1.2)	53 (2.4,144.3)	0.5 (0.3,0.8)	0.7 (0.4,1.2)	43.1 (-3.5,127.8)	11.5 (6.5,18.4)	16.4 (7.7,26)	42.1 (-5.7,134.5)
United Republic of Tanzania	0.6 (0.3,0.9)	0.8 (0.3,1.3)	34.3 (-10.7,104.4)	0.6 (0.3,0.9)	0.7 (0.3,1.2)	22.7 (-18.5,92.9)	13.3 (6.1,19.6)	16.1 (6.3,25.7)	20.5 (-20.2,87)
Uganda	0.3 (0.1,0.4)	0.4 (0.2,0.6)	26 (-21,110.6)	0.3 (0.2,0.4)	0.3 (0.2,0.5)	15.5 (-28.4,87.8)	6.2 (3,9.3)	7.1 (3.3,11.1)	14.1 (-28.8,95.7)
Zambia	0.6 (0.3,0.8)	0.8 (0.3,1.4)	37.9 (-22.2,122.8)	0.6 (0.3,0.8)	0.8 (0.3,1.2)	24.3 (-27.2,96.8)	13.9 (7.3,19.4)	16.8 (6,27.7)	21.2 (-35.4,109.9)
Central Sub-Saharan Africa	0.5 (0.2,0.7)	0.6 (0.2,1)	25.4 (-14.2,79.5)	0.5 (0.2,0.7)	0.6 (0.2,0.9)	14.2 (-22,61.8)	11.8 (4.5,17.2)	13.3 (4.4,20.1)	12.2 (-25.1,64.1)
Angola	0.5 (0.2,0.7)	0.6 (0.2,1)	27.8 (-23,103)	0.5 (0.2,0.7)	0.6 (0.2,0.9)	14.3 (-30.6,77)	11.4 (4.1,17.3)	12.6 (3.8,21.1)	10.4 (-36.5,82.2)
Central African Republic	0.6 (0.2,0.9)	0.6 (0.2,0.9)	7.2 (-29.7,61.7)	0.6 (0.2,0.9)	0.6 (0.2,1)	5.7 (-30.5,59.3)	13.7 (4.9,21.1)	14.3 (4.8,22.7)	3.9 (-33.6,60.9)
Congo	0.7 (0.3,1)	0.8 (0.3,1.2)	23.8 (-19.8,89.7)	0.7 (0.3,0.9)	0.7 (0.2,1)	7.8 (-30.5,65.6)	15.2 (5.7,23)	16.4 (5.4,25.1)	8.1 (-31.5,73)
Democratic Republic of the Congo	0.5 (0.2,0.7)	0.6 (0.2,1)	27 (-17.1,91.8)	0.5 (0.2,0.8)	0.6 (0.2,0.9)	16.8 (-24.4,79.4)	11.5 (4.4,17.4)	13.2 (4.5,20.5)	14.7 (-26,80.3)
Equatorial Guinea	0.5 (0.2,0.8)	0.7 (0.2,1.2)	40.2 (-22.8,144.5)	0.5 (0.2,0.8)	0.6 (0.2,0.9)	5.5 (-40.4,82)	12.4 (4.5,19.3)	12.6 (3.6,22.1)	1.7 (-48.1,89.4)
Gabon	0.6 (0.2,0.8)	0.8 (0.2,1.2)	34.6 (-13,107.9)	0.5 (0.2,0.8)	0.6 (0.2,0.9)	12.8 (-25.7,74.1)	12.4 (4.6,18.3)	13.7 (4.3,21.2)	10.6 (-29.2,74.1)

Table S9 Age-standardized incidence rates, death rates, and DALY rates in 1990 and 2021, and the percentage change in the age-standardized rates for CML among WCBA (15–49 years), by country.

	Incidence (95% UI)			Deaths (95% UI)			DALYs (95% UI)		
	ASRs per 100000 (95% UI), 1990	ASRs per 100000 (95% UI), 2021	Percentage changes in ASRs from 1990 to 2021	ASRs per 100000 (95% UI), 1990	ASRs per 100000 (95% UI), 2021	Percentage changes in ASRs from 1990 to 2021	ASRs per 100000 (95% UI), 1990	ASRs per 100000 (95% UI), 2021	Percentage changes in ASRs from 1990 to 2021
Global	0.8 (0.6,0.9)	0.3 (0.3,0.4)	-54.4 (-62.3,-43.3)	0.6 (0.5,0.8)	0.2 (0.2,0.3)	-61.9 (-69.3,-51.4)	20.3 (14.8,26.4)	7.3 (5.4,10)	-63.7 (-72.4,-50.9)
High-income North America	1.4 (1.4,1.5)	0.5 (0.5,0.5)	-64.9 (-67.2,-62.4)	0.9 (0.8,0.9)	0.2 (0.2,0.3)	-72.7 (-74.9,-70.8)	26.9 (25.6,28.1)	5.9 (5.4,6.3)	-78 (-79.3,-76.5)
Canada	1.5 (1.3,1.8)	0.6 (0.5,0.7)	-61.9 (-71.1,-49.9)	0.8 (0.7,0.9)	0.2 (0.2,0.2)	-77 (-81.5,-71.4)	23.9 (20.8,26.6)	4.9 (3.9,5.9)	-79.6 (-83.8,-74.4)
Greenland	0.1 (0,0.2)	0 (0,0.1)	-86.9 (-95.7,-45.7)	0.1 (0,0.2)	0 (0,0)	-88.3 (-96,-55.8)	3.4 (1.3,4.9)	0.3 (0.1,1.2)	-90.2 (-96.9,-56.1)
United States of America	1.4 (1.4,1.5)	0.5 (0.5,0.5)	-65.4 (-67.7,-62.8)	0.9 (0.8,1)	0.3 (0.2,0.3)	-72.2 (-74.4,-70.1)	27.2 (25.9,28.4)	6 (5.5,6.5)	-77.8 (-79.2,-76.4)
Australasia	1.4 (1.2,1.6)	0.5 (0.4,0.6)	-67.2 (-73.9,-58.6)	0.9 (0.8,1)	0.2 (0.1,0.2)	-78.1 (-83,-72.2)	22.9 (20.9,25.1)	4.4 (3.5,5.4)	-80.9 (-84.6,-76.6)
Australia	1.2 (1.1,1.4)	0.4 (0.3,0.6)	-64.5 (-72.8,-54.7)	0.9 (0.8,1)	0.2 (0.2,0.3)	-77.2 (-82.6,-70.4)	22.7 (20.5,25.3)	4.5 (3.6,5.5)	-80.2 (-84.3,-75.3)
New Zealand	2.3 (1.9,2.7)	0.6 (0.5,0.8)	-74.2 (-81.4,-65.7)	0.8 (0.7,0.9)	0.1 (0.1,0.2)	-84.4 (-87.6,-80.6)	24 (21,27.7)	3.7 (3,4.6)	-84.6 (-88.1,-80.5)
High-income Asia Pacific	0.9 (0.8,1)	0.3 (0.3,0.4)	-65.1 (-71.2,-55.3)	0.6 (0.5,0.7)	0.1 (0.1,0.1)	-80.6 (-83.9,-75.5)	21.3 (17.6,25)	3.4 (2.8,4.2)	-84.3 (-86.8,-79)
Brunei Darussalam	0 (0,0)	0 (0,0)	-89.1 (-96.1,-31.5)	0 (0,0)	0 (0,0)	-90.4 (-96.5,-39.1)	0 (0,0.1)	0 (0,0)	-90.4 (-96.8,-47.9)
Japan	1 (0.9,1.1)	0.3 (0.3,0.4)	-68 (-73.3,-61.9)	0.6 (0.5,0.6)	0.1 (0.1,0.1)	-80.5 (-83,-78.7)	19.6 (18.7,20.5)	3.1 (2.9,3.4)	-84 (-85.1,-82.8)
Singapore	0.9 (0.8,1)	0.4 (0.3,0.4)	-58.8 (-67.2,-47.7)	0.7 (0.6,0.8)	0.1 (0.1,0.1)	-82.2 (-85.1,-78.2)	23.2 (20.8,26.1)	4 (3.4,4.8)	-82.9 (-85.7,-79.1)
Republic of Korea	0.7 (0.4,1.1)	0.4 (0.2,0.6)	-52.5 (-71.8,6.3)	0.7 (0.4,0.9)	0.1 (0.1,0.2)	-80.9 (-88.6,-61.4)	25.2 (11.4,39)	3.8 (2.1,6.6)	-85 (-91.1,-62.3)
Western Europe	1.5 (1.4,1.7)	0.7 (0.6,0.8)	-56 (-61.6,-48.5)	0.9 (0.8,1.1)	0.2 (0.2,0.3)	-75.8 (-79.3,-71.6)	25.3 (22.9,29.3)	4.9 (4.4,5.6)	-80.5 (-82.8,-77.3)

Andorra	0.7 (0.3,1.3)	0.4 (0.2,0.8)	-40.4 (-74,40.7)	0.4 (0.2,0.7)	0.1 (0,0.2)	-70.8 (-87,-34.8)	9.9 (4.5,18.4)	2.8 (1.3,5.2)	-72 (-88,-37.2)
Austria	1.5 (1.3,1.7)	0.7 (0.6,0.9)	-49.9 (-63.1,-33.4)	0.9 (0.8,1.1)	0.2 (0.2,0.3)	-74.3 (-80.6,-66.4)	25.1 (21.8,29)	5.4 (4.3,6.6)	-78.4 (-83.7,-72.5)
Belgium	1.3 (1,1.6)	0.6 (0.5,0.8)	-52.9 (-68,-31.8)	0.8 (0.7,1)	0.2 (0.1,0.3)	-75.8 (-82.5,-67.2)	22.1 (17.7,26.3)	4.8 (3.6,6.4)	-78.2 (-84.1,-69.8)
Cyprus	0.7 (0.4,1.4)	0.5 (0.3,0.9)	-24.9 (-62.5,84.9)	0.6 (0.3,1.2)	0.2 (0.1,0.3)	-66.4 (-84.1,-14.8)	12.6 (6.5,22.2)	4 (2.2,6.5)	-68 (-83.4,-28.4)
Denmark	1 (0.8,1.1)	0.9 (0.7,1.2)	-5.2 (-30.3,27.7)	0.6 (0.5,0.7)	0.3 (0.2,0.4)	-52.1 (-64.6,-36)	15.7 (13.3,18.2)	6.5 (5,8.5)	-58.3 (-68.8,-45)
Finland	0.6 (0.5,0.7)	0.3 (0.3,0.4)	-45.6 (-58.7,-28.9)	0.5 (0.4,0.5)	0.1 (0.1,0.2)	-67.7 (-74.8,-59)	12.4 (10.6,14.3)	3.4 (2.7,4.2)	-72.3 (-78.8,-64.2)
France	1.4 (1.2,1.6)	0.7 (0.6,1)	-49.2 (-61.1,-32.2)	0.9 (0.8,1.1)	0.2 (0.2,0.3)	-73.7 (-79.3,-65.5)	26.7 (23.6,29.6)	5.4 (4.4,7)	-79.7 (-83.6,-74)
Germany	1.8 (1.4,2.6)	0.8 (0.7,1)	-53.4 (-69.1,-33.6)	1.2 (0.9,1.7)	0.3 (0.2,0.3)	-75.8 (-83.6,-65.8)	32.2 (25.2,46.4)	6.3 (5.2,7.5)	-80.5 (-86.7,-73.4)
Greece	1.8 (1.6,2)	0.9 (0.7,1)	-50.6 (-59.7,-41.2)	1 (0.9,1.1)	0.3 (0.3,0.4)	-66.5 (-71.5,-61.1)	25.8 (23.7,28)	7.4 (6.5,8.4)	-71.2 (-74.7,-67.1)
Iceland	0.6 (0.5,0.8)	0.4 (0.3,0.5)	-38.3 (-56.8,-14.7)	0.3 (0.3,0.4)	0.1 (0.1,0.1)	-68.3 (-77.3,-57.1)	8.5 (7.2,10)	2.4 (1.8,3)	-71.7 (-78.9,-62.4)
Ireland	1.3 (1.1,1.4)	0.5 (0.4,0.7)	-58 (-68.5,-42.4)	0.8 (0.7,1)	0.1 (0.1,0.2)	-82.9 (-86.8,-77.5)	21.5 (18.5,24.6)	3.5 (2.8,4.6)	-83.9 (-87.5,-78.7)
Israel	1.2 (0.9,1.5)	0.5 (0.4,0.6)	-58.1 (-69.5,-38.4)	0.8 (0.6,1.1)	0.2 (0.1,0.2)	-78.3 (-84.3,-68.3)	22.9 (17.8,29.1)	4.3 (3.4,5.4)	-81.3 (-86.1,-73.1)
Italy	1.9 (1.7,2.1)	0.7 (0.6,0.9)	-60.9 (-68.1,-52.9)	1 (0.9,1.1)	0.2 (0.2,0.3)	-77.6 (-81.1,-74.1)	31.1 (28.8,33.4)	5.2 (4.4,6.1)	-83.4 (-85.7,-81)
Luxembourg	1.2 (1.1,1.4)	0.5 (0.4,0.7)	-54.8 (-63.7,-42.8)	0.8 (0.7,0.9)	0.2 (0.1,0.2)	-78.3 (-82.6,-72.7)	22.5 (20,24.9)	3.9 (3.3,4.7)	-82.6 (-85.6,-78.7)
Malta	1.1 (0.9,1.3)	0.5 (0.4,0.7)	-50.2 (-64.5,-31.7)	0.8 (0.6,0.9)	0.2 (0.1,0.2)	-76.9 (-83.1,-68.2)	19.2 (16.2,22.7)	4.3 (3.3,5.6)	-77.8 (-83.4,-70.3)
Monaco	1.3 (0.5,2.3)	1.6 (0.7,3.1)	29.7 (-37.9,140)	0.6 (0.2,1.1)	0.5 (0.2,0.9)	-27.3 (-65.6,39.7)	18.1 (7.2,31.8)	12.3 (5.1,23.9)	-31.8 (-65.6,20.3)
Netherlands	1.2 (1,1.4)	0.7 (0.5,0.8)	-45.5 (-59.7,-28.3)	0.7 (0.6,0.7)	0.2 (0.1,0.2)	-71.9 (-78.2,-62.7)	17.6 (15.4,20)	4.3 (3.5,5.4)	-75.3 (-80.8,-69)
Norway	0.7 (0.6,0.8)	0.3 (0.3,0.4)	-57.9 (-65.4,-48.6)	0.4 (0.4,0.5)	0.1 (0.1,0.1)	-80.8 (-83.7,-77.2)	11.7 (10.7,12.9)	2 (1.7,2.4)	-83 (-85.5,-79.9)

Portugal	1.1 (0.9,1.2)	0.5 (0.4,0.7)	-48.2 (-60.4,-30.4)	0.8 (0.7,0.9)	0.2 (0.1,0.2)	-76.9 (-82.3,-70.6)	23.5 (20.2,26.6)	4.6 (3.7,5.8)	-80.4 (-84.6,-75.1)
San Marino	1 (0.5,1.7)	0.5 (0.2,1.1)	-43.8 (-74.9,21.1)	0.5 (0.3,0.9)	0.2 (0.1,0.3)	-68.7 (-85.3,-36.3)	11.9 (6.3,20.7)	3.8 (1.7,7.3)	-67.9 (-85.1,-31.6)
Spain	1.3 (1.1,1.5)	0.5 (0.4,0.7)	-60.4 (-71,-46.7)	0.8 (0.7,0.9)	0.1 (0.1,0.2)	-81.3 (-85.6,-75.4)	23.7 (20.8,26.3)	3.6 (2.8,4.6)	-84.9 (-88.3,-80.4)
Sweden	1.1 (0.9,1.3)	0.5 (0.4,0.7)	-52.8 (-64.5,-37)	0.6 (0.5,0.7)	0.1 (0.1,0.2)	-74.1 (-80.5,-66.4)	15.9 (13.3,18.7)	3.5 (2.8,4.4)	-78 (-83.2,-71.8)
Switzerland	1.5 (1.1,2.1)	0.6 (0.5,0.8)	-57.4 (-71.6,-37.1)	0.7 (0.6,1)	0.2 (0.1,0.2)	-76.6 (-84.1,-67)	20.7 (16.5,28.5)	4 (3.2,5.2)	-80.5 (-86.4,-72.5)
United Kingdom	1.4 (1.2,1.5)	0.5 (0.4,0.5)	-65.9 (-69.2,-60.9)	0.6 (0.6,0.7)	0.1 (0.1,0.2)	-77.1 (-79.3,-74)	17.1 (15.8,18.6)	3.7 (3.4,4)	-78.5 (-80.4,-75.7)
Southern Latin America	0.9 (0.7,1.3)	0.4 (0.3,0.4)	-61.2 (-73.4,-46)	0.9 (0.7,1.3)	0.3 (0.2,0.3)	-69.1 (-79.2,-56.3)	26.6 (20.5,37.8)	6.9 (5.7,8.4)	-74 (-82.5,-63.1)
Argentina	1 (0.7,1.5)	0.3 (0.3,0.4)	-65.8 (-79.1,-48.3)	0.9 (0.7,1.4)	0.3 (0.2,0.3)	-70.6 (-81.9,-56)	28 (20.3,42.8)	6.9 (5.5,8.6)	-75.5 (-85,-62.1)
Chile	0.8 (0.7,1)	0.4 (0.3,0.5)	-49.3 (-61,-33.6)	0.8 (0.6,0.9)	0.3 (0.2,0.3)	-65.4 (-73.1,-54.7)	22.1 (18.2,26.2)	6.5 (5.2,7.9)	-70.8 (-77.2,-62.6)
Uruguay	1.1 (0.9,1.4)	0.5 (0.4,0.6)	-53.5 (-66.5,-33.7)	1 (0.8,1.3)	0.4 (0.3,0.5)	-63.1 (-73.3,-47.9)	31.2 (25.4,39.2)	10 (8.1,12.8)	-67.8 (-76.3,-54.9)
Eastern Europe	0.6 (0.4,0.8)	0.4 (0.3,0.5)	-28.7 (-50.7,-5.7)	0.5 (0.4,0.7)	0.3 (0.2,0.3)	-46.5 (-63.7,-29.3)	16.5 (12.6,23.5)	7.2 (6,8.9)	-56.3 (-69.7,-41.5)
Belarus	0.4 (0.3,0.8)	0.4 (0.3,0.6)	-4.5 (-49,83.5)	0.4 (0.2,0.7)	0.2 (0.2,0.3)	-37.7 (-66.5,19.8)	13.1 (8,21.9)	6.9 (4.9,9)	-47.3 (-70.3,-4.6)
Estonia	0.9 (0.6,1.2)	0.5 (0.3,0.6)	-47 (-63.4,-17.9)	0.7 (0.5,1)	0.2 (0.2,0.3)	-68.5 (-77.8,-52.5)	23.9 (16.8,32.2)	6.5 (4.9,8.8)	-72.7 (-80.8,-59.2)
Latvia	0.8 (0.6,1.1)	0.5 (0.3,0.6)	-41 (-61.6,-4.8)	0.7 (0.5,1)	0.3 (0.2,0.4)	-58.7 (-73.6,-33.7)	19.9 (14.7,27.9)	8.4 (6.2,11.2)	-57.7 (-72.6,-33.1)
Lithuania	0.9 (0.7,1.2)	0.5 (0.4,0.7)	-43 (-61.7,-13.7)	0.8 (0.5,1)	0.3 (0.3,0.4)	-56.3 (-71,-34.4)	24.9 (18.2,32.6)	9.4 (7.3,12.3)	-62.3 (-73.9,-44.6)
Republic of Moldova	0.2 (0.2,0.3)	0.1 (0.1,0.1)	-54.1 (-67.7,-35.3)	0.2 (0.2,0.3)	0.1 (0.1,0.1)	-65.3 (-75.2,-51.1)	6.3 (5,8.3)	2.2 (1.8,2.6)	-65.7 (-75.3,-51.4)
Russian Federation	0.5 (0.3,0.7)	0.4 (0.3,0.5)	-20.4 (-44.9,5.9)	0.4 (0.3,0.6)	0.2 (0.2,0.3)	-41.8 (-60.1,-22.5)	14.6 (10.4,20.8)	6.6 (5.7,7.7)	-54.6 (-68.3,-39.6)
Ukraine	0.7 (0.5,1.1)	0.4 (0.3,0.7)	-41.4 (-68.1,4.4)	0.6 (0.4,0.9)	0.3 (0.2,0.5)	-51.9 (-73.2,-16.2)	22.5 (16.3,33.5)	9.6 (5.6,15.6)	-57.4 (-76.3,-24.5)

Central Europe	0.7 (0.6,0.9)	0.3 (0.3,0.4)	-55.3 (-63.8,-42.1)	0.7 (0.6,0.8)	0.2 (0.2,0.2)	-71.2 (-76.7,-62.3)	19.5 (16.6,24)	4.8 (4.2,5.6)	-75.3 (-80,-67.1)
Albania	0.3 (0.1,0.7)	0.3 (0.1,0.6)	-4.8 (-58.5,146.3)	0.3 (0.1,0.7)	0.2 (0.1,0.4)	-31.1 (-69.4,70.6)	8.5 (2.6,19.8)	5 (1.5,10.6)	-41.3 (-73.7,49.1)
Bosnia and Herzegovina	0.3 (0.1,0.9)	0.3 (0.1,0.8)	5 (-45.5,155.2)	0.3 (0.1,0.8)	0.2 (0.1,0.5)	-24 (-59.2,78.6)	8.6 (2.5,22.5)	5.7 (1.6,12.6)	-33.3 (-64.9,54.1)
Bulgaria	0.5 (0.4,0.8)	0.3 (0.2,0.4)	-49.4 (-70.9,-15.5)	0.5 (0.3,0.7)	0.2 (0.1,0.3)	-59.7 (-77.5,-33.6)	15.7 (11.2,21.2)	5.6 (3.8,8.1)	-64.4 (-78.1,-45.4)
Croatia	0.9 (0.7,1.2)	0.4 (0.3,0.6)	-55.2 (-72,-23.9)	0.7 (0.5,1)	0.2 (0.1,0.3)	-74.3 (-84.4,-57.1)	18.5 (13.7,24.5)	4.7 (3.1,6.6)	-74.8 (-83.9,-59.7)
Czechia	0.5 (0.4,0.7)	0.4 (0.3,0.6)	-28.2 (-50.1,4.2)	0.5 (0.4,0.6)	0.2 (0.1,0.3)	-58.7 (-70.6,-39.4)	13.2 (10.7,15.8)	4.7 (3.3,6.4)	-64.5 (-74.3,-50.2)
Hungary	1.1 (0.9,1.2)	0.6 (0.4,0.7)	-47.5 (-63.2,-28)	0.9 (0.8,1.1)	0.3 (0.2,0.4)	-67.4 (-76.7,-56.1)	28.2 (24.2,32.5)	8.2 (6.2,10.9)	-70.8 (-79.5,-60.5)
Montenegro	0.5 (0.1,1.1)	0.4 (0.1,0.7)	-21.3 (-65.2,65.2)	0.4 (0.1,0.8)	0.2 (0.1,0.4)	-39.3 (-74.8,29.6)	10.6 (3.3,22.1)	5.4 (2.2,9.9)	-48.7 (-76.3,1.8)
North Macedonia	0 (0,0)	0 (0,0)	-31.9 (-87.9,51.3)	0 (0,0)	0 (0,0)	-48.7 (-89.9,11.3)	0 (0,0)	0 (0,0)	-66.6 (-96.1,8)
Poland	1.1 (0.8,1.5)	0.3 (0.3,0.4)	-70.2 (-78.1,-56.5)	1 (0.8,1.4)	0.2 (0.2,0.2)	-79.9 (-85.2,-71.5)	30 (22,39.6)	4.9 (4.3,5.6)	-83.6 (-88,-75.9)
Romania	0.4 (0.3,0.5)	0.2 (0.2,0.2)	-49 (-65.6,-26.5)	0.4 (0.3,0.5)	0.1 (0.1,0.2)	-65.1 (-76.6,-49.2)	12.6 (9.8,17.2)	3.5 (2.9,4.3)	-72.1 (-81,-59.9)
Serbia	0.4 (0.2,0.6)	0.3 (0.2,0.5)	-35.5 (-62.1,9.6)	0.4 (0.2,0.6)	0.2 (0.1,0.3)	-58.8 (-75.5,-31)	11.1 (6.8,17.9)	4 (2.4,7.2)	-64.2 (-79.2,-36.8)
Slovakia	0.3 (0.2,0.5)	0.2 (0.1,0.4)	-37 (-69.2,38.5)	0.3 (0.1,0.4)	0.1 (0.1,0.2)	-58.7 (-79.7,-10.6)	7.6 (3.9,13.4)	2.9 (1.3,5.6)	-61.5 (-81.1,-15.4)
Slovenia	1.2 (1,1.5)	0.9 (0.7,1.3)	-23.1 (-44.7,6.9)	0.9 (0.8,1.1)	0.4 (0.3,0.5)	-61.3 (-72,-48.3)	26.5 (22,31.6)	8.6 (6.5,11.1)	-67.4 (-75.6,-56.2)
Central Asia	0.4 (0.3,0.6)	0.3 (0.2,0.3)	-42.8 (-61.5,-9.4)	0.4 (0.3,0.6)	0.2 (0.2,0.3)	-50.2 (-66.4,-21.3)	16.7 (12.4,23.1)	7.6 (5.7,10.2)	-54.5 (-68.9,-27.9)
Armenia	0.8 (0.5,1.1)	0.3 (0.3,0.4)	-56.2 (-70.6,-32.9)	0.7 (0.5,1)	0.2 (0.2,0.3)	-65.3 (-76.8,-46.5)	26 (18.8,36.4)	7.3 (6,9)	-71.7 (-80.4,-56.9)
Azerbaijan	0.4 (0.2,0.9)	0.3 (0.1,0.7)	-22 (-70.5,122.4)	0.4 (0.2,0.8)	0.2 (0.1,0.5)	-33.9 (-74.7,80.3)	15.4 (7.4,30.8)	9.5 (3.6,21.4)	-38.3 (-74.9,61.8)
Georgia	0.3 (0.2,0.4)	0.3 (0.2,0.4)	1.4 (-43.5,83.5)	0.2 (0.1,0.3)	0.2 (0.1,0.3)	-8 (-48,67.7)	7.9 (4.9,12.5)	6.5 (4.6,8.8)	-17.9 (-52.5,45)

Kazakhstan	0.5 (0.3,0.8)	0.3 (0.2,0.4)	-43.3 (-68.3,4.9)	0.4 (0.3,0.7)	0.2 (0.1,0.3)	-54.2 (-74.9,-14.1)	16.4 (11.1,26.3)	6.4 (4.8,8.8)	-61 (-78,-27.1)
Kyrgyzstan	0.3 (0.2,0.4)	0.2 (0.2,0.3)	-35.4 (-56,-2.6)	0.3 (0.2,0.4)	0.2 (0.1,0.2)	-45.5 (-62.4,-19.1)	13.4 (10.2,17.2)	6.3 (4.8,8.3)	-52.8 (-67.5,-29.8)
Mongolia	0.4 (0.1,0.8)	0.2 (0.1,0.3)	-52.7 (-83.3,26.1)	0.4 (0.1,0.8)	0.2 (0.1,0.3)	-57.8 (-84.9,10.7)	15.1 (5.8,31.9)	5.6 (2.5,9.8)	-63 (-87.2,-4.8)
Tajikistan	0.4 (0.2,0.7)	0.2 (0.1,0.4)	-50.6 (-81.5,19.3)	0.4 (0.1,0.7)	0.2 (0.1,0.3)	-53.1 (-82.1,11.7)	15.4 (6.6,29.8)	6.8 (2.5,14.6)	-55.6 (-84.1,6.2)
Turkmenistan	0.4 (0.3,0.6)	0.3 (0.2,0.4)	-32 (-58.3,12.1)	0.4 (0.2,0.6)	0.2 (0.1,0.3)	-41.1 (-63.6,-3.9)	16.2 (10.1,25.5)	9.3 (5.7,14)	-42.5 (-65.1,-2.4)
Uzbekistan	0.5 (0.3,0.8)	0.2 (0.2,0.3)	-54.7 (-77.2,-11.2)	0.5 (0.3,0.8)	0.2 (0.1,0.3)	-58.9 (-79.4,-19.8)	19.6 (12.4,30.9)	7.7 (5.3,11.1)	-60.5 (-79,-26.7)
Central Latin America	0.6 (0.5,0.7)	0.3 (0.3,0.4)	-44.1 (-54.1,-33.8)	0.6 (0.5,0.7)	0.3 (0.2,0.3)	-53.4 (-61.8,-45.1)	19.3 (17.8,21.7)	8.2 (7.1,9.5)	-57.4 (-65.3,-49.4)
Colombia	0.7 (0.6,0.8)	0.4 (0.3,0.5)	-47.1 (-60.4,-31.9)	0.7 (0.6,0.8)	0.3 (0.2,0.4)	-59.5 (-69.2,-47.7)	22.6 (19.8,25.9)	8.4 (6.5,10.6)	-62.8 (-71.8,-52.3)
Costa Rica	1.1 (0.8,1.4)	0.6 (0.5,0.7)	-45 (-61.7,-22.5)	1 (0.8,1.3)	0.4 (0.3,0.5)	-59.1 (-71.4,-41.7)	28.3 (22.7,35.7)	11.9 (9.6,14.6)	-58.1 (-70.3,-42.2)
El Salvador	0.2 (0.1,0.3)	0.2 (0.1,0.3)	-19.3 (-51.9,24.7)	0.2 (0.1,0.3)	0.2 (0.1,0.2)	-34.6 (-60.6,-0.5)	8.4 (4.7,12.2)	4.9 (2.8,7.2)	-41.2 (-63.6,-7.2)
Guatemala	0.3 (0.2,0.5)	0.2 (0.1,0.2)	-54.2 (-70.5,-29.8)	0.4 (0.3,0.5)	0.1 (0.1,0.2)	-58.9 (-74,-36)	12.3 (8.6,18.4)	4.8 (4,5.7)	-61.2 (-76,-39.4)
Honduras	0.6 (0.3,1)	0.5 (0.3,0.9)	-21.9 (-61.6,39.6)	0.6 (0.4,1)	0.5 (0.3,0.8)	-24 (-64,33.1)	24.7 (11.6,40.4)	14.7 (7.9,24.9)	-40.6 (-68.8,3.7)
Mexico	0.5 (0.5,0.5)	0.3 (0.2,0.3)	-44.9 (-54.9,-34.3)	0.5 (0.5,0.5)	0.2 (0.2,0.3)	-53.6 (-61.6,-45.2)	17 (16.1,17.8)	7.3 (6,8.6)	-57.1 (-64.9,-48.7)
Nicaragua	0.5 (0.3,0.8)	0.4 (0.2,0.6)	-26.9 (-51.5,9.6)	0.5 (0.3,0.7)	0.3 (0.2,0.5)	-36.7 (-57.9,-5.8)	19.3 (12.2,28.1)	10.2 (6.5,16)	-47.2 (-65.5,-18.9)
Panama	0.5 (0.4,0.7)	0.3 (0.3,0.4)	-36.3 (-56.6,-10.2)	0.5 (0.4,0.6)	0.2 (0.2,0.3)	-50.1 (-65.9,-29.9)	16.9 (13.8,21.7)	7.8 (6.2,9.7)	-54.1 (-68.5,-34.5)
Venezuela (Bolivarian Republic of)	0.8 (0.7,1.1)	0.5 (0.3,0.6)	-45.5 (-69.2,-18.7)	0.8 (0.7,1.1)	0.4 (0.3,0.5)	-52.8 (-73.4,-29.3)	27.4 (22.7,36.4)	12 (8,17)	-56.4 (-76.2,-33.5)
Andean Latin America	0.4 (0.3,0.6)	0.4 (0.2,0.5)	-10.3 (-42.5,44.8)	0.4 (0.3,0.6)	0.3 (0.2,0.4)	-25.8 (-52.2,15.7)	14.3 (8.7,21.8)	9.2 (5.8,12.8)	-35.8 (-59.4,1.8)

Bolivia (Plurinational State of)	0.8 (0.4,1.4)	0.5 (0.3,0.8)	-36.3 (-63.5,11.8)	0.8 (0.4,1.4)	0.5 (0.3,0.9)	-39.3 (-65,3.4)	29.4 (13.3,54.9)	14.9 (8,24.3)	-49.4 (-72.1,-3.3)
Ecuador	0.3 (0.2,0.4)	0.3 (0.2,0.4)	16.4 (-32,77.7)	0.3 (0.2,0.4)	0.3 (0.2,0.3)	-0.3 (-41.3,50.9)	8.8 (6.6,13.4)	7.9 (5.9,10.3)	-9.6 (-47.8,36.1)
Peru	0.3 (0.2,0.5)	0.3 (0.2,0.6)	-0.6 (-48.9,87.7)	0.3 (0.2,0.6)	0.3 (0.1,0.4)	-24.1 (-61.7,42.4)	12.5 (7.2,20.1)	8.2 (4.2,13.4)	-34.5 (-64,18.8)
Caribbean	0.9 (0.7,1.3)	0.6 (0.5,0.8)	-35.7 (-50.2,-18.2)	0.9 (0.7,1.2)	0.5 (0.4,0.6)	-45.2 (-57.3,-29.9)	28.8 (20.1,51.3)	15.1 (10.7,24.1)	-47.7 (-63.1,-28.5)
Antigua and Barbuda	1.2 (1,1.4)	0.9 (0.8,1)	-22.2 (-37,-4)	1.2 (1,1.3)	0.8 (0.7,0.9)	-31.8 (-43.9,-16)	33.7 (29,38.3)	19.6 (17.5,22.3)	-41.8 (-52,-27)
Barbados	0.4 (0.3,0.6)	0.4 (0.3,0.5)	-7.7 (-36.3,33.4)	0.4 (0.3,0.6)	0.3 (0.3,0.4)	-20.1 (-45.2,13.2)	12.4 (10,16.3)	8.6 (6.4,11)	-30.8 (-52.2,-0.8)
Belize	0.3 (0.2,0.4)	0.2 (0.2,0.3)	-19.8 (-44.1,17.6)	0.3 (0.2,0.4)	0.2 (0.2,0.3)	-27.3 (-49.1,6.8)	9.6 (7.3,12.5)	6.4 (5.3,7.8)	-33.4 (-54.3,-1.2)
Bermuda	0 (0,0)	0 (0,0)	-78.3 (-94.1,-26.8)	0 (0,0)	0 (0,0)	-88.8 (-96.4,-64.9)	0 (0,0.1)	0 (0,0)	-90.8 (-98,-65.7)
Bahamas	0.7 (0.5,1)	0.4 (0.3,0.5)	-42.9 (-64.2,-13.4)	0.6 (0.5,0.9)	0.3 (0.2,0.4)	-48.9 (-68.2,-23.4)	22.9 (16.9,32.7)	10.8 (8.1,14.2)	-52.6 (-70,-27.2)
Cuba	1.2 (1.1,1.5)	0.9 (0.7,1.1)	-28.8 (-46.8,-7.3)	1.1 (1,1.4)	0.6 (0.5,0.8)	-45.5 (-59.3,-30.9)	32.7 (28.5,39.6)	16.5 (13.5,20.1)	-49.6 (-62.6,-35.6)
Dominica	0 (0,0)	0 (0,0)	-11 (-58.8,84.6)	0 (0,0)	0 (0,0)	-15.9 (-61.1,79.3)	0 (0,0)	0 (0,0)	-12.5 (-61.7,80.3)
Dominican Republic	0.2 (0.1,0.4)	0.2 (0.1,0.3)	-33.9 (-63.1,19.2)	0.2 (0.1,0.4)	0.1 (0.1,0.3)	-39.2 (-65.7,6.8)	7.7 (4.2,12.7)	4.2 (2.4,7.3)	-45.9 (-69.7,-1.5)
Grenada	0.7 (0.5,0.9)	0.4 (0.3,0.4)	-46.6 (-62.8,-14.4)	0.7 (0.5,0.9)	0.3 (0.3,0.4)	-51.7 (-66.8,-23.2)	26 (18,34.7)	10.5 (8.7,12.4)	-59.7 (-72.3,-34.3)
Guyana	0.6 (0.5,0.8)	0.7 (0.4,1)	5.6 (-29.8,62)	0.6 (0.5,0.8)	0.6 (0.4,0.9)	-0.6 (-35.1,48.7)	20.6 (17,25.5)	20.7 (14,30.1)	0.5 (-33.2,51.5)
Haiti	1.3 (0.5,2.7)	0.8 (0.3,1.4)	-40.9 (-66.2,5.7)	1.3 (0.5,2.7)	0.8 (0.3,1.4)	-41 (-65.9,2.8)	51.6 (14.5,145.6)	26 (9.7,55)	-49.5 (-72.8,-1.7)
Jamaica	0.2 (0.2,0.3)	0.3 (0.2,0.4)	22 (-26.5,104.5)	0.2 (0.2,0.3)	0.3 (0.2,0.4)	7.4 (-35.8,78.6)	7.4 (5.1,10.1)	7.3 (5,10.5)	-0.9 (-40.7,64.3)
Puerto Rico	0.8 (0.7,1.1)	0.4 (0.3,0.5)	-51 (-66,-28.8)	0.8 (0.6,1)	0.3 (0.2,0.3)	-66.4 (-76.4,-52)	21.7 (17.3,27.3)	6.5 (4.8,8.3)	-70.3 (-79.4,-56.8)

Saint Kitts and Nevis	1.1 (0.8,1.5)	0.4 (0.4,0.6)	-59.9 (-73.8,-35.6)	1.1 (0.8,1.6)	0.4 (0.3,0.5)	-63.5 (-76.2,-41.8)	37.9 (27.1,52.3)	11.2 (8.7,14.2)	-70.5 (-80.9,-50.6)
Saint Lucia	1.3 (1,1.6)	0.5 (0.4,0.6)	-64.8 (-73.4,-51.6)	1.4 (1.1,1.6)	0.4 (0.3,0.5)	-69.2 (-76.9,-57.8)	40.2 (31.3,49.3)	12 (9.3,14.8)	-70 (-77.8,-58.3)
Saint Vincent and the Grenadines	1.2 (1,1.3)	0.7 (0.6,0.8)	-42.9 (-53.8,-30.4)	1.2 (1,1.3)	0.6 (0.5,0.7)	-47.1 (-57.5,-35.7)	38.7 (33.9,43.9)	19 (15.9,22.7)	-50.8 (-60.5,-39.4)
Suriname	0.7 (0.4,1)	0.4 (0.3,0.7)	-38.2 (-65.5,3.3)	0.7 (0.5,1)	0.4 (0.2,0.6)	-42.5 (-67.4,-3.1)	23.4 (14.2,32.9)	13.4 (7.9,20.4)	-42.9 (-68.1,-7.1)
Trinidad and Tobago	0.9 (0.8,1.1)	0.6 (0.4,0.7)	-39.9 (-58.4,-8.7)	0.9 (0.8,1.1)	0.5 (0.4,0.6)	-48.4 (-63.9,-22.3)	27.1 (22,32.2)	14.1 (10.4,18.8)	-48 (-64.4,-18.7)
United States Virgin Islands	0.6 (0.4,0.9)	0.2 (0.1,0.4)	-63.2 (-82.3,-26.4)	0.6 (0.4,0.9)	0.2 (0.1,0.3)	-68.4 (-84.6,-39.7)	17.5 (10.8,25.9)	5.4 (2.4,9.8)	-69.3 (-85.5,-35.8)
Tropical Latin America	0.7 (0.6,0.8)	0.3 (0.2,0.3)	-59.4 (-64.6,-53.4)	0.7 (0.6,0.8)	0.3 (0.2,0.3)	-63.5 (-68.3,-58)	22.2 (20.4,24.1)	6.8 (6,7.8)	-69.5 (-73.2,-65.2)
Brazil	0.7 (0.6,0.8)	0.3 (0.2,0.3)	-60.1 (-65.4,-54.1)	0.7 (0.6,0.8)	0.2 (0.2,0.3)	-64 (-68.9,-58.6)	22.2 (20.4,24)	6.6 (5.9,7.6)	-70.1 (-73.8,-65.8)
Paraguay	0.7 (0.4,1)	0.5 (0.3,0.7)	-31.1 (-64,10.2)	0.7 (0.4,1.1)	0.4 (0.3,0.6)	-39.3 (-68.2,-1)	21.3 (12.3,32)	11.7 (7.1,18.2)	-44.9 (-70.9,-9.4)
East Asia	0.4 (0.2,0.6)	0.2 (0.1,0.3)	-51.5 (-73.3,-2.5)	0.3 (0.1,0.6)	0.1 (0.1,0.1)	-75.8 (-86.9,-49.6)	14.9 (5.8,24.8)	3.1 (2,5.2)	-79.5 (-89.2,-54.8)
China	0.4 (0.2,0.6)	0.2 (0.1,0.3)	-52.3 (-74.2,-3.2)	0.3 (0.1,0.6)	0.1 (0.1,0.1)	-76.6 (-87.8,-49.9)	15 (5.6,25.1)	3 (1.9,5.2)	-80.3 (-89.7,-55)
Democratic People's Republic of Korea	0.3 (0.2,0.6)	0.2 (0.1,0.4)	-37.3 (-75.1,10)	0.3 (0.2,0.6)	0.2 (0.1,0.4)	-46.6 (-78.3,-5)	11.8 (6,21.6)	6.1 (2.4,14.8)	-48.4 (-80.3,-4.8)
Taiwan (Province of China)	0.3 (0.2,0.7)	0.3 (0.2,0.4)	-14.2 (-59.5,129.8)	0.3 (0.1,0.6)	0.1 (0.1,0.2)	-51.3 (-76.9,28.3)	9.6 (4.3,20.7)	4.1 (3.4,5.1)	-57 (-80.3,14.1)
Southeast Asia	0.7 (0.4,1)	0.4 (0.3,0.7)	-40.4 (-58.2,2.1)	0.7 (0.4,1)	0.4 (0.2,0.6)	-46.2 (-62.3,-9.5)	26.1 (13.6,41.9)	12.6 (8.4,20.9)	-51.6 (-67.8,-11.6)
Cambodia	1.3 (0.6,2.4)	0.9 (0.5,1.8)	-30 (-72.2,61.6)	1.4 (0.6,2.5)	0.9 (0.4,1.7)	-34 (-73,54)	52.1 (20.4,102.6)	30.2 (14.7,60)	-42 (-78.6,41.2)
Indonesia	0.8 (0.4,1.3)	0.4 (0.2,0.8)	-42.8 (-68.4,4.4)	0.8 (0.4,1.3)	0.4 (0.1,0.8)	-45.8 (-70.2,-4.4)	29.6 (13.5,51.5)	13.7 (5.1,26.3)	-53.8 (-74.5,-6.5)

Lao People's Democratic Republic	1.6 (0.6,3)	0.8 (0.4,1.7)	-49.8 (-78.2,11.6)	1.7 (0.6,3.1)	0.8 (0.4,1.7)	-51.6 (-78.8,6.4)	64.1 (21,137)	28.7 (13.4,63.1)	-55.3 (-82.1,10.8)
Malaysia	0.2 (0.1,0.4)	0.1 (0.1,0.2)	-30.8 (-69.1,30.1)	0.2 (0.1,0.4)	0.1 (0.1,0.2)	-40.7 (-73.5,9.2)	6.4 (3.5,12)	3.2 (2,5.6)	-48.9 (-77.4,-1)
Maldives	1 (0.4,1.9)	0.3 (0.2,0.6)	-67 (-87,2.4)	1 (0.4,2)	0.3 (0.2,0.4)	-73.9 (-89.4,-23)	38.4 (13.1,83.1)	7.6 (4.4,13.2)	-80.3 (-92.6,-29.7)
Mauritius	1.3 (1.1,1.5)	0.4 (0.3,0.4)	-71 (-77.3,-62.3)	1.2 (1,1.4)	0.3 (0.3,0.3)	-75.4 (-80.5,-68.4)	43.5 (36.2,49.7)	9.5 (8.2,10.7)	-78.2 (-82.7,-72.1)
Myanmar	1.5 (0.5,2.6)	0.6 (0.4,1.2)	-56.5 (-77.8,-3.5)	1.5 (0.5,2.5)	0.6 (0.3,1.2)	-58.5 (-78.9,-8.4)	59.3 (20.3,122.1)	21.3 (12.1,41.7)	-64 (-83.4,-12.2)
Philippines	0.7 (0.5,1)	0.6 (0.4,0.9)	-21.1 (-46.4,21)	0.7 (0.5,1.1)	0.5 (0.4,0.9)	-26.7 (-50.5,12.6)	26 (15.7,38.7)	18.5 (12.4,29)	-28.8 (-51.1,8.5)
Sri Lanka	0.4 (0.1,0.7)	0.3 (0.1,0.5)	-23.9 (-63.5,238)	0.4 (0.1,0.7)	0.2 (0.1,0.4)	-43 (-71.9,144.4)	12 (3.7,21.9)	6.2 (3.3,11.3)	-48.2 (-76.2,105.1)
Seychelles	1.4 (0.7,2.1)	0.9 (0.5,1.4)	-31.9 (-63.8,13)	1.3 (0.7,2.1)	0.8 (0.5,1.1)	-40.4 (-67.8,-1.7)	49.4 (26.6,76.4)	25.7 (14.8,38.6)	-47.9 (-70.8,-12.4)
Thailand	0.7 (0.3,1.2)	0.4 (0.2,0.7)	-39.1 (-68.3,41.5)	0.7 (0.3,1.2)	0.3 (0.2,0.5)	-55.7 (-76.1,-5)	22.5 (10.9,34.9)	9.8 (5.5,16.2)	-56.6 (-76.5,-4.8)
Timor-Leste	1 (0.4,1.8)	0.6 (0.3,1.2)	-36.9 (-68.5,21.2)	1 (0.4,1.8)	0.6 (0.3,1.2)	-38.8 (-70.1,15.3)	38.7 (13.7,83.2)	20.5 (10.7,38.7)	-47 (-78.6,12.7)
Viet Nam	0.1 (0,0.2)	0.1 (0,0.2)	-18.2 (-67.8,99.1)	0.1 (0.1,0.2)	0.1 (0,0.1)	-34.8 (-73.8,60.6)	3.1 (1.5,5.7)	2 (1,4.3)	-34.5 (-75.1,61.2)
Oceania	1.8 (1.1,2.8)	1.7 (1,2.5)	-6.2 (-28.7,25.2)	1.8 (1.1,2.8)	1.6 (0.9,2.4)	-9.8 (-31.2,19.9)	63 (37.5,101.2)	59.4 (33.8,90.3)	-5.8 (-30.8,30.1)
American Samoa	0.5 (0.1,2.3)	2.2 (1,3.1)	384.1 (-33.1,2478.2)	0.5 (0.1,2.1)	1.9 (0.9,2.7)	328.7 (-37.1,2137)	13.5 (2.8,66.1)	66.7 (26.3,95.3)	392.5 (-34.1,2670.3)
Cook Islands	0.7 (0.5,1.1)	0.5 (0.2,0.9)	-32.6 (-71.6,38.9)	0.7 (0.5,1)	0.3 (0.2,0.6)	-51.1 (-77.4,-1.4)	22.2 (14.6,35.8)	10.8 (4.4,18.7)	-51.3 (-80.1,7)
Micronesia (Federated States of)	2.7 (1.7,4)	2.4 (1.5,3.5)	-8.1 (-39.8,46)	2.7 (1.7,3.9)	2.3 (1.4,3.3)	-13.2 (-42.9,37.3)	91.7 (56.4,143.3)	79.6 (47.9,117.1)	-13.1 (-45.5,43.7)
Fiji	1.1 (0.5,1.9)	0.7 (0.3,1.2)	-34.4 (-64.4,21.4)	1.1 (0.5,1.8)	0.7 (0.3,1.1)	-36.7 (-65.9,15.5)	35.5 (15.3,58)	21.5 (9.6,35)	-39.4 (-67.9,13.5)
Guam	2.1 (1.6,3.2)	1.9 (1.3,2.4)	-8.2 (-53.2,25.3)	1.9 (1.5,2.9)	1.4 (0.9,1.7)	-28.4 (-61.6,-2.1)	57.5 (44.4,92.6)	48.7 (31.7,61)	-15.3 (-58.2,19)

Kiribati	1.6 (1,2.8)	1.6 (1.1,2.5)	2.7 (-29.1,65.9)	1.6 (1,2.9)	1.6 (1.1,2.5)	1.1 (-29.7,60.6)	58.4 (35.6,101.8)	57.1 (39.9,88.9)	-2.3 (-35.1,64.4)
Marshall Islands	2.1 (1.4,2.8)	2.3 (1.2,3.5)	9.6 (-34.5,62.6)	2.1 (1.4,2.9)	2.2 (1.2,3.4)	4.7 (-37.2,55.4)	71.7 (48,101.1)	79.2 (41,124.8)	10.5 (-34.7,70.2)
Nauru	3.1 (2,4.5)	3.1 (1.7,4.6)	1.5 (-33.5,54.9)	3 (2,4.4)	2.9 (1.7,4.3)	-4.2 (-36.4,44.9)	105 (66,164.3)	104.1 (55.7,159.2)	-0.9 (-37.1,55.6)
Niue	1.7 (1.1,2.6)	2.2 (1.4,3)	30.8 (-17.8,102.7)	1.7 (1.1,2.5)	1.9 (1.1,2.5)	12.6 (-29.3,72.2)	54.4 (33.1,85.8)	79.8 (45.2,115)	46.7 (-12.1,137.7)
Northern Mariana Islands	2.1 (1.4,3.5)	1.5 (1.1,2.2)	-26.7 (-49.3,5.4)	1.9 (1.4,3.1)	1.2 (1,1.8)	-35.2 (-54.7,-8.6)	56.4 (37.4,103.6)	36.5 (27.5,55.3)	-35.4 (-56.5,-4.6)
Palau	1.2 (0.7,2)	1.1 (0.7,1.8)	-9.5 (-37.7,29.7)	1.2 (0.7,2)	1 (0.7,1.7)	-14.7 (-41.5,23)	45.7 (27.3,72.6)	34.4 (19.3,55)	-24.7 (-47.1,10)
Papua New Guinea	1.9 (1.1,3.2)	1.7 (1,2.8)	-8.5 (-38.8,39.4)	1.9 (1.1,3.1)	1.7 (1,2.7)	-10.8 (-40.2,34.4)	68.6 (38,116.3)	61.9 (33.3,99.8)	-9.7 (-40.1,39)
Samoa	2.7 (1.8,4.4)	3.2 (2,4.6)	17.2 (-32,93.5)	2.8 (1.8,4.3)	3 (1.8,4.2)	7 (-37,74.3)	83.8 (54.3,134.5)	92.3 (58.2,133.3)	10.2 (-38.1,88.3)
Solomon Islands	2.1 (1,3.3)	2.3 (1.4,3.3)	11.5 (-25.1,80.4)	2.1 (1,3.4)	2.2 (1.3,3.2)	6.4 (-27.8,67.7)	71.1 (31,120.6)	80 (47.1,115.8)	12.6 (-24.4,90.7)
Tokelau	2.2 (1.5,3.2)	2.4 (1.5,3.2)	10.5 (-26.6,64.7)	2.2 (1.5,3.1)	2.1 (1.3,2.7)	-4.9 (-36.4,39)	73.9 (48,109.5)	87.9 (52,117.9)	19 (-25.8,85.9)
Tonga	0.7 (0.5,1.1)	0.7 (0.5,1)	-0.3 (-36.6,62)	0.7 (0.5,1.1)	0.6 (0.4,0.9)	-8.3 (-41.3,48.3)	23.1 (14.9,37.2)	20.9 (13.2,29.7)	-9.4 (-44.8,50.1)
Tuvalu	2.6 (1.7,3.6)	2.1 (1.3,3)	-16.9 (-41.6,29.5)	2.6 (1.7,3.6)	2 (1.2,2.7)	-22.2 (-45.2,17.8)	90.3 (55.9,132.5)	68.3 (41.9,98.7)	-24.3 (-49.3,22.2)
Vanuatu	1.7 (1.1,2.5)	1.8 (1.1,2.5)	6.3 (-26.2,59.8)	1.7 (1.1,2.6)	1.8 (1.1,2.4)	2.4 (-28,50.4)	58.3 (35.5,88.8)	62.4 (37,85.3)	7.1 (-27.7,67.8)
North Africa and Middle East	0.7 (0.4,1.3)	0.6 (0.3,0.7)	-25 (-54.2,18.9)	0.8 (0.4,1.3)	0.5 (0.2,0.6)	-39.4 (-63.9,-8.1)	26.4 (13.2,44.4)	14.4 (6.6,19.6)	-45.4 (-67.5,-11.6)
Afghanistan	1.8 (0.5,4.2)	1.4 (0.5,3.1)	-22.5 (-58.5,43)	1.8 (0.5,4.4)	1.4 (0.4,3)	-25.1 (-59.7,39.5)	74.6 (18.8,182.8)	52.4 (17.7,109.7)	-29.8 (-64,30.5)
Algeria	0.3 (0.1,0.5)	0.2 (0.1,0.3)	-30.2 (-63.5,32.8)	0.3 (0.1,0.6)	0.2 (0.1,0.3)	-41.8 (-69.7,11.4)	10.5 (3.9,17.5)	4.9 (1.8,7.8)	-53.2 (-75.7,-0.8)
Bahrain	0.6 (0.2,1.4)	0.3 (0.1,0.5)	-54.7 (-79.5,-4.5)	0.6 (0.2,1.3)	0.2 (0.1,0.4)	-66.4 (-84.2,-31)	17.3 (6.4,37.3)	4.9 (2.1,7.7)	-71.4 (-86.6,-35.5)
Egypt	0.4 (0.1,1.4)	0.4 (0.1,1)	5.2 (-50.5,129.7)	0.4 (0.1,1.5)	0.4 (0.1,1)	-6 (-57.8,103)	15.1 (6.5,38.2)	10.9 (2.7,22.2)	-27.6 (-69,61.8)

Iran (Islamic Republic of)	0.9 (0.2,1.5)	0.6 (0.1,0.8)	-36.3 (-57.3,4.8)	0.9 (0.2,1.4)	0.5 (0.1,0.6)	-51.7 (-67.9,-20)	34.8 (6.2,50.7)	13.7 (2.8,19.3)	-60.6 (-72.8,-27.3)
Iraq	2.6 (1.2,4.2)	2.6 (0.9,4)	1 (-42.9,69.2)	2.5 (1.2,4.1)	2.1 (0.7,3)	-17.8 (-52,38)	88.6 (39.9,149.7)	61.1 (19.8,93.9)	-31.1 (-63.6,18.2)
Jordan	1.7 (0.9,2.8)	0.6 (0.3,1.1)	-63.1 (-80.1,-25.4)	1.7 (0.9,2.8)	0.5 (0.3,0.9)	-70.2 (-84.1,-41.2)	45.7 (23.7,75.3)	11.2 (6,20.5)	-75.5 (-86.5,-47.2)
Kuwait	0 (0,0)	0.2 (0.1,0.2)	31085.9 (20298.1,48551.7)	0 (0,0)	0.1 (0.1,0.1)	21770.9 (14045.9,33496.7)	0 (0,0)	2.2 (1.6,3)	13885.4 (8176.8,22132.5)
Lebanon	0.8 (0.3,1.1)	0.4 (0.2,0.8)	-46.2 (-72.1,34.8)	0.8 (0.3,1.1)	0.3 (0.1,0.5)	-61.3 (-80.2,-2.8)	21 (7.6,32.4)	6.6 (2.5,11.7)	-68.3 (-83.9,-24.3)
Libya	0.6 (0.1,1.6)	0.4 (0.1,1.1)	-23.6 (-74.4,58.5)	0.5 (0.1,1.5)	0.3 (0.1,0.9)	-35.1 (-77.2,34.8)	18.7 (6.5,46.2)	11.4 (3.5,24)	-39 (-80,31.6)
Morocco	0.1 (0,0.2)	0.1 (0,0.1)	-29.7 (-68.3,34.4)	0.1 (0,0.2)	0.1 (0,0.1)	-37.1 (-70.5,19.1)	3.4 (1.6,7.4)	1.8 (0.4,3.6)	-48.1 (-80.9,5.8)
Palestine	0.6 (0.3,1.3)	0.4 (0.2,0.7)	-39.9 (-72.3,25)	0.6 (0.3,1.3)	0.3 (0.2,0.6)	-49.4 (-77.1,-0.1)	17.8 (9.1,36.1)	7.9 (4.9,14.5)	-55.7 (-79.3,-7.3)
Oman	0.2 (0.1,1)	0.1 (0,0.7)	-46 (-81.4,18.3)	0.2 (0.1,1)	0.1 (0,0.5)	-61 (-88,-20.6)	8.1 (3.2,30.9)	2.5 (0.8,11.4)	-69.7 (-89.5,-27.1)
Qatar	0.7 (0.3,1.3)	0.3 (0.1,0.6)	-54.2 (-79.5,-4.2)	0.7 (0.3,1.2)	0.2 (0.1,0.3)	-71.3 (-86.6,-43.2)	17.4 (7,31.7)	4.2 (2,7.5)	-75.9 (-89.2,-45.8)
Saudi Arabia	0.6 (0.2,1.9)	0.6 (0.3,1.9)	8.7 (-50.5,145)	0.6 (0.2,1.9)	0.4 (0.2,1.3)	-24 (-66.1,66.4)	18 (7.2,59.9)	12.3 (5.7,35.5)	-31.4 (-69.7,60.4)
Sudan	0.8 (0.3,2.4)	0.4 (0.1,0.8)	-50.1 (-86.3,37.9)	0.8 (0.3,2.5)	0.4 (0.1,0.7)	-54.9 (-87.7,20.5)	35.9 (10,93.5)	13.7 (5.3,24.8)	-61.7 (-91.2,21)
Syrian Arab Republic	0.7 (0.3,1.7)	0.4 (0.1,1.1)	-38.6 (-71.5,23)	0.7 (0.2,1.6)	0.3 (0.1,0.9)	-49.9 (-74.9,-3.2)	25.1 (11,50.9)	10.1 (2.4,21.7)	-59.8 (-82.8,-9.7)
Tunisia	0.3 (0.1,0.7)	0.2 (0,0.5)	-27.6 (-68.1,43.7)	0.3 (0.1,0.7)	0.1 (0,0.4)	-44.7 (-74.1,6.4)	9.2 (4,20.3)	4.2 (0.9,10.5)	-54.5 (-83.1,-6.1)
Turkey	0.7 (0.4,1.2)	0.3 (0.2,0.5)	-52.4 (-78.9,-4.6)	0.8 (0.4,1.3)	0.3 (0.2,0.4)	-62.7 (-84,-27.6)	21.6 (11.3,36)	6 (3.3,9.4)	-72.4 (-86.7,-41.7)
United Arab Emirates	1.4 (0.6,2.8)	1.1 (0.5,2)	-20.9 (-74.4,99.5)	1.4 (0.6,2.8)	1 (0.5,2)	-24.9 (-75.8,86.4)	36.5 (16.9,73.9)	18.6 (8.9,35.4)	-48.9 (-82.5,28.3)
Yemen	0.7 (0.3,2.1)	0.4 (0.1,0.9)	-33.4 (-75.2,39.9)	0.7 (0.3,2.2)	0.4 (0.1,0.9)	-37.6 (-76.1,33)	26.6 (8.8,72.9)	15.2 (4.2,28.4)	-43 (-83.4,35.4)
South Asia	0.5 (0.2,0.8)	0.3 (0.2,0.5)	-30.7 (-60,15.7)	0.5 (0.3,0.8)	0.3 (0.2,0.5)	-35.1 (-62.6,7.8)	17.4 (8.8,28.4)	9.7 (6.7,15.1)	-44.1 (-66.6,-1.7)

Bangladesh	0.5 (0.2,1)	0.3 (0.1,0.5)	-49.7 (-75.6,7.1)	0.5 (0.2,1.1)	0.2 (0.1,0.5)	-53.4 (-77.1,-2)	20 (8.2,37)	7.7 (3.7,15.4)	-61.6 (-84.2,-3.8)
Bhutan	0.5 (0.2,1.2)	0.3 (0.2,0.8)	-41.8 (-73.4,31.8)	0.6 (0.2,1.3)	0.3 (0.2,0.7)	-44.5 (-74.3,22.5)	20.3 (7.8,44.6)	9.2 (4,24)	-54.8 (-81.2,22.5)
India	0.5 (0.2,0.7)	0.3 (0.2,0.5)	-30.7 (-59.1,21.6)	0.5 (0.2,0.8)	0.3 (0.2,0.5)	-35.4 (-61.8,12.9)	16.8 (8.5,27.6)	9.1 (6.3,14.8)	-45.9 (-67.6,-0.9)
Nepal	0.6 (0.2,1.3)	0.4 (0.2,0.8)	-33.8 (-65.5,34.7)	0.6 (0.2,1.5)	0.4 (0.2,0.8)	-35.8 (-65.5,27.1)	20.8 (8,45.5)	10.8 (4.9,24.5)	-48.2 (-78.3,13.4)
Pakistan	0.5 (0.2,0.9)	0.5 (0.2,0.7)	-10.4 (-59,49.2)	0.5 (0.3,1)	0.5 (0.3,0.8)	-12.6 (-60.1,44.4)	18.3 (8.7,32.2)	15.2 (7.8,25.1)	-17 (-61.2,41.4)
Southern Sub-Saharan Africa	0 (0,0.1)	0 (0,0.1)	-23.7 (-64.2,40.5)	0.1 (0,0.1)	0 (0,0.1)	-27.6 (-67.6,39.1)	1.8 (0.8,4.2)	1.2 (0.6,2.2)	-32.5 (-64.9,19.2)
Botswana	0.1 (0,0.1)	0 (0,0.1)	-37.5 (-73.9,36.1)	0.1 (0,0.2)	0 (0,0.1)	-39.2 (-75,34.2)	2 (0.6,5.5)	1.1 (0.3,3.2)	-42.4 (-74.1,34.5)
Lesotho	0 (0,0.1)	0.1 (0,0.1)	47.9 (-55.9,287.9)	0 (0,0.1)	0.1 (0,0.1)	43.3 (-56.3,276.5)	1.3 (0.4,3.5)	2 (0.7,3.4)	49.3 (-52.2,287.7)
Namibia	0 (0,0.1)	0 (0,0.1)	-22.4 (-63.4,77.2)	0 (0,0.1)	0 (0,0.1)	-26.4 (-63.7,64.7)	1.4 (0.4,3.6)	1 (0.3,2.2)	-30.7 (-68,58.6)
South Africa	0 (0,0.1)	0 (0,0.1)	-36.3 (-69.9,26.1)	0 (0,0.1)	0 (0,0.1)	-39.9 (-73.5,22.1)	1.9 (0.8,4.3)	0.9 (0.5,1.7)	-49.8 (-73.8,1.1)
Eswatini	0.1 (0,0.2)	0.1 (0,0.1)	-11.6 (-64.7,98.2)	0.1 (0,0.2)	0.1 (0,0.1)	-14.5 (-67.6,98.1)	2.2 (0.7,5.4)	1.9 (0.7,3.8)	-15.6 (-64.5,96.4)
Zimbabwe	0.1 (0,0.1)	0.1 (0,0.1)	26.7 (-54.7,201.4)	0.1 (0,0.1)	0.1 (0,0.1)	20.5 (-56,193.5)	1.8 (0.5,4.5)	2.4 (0.8,5)	39.6 (-48.5,226.5)
Western Sub-Saharan Africa	0.1 (0,0.2)	0 (0,0.1)	-33.1 (-66.9,17.8)	0.1 (0,0.2)	0 (0,0.1)	-34.5 (-66.9,16.5)	3 (1,6.9)	1.6 (0.7,3.9)	-45.8 (-78.3,11.1)
Benin	0.1 (0,0.2)	0 (0,0.1)	-25.8 (-68.5,55.4)	0.1 (0,0.2)	0 (0,0.1)	-27.6 (-66.1,50.2)	3 (0.8,7.8)	1.9 (0.6,5.2)	-37.8 (-79.4,47)
Burkina Faso	0.1 (0,0.3)	0.1 (0,0.2)	-18.2 (-63.2,65.2)	0.1 (0,0.3)	0.1 (0,0.2)	-19.3 (-62.6,61.5)	4 (1.1,12.1)	2.9 (1.1,8.9)	-28.3 (-75.6,47.2)
Cameroon	0.1 (0,0.2)	0.1 (0,0.1)	-19.2 (-57.9,88.3)	0.1 (0,0.2)	0.1 (0,0.1)	-22 (-58.3,81.2)	3 (1,7.3)	2.1 (0.8,4.3)	-30.5 (-67.5,65)
Cabo Verde	0.1 (0,0.5)	0.1 (0,0.3)	-16.2 (-60.2,91.2)	0.1 (0,0.5)	0.1 (0,0.3)	-23.6 (-64.5,80.7)	4.8 (1.1,17.5)	2.8 (0.6,10.1)	-42.7 (-72.8,43.5)
Chad	0.1 (0,0.2)	0.1 (0,0.2)	10.8 (-39.7,112.5)	0.1 (0,0.2)	0.1 (0,0.2)	8.8 (-38.9,112.4)	2.9 (0.6,7.7)	2.8 (0.9,7.3)	-3.3 (-56.7,88)

CÃ´te d'Ivoire	0 (0,0.1)	0 (0,0.1)	-19.4 (-57.3,47.8)	0 (0,0.1)	0 (0,0.1)	-23.5 (-59.4,43.4)	1.8 (0.6,4.9)	1.3 (0.4,3.5)	-28.2 (-66,37.5)
Gambia	0 (0,0.1)	0 (0,0.1)	-19.9 (-69.3,42.6)	0 (0,0.1)	0 (0,0.1)	-22.1 (-69,39)	1.4 (0.4,3.5)	0.9 (0.3,2.8)	-35.4 (-82.4,37)
Ghana	0.1 (0,0.2)	0 (0,0.1)	-62 (-84.4,-8.9)	0.1 (0,0.2)	0 (0,0.1)	-62.8 (-85.3,-11.7)	4.3 (1.9,9.4)	1.4 (0.4,4.9)	-68.2 (-88.1,-19.8)
Guinea	0 (0,0.1)	0 (0,0.1)	-40.9 (-83.5,29.4)	0 (0,0.1)	0 (0,0.1)	-42.3 (-83.4,24.8)	1.7 (0.4,4.8)	0.8 (0.2,2.2)	-54.6 (-89.8,23.7)
Guinea-Bissau	0.1 (0,0.3)	0.1 (0,0.2)	-18.9 (-60.8,60)	0.1 (0,0.3)	0.1 (0,0.2)	-19.5 (-60.4,58.4)	4.6 (1.3,11.8)	3.1 (1.1,7.8)	-33.2 (-76.8,38.3)
Liberia	0.1 (0,0.2)	0.1 (0,0.2)	-26.7 (-66.2,54.5)	0.1 (0,0.2)	0.1 (0,0.1)	-30.1 (-66.6,44.1)	4.2 (1.3,9.9)	2.3 (0.9,5.5)	-45.5 (-82.1,38)
Mali	0.1 (0,0.3)	0.1 (0,0.2)	-44.8 (-83.7,8.8)	0.1 (0,0.3)	0.1 (0,0.2)	-45.4 (-83.3,8)	4.9 (1.1,12.6)	2.1 (0.5,6.4)	-56.6 (-90.2,5.8)
Mauritania	0.1 (0,0.2)	0 (0,0.1)	-30.7 (-64.8,44.8)	0.1 (0,0.2)	0 (0,0.1)	-36 (-67,32.9)	2.1 (0.6,5.7)	1.2 (0.4,3.1)	-43.3 (-72.2,29)
Niger	0.1 (0,0.2)	0.1 (0,0.2)	-33 (-83.4,46.2)	0.1 (0,0.2)	0.1 (0,0.2)	-33.5 (-82.1,45.2)	4.4 (0.9,13.9)	2.2 (0.5,6.1)	-51.2 (-89.8,25.1)
Nigeria	0.1 (0,0.1)	0 (0,0.1)	-36.2 (-72.8,39.8)	0.1 (0,0.1)	0 (0,0.1)	-37.5 (-72.6,32.5)	2.6 (0.8,7.2)	1.3 (0.5,3.1)	-49.6 (-80.8,30.9)
Sao Tome and Principe	0.1 (0,0.2)	0 (0,0.1)	-22.3 (-64.4,55.6)	0.1 (0,0.2)	0 (0,0.1)	-28.3 (-68,44.6)	2.4 (0.8,6.7)	1.4 (0.4,4.4)	-42.2 (-78.9,26.4)
Senegal	0.1 (0,0.2)	0 (0,0.1)	-21.2 (-67.1,55.5)	0.1 (0,0.2)	0 (0,0.1)	-23.9 (-67.9,48.9)	2.7 (0.6,7.2)	1.7 (0.5,5.2)	-38.9 (-82.5,35.1)
Sierra Leone	0.1 (0,0.2)	0.1 (0,0.2)	-14.9 (-68.1,78.6)	0.1 (0,0.2)	0.1 (0,0.2)	-17.6 (-69.4,70.2)	3.6 (1,10.3)	2.4 (0.8,5.7)	-33.1 (-81.3,70.3)
Togo	0.1 (0,0.2)	0.1 (0,0.1)	-16.2 (-53.9,68)	0.1 (0,0.2)	0.1 (0,0.1)	-19.1 (-53.5,59.8)	2.7 (0.8,6.6)	1.9 (0.6,4.7)	-30.8 (-69.4,53.2)
Eastern Sub-Saharan Africa	0.2 (0.1,0.6)	0.1 (0.1,0.4)	-38.1 (-69.6,13.6)	0.2 (0.1,0.7)	0.1 (0.1,0.4)	-39.4 (-69.7,9.9)	9.1 (3.4,22.9)	5 (1.9,14.9)	-44.6 (-76.8,10)
Burundi	0.2 (0.1,0.7)	0.1 (0,0.4)	-32.5 (-67,55.3)	0.2 (0.1,0.7)	0.1 (0,0.5)	-33.7 (-66.7,51.7)	8.4 (2.8,28.1)	5.2 (1.8,15.9)	-38.3 (-70.7,48)
Comoros	0.1 (0,0.4)	0.1 (0,0.3)	-23.3 (-59.9,46.2)	0.1 (0,0.4)	0.1 (0,0.3)	-24.9 (-60,40.5)	5.5 (1.8,13.5)	3.9 (1.3,10.6)	-30 (-63.9,44.5)
Djibouti	0.1 (0,0.3)	0.1 (0,0.3)	-13 (-55.3,58.1)	0.1 (0,0.4)	0.1 (0,0.3)	-16.8 (-55.9,49.9)	4 (1.2,12.4)	3.1 (0.8,10.3)	-21.6 (-60.8,53)

Eritrea	0.2 (0.1,0.4)	0.1 (0,0.4)	-14.9 (-57.9,77.1)	0.2 (0.1,0.4)	0.1 (0,0.4)	-15.4 (-57.5,74.8)	6.6 (2.2,14.7)	5.1 (1.6,13.1)	-22.6 (-62.5,61.4)
Ethiopia	0.5 (0.1,1.4)	0.2 (0.1,0.8)	-53.2 (-84.9,29)	0.5 (0.1,1.5)	0.2 (0.1,0.8)	-54.5 (-85.2,22.1)	18.7 (5.3,48.7)	7.8 (2.3,25.8)	-58.1 (-88.4,33.3)
Kenya	0.1 (0.1,0.4)	0.1 (0.1,0.3)	-13.8 (-50.4,62.5)	0.2 (0.1,0.4)	0.1 (0.1,0.3)	-16.6 (-52.7,56.4)	5.3 (2.4,14.8)	4 (1.7,8.9)	-23.7 (-55.4,55.5)
Madagascar	0.1 (0.1,0.3)	0.1 (0,0.2)	-24.9 (-53.6,31.7)	0.1 (0.1,0.3)	0.1 (0,0.2)	-27.3 (-55.3,28.1)	5.3 (2.1,11.6)	3.7 (1.4,7.8)	-30.2 (-58.4,31.3)
Malawi	0.1 (0,0.1)	0 (0,0.1)	-33.1 (-65.3,23.4)	0.1 (0,0.2)	0 (0,0.1)	-35 (-65.4,18.4)	2.2 (0.8,5.6)	1.3 (0.4,3.4)	-42.2 (-74,16.1)
Mozambique	0.2 (0.1,0.6)	0.2 (0,0.4)	-23.8 (-60.6,28.8)	0.2 (0.1,0.6)	0.2 (0.1,0.5)	-24.1 (-60.4,30.9)	7.4 (2.4,19.3)	5.1 (1.5,16)	-31.3 (-70.5,16.7)
Rwanda	0.3 (0.1,0.7)	0.1 (0,0.5)	-48.3 (-79.9,13)	0.3 (0.1,0.7)	0.1 (0,0.5)	-49.2 (-80.2,9.3)	10.5 (4.3,24.9)	4.7 (1.3,14.9)	-55.6 (-84.8,7)
Somalia	0.2 (0,0.5)	0.2 (0,0.6)	-2.6 (-47.1,61.4)	0.2 (0,0.5)	0.2 (0,0.6)	-4.7 (-46.6,56.5)	6.6 (1.7,18.9)	5.9 (1.3,21.4)	-10.6 (-53.1,52.6)
South Sudan	0.1 (0,0.4)	0.1 (0,0.4)	7.8 (-37.2,80.4)	0.1 (0,0.4)	0.1 (0,0.4)	3.3 (-37.3,70.8)	4.9 (1.4,13.8)	5.1 (1.3,14.6)	4.7 (-39.1,88.2)
United Republic of Tanzania	0.2 (0.1,0.5)	0.1 (0,0.4)	-22.9 (-54.9,46.6)	0.2 (0.1,0.5)	0.1 (0,0.4)	-25.2 (-57.1,41.5)	6.1 (2.3,17.8)	4.3 (1.4,12.1)	-29.4 (-60.8,38.6)
Uganda	0.1 (0,0.3)	0.1 (0,0.3)	12.3 (-46.8,136.8)	0.1 (0,0.3)	0.1 (0,0.3)	8.8 (-50.1,131.2)	3 (1.1,9.7)	3.2 (0.8,10.1)	7.8 (-52.6,114.5)
Zambia	0.2 (0.1,0.4)	0.1 (0,0.3)	-38 (-75.7,36.3)	0.2 (0.1,0.4)	0.1 (0,0.3)	-39.3 (-75,30)	6.7 (3.1,14.4)	3.7 (1.1,9.9)	-44.7 (-80.5,31.2)
Central Sub-Saharan Africa	0.1 (0,0.3)	0.1 (0,0.2)	-31.3 (-68.1,26.3)	0.1 (0,0.3)	0.1 (0,0.2)	-32.3 (-68.3,21)	5.2 (1.7,11.9)	3 (1,8)	-42.4 (-79.2,23.2)
Angola	0.1 (0,0.3)	0.1 (0,0.3)	-33 (-81.2,45.1)	0.1 (0,0.3)	0.1 (0,0.3)	-33.8 (-79.5,39.2)	6.3 (1.7,16.5)	3.3 (1.1,9.2)	-47 (-89,37)
Central African Republic	0.2 (0.1,0.4)	0.1 (0,0.3)	-30.3 (-64.5,26)	0.2 (0.1,0.4)	0.1 (0,0.3)	-31 (-63.1,24.5)	7 (2.1,16.1)	4.5 (1.1,11.4)	-36.1 (-69.4,30.8)
Congo	0.1 (0.1,0.4)	0.1 (0,0.3)	-27.4 (-61.3,55.9)	0.1 (0.1,0.4)	0.1 (0,0.3)	-30.8 (-63,48.6)	5.1 (2.1,13.3)	3.3 (1.6,9.2)	-35.1 (-67.2,45.7)
Democratic Republic of the Congo	0.1 (0,0.3)	0.1 (0,0.2)	-30.7 (-66.9,34.3)	0.1 (0,0.3)	0.1 (0,0.2)	-31.3 (-65.7,29.2)	4.8 (1.5,11.3)	2.8 (0.8,7.8)	-41.9 (-78.2,22.7)

Equatorial Guinea	0.1 (0,0.3)	0.1 (0,0.2)	-45.4 (-81.1,61)	0.1 (0,0.4)	0.1 (0,0.2)	-50.3 (-82.7,41.5)	5.3 (1.6,12.4)	2.2 (0.7,5.5)	-58 (-86,34.1)
Gabon	0.1 (0,0.3)	0.1 (0,0.2)	-37.7 (-67.2,25.1)	0.1 (0,0.4)	0.1 (0,0.2)	-41.6 (-70.2,17)	4.4 (1.5,11)	2.4 (0.8,5.8)	-46.2 (-71.3,18.5)
