# Supplementary material for: Global burden of leukemia in women of child-bearing age, 1990 to 2021: An update from the Global Burden of Disease Study 2021
Source: Medicine (Baltimore). 2026 Jan 23;105(4):e47217. doi: 10.1097/MD.0000000000047217 (PMC12851764; doi:10.1097/MD.0000000000047217)

Figure S1 National age-standardized incidence, deaths, and DALY rates in 2021, and the percentage change in the age-standardized rates from 1990 to 2021 for ALL among WCBA (15–49 years). Age-standardized rates of incidence (A), deaths (B), and DALYs (C). The percentage change in the age-standardized rates from 1990 to 2021 of incidence (D), deaths (E), and DALYs (F).

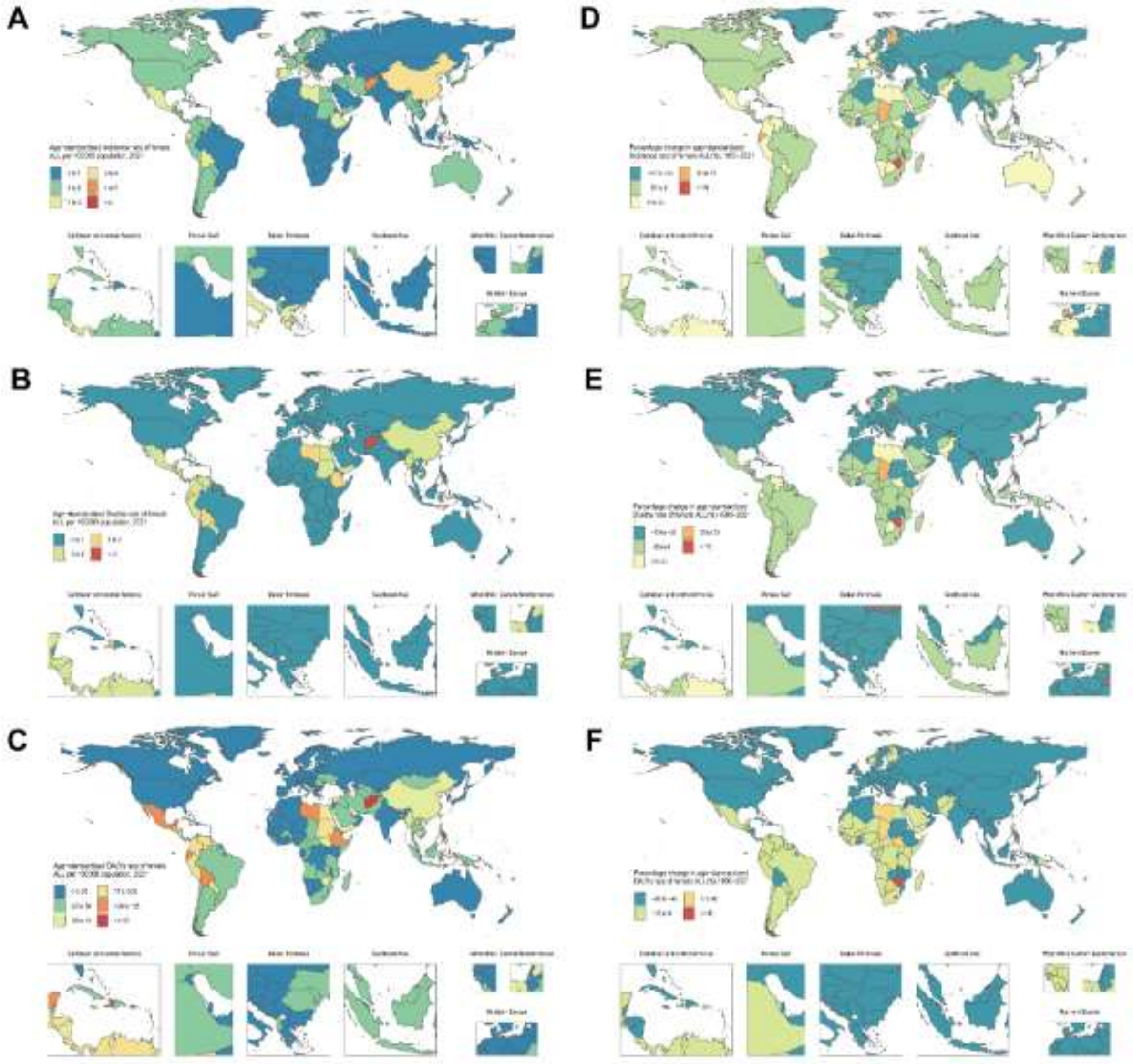

Figure S2 National age-standardized incidence, deaths, and DALY rates in 2021, and the percentage change in the age-standardized rates from 1990 to 2021 for AML among WCBA (15–49 years). Age-standardized rates of incidence (A), deaths (B), and DALYs (C). The percentage change in the age-standardized rates from 1990 to 2021 of incidence (D), deaths (E), and DALYs (F).

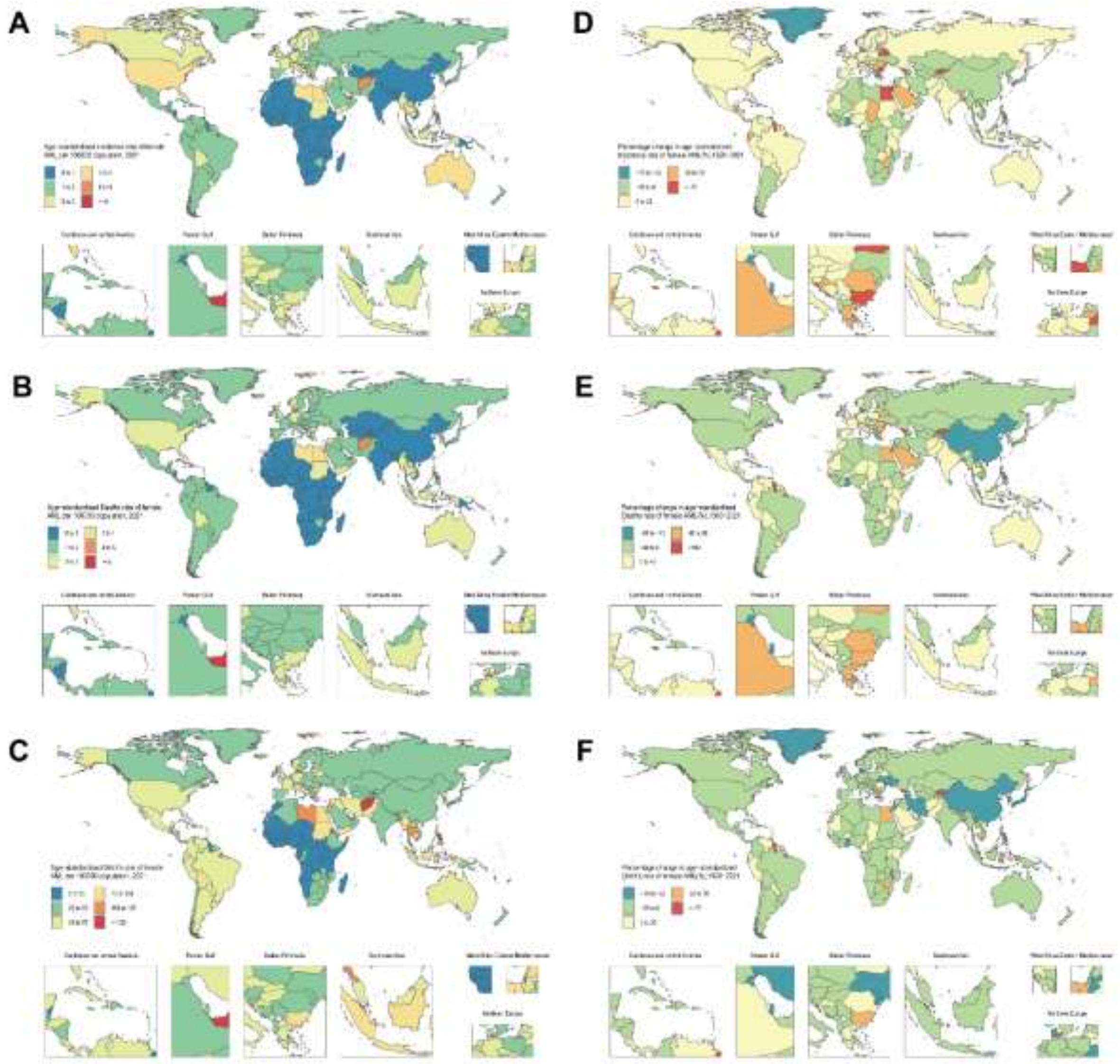

Figure S3 National age-standardized incidence, deaths, and DALY rates in 2021, and the percentage change in the age-standardized rates from 1990 to 2021 for CLL among WCBA (15–49 years). Age-standardized rates of incidence (A), deaths (B), and DALYs (C). The percentage change in the age-standardized rates from 1990 to 2021 of incidence (D), deaths (E), and DALYs (F).

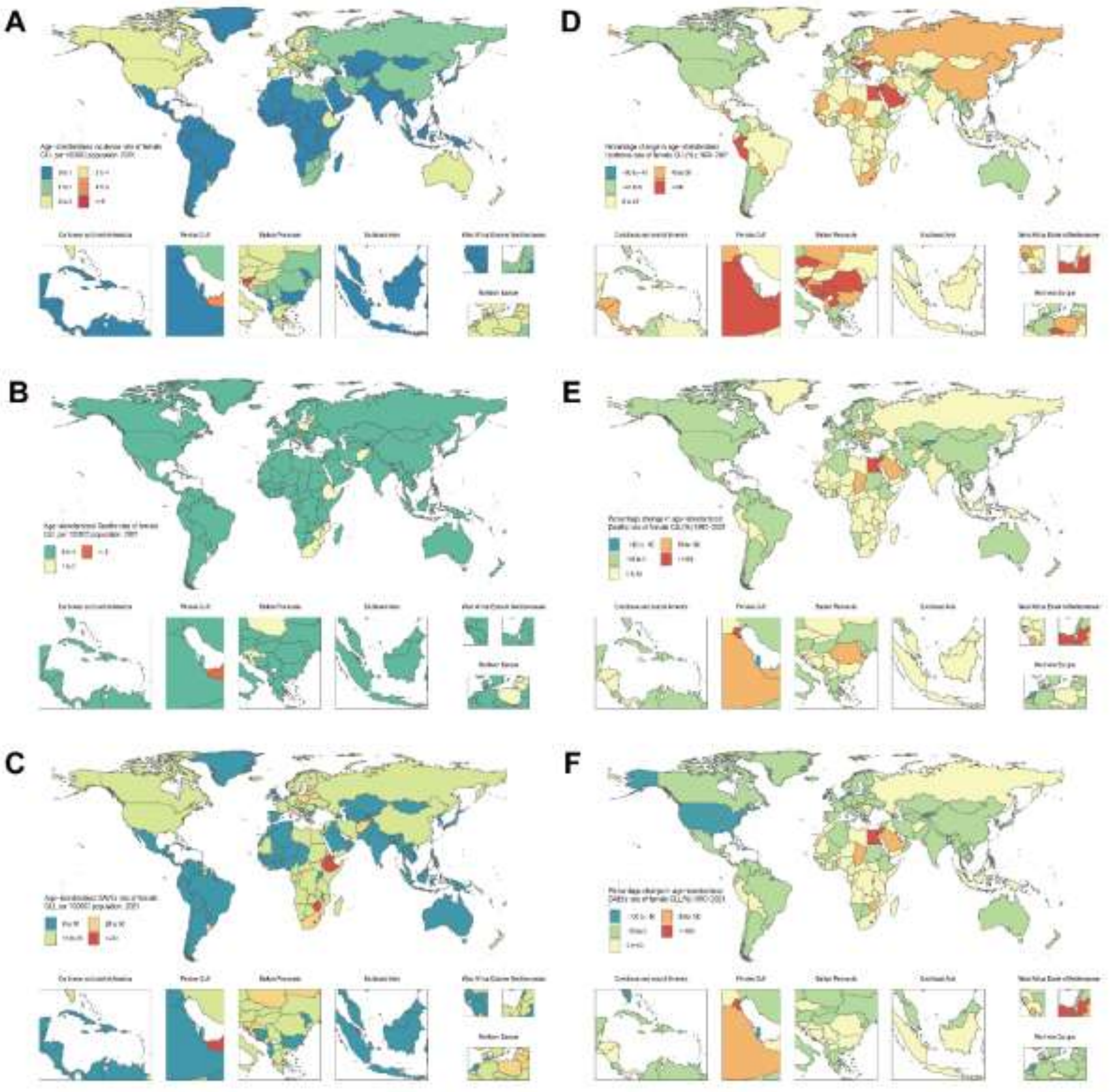

Figure S4 National age-standardized incidence, deaths, and DALY rates in 2021, and the percentage change in the age-standardized rates from 1990 to 2021 for CML among WCBA (15–49 years). Age-standardized rates of incidence (A), deaths (B), and DALYs (C). The percentage change in the age-standardized rates from 1990 to 2021 of incidence (D), deaths (E), and DALYs (F).

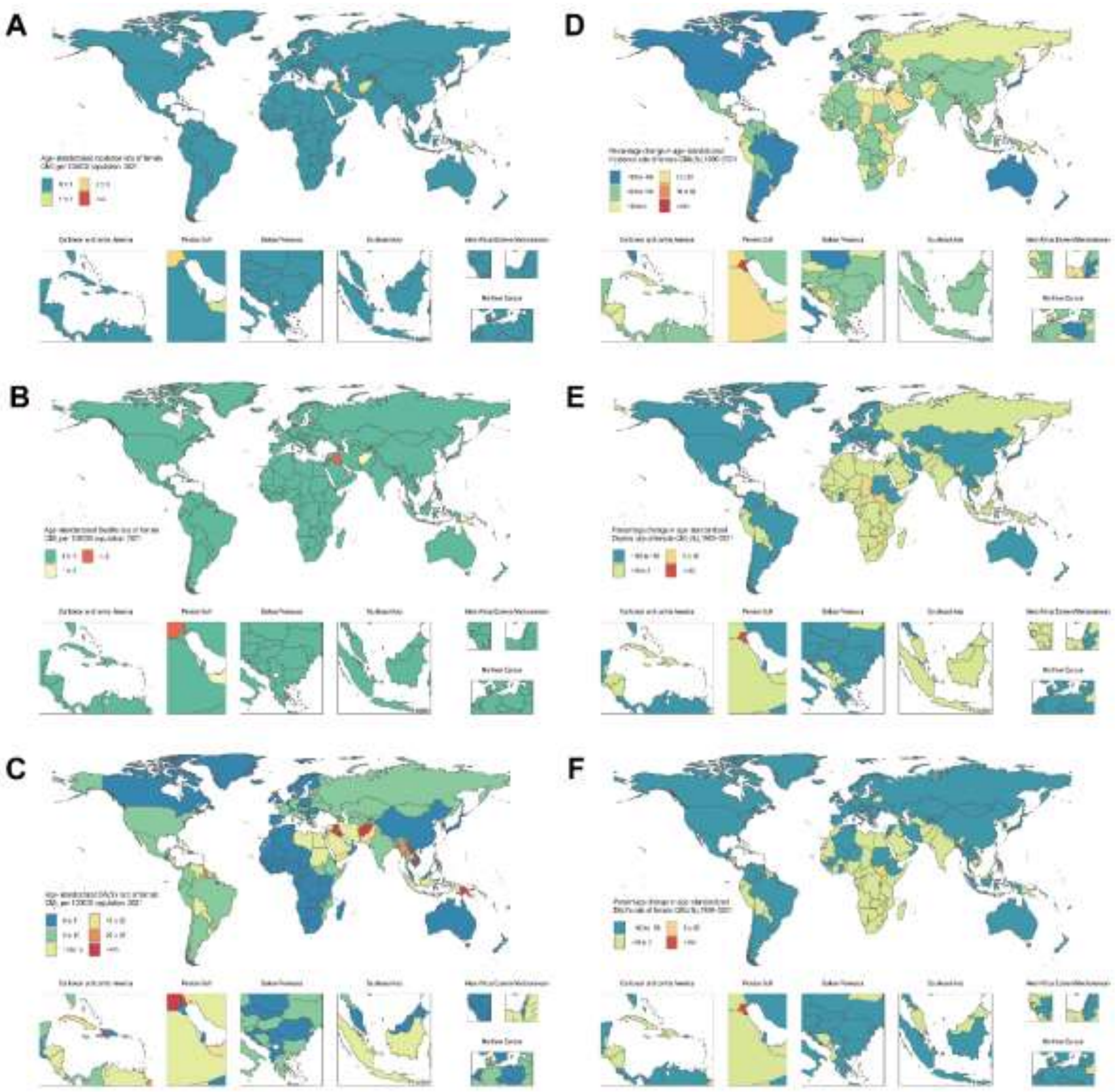

Figure S5 Age-standardized rates of incidence, deaths, and DALYs of leukemia in WCBA (15–49 years), globally and for 21 GBD regions, by SDI (2021), from 1990 to 2021. Age-standardized incidence rates (A), death rate (B), and DALY rate (C) of leukemia in WCBA (15–49 years), by SDI in 2021.

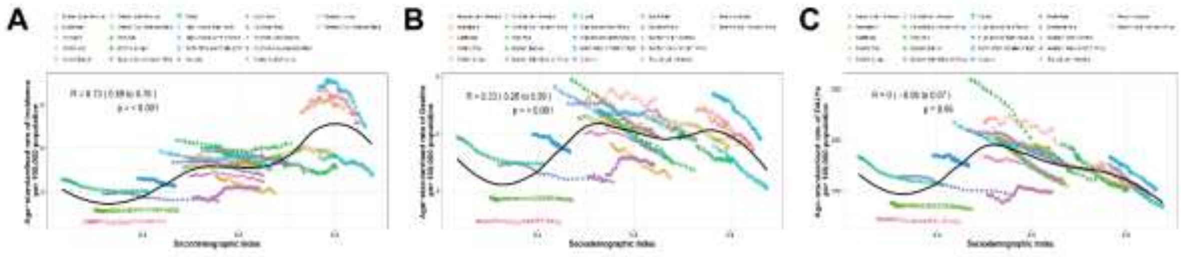

Figure S6 The percentage of deaths (A) and DALYs (B) due to ALL among WCBA (15–49 years) attributable to each risk factor for the 21 global burden of disease regions in 2021. The percentage of deaths (C) and DALYs (D) due to ALL among WCBA (15–49 years) attributable to each risk factor, by age, in 2021.

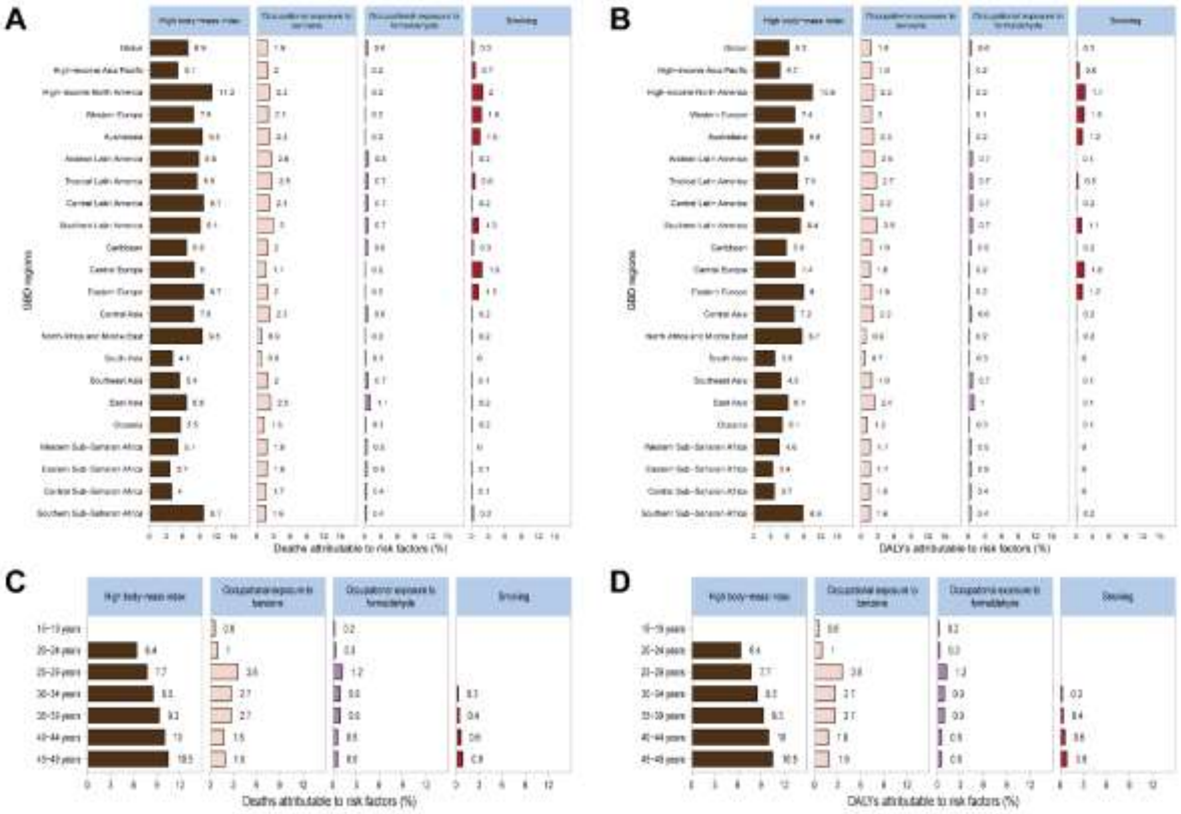

Figure S7 The percentage of deaths (A) and DALYs (B) due to AML among WCBA (15–49 years) attributable to each risk factor for the 21 global burden of disease regions in 2021. The percentage of deaths (C) and DALYs (D) due to AML among WCBA (15–49 years) attributable to each risk factor, by age, in 2021.

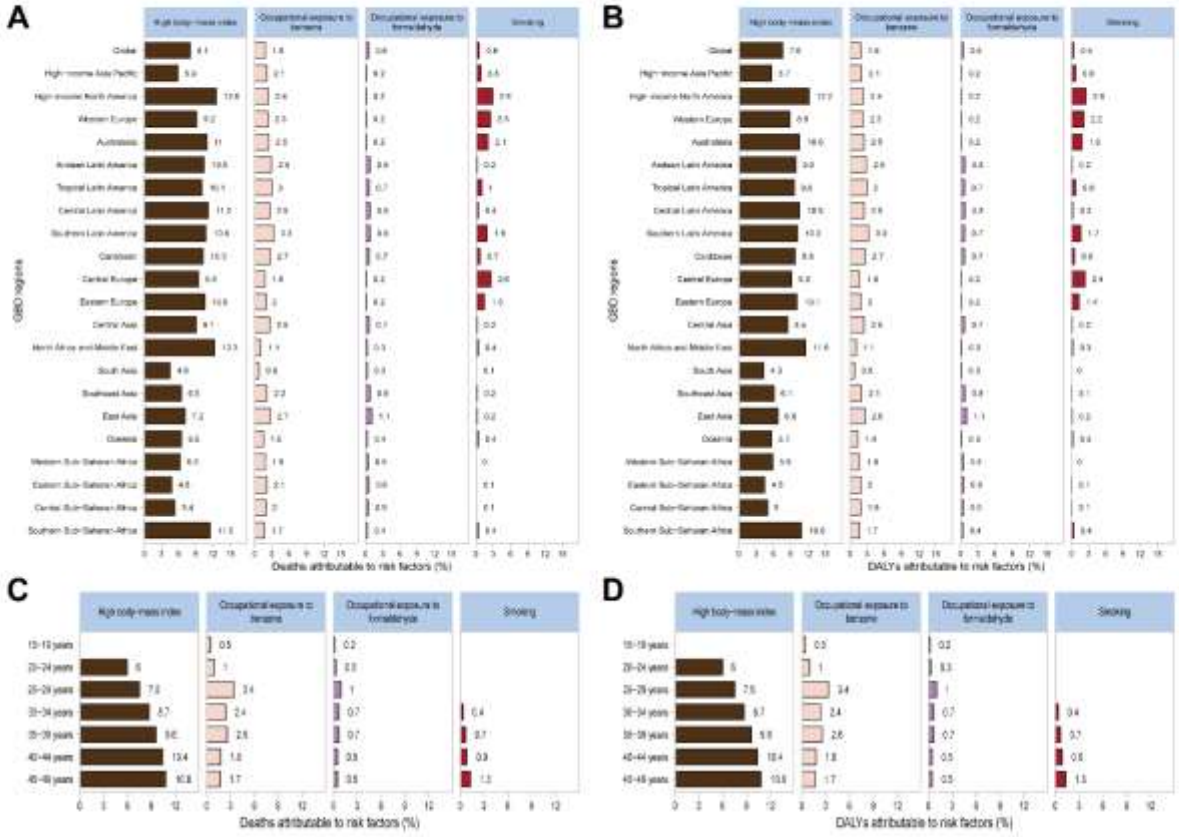

Figure S8 The percentage of deaths (A) and DALYs (B) due to CLL among WCBA (15–49 years) attributable to each risk factor for the 21 global burden of disease regions in 2021. The percentage of deaths (C) and DALYs (D) due to CLL among WCBA (15–49 years) attributable to each risk factor, by age, in 2021.

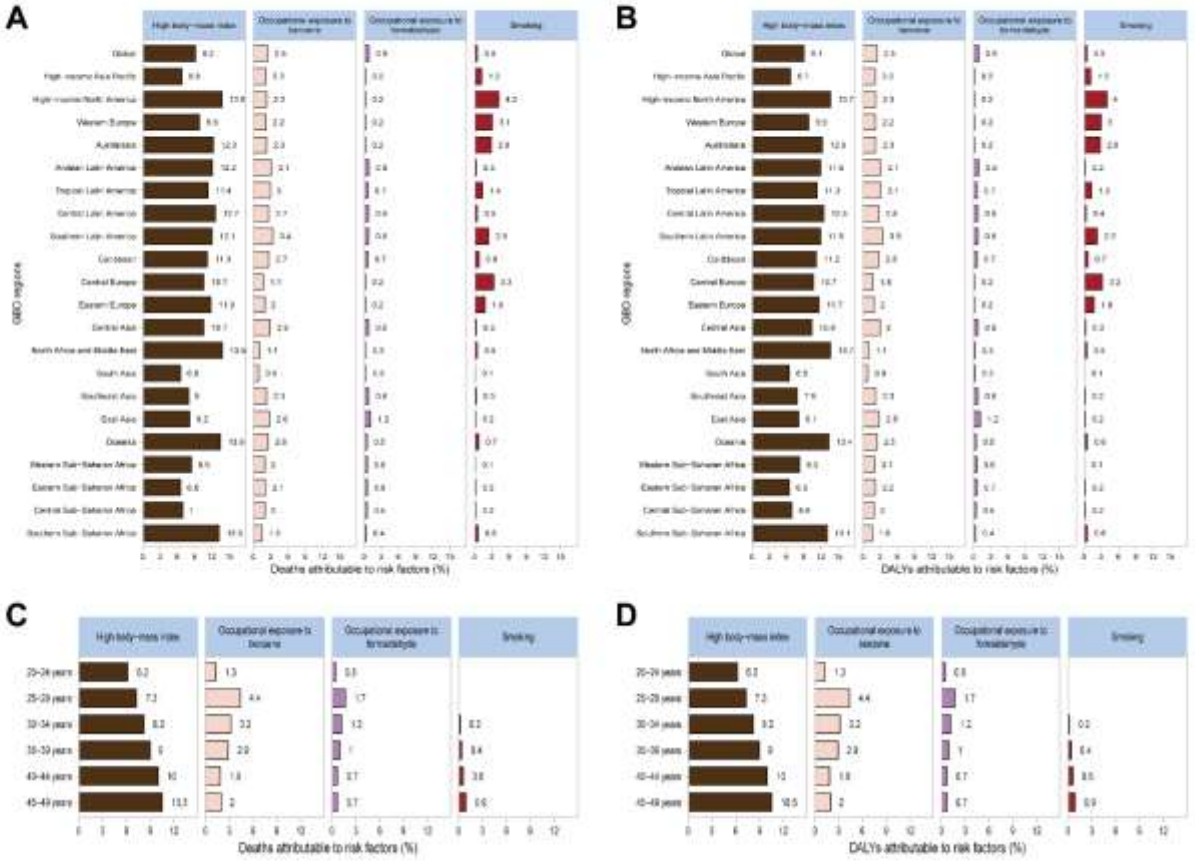

Figure S9 The percentage of deaths (A) and DALYs (B) due to CML among WCBA (15–49 years) attributable to each risk factor for the 21 global burden of disease regions in 2021. The percentage of deaths (C) and DALYs (D) due to CML among WCBA (15–49 years) attributable to each risk factor, by age, in 2021.

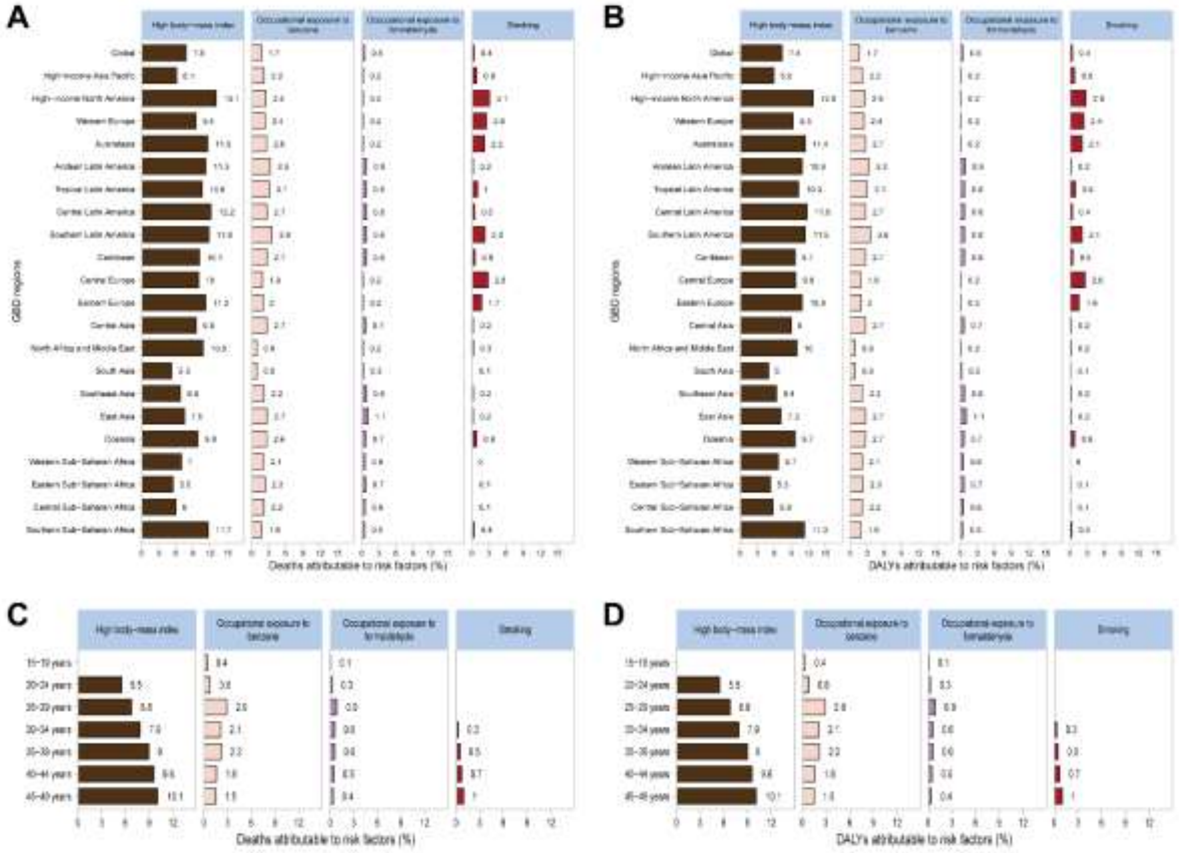

Supplement: Supplementary file 2 [file medi-105-e47217-s002.pdf]
